# Collected data on bending, vibration, and push-out tests of shallow steel-timber composite beams—Nordic system

**DOI:** 10.1016/j.dib.2024.111172

**Published:** 2024-11-26

**Authors:** Aku Aspila, Markku Heinisuo, Virpi Leivo, Mikko Malaska, Kristo Mela, Sami Pajunen, Mika Vuorela

**Affiliations:** Tampere University, Faculty of Built Environment, P.O. Box 600, FI-33014 Tampere, Finland

**Keywords:** WQ-beam, CLT, Hybrid floor, Shallow-floor, Slim-floor, Structural performance, Composite action, Shear force

## Abstract

In a slim-floor structural system, beams and slabs are placed at the same level, reducing the overall floor height and material usage in vertical structures, thereby improving economic efficiency. The use of slim-floor structures is common practice in Finnish construction where these structures are typically constructed using hollow-concrete slabs and welded steel box beams. However, in Finland, only a few buildings utilise cross-laminated timber (CLT) slabs in slim-floor structures, and none have incorporated the composite action between CLT and steel beams. This paper presents the laboratory test results of combining CLT slabs and asymmetric welded box beams (WQ-beams) to further assess their potential in optimising structural performance. The structural behaviour of the test construction was evaluated for serviceability and ultimate limit states. The testing involved six full-scale specimens to observe structural responses by measuring total jack forces, deflections, strains, and slip between timber and steel. Vibration tests were conducted on four specimens, capturing deflection and acceleration during heel-drop and walking tests. Additionally, push-out tests were performed to determine connection properties of the wood screws by measuring jack force and displacements. The same skilled faculty team meticulously carried out all tests in the Structural Behaviour Laboratory at Tampere University in Finland. The comprehensive datasets are presented in Excel sheets along with illustrative graphs. Furthermore, the report contains images taken during and after the tests. The provided dataset can serve construction companies, product developers, and researchers aiming to deepen their understanding of the structural behaviour of steel-timber slim-floor composite structures. Additionally, it can be utilised as a reliable reference to validate finite element models (FEM) and analytical calculation models, and to aid in the development of design guides or standards.

Specifications Table

**Table:** 

Subject	*Civil and Structural Engineering*
Specific subject area	Structural behaviour of slim-floor steel-timber composite floor under full-scale bending and vibration tests. Shear force between wood screws and CLT panels.
Type of data	Raw and Filtered data, Tables, Images, Graphs and Figures
Data collection	Full-scale bending test: - The deflection of the specimens was measured by using a displacement transducer (Novotechnik, type TRS-0100). - The force in the hydraulic jack was measured directly through the sensors of the jacks themselves. Also, the contact force at one end of the steel beam was recorded by using the load transducer. - Strains of the specimens were recorded by using wood and steel strain gages from Kyowa. Testing setup used Quarter-bridge system (1-gage system) ○ Steel: Kyowa, KFGS-5-120-C1-11, length 5 mm, gage factor 2.09 ○ Wood: Kyowa KFGS-30-120-C1-5, length 30 mm, gage factor 2.09 Vibration test: - The deflection of the specimens was measured by using a displacement transducer (Novotechnik, type TRS-0100). - Acceleration of the platform was recorded by using triaxial acceleration monitor from TE connectivity (Model 4030-002, ±6g range with a nominal 0–200 Hz bandwidth) Push-Out tests: - The force in the hydraulic jack was measured directly through the sensors of the jacks themselves. - The displacement of the specimens was measured by using a displacement transducer (Novotechnik, type TRS-0100). Jacks: Interface, Model 1032-AF 250 kN SN. 27,820, force range ± 250 kN. Calibrated according to SFS-EN ISO 7500-1, accuracy class 1
Data source location	Tampere University, Faculty of Built Environment, P.O. Box 600, FI-33014 Tampere, Finland
Data accessibility	Repository name: Zenodo Data identification number: 10.5281/zenodo.13751932 Direct URL to data: https://zenodo.org/records/13751932
Related research article	*None.*

## 1 Value of the Data

• The dataset offers insights into the composite action potential between cross-laminated timber (CLT) slabs and asymmetric welded box beams (WQ-beam), providing valuable information for analysing their impact on structural performance.
• The dataset, derived from full-scale and push-out tests conducted under consistent conditions by a dedicated team at Tampere University, ensures reliability, comparability, and data accuracy across the experiments.
• Multiple tests using the same dimensions and test setups enhance the datasetʼs reliability and enable construction companies, product developers, and researchers to comprehend the mechanical performance of steel-timber slim-floor composite structures.
• The dataset serves as a valuable reference for validating finite element models (FEM) and analytical calculations, aiding in the formulation of design standards and guides for such structural systems.
• Provided in Excel format with accompanying Python code-generated graphs, the dataset allows for convenient analysis and potential integration into various software applications, enhancing its usability and applicability.
• By capturing structural nuances and behaviours through detailed data and visual documentation, this dataset facilitates a comprehensive exploration and utilization of steel-timber slim-floor composite structures in diverse engineering contexts.

## 2 Background

This dataset is part of the ongoing research and part of the PhD thesis that is formulating an analytical calculation method for the steel-timber composite floor utilising slim-floor design. The presented dataset works as a reference point to validate the FEM and analytical model.

## 3 Data Description

Provided datasets are deviated into three main categories based on the test configuration:

• Full-scale
• Push-out
• Vibration

Each folder contains test specific Excel files, graphs and photographs that capture the behaviour of the specimens before, during and after the tests. Excel files are containing both raw and organised data from individual tests, such as bending, or groups of tests, such as vibration and push-out. In the case of vibration and push-out test, Excel files are containing graphs but because of the vastness of the data presented in the full-scale tests the data is processed to visual format by using Python code. In all cases the raw data is obtained directly from the recording device and then converted into a more comprehensive format for analysis.

### 3.1 Full-scale bending

The datasets from the full-scale tests are divided into twelve different folders:

• Matrix-Displacement
• Matrix-Strain bottom
• Matrix-Strain top
• Test-1
• Test-2
• Test-3 with connectors
• Test-3 without connectors
• Test-4 first with connectors
• Test-4 second with connectors
• Test-4 without connectors
• Test-5
• Test-6

The first three folders contain matrix graphs that show how the different tests compared to each's other. The graphs in the folders adopt the notation given in Fig. 17, Fig. 18, Fig. 19, Fig. 20. Test specific folders are containing photos taken during and after the testing, matrix graphs from the test and Excel file labelled based on the test number and the use of connectors between CLT and WQ-beam. Table 1 gives a summary of the conducted test and the variation between them. For example, the file “Full-scale-3-without connectors” contains data from the third full-scale test without connector plates between CLT and WQ-beam. In the case of tests 3 and 4 the pictures are stored on the first folders. In the Excel files, first sheet is a info page of the test, second presents the raw dataset, and subsequent sheets displaying organised data. To improve the readability, the datasets in the organised sheets are arranged to mimic the location of the sensors. For the full-scale tests, 2, 3 and 5, a Digital Image Correlation (DIC) method was used to capture the vertical and horizontal movements of the platform, but these datasets are excluded from this paper.

**Table 1:** Summary of the conducted Full-scale tests and their modifications.

	Test-1	Test-2	Test-3	Test-4	Test-4.1	Test-5	Test-6
Span length of CLT [m]	4	4	4	2.6	2.6	2.6	2.6
Loading line [m]	1.2	1.2	1.2	1.2	0.4	0.4	0.4
Connectors	x	X	x	x	x	x	x
No Connectors	–	–	x	x		–	–

### 3.2 Push-out

**Fig. 1:**
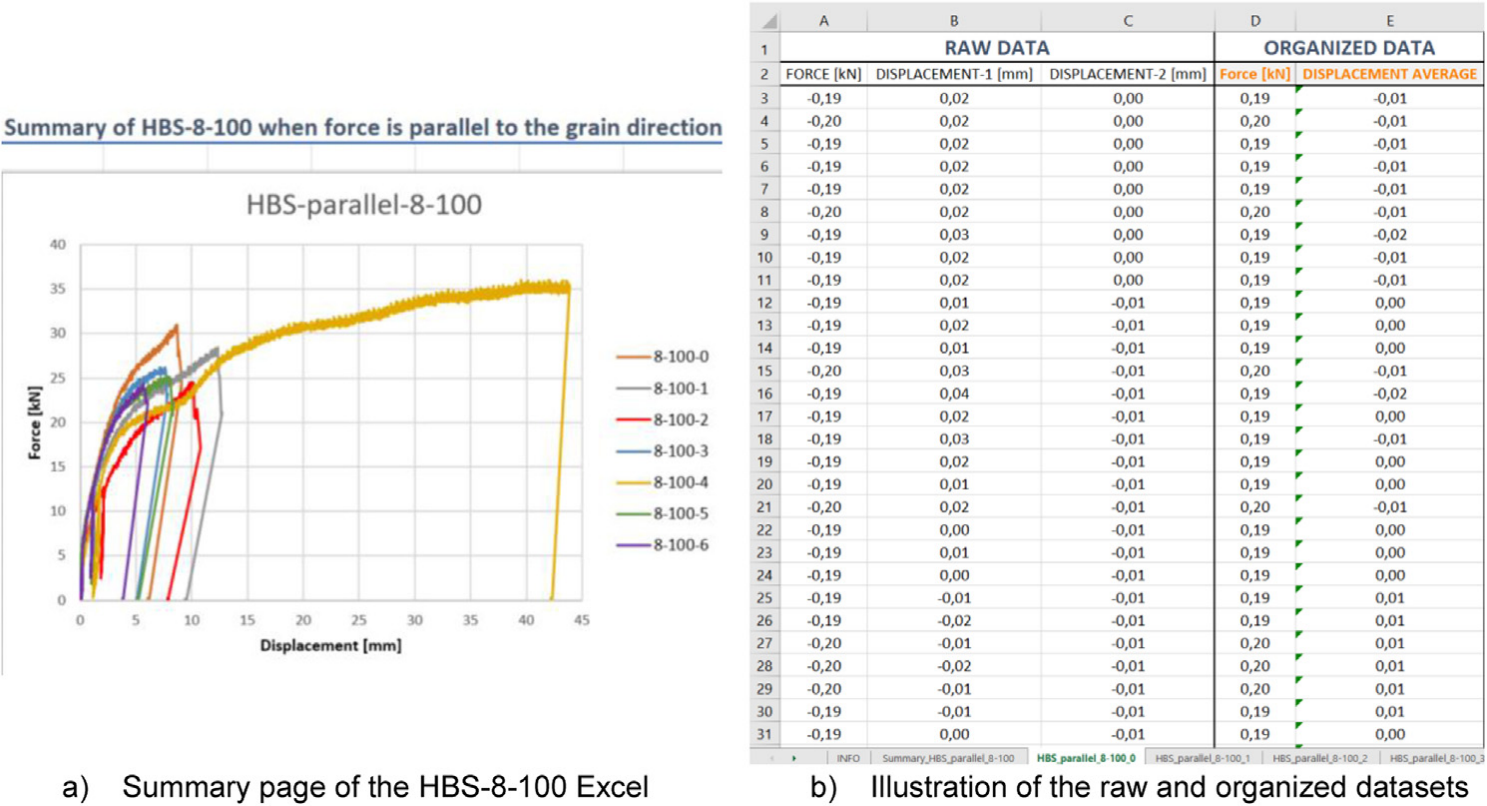
Illustration of the Push-out dataset. All datasets regarding one screw size and loading direction are gathered in the same Excel file.

The push-out datasets are segregated into two main folders by the screw type:

• HBS-type
• VGS-type

eight Excel files, each corresponding to a particular screw size and loading direction. As an example, the file “Push-out_parallel_HBS-8–100” presents data about the HBS-type screw, featuring an 8 mm diameter and 100 mm length, with a loading direction parallel to the topmost CLT layer. The layout of the Excel file, illustrated in Fig. 1, includes a summary of test results on the first sheet, followed by individual test data on subsequent sheets (Table 2).

**Table 2:** Summary of the Push-out tests.

Model name	Nominal diameter [mm]	Total length [mm]	Parallel	Perpendicular
HBS 8-100	8	100	x	x
HBS 10-100	10	100	x	x
VGS 9-100	9	100	x	x
VGS 9-120	9	120	x	x

### 3.3 Vibration

The vibration dataset consists of four data folders:

• Test-1
• Test-2
• Test-5
• Test-6

**Fig. 2:**
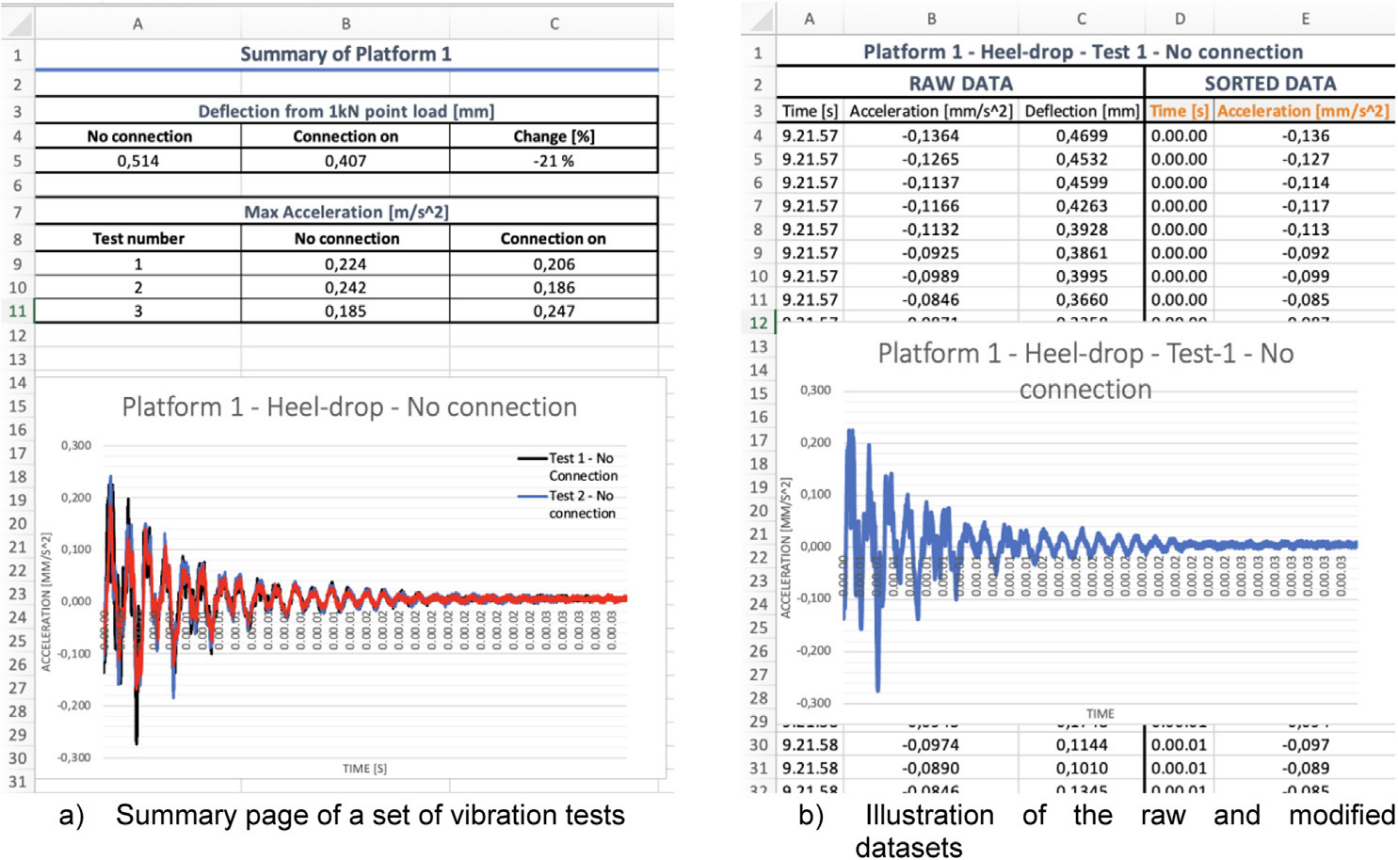
Illustration on how the vibration dataset is presented in Excel.

Each folder contains the distinct Excel file for deflection, Heel-drop and walking test. These tests were carried out before and after the installation of connector plates between the WQ-beam and the CLT slabs, see Table 3 for summary of conducted tests. For instance, the Excel file named “1-Platform_Vibration” encompasses summary, deflection, and vibration data from the initial platform tests, as shown in Fig. 2. Time stamps reflect real time according to the measuring instrument.

**Table 3:** Summary of the conducted Vibration tests.

	Test-1	Test-2	Test-3	Test-4	Test-5	Test-6
Span length of CLT [m]	4	4	4	2.6	2.6	2.6
Hell-drop	x	x	–	–	x	x
Walking	–	x	–	–	x	x
Deflection	x	x	–	–	x	x

## 4 Experimental Design, Materials and Methods

**Fig. 3:**
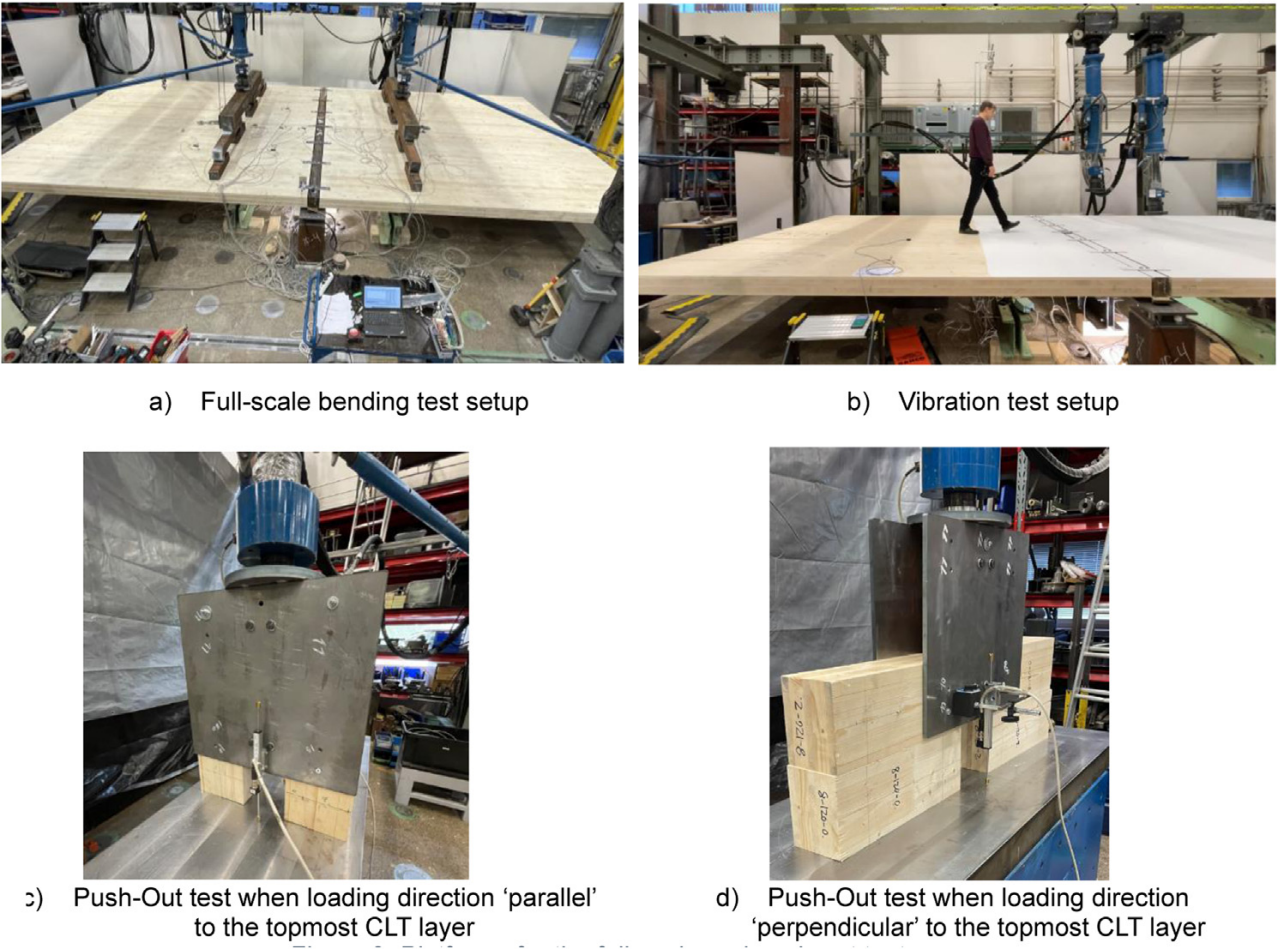
Platforms for the full-scale and push-out tests.

The experiments took place from 2021 to 2023 at the Laboratory of Structural Behaviour at Tampere University. The tests were categorised into three sections: (1) full-scale bending, (2) vibration, and (3) push-out. The platforms for the tests are depicted in Fig. 3. The subsequent sections provide crucial details on dimensions, material properties, instrumentation, fabrication, testing procedures, and test variables. Further specifics about the dimensions and measurements of the test specimens are available in Appendix A, showcasing all assembly drawings utilised for ordering and constructing the test specimens.

### 4.1 Dimensions of bending and vibration tests

**Fig. 4:**
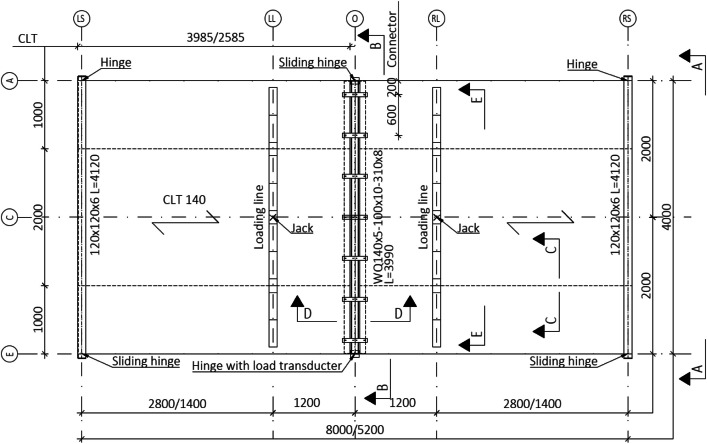
Blueprint of the full-scale bending test.

**Fig. 5:**
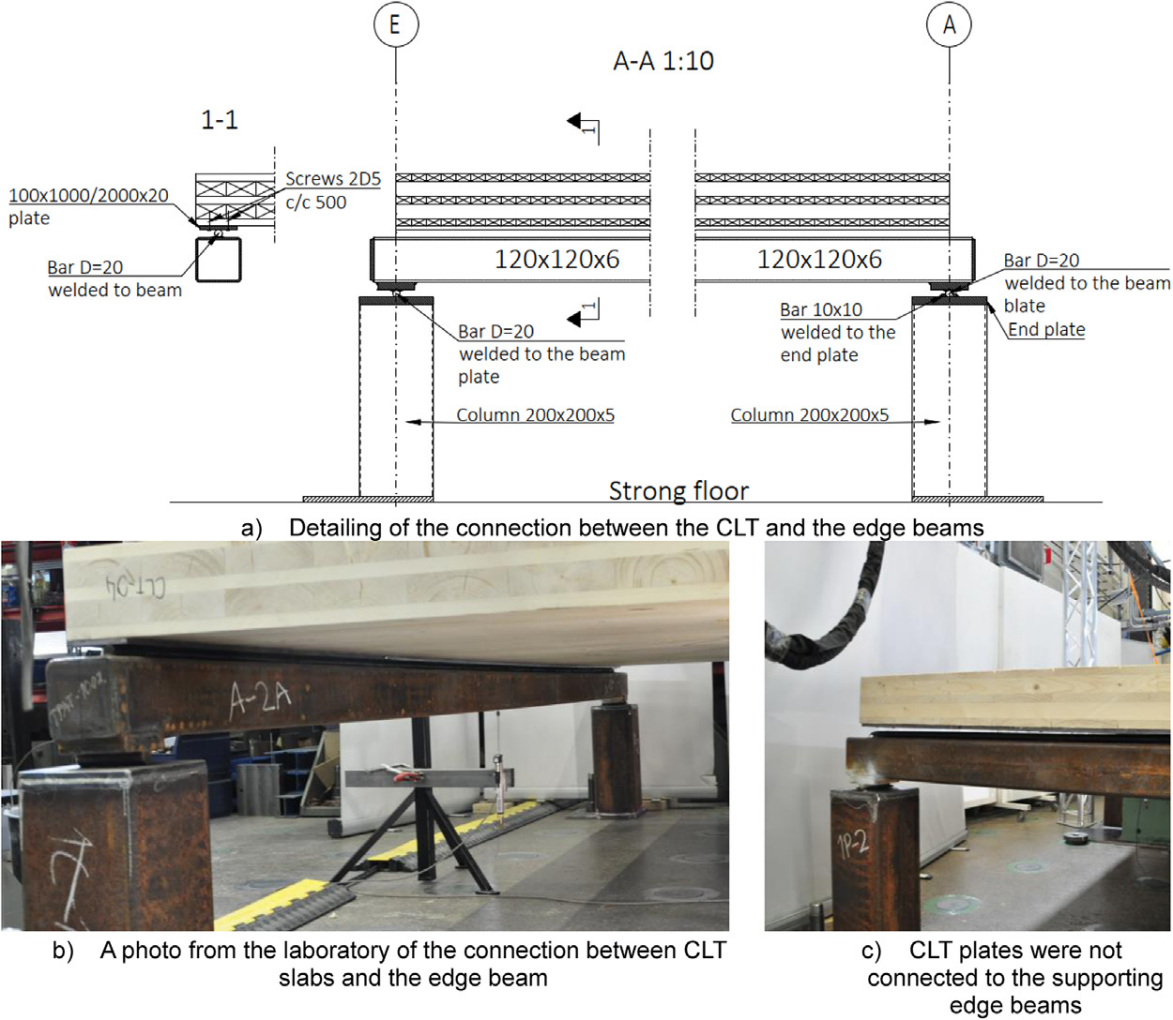
Illustration of the connections used with edge beams. The columns were rigidly connected to the strong floor of the laboratory to prevent them from moving during the experiment and between different tests.

**Fig. 6:**
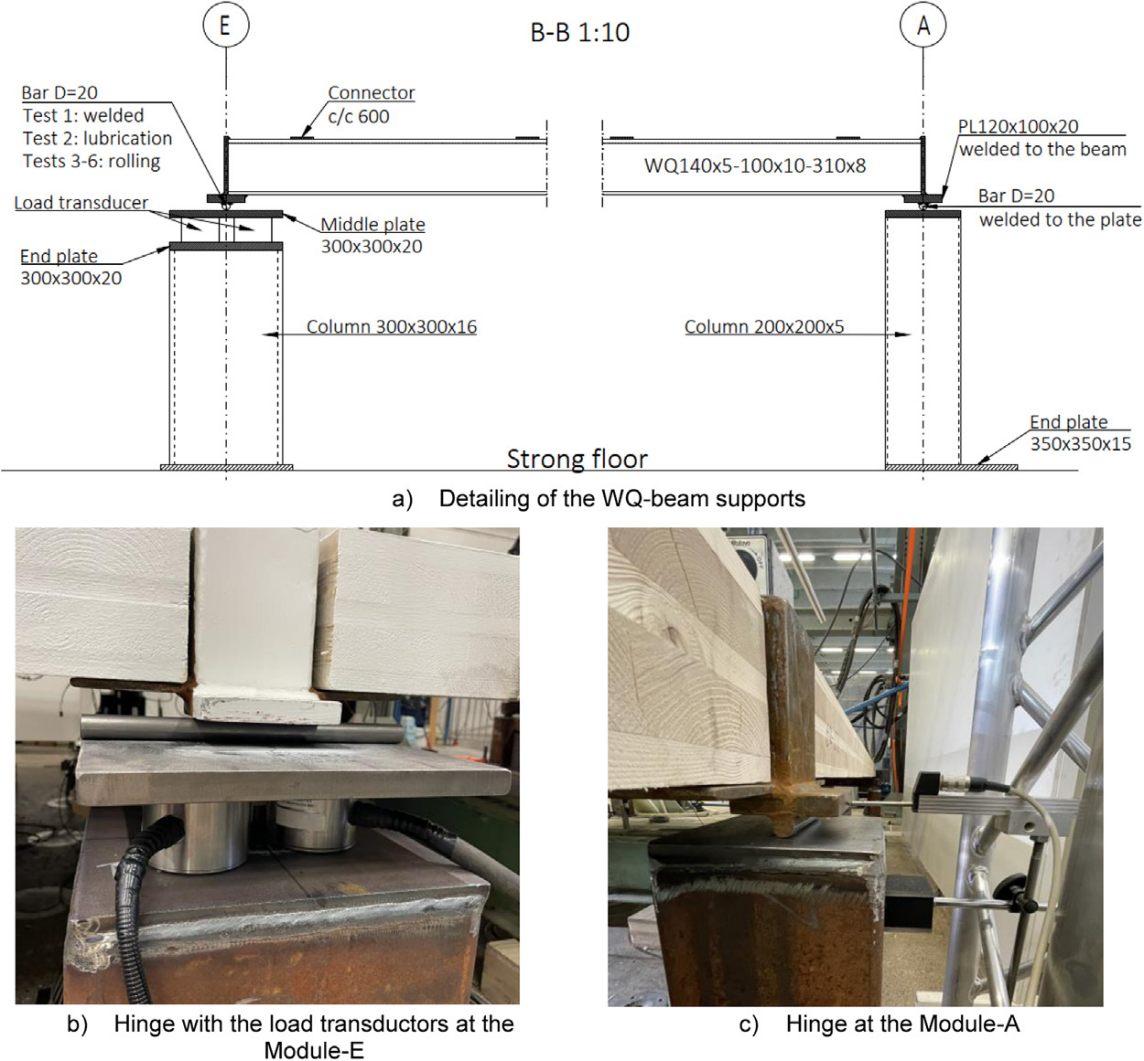
Illustration of connection types used with WQ-beam. Modifications were made to the Module-E hinge between different tests, these modifications are described in Fig. 23.

**Fig. 7:**
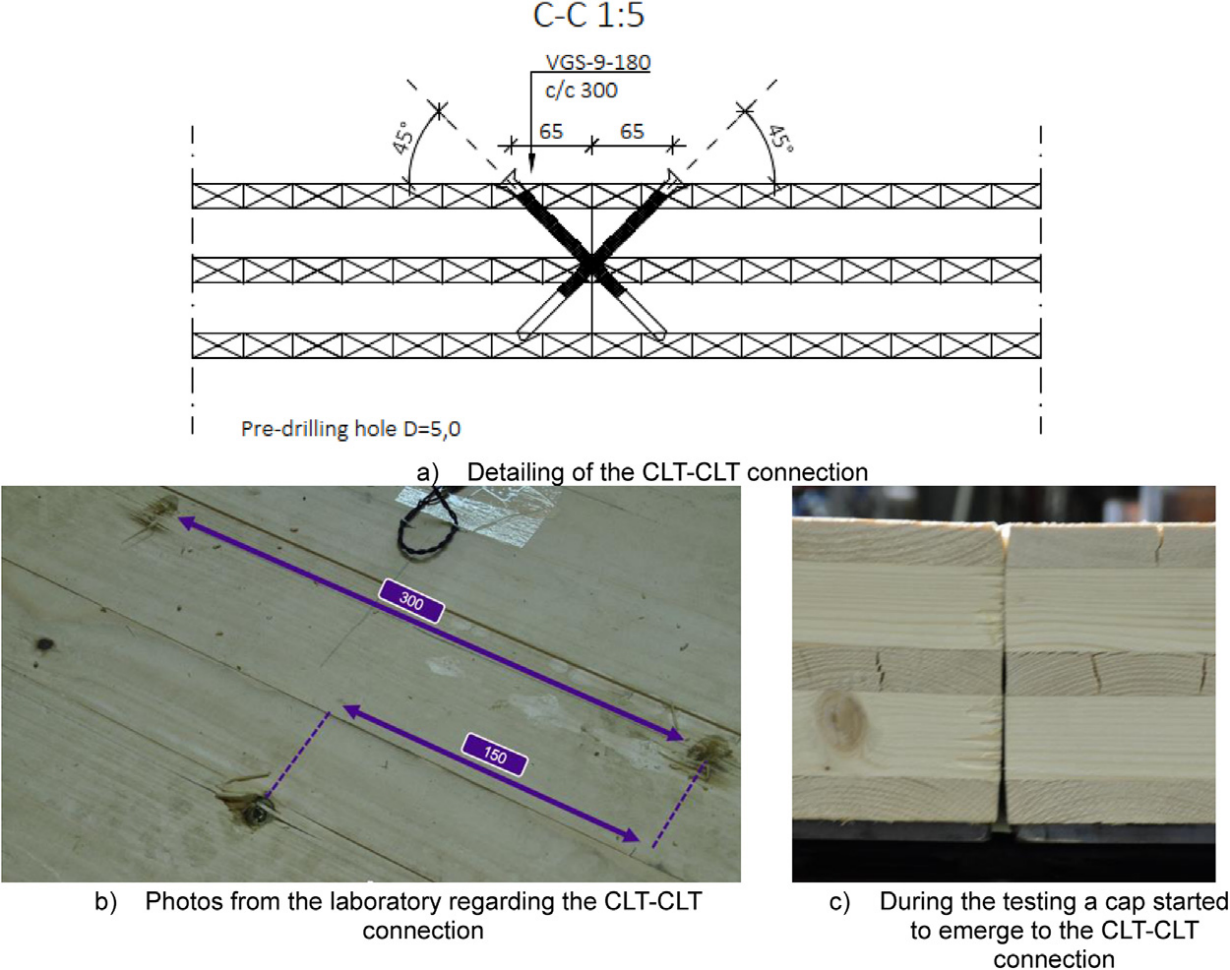
Illustration of CLT-CLT connection that were all done at the laboratory.

**Fig. 8:**
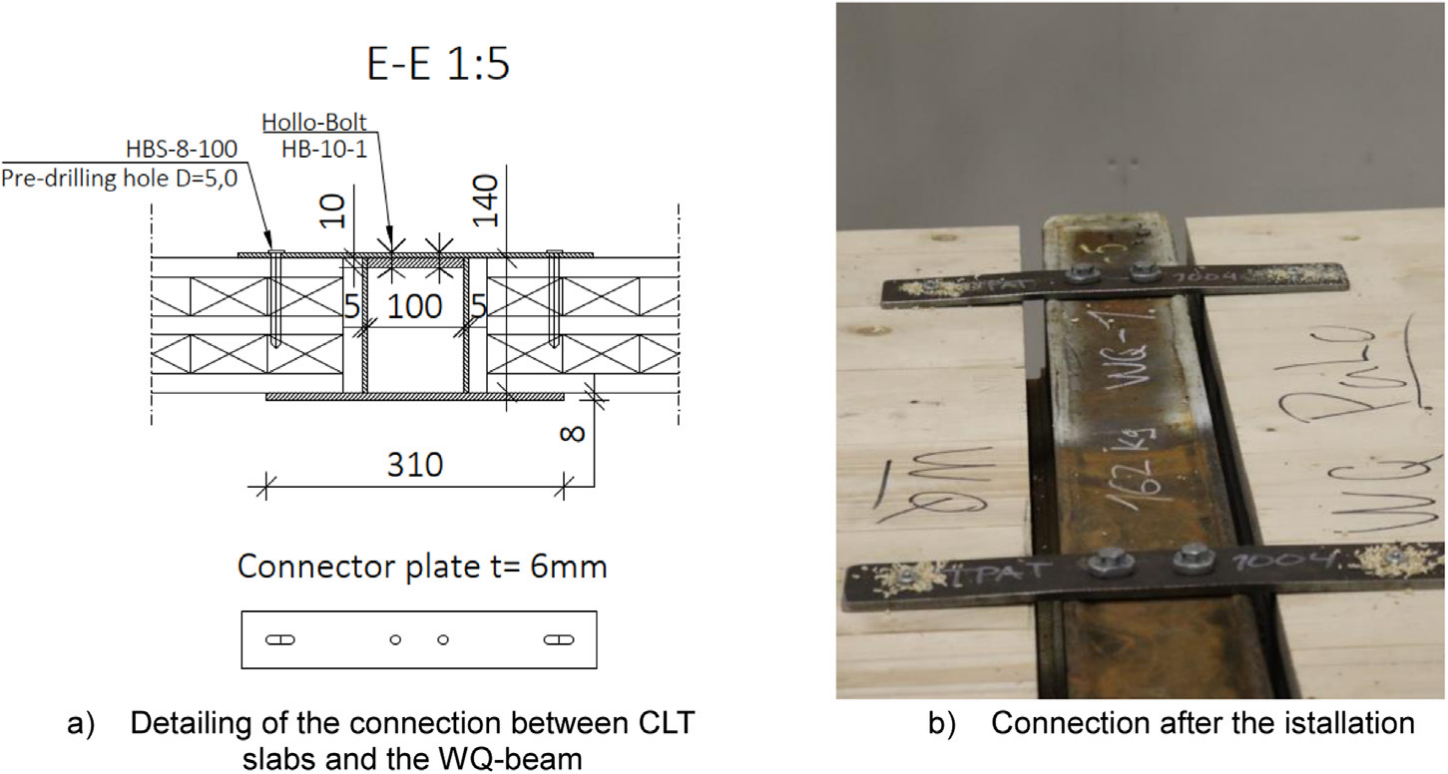
Illustration of connector plates that were used to initiate the composite action between the CLT and the WQ-beam.

**Fig. 9:**
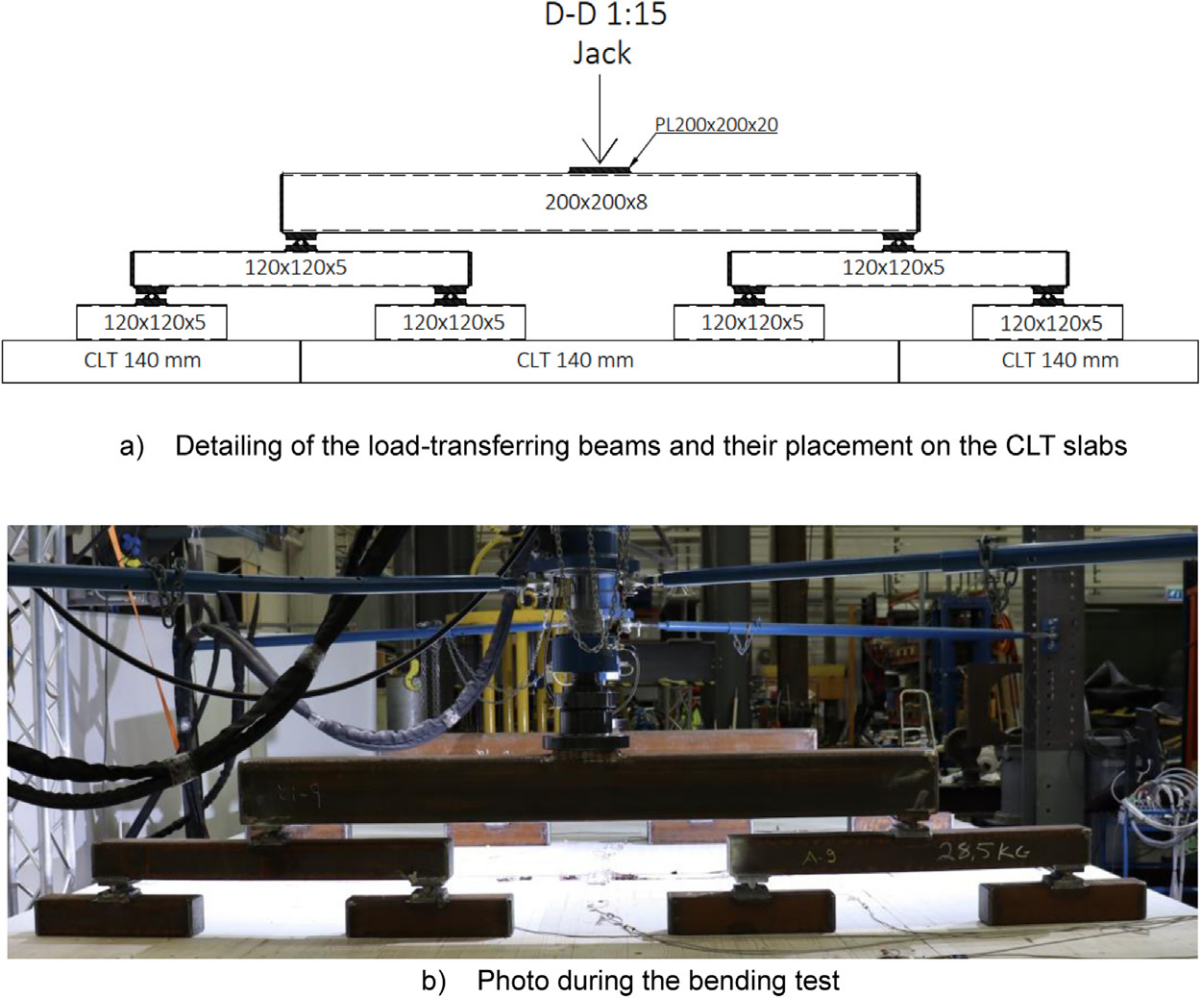
Illustration of load-transferring beams where all the connections were hinge joints.

A total of six platforms were constructed, with modifications made to the CLT span between tests, as depicted in Fig. 4. The CLT slabs were supported by cold-formed rectangular hollow section (RHS 120 × 120 × 5) beams on both sides, Fig. 5, and by a WQ-beam (140 × 5–100 × 10–310 × 8) in the middle, Fig. 6. The platform consisted of six CLT slabs (20-40-20-40-20), interconnected using slant screwing, as shown in Fig. 7. Noteworthy is that the CLT boards were not edge-glued and the topmost layer of CLT was perpendicular to the axis of the beam. Composite action was activated by using a steel connector shown in Fig. 8. The full-scale bending test simulated a continuous loading line with load-distributing beams, as shown in Fig. 9.

#### 4.1.1 Edge beams (Section A-A)

Fig. 5 illustrates the edge beam supports and connection between CLT slabs and the edge beam. Module-A joint is a hinge, and the Module-E is a sliding hinge joint. The hinge joint was made by welding a 120 × 100 × 20 base plate to the bottom flange of the beam and then a D20 round bar was welded to the base plate. The round bar was installed between two 10 × 10 solid steel bars which were welded to the end plate of the supporting column so that the round D20 bar could rotate but it cannot slide. The sliding hinge was manufactured similarly but without the 10 × 10 bars. Section 1-1 in Fig. 5 illustrates the connection between the edge beam and CLT slabs. Here the joint allows the rotation and sliding of CLT slabs. The joint is made by connecting a 100 × 1000/2000 × 20 plate to the CLT with wood screws and then placed on top of the D20 bar that is welded to the steel beam.

#### 4.1.2 WQ-beam (Section B-B)

Fig. 6 illustrates the supports of the WQ-beam where Module-E and Module-A joints are sliding hinges. Positioned under the left side of the WQ-beam are load transducers that measure the support reaction. Three modifications to the Module-E hinge were introduced in the first three tests, detailed in Fig. 23.

#### 4.1.3 CLT to CLT connection (Section C-C)

Fig. 7 illustrates the connection between adjacent CLT slabs where the angle of screws is 45° and the edge spacing fulfils the requirements set by the manufacturer. Pre-drilling was done at the laboratory.

#### 4.1.4 Shear connector plates (Section D-D)

Fig. 8 illustrates the connection between the WQ-beam and CLT slabs, where the composite effect is initiated by using the steel connectors. The connection to the CLT was established using wood screws (HBS-8-100) from Rothoblass, designed to be used with steel plates to ensure optimal connection. The plates were affixed to the WQ-beam using Hollo-bolts (HB-10-1) from Lindapter that could be easily installed and removed in the laboratory with a bolt driver. Both connectors were initially installed using a bolt or screwdriver and then hand-tightened, with a torque of 45 Nm for Hollo-bolts and 20Nm for wood screws. Additional technical details from manufacturers can be accessed in Appendix B. Itʼs essential to avoid over-tightening wood screws to prevent wood damage, which could compromise the connection's ability to bear vertical loads.

#### 4.1.5 Loading line (Section E-E)

Fig. 9 depicts the load-transfer beams positioned symmetrically on both sides of the WQ-beam and connected by a hinge joint. These beams' configurations aimed to mimic a continuous line load on the CLT slab. Consistently, all tests were conducted using these same load-transfer beams.

### 4.2 Dimensions of push-out tests

**Fig. 10:**
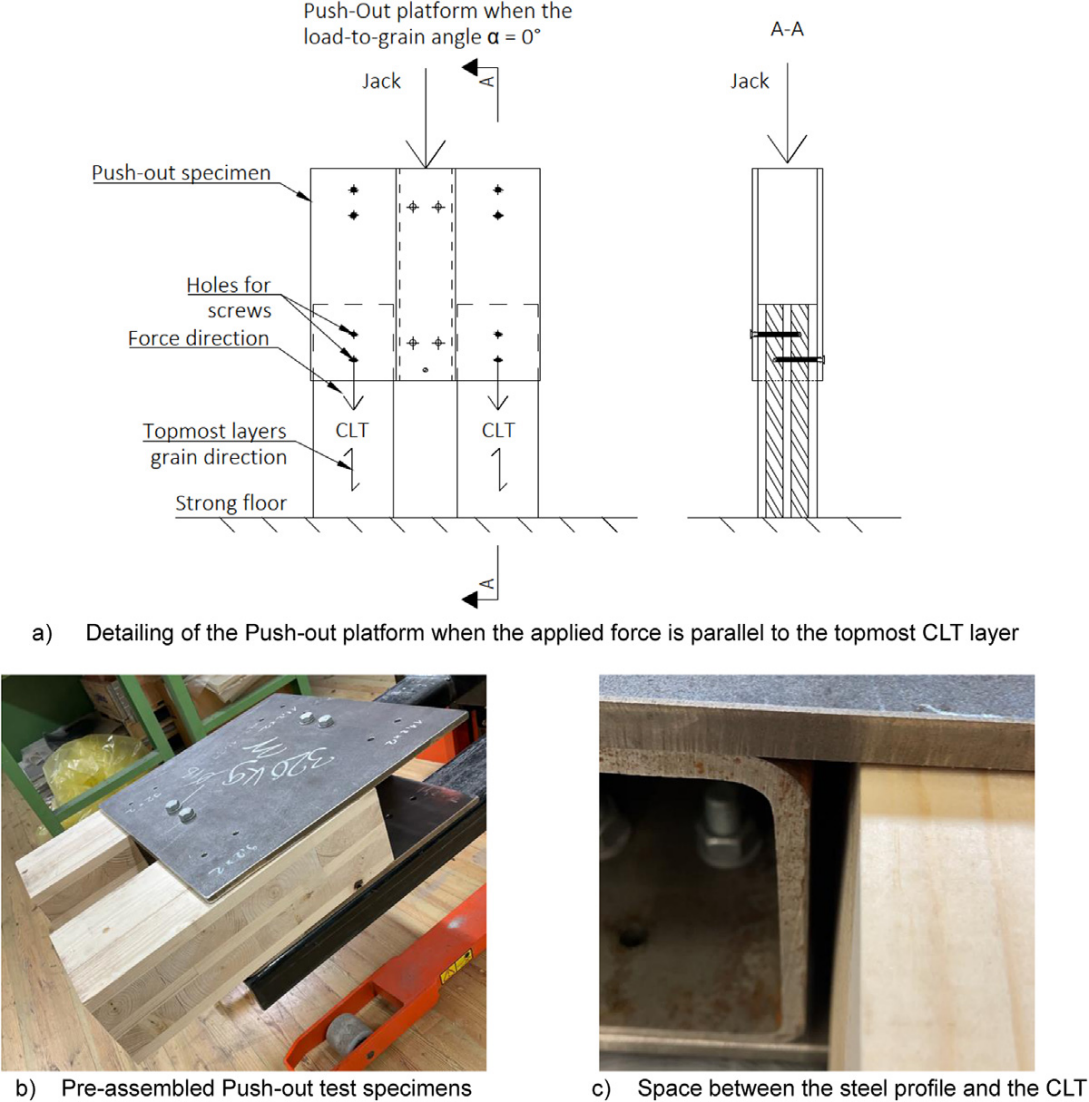
Assembled Push-out test specimens and CLT dimensions check before testing.

The push-out test specimens and setup were modelled based on prior studies [1,2] and [3]. Consistent with the connections in the full-scale tests, the CLT specimens were situated between two steel plates, designed to withstand forces without the necessity for replacement. To minimise friction between the CLT and the steel profile used for load introduction, a gap was deliberately left, as shown in Fig. 10. The surface of the CLT was sanded to reduce friction if any roughness was detected.

#### 4.2.1 Push-out specimen

**Fig. 11:**
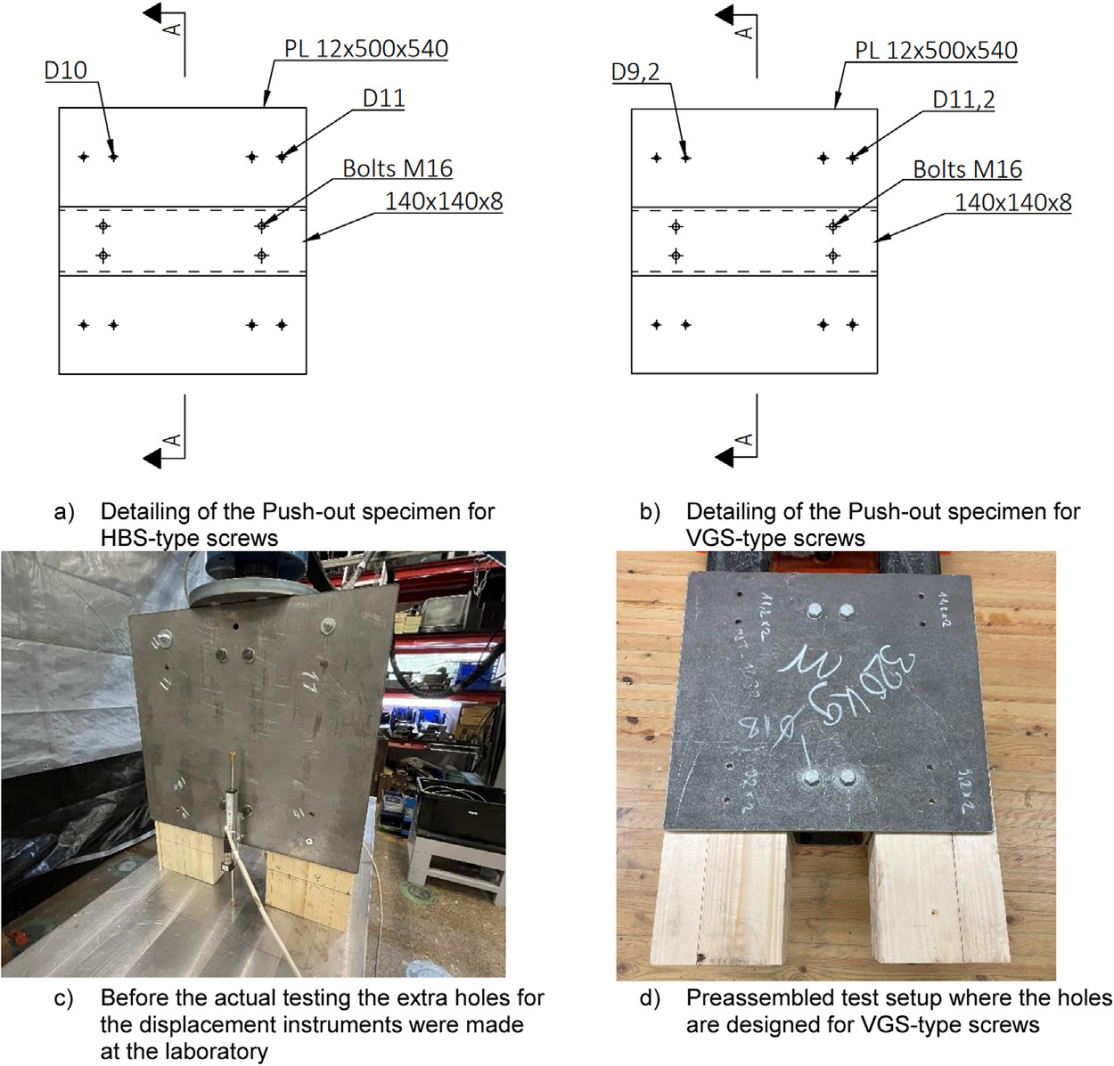
Differences between the Push-out test specimen.

The steel component comprised two 12 × 500 × 540 steel plates connected to an RHS 140 × 140 × 8 profile using four M16 bolts, as depicted in Fig. 11. These steel plates were equipped with pre-drilled holes tailored for timber screws, fulfilling the edge distance criteria outlined by the manufacturer, see Appendix B. Given the variation in screw diameters and types, two sets of steel plates were manufactured. Total weight of the push-out specimen was 0,7 kN which includes the additional weight of the steel plates that was used to transfer the loads from the jack, see Fig. 11. The distinguishing factor between the plates was primarily the diameter of the holes for the timber screws, demonstrated in Fig. 11.

#### 4.2.2 CLT specimens

**Fig. 12:**
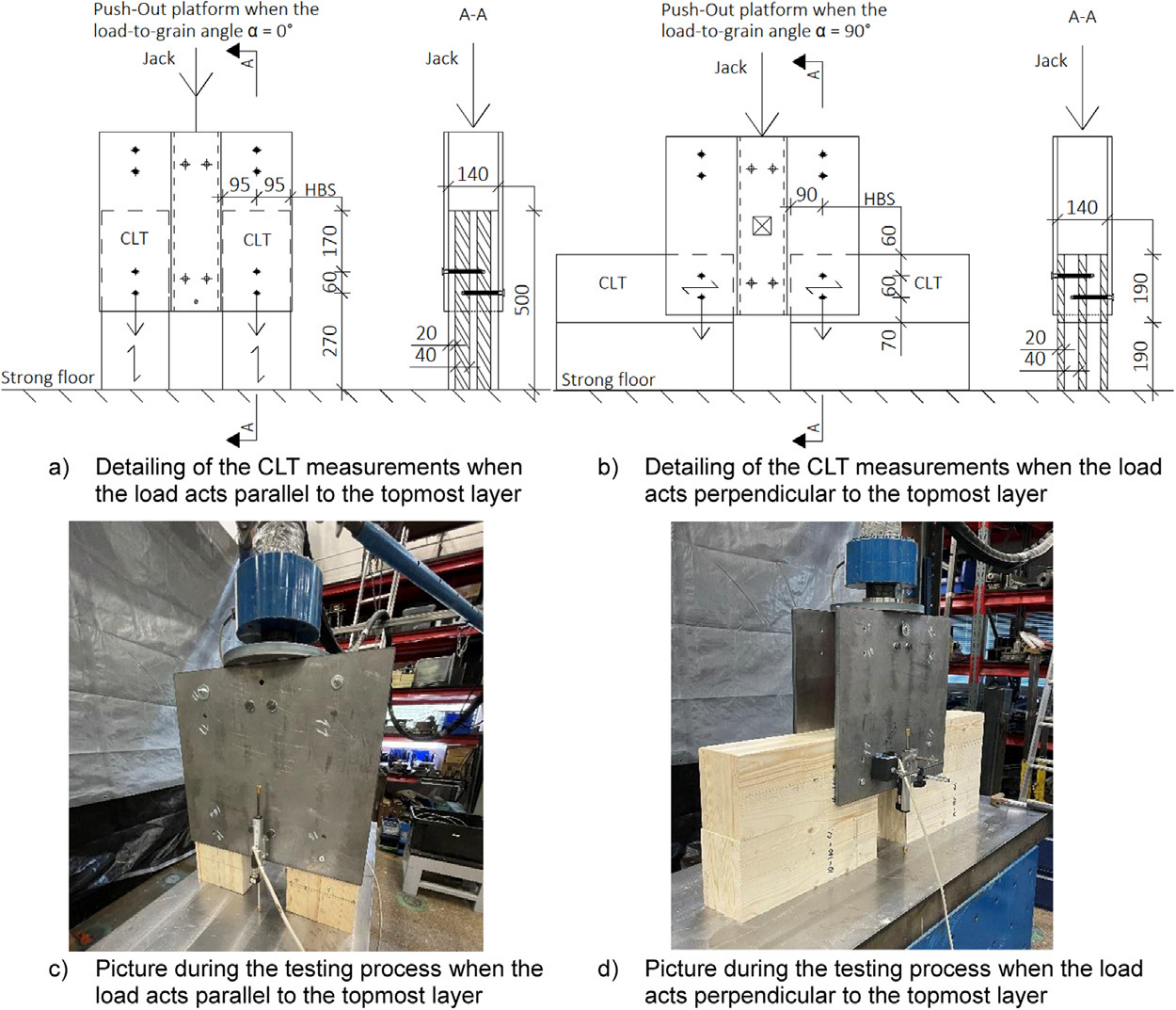
Dimensions of the CLT specimens and how they HBS-type crews were located.

All tests were executed using the identical CLT profile, 20-40-20-40-20, consistent with that used in full-scale tests. The manufacturer (Arcwood) pre-cut the CLT specimens directly to ensure dimensional precision between different test pieces. Table 4 provides details on the screw type, CLT specimen diameters, and the pre-drill hole diameter. The hole dimensions for pre-drilling and the recommended spacing distances were determined based on the manufacturer's specifications, as outlined in the manufacturer's guides, see Appendix B (Fig. 12).

**Table 4:** Screw type, size of the used CLT specimens and pre-drilling hole diameters.

Screw type	CLT size [mm]	Pre-drilled hole [mm]
VGS 9-100	140 × 200 × 500	6
VGS 11-120	140 × 200 × 500	7
HBS 8-100	140 × 190 × 500	5
HBS 10-100	140 × 190 × 500	5

### 4.3 Material properties

#### 4.3.1 CLT

**Fig. 13:**
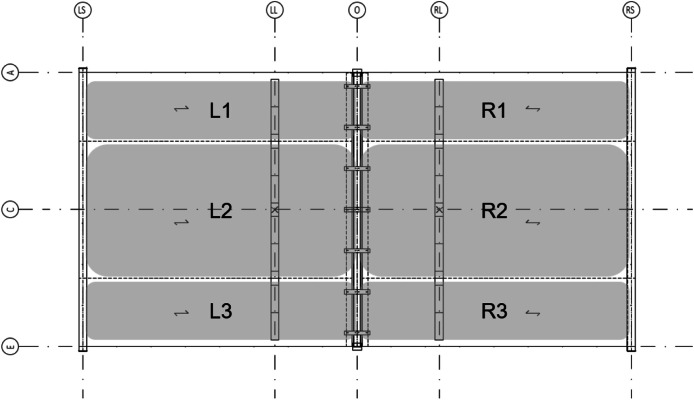
Position illustration of the weights that are given in Table 6.

The CLT thickness was 140 mm consisting of 20-40-20-40-20 lamellas were all the lamellas were made from C24 strength graded timber. Material properties given by the manufacturer are listed in Table 5 and all the weights recorded before testing are presented in Table 6. Fig. 13 illustrates the weight of the recorded CLT panels.

**Table 5:** Material properties of the CLT are based on the manufacturer's data and laboratory measurements.

Strength class for layers (EN 338, EN 14,080, EN 16,351)	C24
Rolling shear strength, *f_r,k_* [MPa]	1,2
Rolling shear strength, *G*_90_ [MPa]	65
Moisture [%]*	10,6
Density [kg/m^3^]*	427,3

⁎ measured at the laboratory from tests 4, 5 and 6.

**Table 6:** Weights of the CLT panels.

CLT panel	Test-1	Test-2	Test-3	Test-4	Test-5	Test-6
L1 [kg]	262	274	268*	173*	175	172
L2 [kg]	546	542	544*	347*	349	345
L3 [kg]	260	274	267*	173*	175	171
R1 [kg]	262	274	268*	172*	170	174
R2 [kg]	538	536	537*	342	347	348
R3 [kg]	264	266	265*	171	174	171

⁎ calculated based on the tests 1, 2, 5 and 6.

#### 4.3.2 Steel parts

All the steel components were supplied by Nordec and fabricated using steel grade S355K2+*N* for the steel plates, and S355J2H for the beam profiles. The material properties provided by the manufacturer are detailed in Table 7. These values represent the standard information, quality control metrics, given by the manufacturer; thus, the number of test samples varied between 1 and 8, from which the average values were calculated. The maximum deviation from the average yield strength was 1,6 %, while the deviation from the average tensile strength was 0,3 %. The weights of the WQ beams and the load-transferring beams are shown in Table 8, Table 9, respectively.

**Table 7:** Material properties of steel components given by the manufacturer.

Part [%]	Steel grade	Yield strength [MPa]	Deviation [%]	Tensile strength [MPa]	Deviation [%]	Standard
WQ beam: bottom flange	S355K2+N	449	1,6*	565	0,1*	EN 10025-2:2004
WQ beam: web	S355K2+N	406	0,3⁎⁎	552	0,3⁎⁎	EN 10025-2:2004
WQ beam: top flange	S355K2+N	432	–	561	–	EN 10025-2:2004
Connector plates	S355K2+N	396	0⁎⁎⁎	535	0⁎⁎⁎	EN 10025-2:2004
Edge beams	S355J2H CF	538	–	602	–	EN 10219-1:2006

⁎ based on 3 samples.

⁎⁎ based on 8 samples.

⁎⁎⁎ based on 4 samples.

**Table 8:** Laboratory-measured weights of the WQ beams.

Part	Test-1	Test-2	Test-3	Test-4	Test-5	Test-6
WQ beam [kg]	162	162	162^a^	162	161	162

a calculated based on the tests 1, 2, 4, 5 and 6.

**Table 9:** Laboratory-measured weights of the load-transferring beams.

Profile	Weight [kg]
RHS 200 × 200 × 8	113,5
RHS 120 × 120 × 5, long	28,5
RHS 120 × 120 × 5, short	13,0

#### 4.3.3 Fasteners

**Fig. 14:**
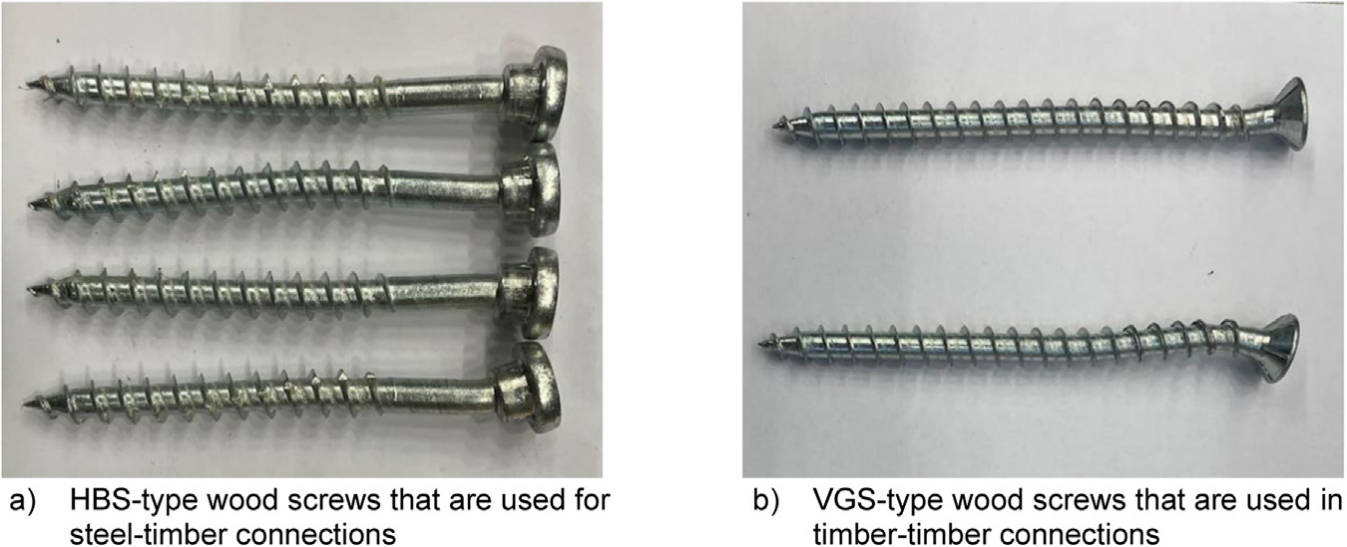
Illustration of the head shape difference between HBS and VGS screws.

All the wood screws were supplied by Rothoblaas and the Hollo-bolts by Pretec. Two types of screws were used during the full-scale and push-out tests: HBS and VGS screws. The HBS screws are designed to be used with steel plates, while the VGS screws are used without steel plates, as shown in Fig. 14. Table 10 provides details on the types of wood screws, their use cases, diameters, lengths, maximum forces, and tensile strengths. The values for maximum force and tensile strength were determined according to the EN-ISO 6892-1:2019 (A) standard. The tests were conducted by METLAB. The number of samples in all screw sizes was five, and the maximum deviation from the average maximum force was 3,7 %, while the deviation from the tensile strength was below 3,8 %. More detailed material properties and dimensions of the wood screws and Hollo-bolts are presented in Annex B.

**Table 10:** Summary of the different wood screws, their use case and material properties.

Use case	Model name	Tip diameter [mm]	Total length [mm]	Max force [kN]	Deviation [%]	Tensile strength [MPa]	Deviation [%]
Push-out	VGS 9-100	5,9	100	37,5	0,7	1370,6	0,7
CLT-CLT	VGS 9-180	5,9	180	37,5	0,7	1370,6	0,7
Connectors / Push-out	HBS 8-100	5,4	100	29,2	3,7	1273,8	3,8
Push-out	HBS 10-100	6,4	100	39,5	1,1	1229,6	1,0

### 4.4 Instrumentations and fabrication

**Fig. 15:**
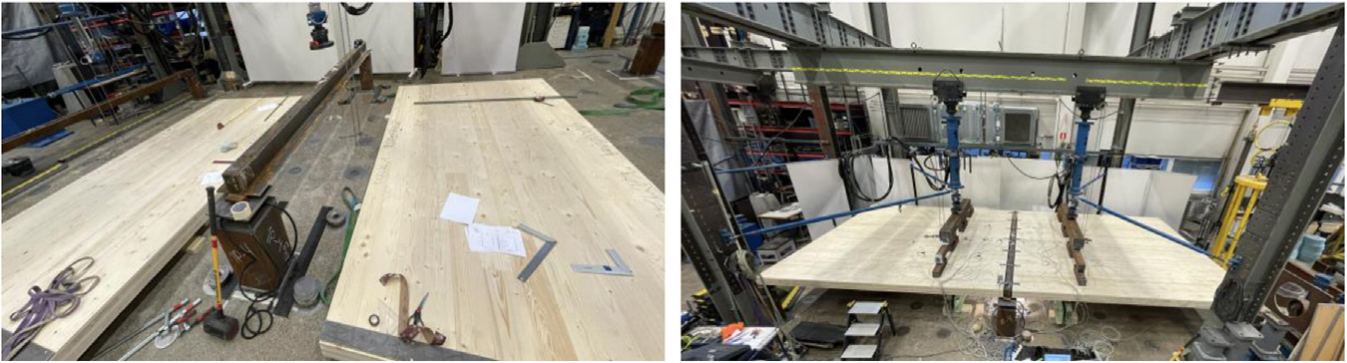
Assembling the first Full-scale test platform.

The tested platforms were constructed within the laboratory's loading frame, with columns anchored to the strong floor to minimise any lateral movements during testing. This arrangement also facilitated the reassembly of subsequent tests, as only the CLT, WQ beams, connector plates, and connectors (wood screws and Hollo-bolts) needed to be replaced. Fig. 15 illustrates the force grid, strong floor, and the overall assembly of the platform. It should be noted that all necessary steps, from assembly and instrumentation to measurement, were carried out by the same laboratory staff members and the responsible writer.

#### 4.4.1 Full-scale bending

During the bending test, the recorded variables were:

• Forces from jacks 1 (left-hand side) and 2 (right-hand side), see Fig. 15
• Contact force at one end of the WQ-beam, measured using load transducers
• Deflections of the platform and lateral displacements at the WQ-beam supports
• Strains on the top and bottom parts of the WQ-beam and CLT slabs, measured using strain gauges

**Fig. 16:**
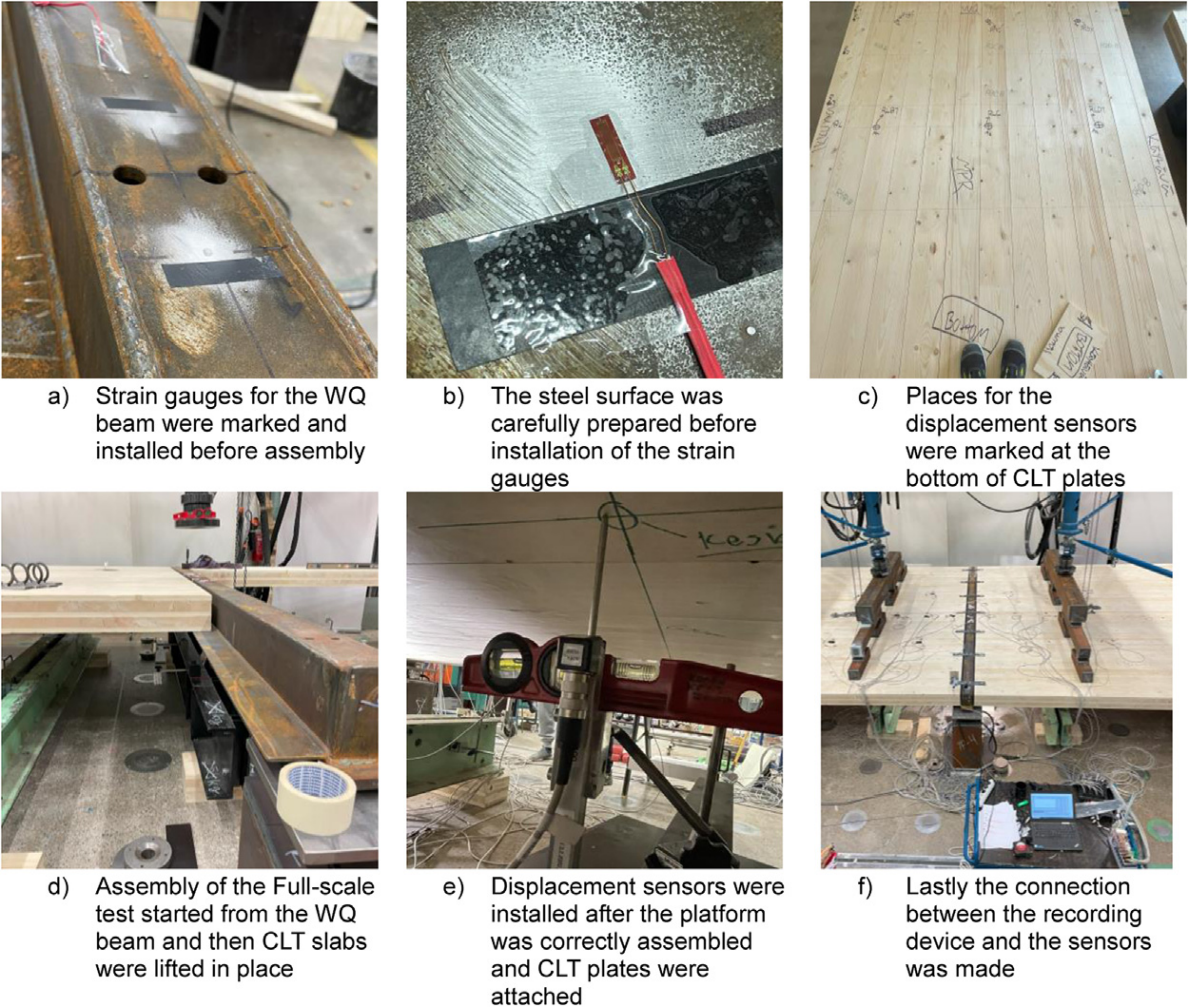
Installation process of the measuring instruments for the Full-scale tests.

All variables were recorded every second using the same computer to ensure that the data points were time-linked. The same recording setup was used throughout all tests. Fig. 16 shows photographs taken during the installation phase of the measuring instruments. The process followed the order: placements for the strain gauges and displacement sensors were first marked and then installed. After this, the strain gauges were installed, and parts were assembled. Finally, the rest of the instrumentation was carried out, such as connecting strain gauges to the computer and adjusting the displacement sensors to their places.

**Fig. 17:**
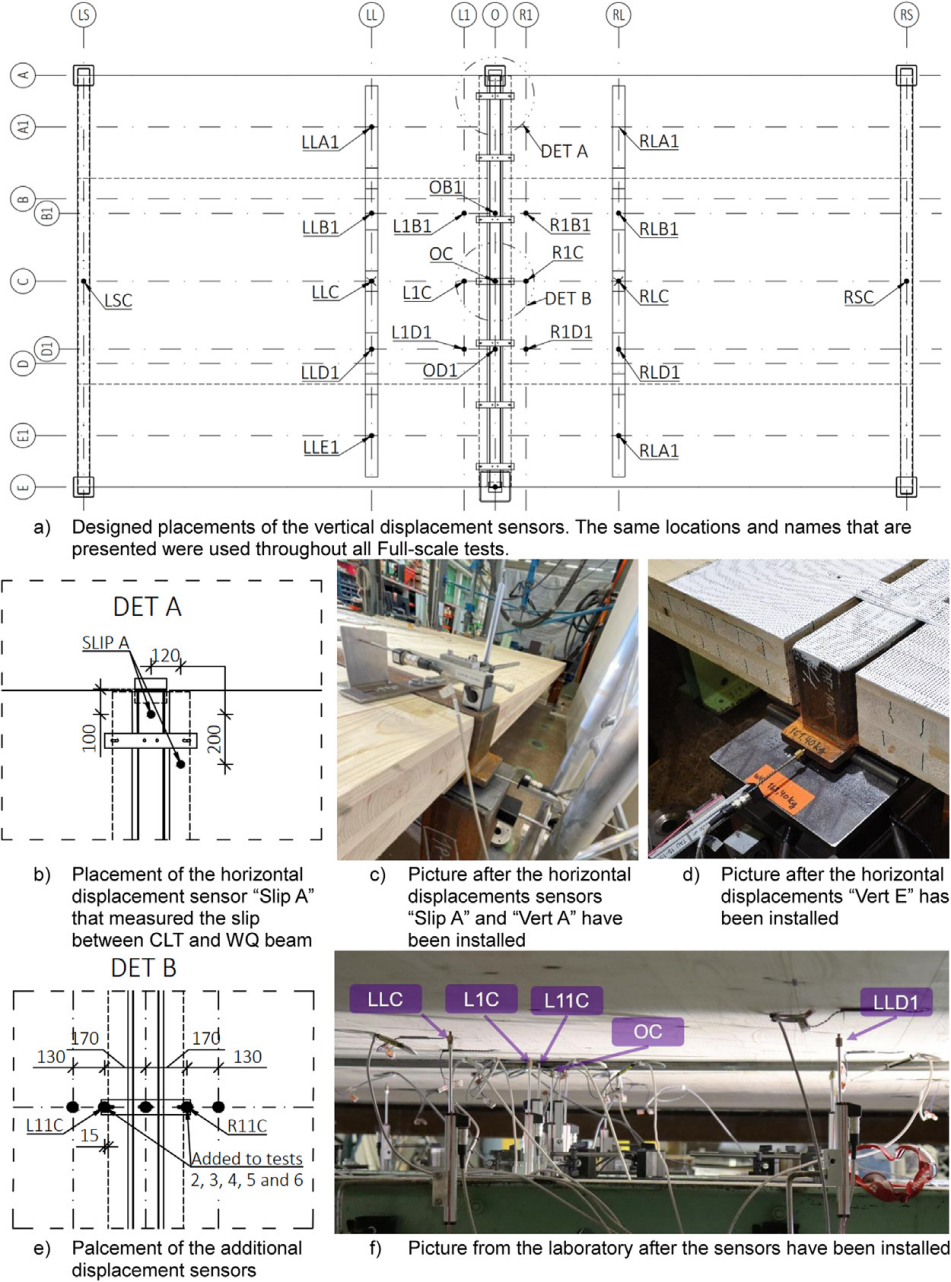
Designed placements of the displacement sensors and pictures of how they were arranged at the laboratory.

**Fig. 18:**
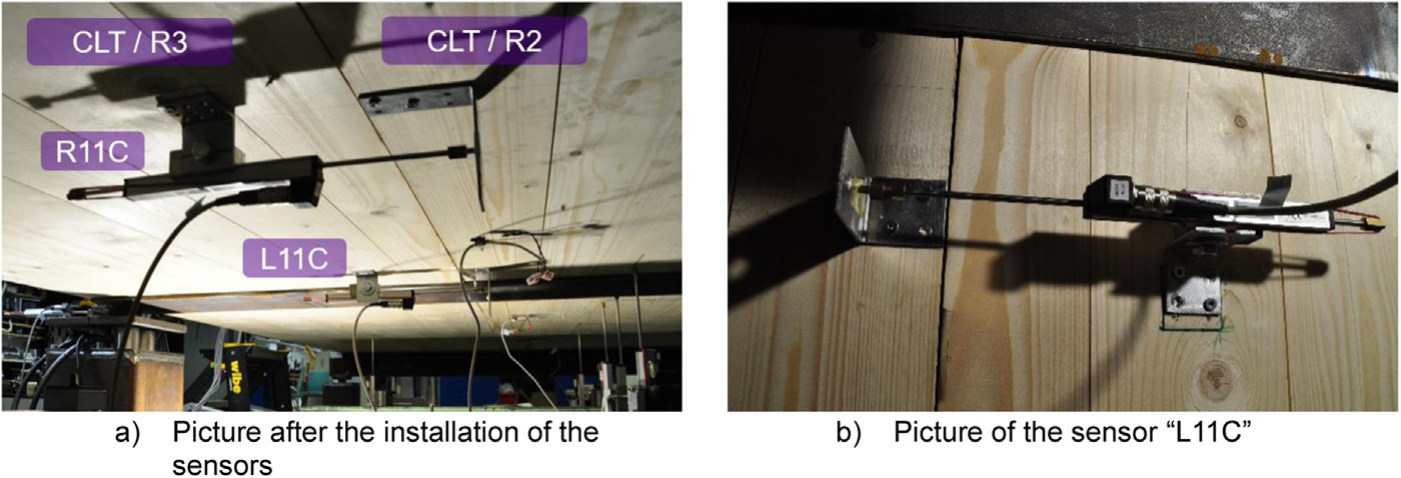
Movement of the CLT seam was captured in the last Full-scale test using sensors “L11C” and “R11C”.

Fig. 17 shows the positions of the vertical and horizontal displacement sensors utilised for the full-scale tests. All vertical sensors were installed to measure the displacement from the bottom of the steel beams and CLT slabs, as can be seen from Figs. 16 to 17. After the first full-scale test, two additional vertical sensors, “L11C” and “R11C”, were added to better capture the deflection difference between the CLT plate and the WQ beam at the midspan, as shown in Fig. 17. These sensors, “L11C” and “R11C”, were used in tests 2, 3, 4, and 5. For the final test, the sensors were moved to capture the movement of the CLT seam between the R2 and R3 slabs, as shown in Fig. 18. Sensor “L11C” was located along Module-R1 and sensor “R11C” was located on Module-RL. Additionally, two horizontal sensors, “VERT A” and “VERT E”, were added after the first test to capture the movement of the WQ beam at the support area, as indicated in Fig. 17.

Table 11 summarizes the modifications made to the displacement sensors between tests.

**Table 11:** Summary of the modifications made to the displacement sensors between different Full-scale tests.

Sensor name	Type	Purpose	Position	Usage in tests
L11C & R11C	Vertical	Deflection between CLT plate and WQ beam at midspan	Midspan of the beam	Tests 2, 3, 4, 5
L11C & R11C	Horizontal	Movement of the CLT seam between R2 and R3 slabs	Module-R1 & Module-RL	Test 6
VERT A & VERT E	Horizontal	Movement of WQ beam at support area	Module-A & Module-E	Tests 2, 3, 4, 5

**Fig. 19:**
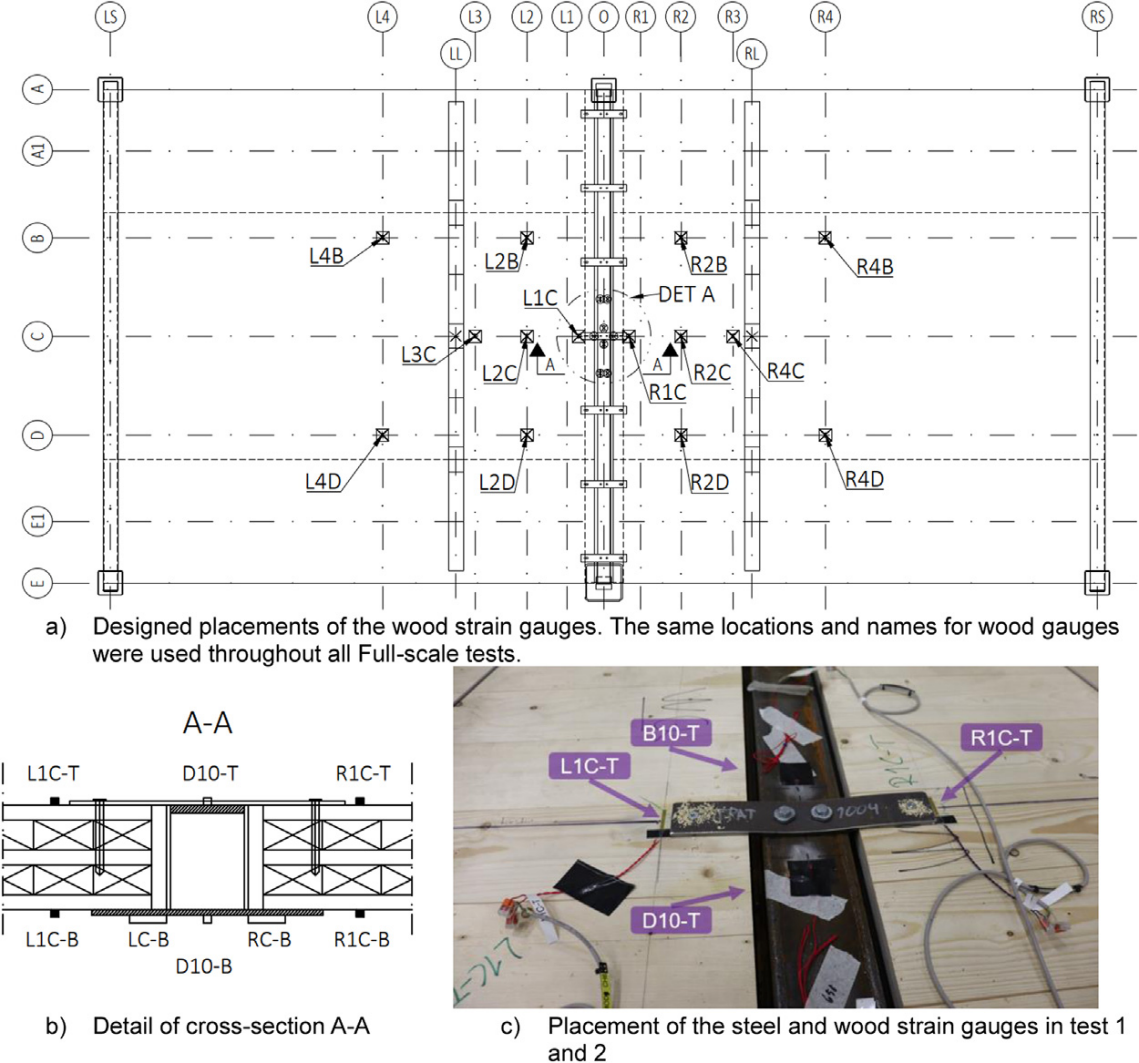
Designed placements of the strain gauges and pictures of how they were arranged at the laboratory.

**Fig. 20:**
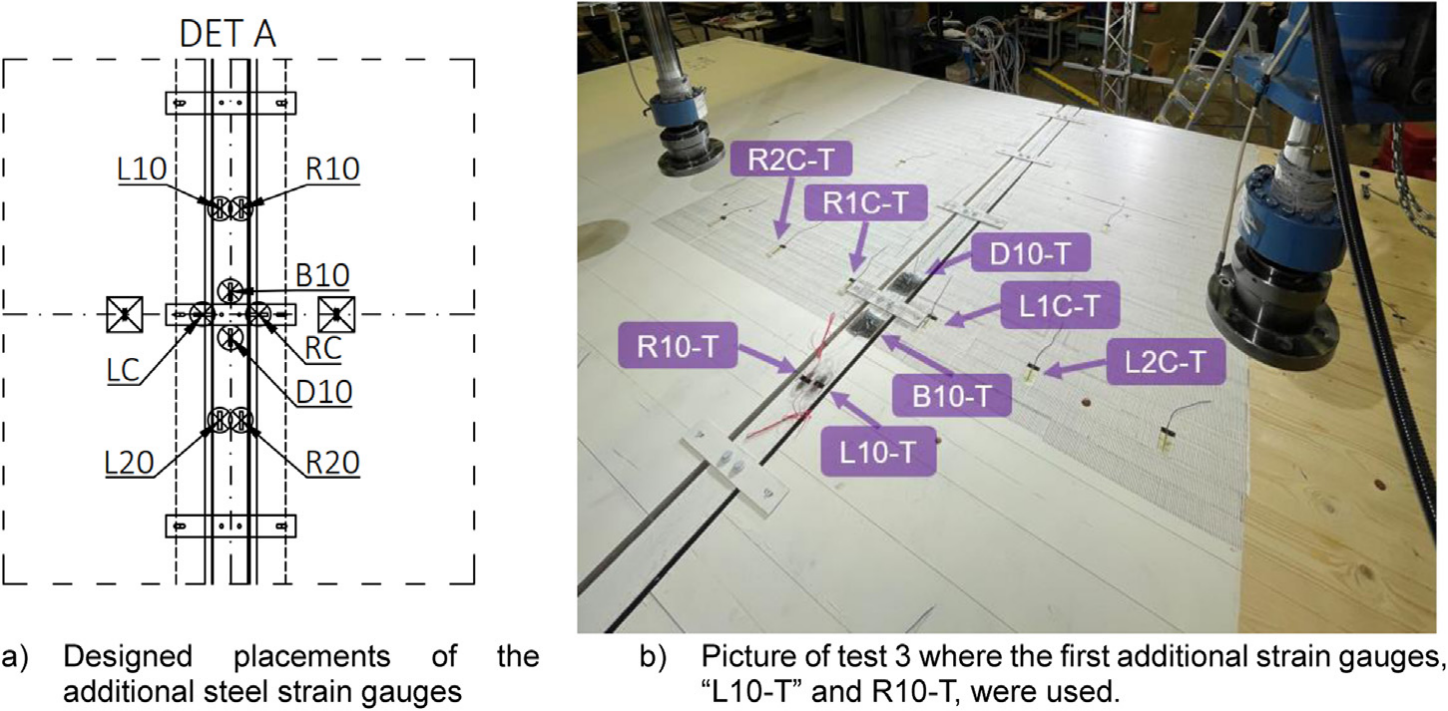
Additional steel strain gauges were added to the tests 3, 4, 5 and 6.

Fig. 19 illustrates the positions of the wood and steel strain gauges as they were designed and then installed in the laboratory. For the CLT, strain gauges were installed on both the top and bottom surfaces at the same locations. After the first test, additional steel gauges, “L10-T” and “R10-T”, were installed on the top surface of the WQ beam and after the fourth test “L20-T” and “R20-T” were also installed, as shown in Fig. 20 and Table 12.

**Table 12:** Summary of the additional steel strain gauges between different Full-scale tests.

Sensor name	Type	Purpose	Position	Usage in tests
L10-T & R10-T	Strain gauges	Steel	Top surface of WQ beam	Tests 2, 3, 4, 5, 6
L20-T & R20-T	Strain gauges	Steel	Top surface of WQ beam	Tests 5, 6

#### 4.4.2 Vibration

**Fig. 21:**
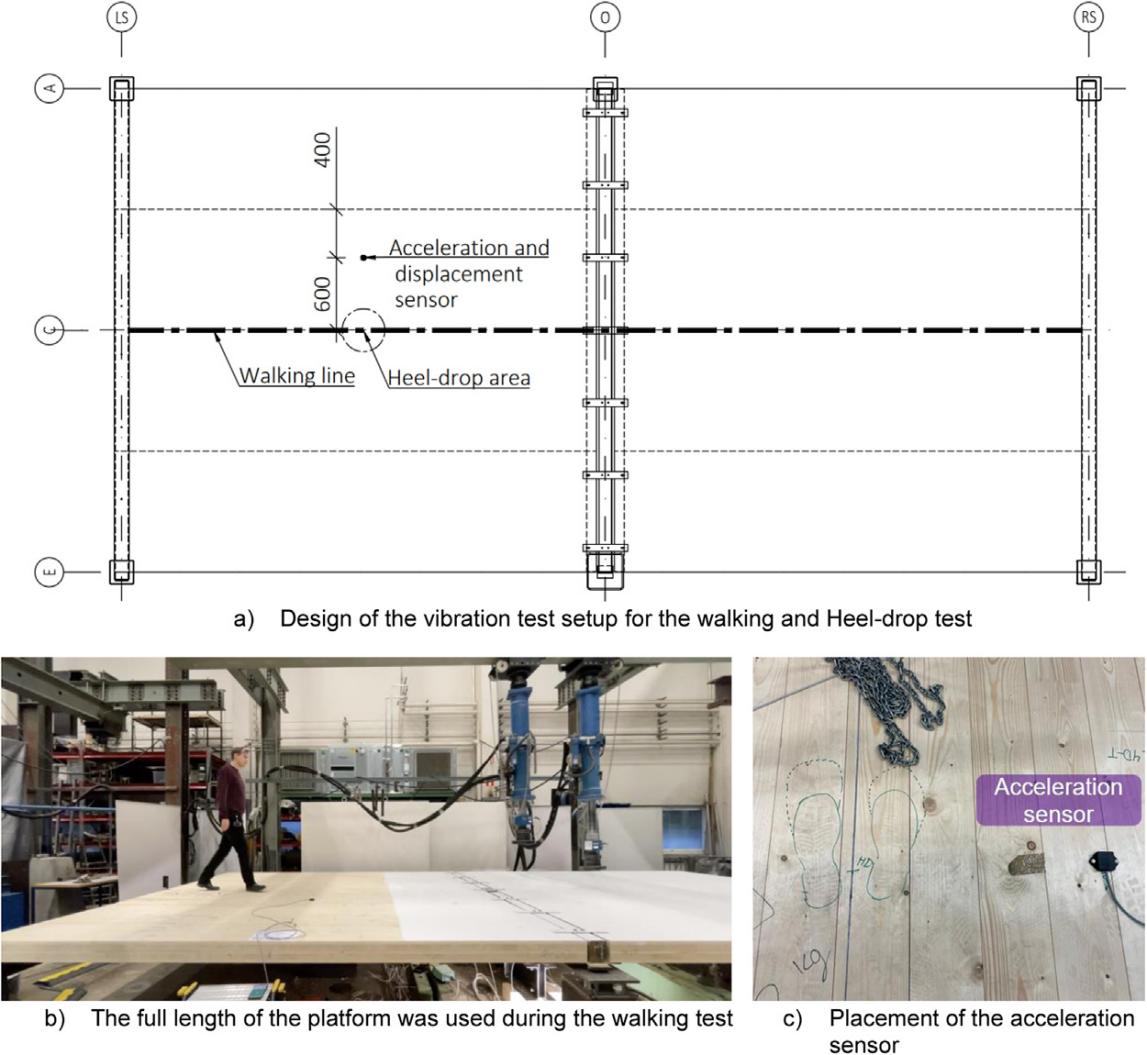
Instrumentation of vibration tests.

Vibration tests were designed and conducted in two phases by following the standard and prior studies [[4], [5], [6]]. Initially, the deflection of the CLT under a 1 kN point load was tested and measured using a displacement sensor positioned at the same location as the load. The deflection was recorded at the mid-span of the central CLT slab and same place was used for Heel-drop tests. In the second phase, heel-drop and walking tests were performed, during which the deflection and acceleration of the platform were recorded. Fig. 21 illustrates the designed and executed placements of the measuring instruments during these two phases.

#### 4.4.3 Push-out

**Fig. 22:**
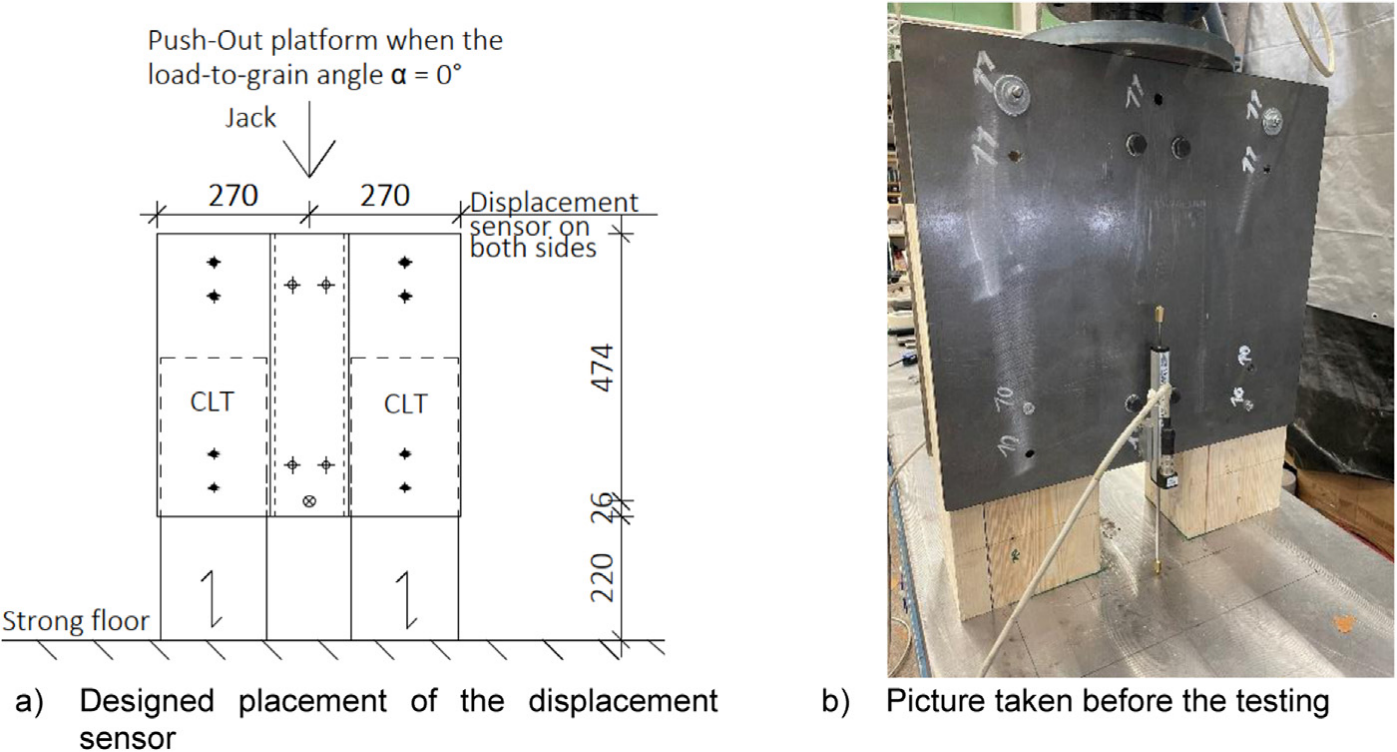
Displacement of the Push-out specimen was recorded by using two sensors.

In the push-out tests, the measured variables were the jack force and displacement, which were recorded using two displacement sensors positioned on either side of the test specimen. Fig. 22 illustrates the positions of the displacement sensors.

### 4.5 Testing procedure

#### 4.5.1 Full-scale bending

The testing procedure for the bending test is separated into two because some tests were conducted only with shear connectors and some first without and then with the connectors as presented in Table 1.

**Test with shear connectors included the following steps**:

1. Installation of the shear connectors
2. Installation of the load-transferring beams
3. Ramping the jack force from 0 to 0,4 *F_y_ where the load was held for 30 s
4. Reducing the jack force from 0,4 *F_y_ to 0,1 *F_y_ where the load was held for 30 s
5. Ramping the jack force from 0,1 *F_y_ to maximum jack load

**Test without shear connectors first and then with the connectors included the following steps:**

1. Installation of the load-transferring beams
2. Ramping the jack force from 0 to 0,4 *F_y_ where the load was held for 30 s
3. Reducing the jack force from 0,4 *F_y_to 0
4. Installation of the shear connectors
5. Ramping the jack force from 0 to 0,4 *F_y_ where the load was held for 30 s
6. Reducing the jack force from 0,4 *F_y_ to 0,1 *F_y_ where the load was held for 30 s
7. Ramping the jack force from 0,1 *F_y_ to maximum jack load

The required force for yielding of the WQ beam, *F_y_*, was estimated using the equations and assumptions presented by Heinisuo, Mela [7], Aspila, Heinisuo [8]. However, this load was warried between tests as can be seen from the datasets. Notably, the failure load value was not reached, and the test was stopped when the maximum load of the jacks (250 kN each) was achieved. During the bending tests, the jack loads were displacement-controlled, with a force increase rate of 0.01 mm/s.

#### 4.5.2 Vibration

The testing procedure for vibration tests included the following steps:

1. 1 kN point load test
2. Heel-drop test
3. Walking test
4. Installation of the shear connector plates
5. 1 kN point load test
6. Heel-drop test
7. Walking test

The vibration tests were conducted both with and without the shear connector plate to determine what is the impact of the shear connectors. For the heel-drop and walking tests, a laboratory staff member weighing 80 kg was used to generate the recorded impact.

#### 4.5.3 Push-out

The testing procedure for the Push-out test included the following steps:

• Ramping the jack force from 0 to 0,4 *F_yield_ where the load was held for 30 s
• Reducing the jack force from 0,4 *F_yield_ to 0,1 *F_yield_ where the load was held for 30 s
• Ramping the jack force from 0,1 *F_yield_ to maximum jack load

For the Push-Out test, the standard [9] and the example of previous studies such as [1,3,10] were followed. The force for the yielding, *F_yield_*, was determined by testing and is referred to as number 0 in the presented datasets.

### 4.6 Test variables

#### 4.6.1 Full-scale bending

**Fig. 23:**
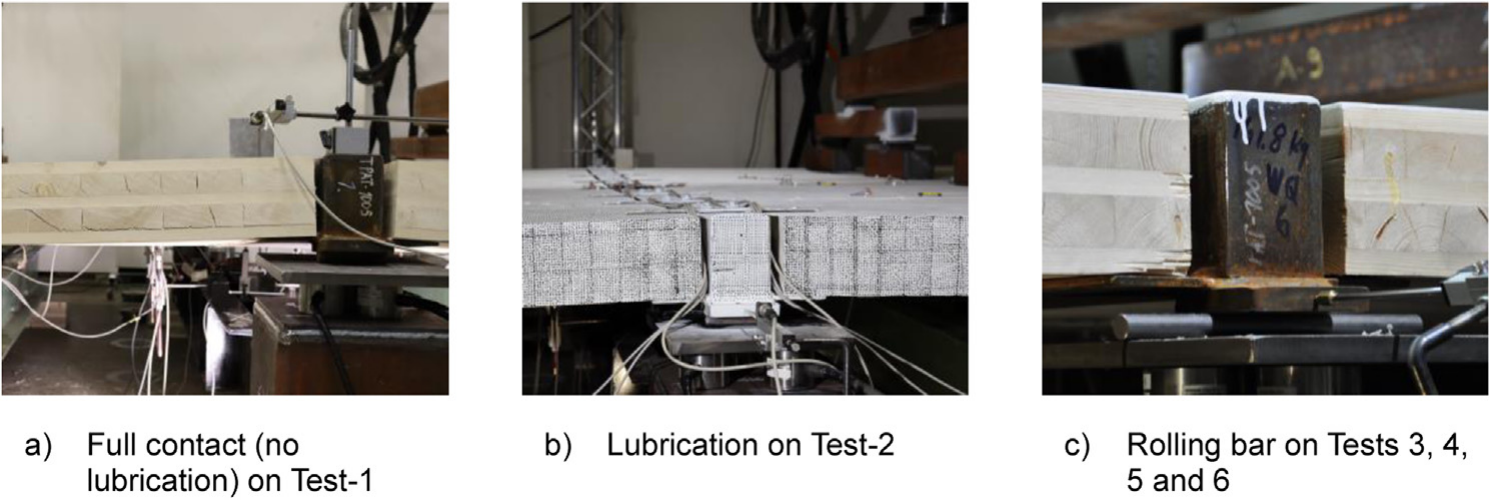
Different modifications, a, b and c for the hinge of the WQ-beam.

In the case of the bending and vibration tests, the most significant variable was the hinge support of the WQ beam. See Table 13 and Fig. 23 for illustrations of the different hinges used during the full-scale tests. Other variables included the natural strength variation between different CLT plates, as evidenced in the push-out test's dataset, and the installation tolerance. To minimise installation tolerance, the same laboratory team assembled all the platforms.

**Table 13:** Testing variables during bending and vibration tests.

	Test-1	Test-2	Test-3	Test-4	Test-5	Test-6
Span length of CLT [m]	4	4	4	2.6	2.6	2.6
Loading line [m]	1.2	1.2	1.2	1.2	0.4	0.4
WQ-beam hinge on Module-A	Full contact	Lubrication	Lubrication	Lubrication	Lubrication	Lubrication
WQ-beam hinge on Module-E	Full contact	Lubrication	Rolling bar	Rolling bar	Rolling bar	Rolling bar
Hinges on support beams	Full contact	Lubrication	Lubrication	Lubrication	Lubrication	Lubrication
Hinges between CLT and support beam	Full contact	Full contact	Full contact	Lubrication	Lubrication	Lubrication

#### 4.6.2 Vibration

The biggest variable during the vibration tests was the induced force to the platform because the heel-drop and walking tests were conducted by using laboratory staff members. Although the person was the same there was always a slight difference regarding the impacting force. Also, the variables listed in Table 13 affected the vibration tests.

#### 4.6.3 Push-out

**Fig. 24:**
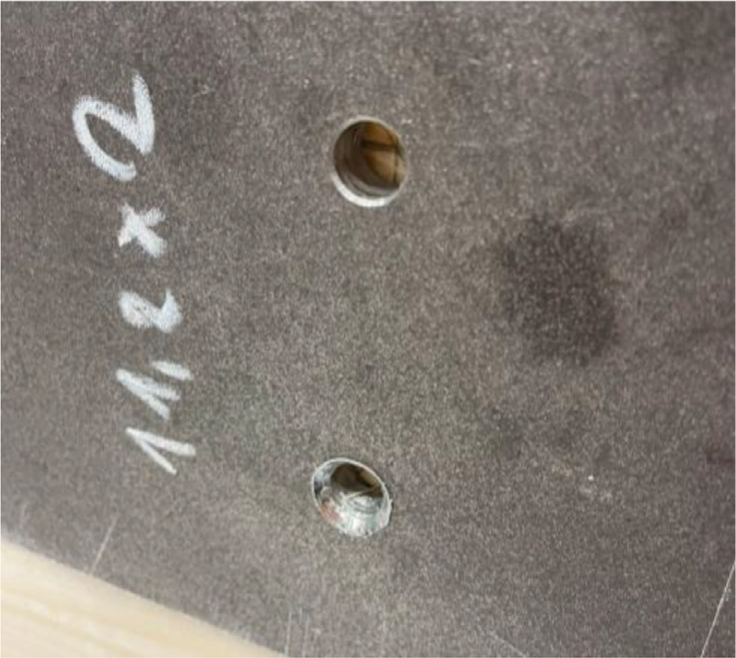
How the embedment of the VGS-types changed during testing.

**Fig. 25:**
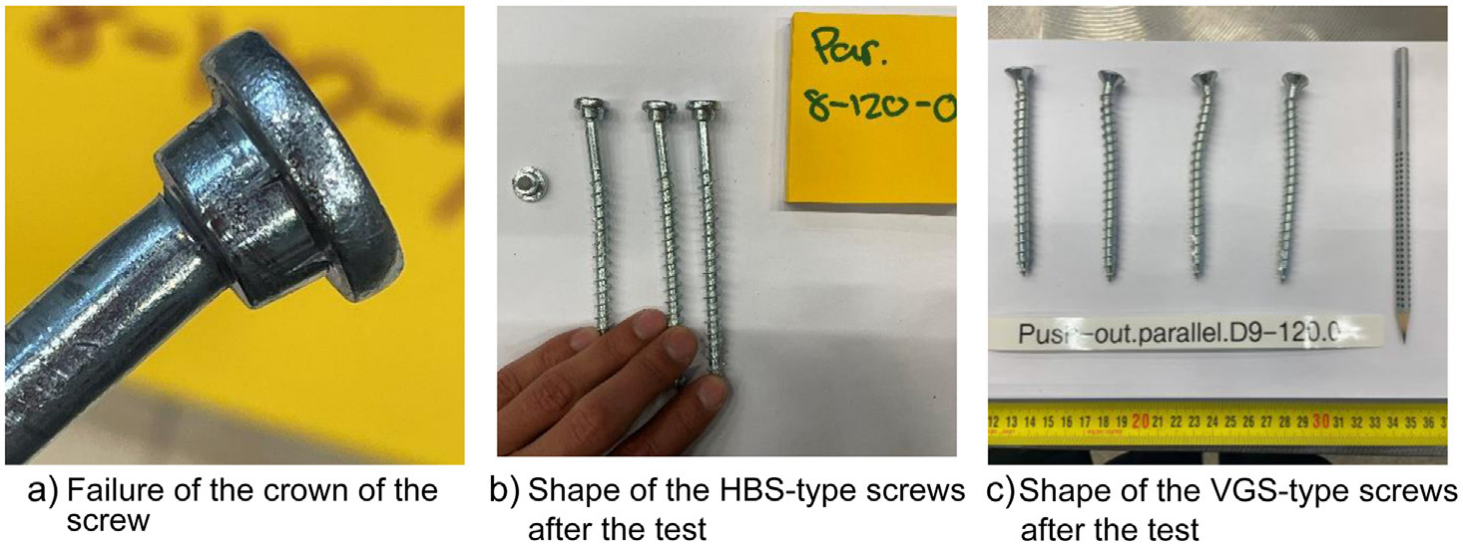
Illustration of how the HBS and VGS-type screws looked after the test.

Push-out tests were conducted using two types of screws: HBS-type and VGS-type. The HBS-type screws were designed for use with steel plates and did not require additional embedding into the steel plate. In contrast, the VGS-type screws were intended for timber-to-timber connections and required embedding. However, the initial embedment was inadequate, and as successive screws were tested, the embedment size increased. Fig. 24. How the embedment of the VGS-types changed during testing. Fig. 24 illustrates the changes in embedment during the testing process. This increase in embedment did not affect the total load capacity, as the tests were stopped once the required displacement was reached, unlike the HBS-type screws which tended to break at the crown part more frequently, see Fig. 25.

## Limitations

None.

## Ethics Statement

The authors have read and followed the ethical requirements for publication in Data in Brief and thus are confirming that the presented work does not involve human subjects, animal experiments, or any data collection from social media platforms.

## CRediT Author Statement

**Aku Aspila:** Methodology, Validation, Formal analysis, Investigation, Data Curation, Writing - Original Draft, Visualization, Supervision, Project administration, **Markku Heinisuo:** Conceptualization, Methodology, Resources, Writing - Review & Editing, **Virpi Leivo:** Validation, Investigation, Resources, Writing - Original Draft, **Mikko Malaska:** Writing - Review & Editing, **Kristo Mela:** Methodology, Resources, Writing - Review & Editing, **Sami Pajunen:** Methodology, Resources, Writing - Original Draft, Supervision, Funding acquisition, **Mika Vuorela:** Validation, Investigation, Resources, Writing - Review & Editing.

## Data Availability

ZenodoCollected Data on Bending, Vibration, and Push-out Tests of Shallow Steel-Timber Composite Beams – Nordic System (Original data).

## Appendix Supplementary materials

Image, application 1

Image, application 2

## References

[1] Asiz A., Smith I. (2011). Connection system of massive timber elements used in horizontal slabs of hybrid tall buildings. J. Struct. Eng.-ASCE.

[2] Hassanieh A., Valipour H.R., Bradford M.A. (2016). Load-slip behaviour of steel-cross laminated timber (CLT) composite connections. J. Constr. Steel. Res..

[3] Yang R., Li H.T., Lorenzo R., Ashraf M., Sun Y.F., Yuan Q. (2020). Mechanical behaviour of steel timber composite shear connections. Constr. Build. Mater..

[4] Hassanieh A. (2019). Vibration behaviour of steel-timber composite floors, part (2): evaluation of human-induced vibrations. J. Constr. Steel. Res..

[5] Toratti T., Talja A. (2016). Classification of human induced floor vibrations. Build. Acoust..

[6] CEN (2018).

[7] Heinisuo M. (2019). XII Conference on Steel and Composite Construction.

[8] Aspila A. (2022). Elastic design of steel-timber composite beams. Wood. Mater. Sci. Eng..

[9] CEN, Timber structures. Joints made with mechanical fasteners. General principles for the determination of strength and deformation characteristics (ISO 6891:1983), in EN 26891. 2014.

[10] Hassanieh A., Valipour H.R., Bradford M.A. (2017). Composite connections between CLT slab and steel beam: experiments and empirical models. J. Constr. Steel. Res..

