# Supplementary material for: Collected data on bending, vibration, and push-out tests of shallow steel-timber composite beams—Nordic system
Source: Data Brief. 2024 Nov 26;57:111172. doi: 10.1016/j.dib.2024.111172 (PMC11683325; doi:10.1016/j.dib.2024.111172)

Content:

M1: Platform 1 measurements
M2: Platform 2 (loading line at 0,4m) measurements
M3: Platform 1 & 2 strains gauges placement
M4: Platform 1 & 2 Horizontal and vertical displacements
M5: Walking and Heel-drop tests
S1: A-A
S2: B-B & E-E
S3: C-C
S4: D-D
X1: Push-out platform
X2: Push-out test when using HBS-type screws
X3: Push-out test when using VGS-type screws

TUNN.	LUKUM.	MUUTOS	SUUNN.	PVM.	TARK./HYVÄKS.

K.OSA/KYLÄ	KORTTELI/TILA	TONTTI/RNo	RAKENNUSLUVAN TUNNUS					
RAKENNUKSEN TAI RAKENNUSTEN NUMEROT TAI TUNNUKSET								
				JUOKSEVA NRO				
Nordic system tests<br>Tampere University			MITTAKAAVAT ENNEN PIEN.					
			TARKASTAJA	SUUNNITTELUALA				
			HYVÄKSYJÄ	SUUNN. TYÖN NRO 1				
PVM. 25.09.23	PIIRTÄJÄ FIAKUA	SUUNNITTELIJA AKU_ASPILA	S.LAJI	LOHKO	KRS	LAJI	NRO	MUUTOS

DWG

		SUUNN. TYÖN NRO<br>95		M1
		PVM.<br>16.07.2024	PIIRTÄJÄ<br>FIAKUA	
KOHDE<br>Nordic system test<br>Tampere University		SISÄLTÖ<br>Platform 1<br>Scale 1:30<br>.		

Platform 1. 1:30

Tests 1, 2, 3 and 4

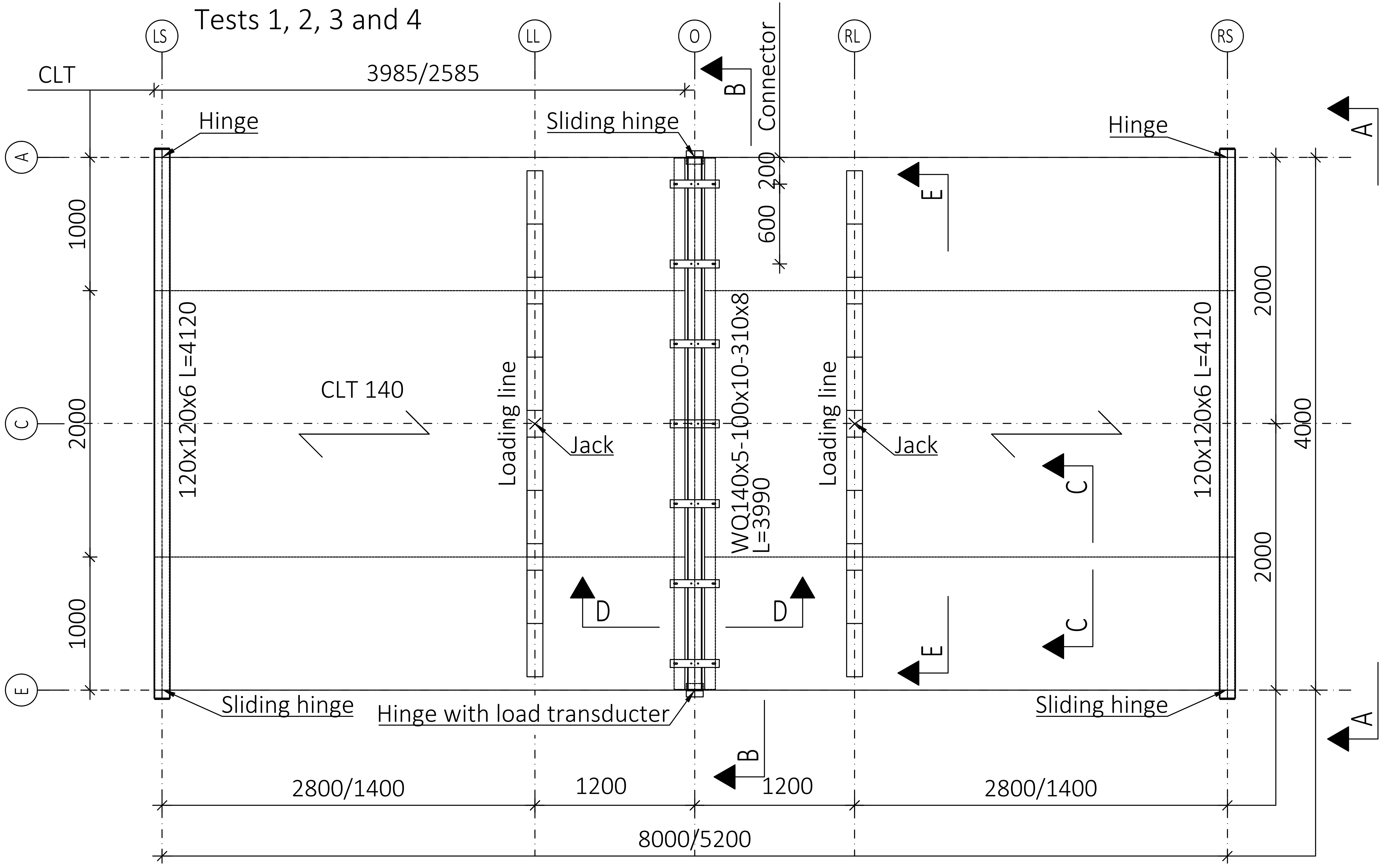

		SUUNN. TYÖN NRO<br>95		M2
		PVM.<br>16.07.2024	PIIRTÄJÄ<br>FIAKUA	
KOHDE<br>Nordic system test<br>Tampere University		SISÄLTÖ<br>Platform 2<br>Scale 1:30<br>.		

Platform 2. 1:30

Tests 5 and 6

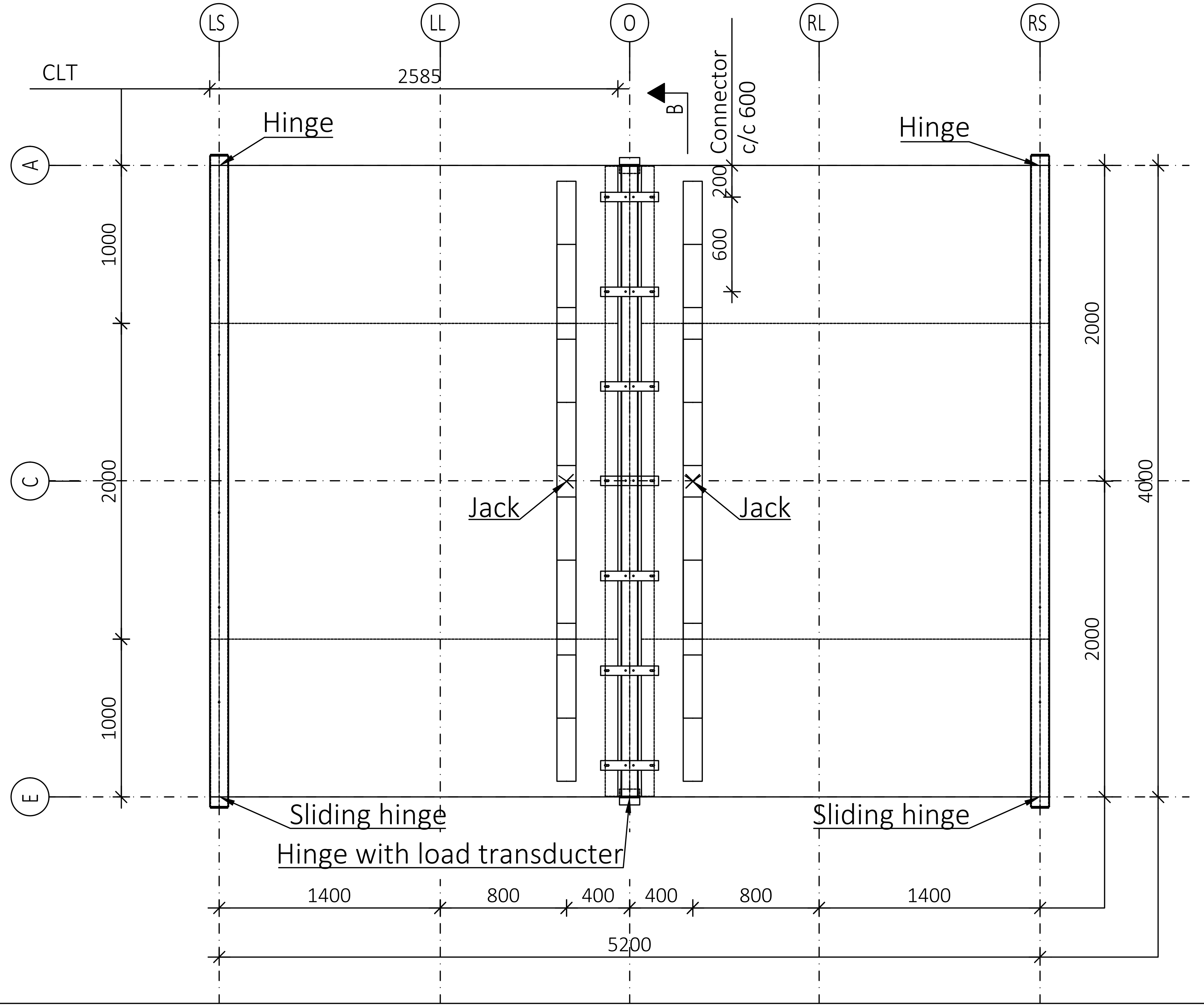

		SUUNN. TYÖN NRO 95	M3
		PVM. 16.07.2024 / PIIRTÄJÄ FIAKUA	
KOHDE Nordic system test Tampere University		SISÄLTÖ Strain gauges placement Scale 1:30 .	

A-A 1:10

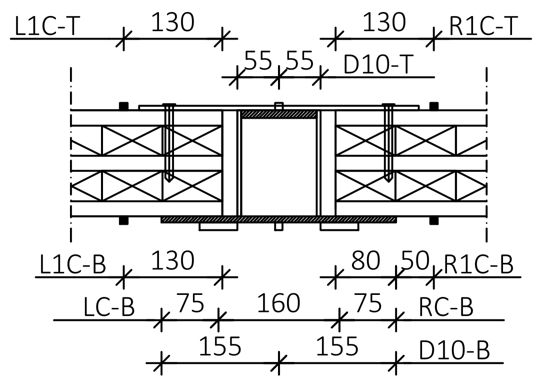

DET A 1:20

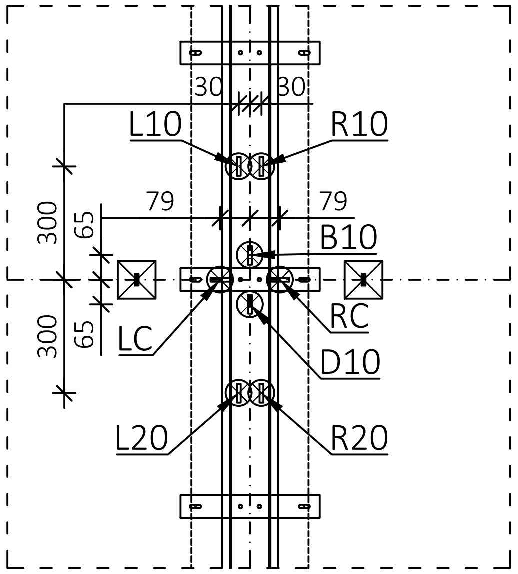

Platform 1 and 2

Strain gauges placement

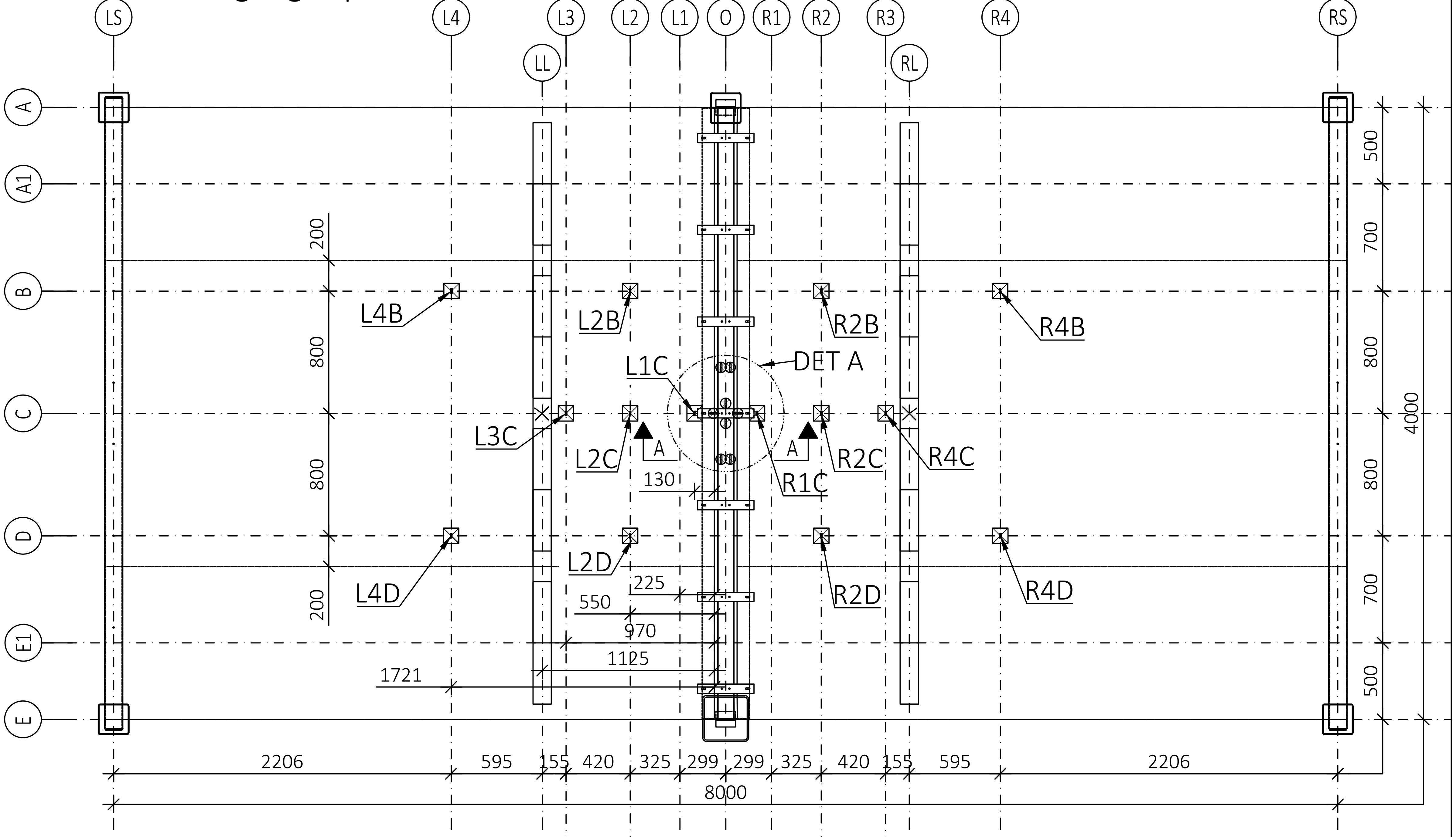

☒ Wood strain gauges, on top and bottom surface. Total of 28

⊗ Steel strain gauges, see section. Total of 6 in Test 1 and 2, 8 in Test 3 and 10 in Tests 4, 5 and 6

		SUUNN. TYÖN NRO<br>95		M4
		PVM.<br>16.07.2024	PIIRTÄJÄ<br>FIAKUA	
KOHDE<br>Nordic system test<br>Tampere University		SISÄLTÖ<br>Horizontal and vertical displacemen sensors<br>Scale 1:30<br>.		

Platform 1 and 2

Horizontal and vertical displacement sensors

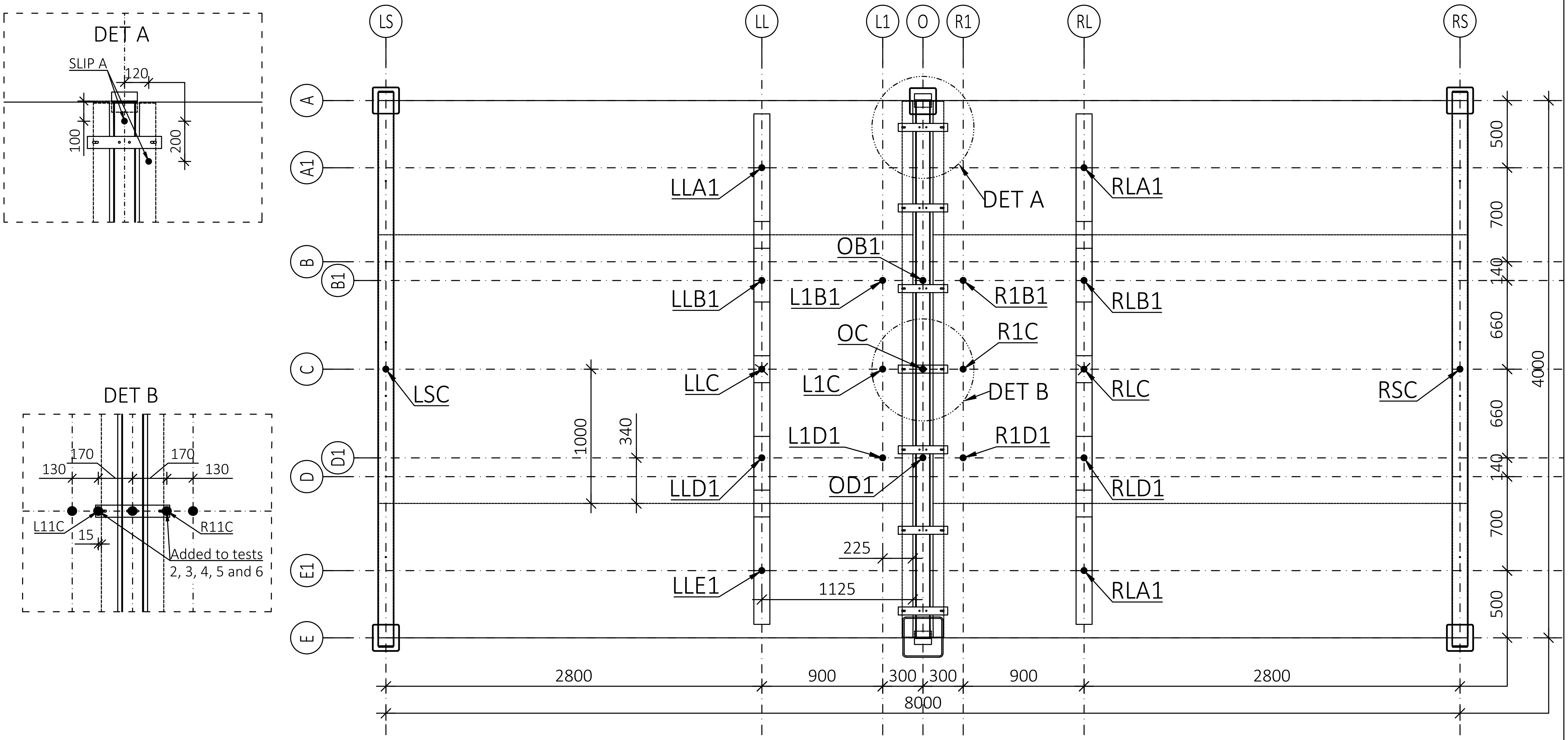

Total of 21 horizontal measuring points

		SUUNN. TYÖN NRO<br>95		M5
		PVM.<br>16.07.2024	PIIRTÄJÄ<br>FIAKUA	
KOHDE<br>Nordic system test<br>Tampere University		SISÄLTÖ<br>Walking and Heel–drop tests<br>Scale 1:30<br>.		

Platform 1 & 2

Walking and Heel-drop tests

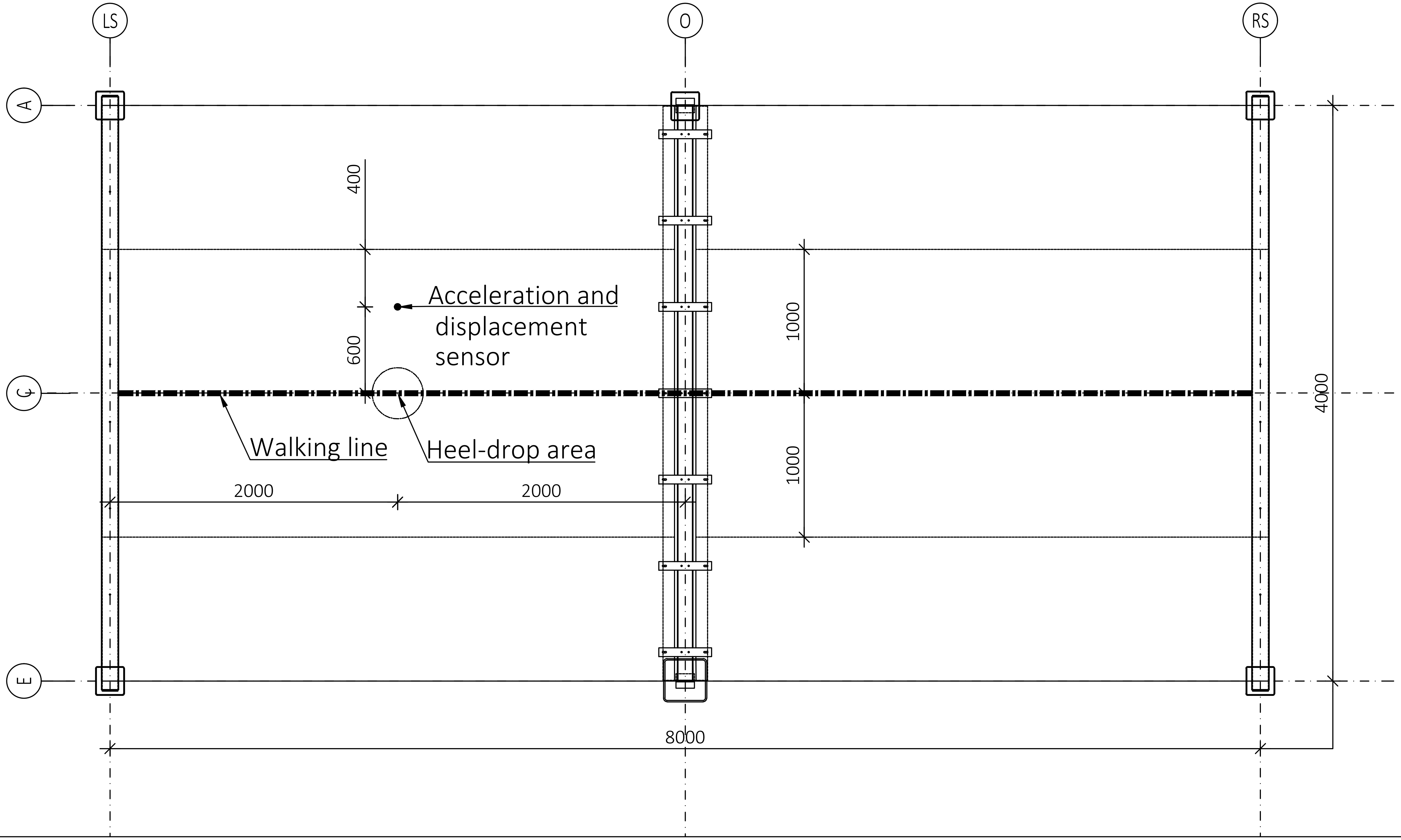

		SUUNN. TYÖN NRO<br>95		S1
		PVM.<br>16.07.2024	PIIRTÄJÄ<br>FIAKUA	
KOHDE<br>Nordic system test<br>Tampere University		SISÄLTÖ<br>Section A–A<br>Scale 1:10<br>.		

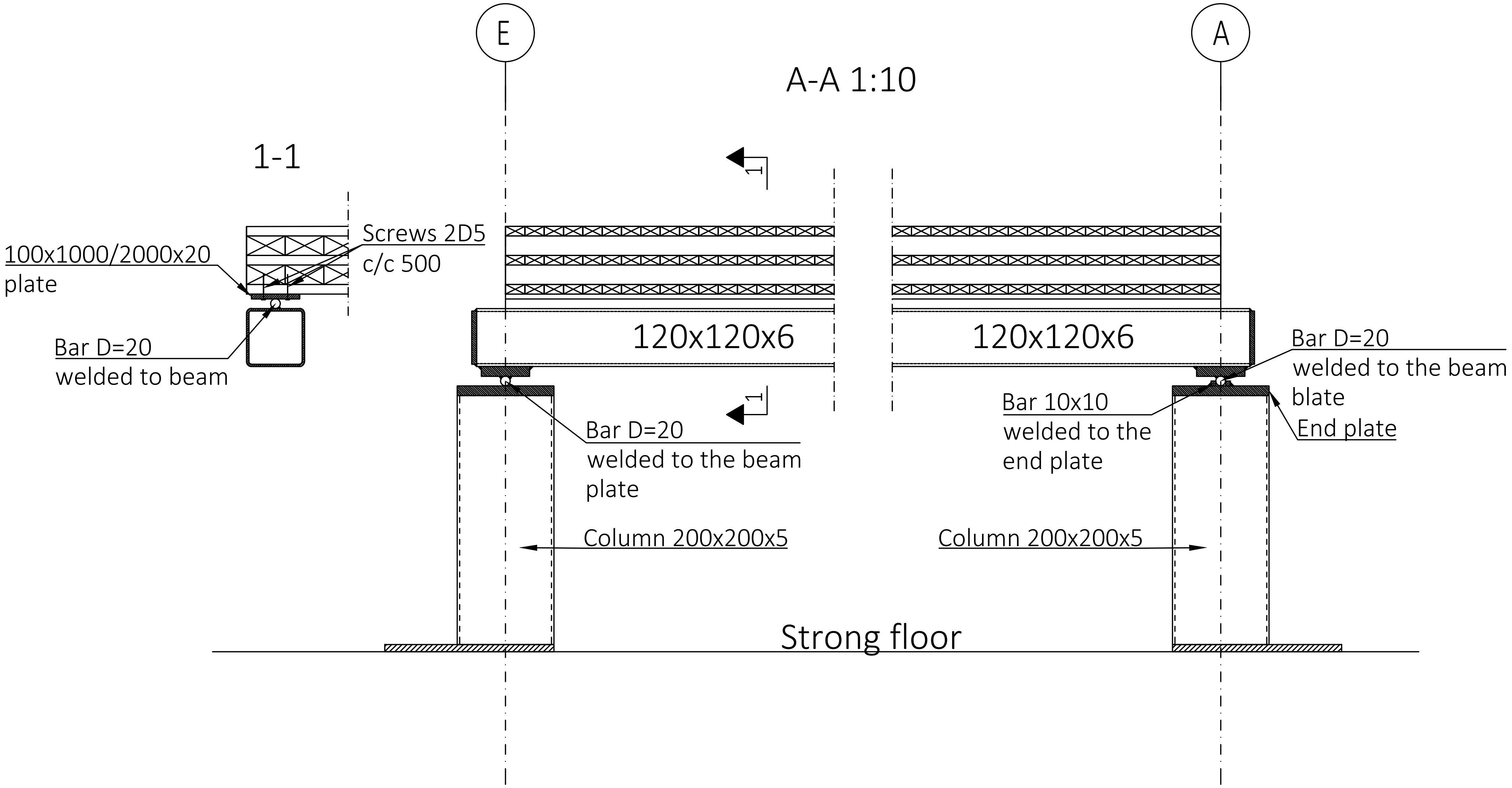

		SUUNN. TYÖN NRO<br>95		S2
		PVM.<br>16.07.2024	PIIRTÄJÄ<br>FIAKUA	
KOHDE<br>Nordic system test<br>Tampere University		SISÄLTÖ<br>Section B–B<br>Scale 1:10<br>.		

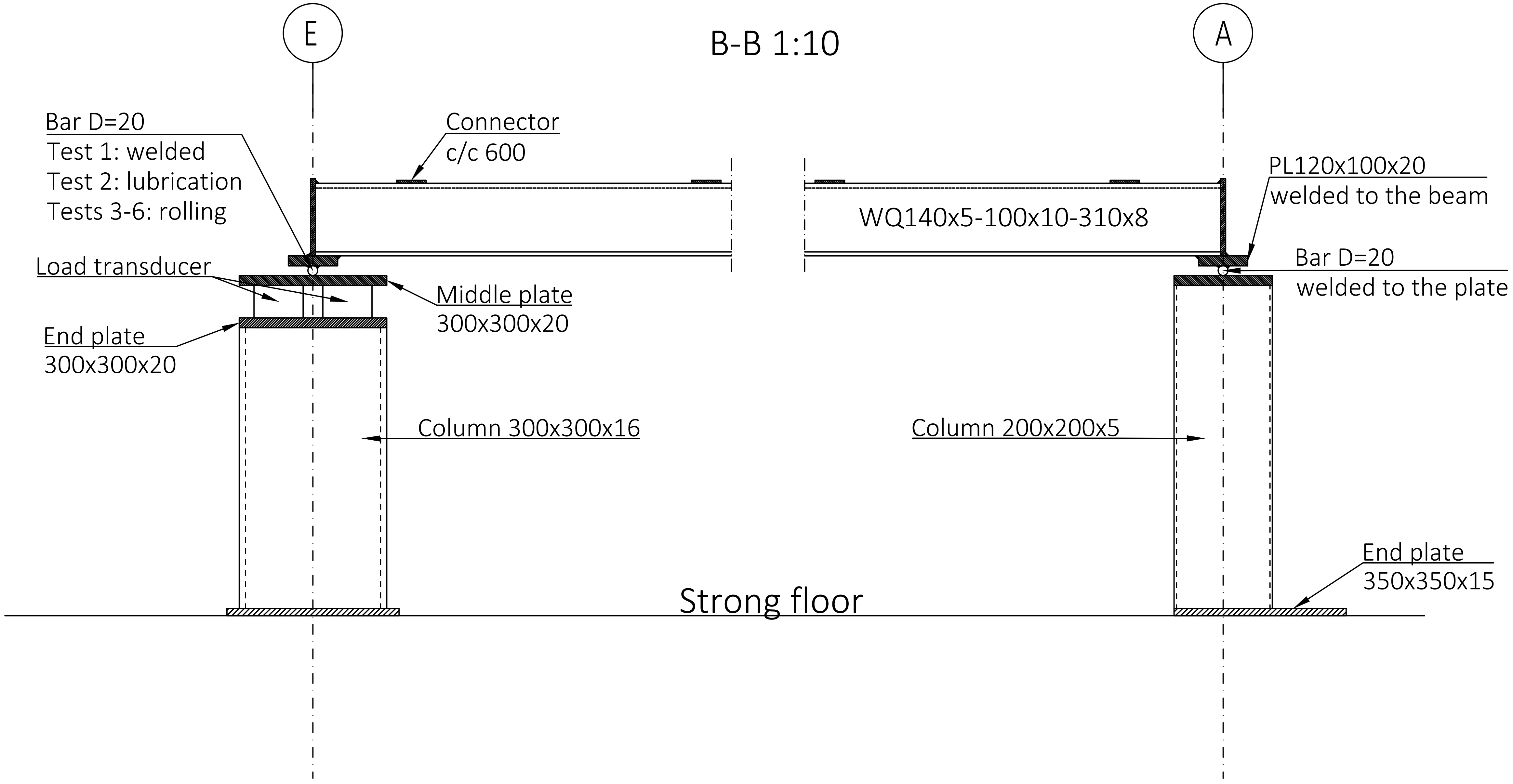

		SUUNN. TYÖN NRO<br>95	S3
		PVM.<br>16.07.2024 / PIIRTÄJÄ<br>FIAKUA	
KOHDE<br>Nordic system test<br>Tampere University		SISÄLTÖ<br>Section C–C & D–D<br>Scale 1:5<br>.	

C-C 1:5

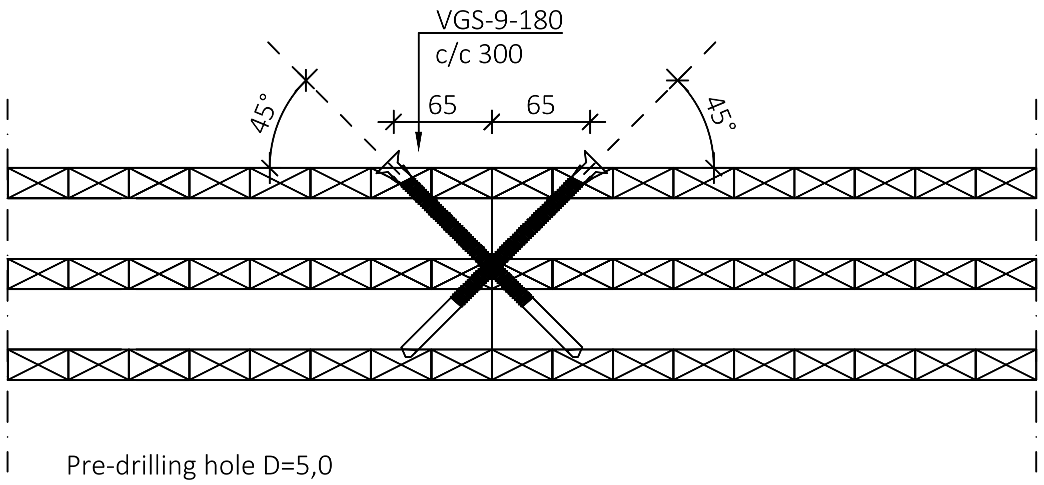

D-D 1:5

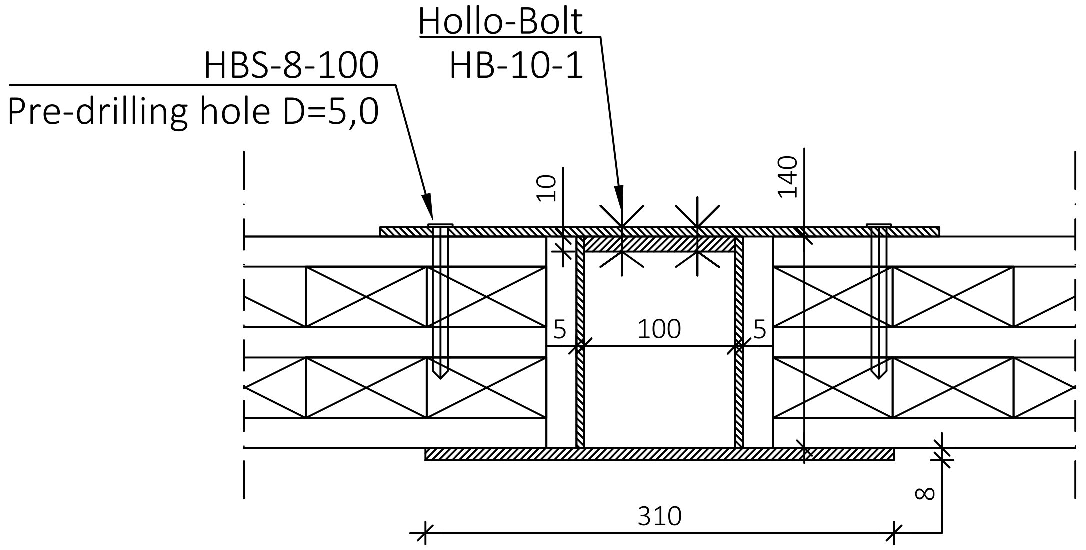

Connector plate t= 6mm

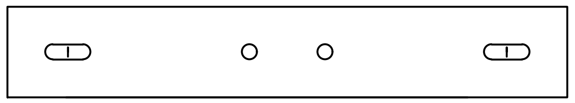

		SUUNN. TYÖN NRO<br>95	S4
		PVM.<br>16.07.2024 / PIIRTÄJÄ<br>FIAKUA	
KOHDE<br>Nordic system test<br>Tampere University		SISÄLTÖ<br>Section D–D<br>Scale 1:15<br>.	

D-D 1:15
Jack

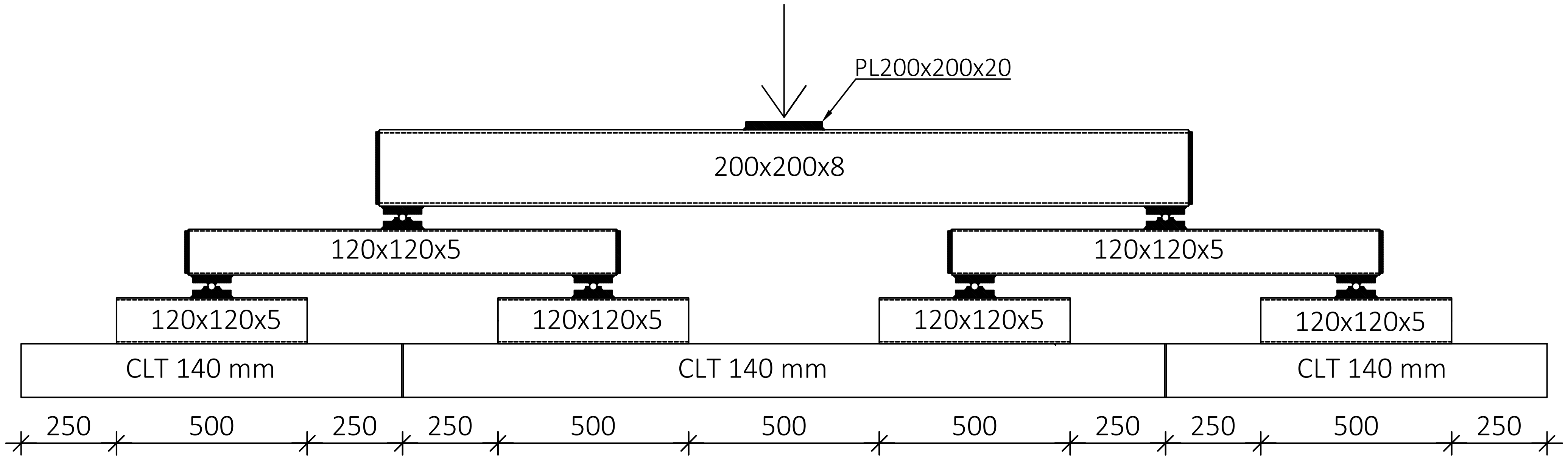

Left load bearing beam

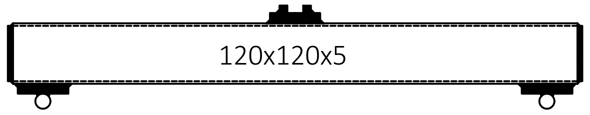

Right load bearing beam

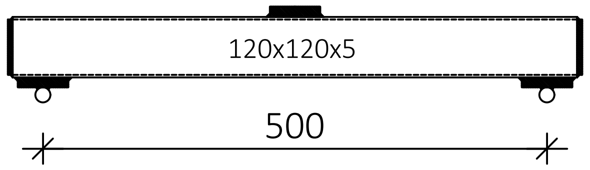

		SUUNN. TYÖN NRO<br>95	X1
		PVM.<br>16.07.2024 / PIIRTÄJÄ<br>FIAKUA	
KOHDE<br>Nordic system test<br>Tampere University		SISÄLTÖ<br>Push–Out<br>Scale 1:10<br>.	

HBS-type screw

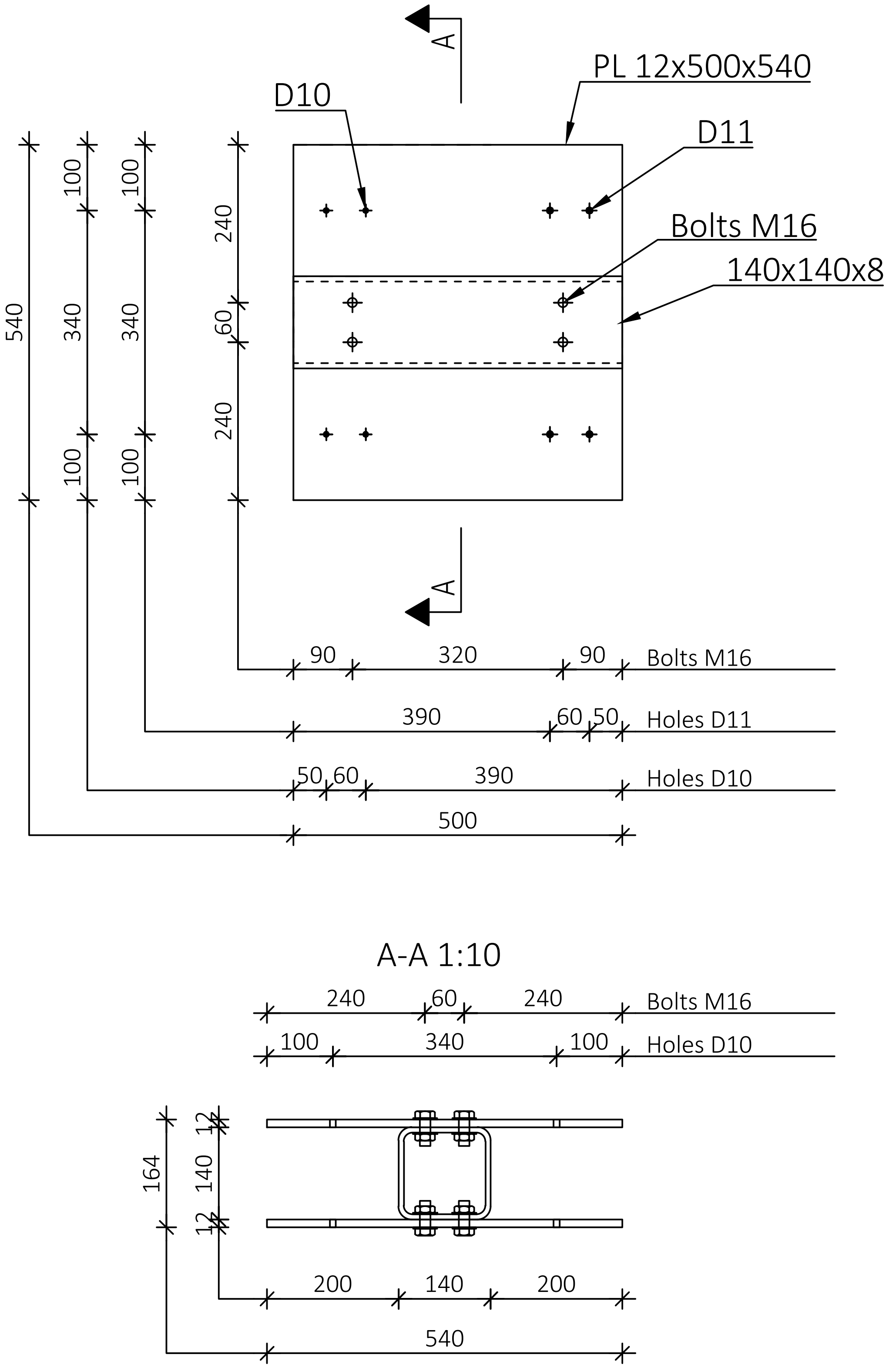

VGS-type screw

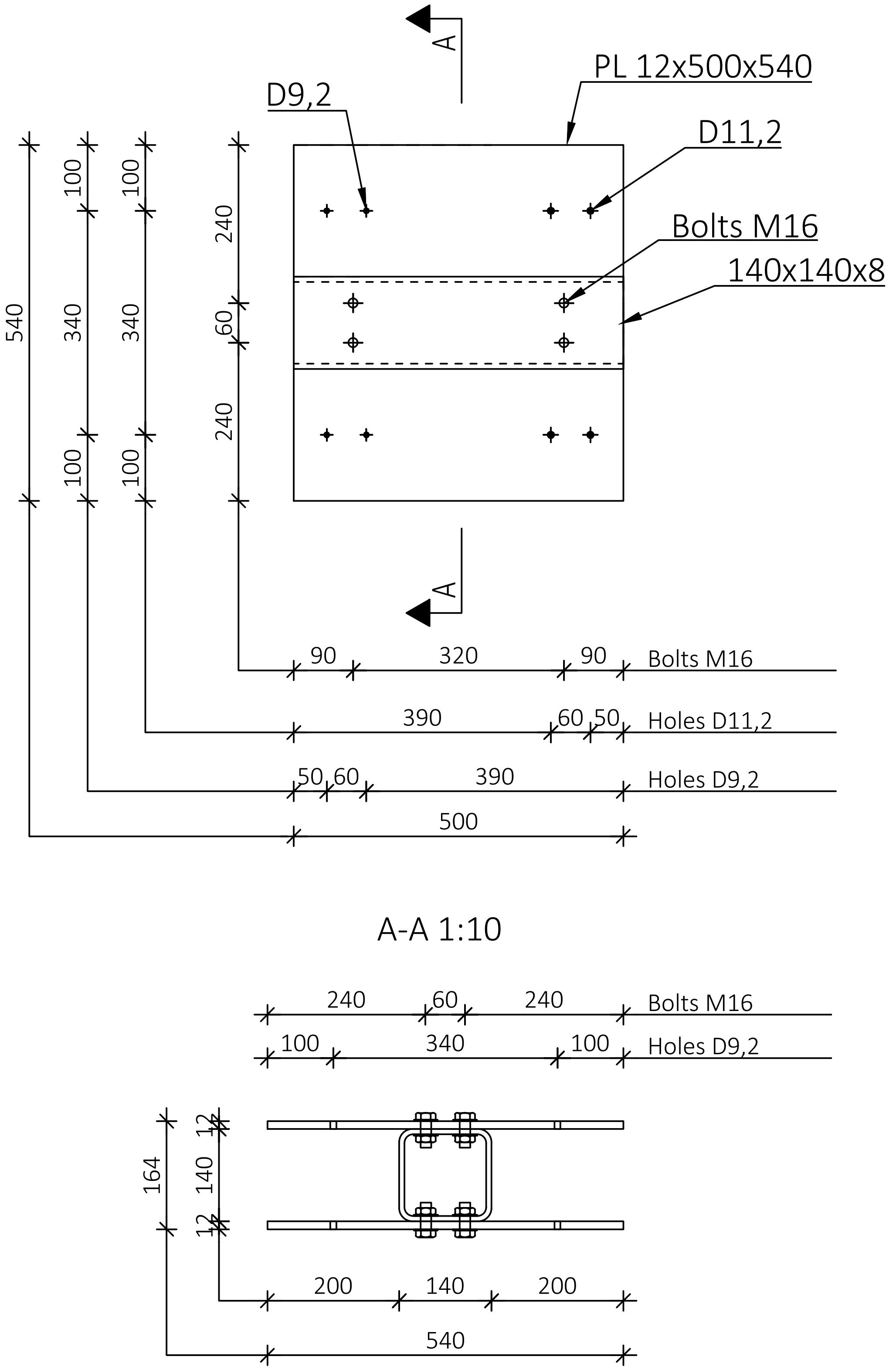

		SUUNN. TYÖN NRO<br>95		X2
		PVM.<br>16.07.2024	PIIRTÄJÄ<br>FIAKUA	
KOHDE<br>Nordic system test<br>Tampere University		SISÄLTÖ<br>Push–Out, test when using HBS–type srews<br>Scale 1:10		

HBS-type screw

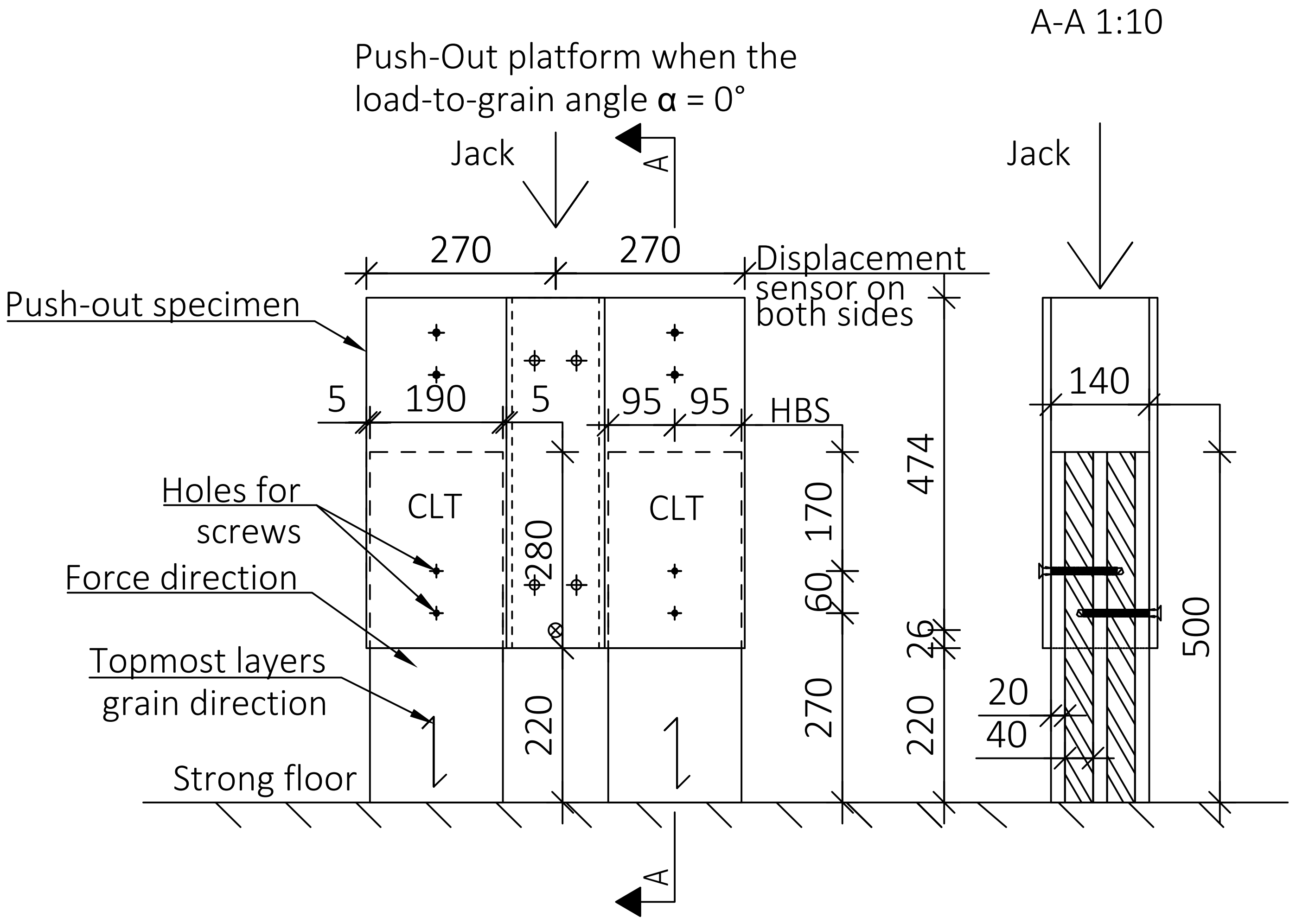

HBS-type screw

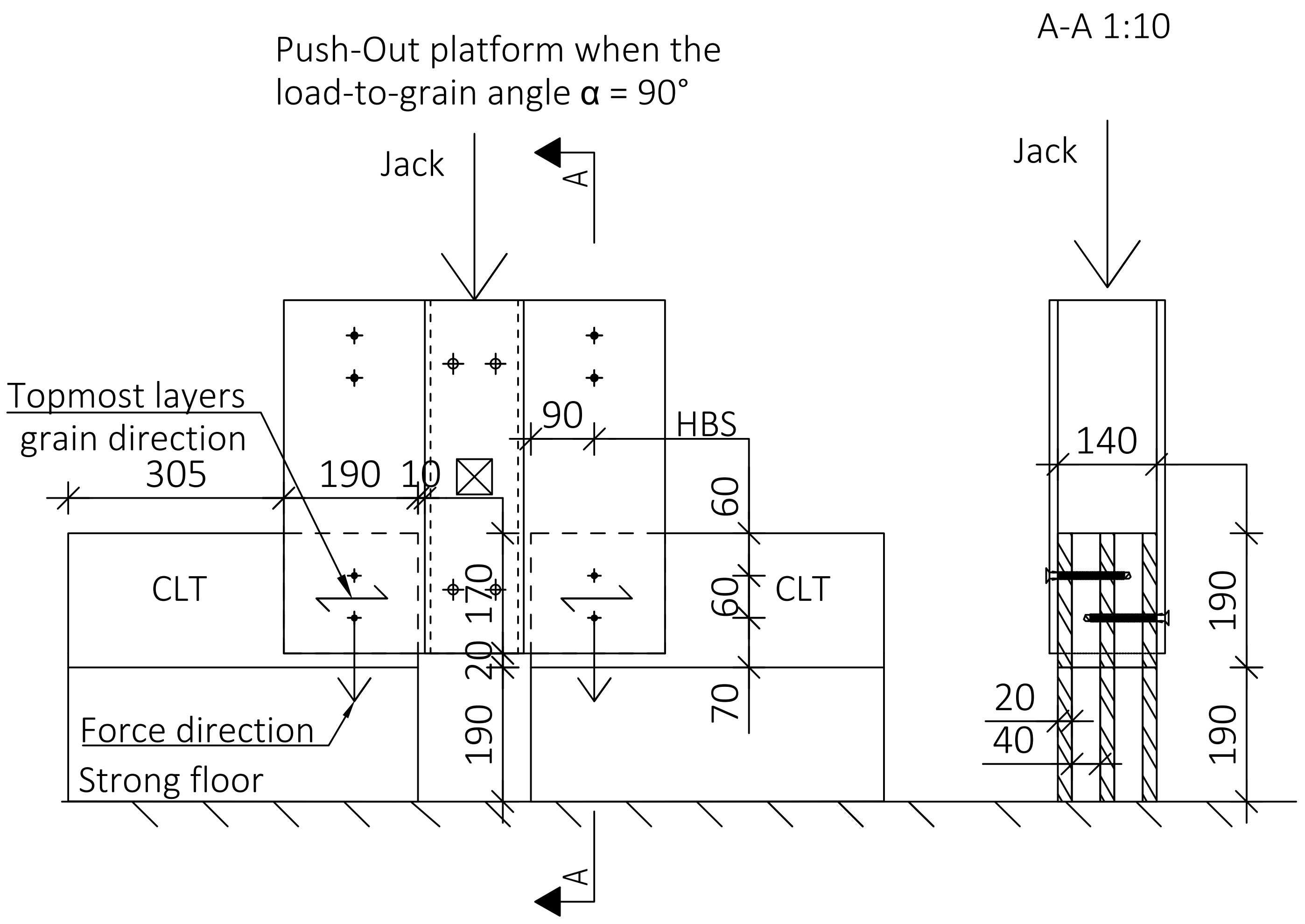

		SUUNN. TYÖN NRO<br>95<br>PVM. 16.07.2024 / PIIRTÄJÄ FIAKUA	X3
KOHDE<br>Nordic system test<br>Tampere University		SISÄLTÖ<br>Push–Out, test when using VGS–type srews<br>Scale 1:10	

VGS-type screw

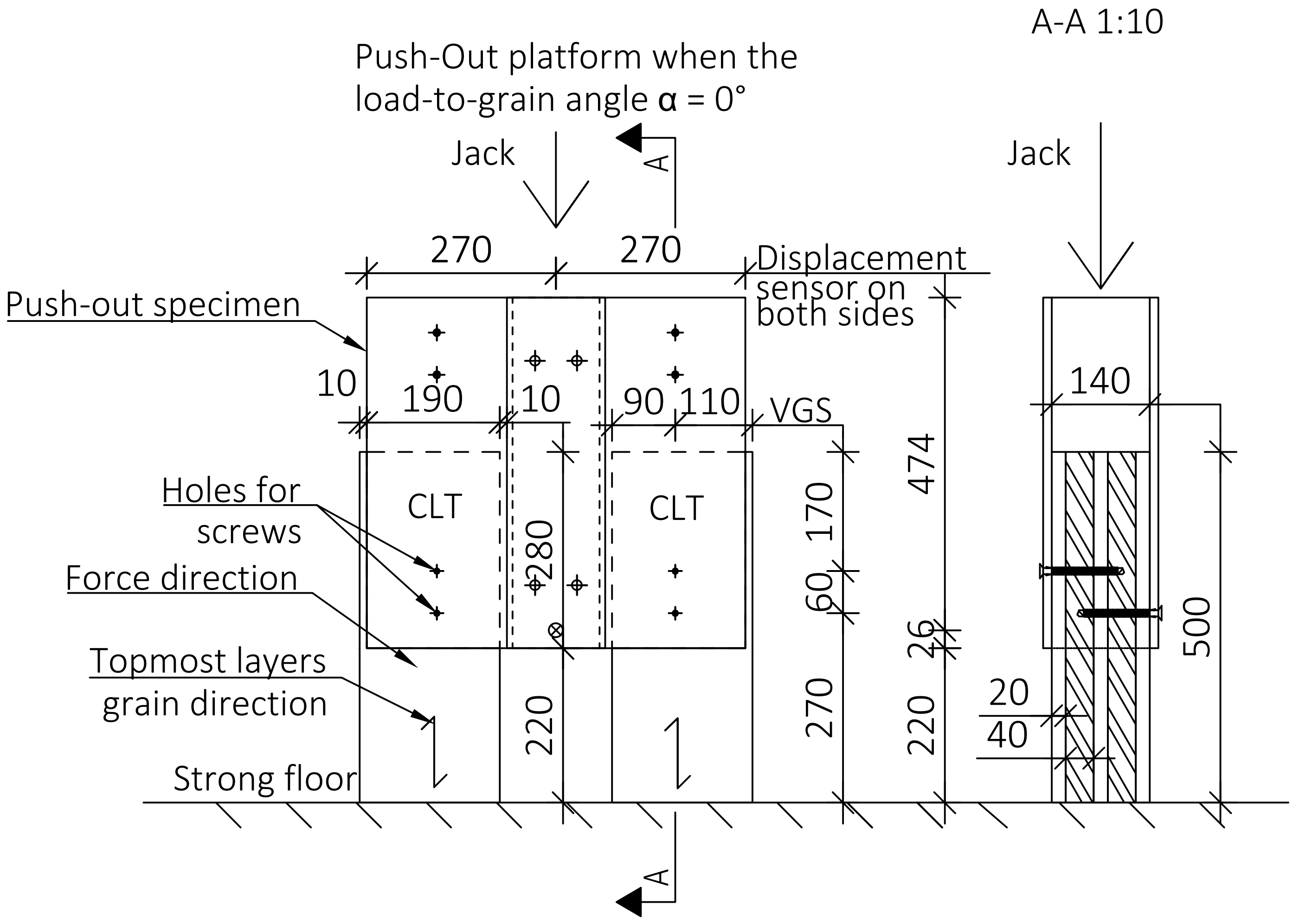

VGS-type screw

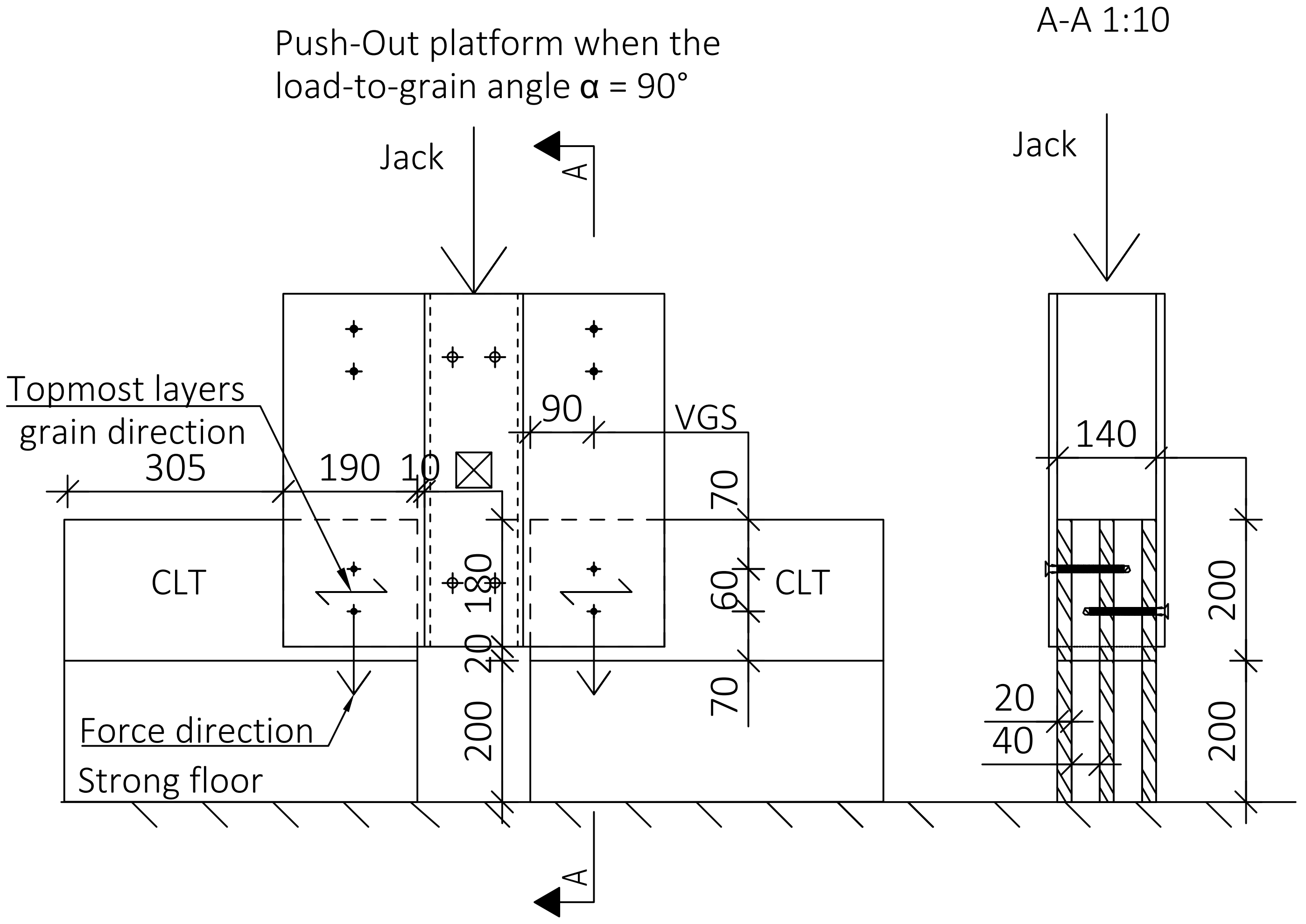

Supplement: Supplementary file 1 [file mmc1.pdf]
