# Supplementary material for: Collected data on bending, vibration, and push-out tests of shallow steel-timber composite beams—Nordic system
Source: Data Brief. 2024 Nov 26;57:111172. doi: 10.1016/j.dib.2024.111172 (PMC11683325; doi:10.1016/j.dib.2024.111172)
Supplement: Supplementary file 2 [file mmc2.pdf]

HBS PLATE

PAN HEAD SCREW FOR PLATES

BIT INCLUDED | MY PROJECT SOFTWARE | ETA-11/0030

HBS P
Designed for steel-to-timber joints: the head has a shoulder and the thickness is increased for completely safe, reliable fastening plates to the timber.

PLATE FASTENING
The under-head shoulder achieves an interlocking effect with the circular hole in the plate, thus guaranteeing excellent static performance.

LONGER THREAD
Increased thread length for excellent shear strength and tensile strength in steel-to-timber joints. Values higher than normal.

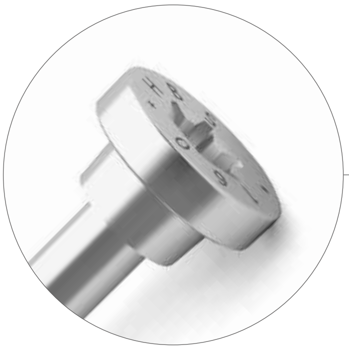

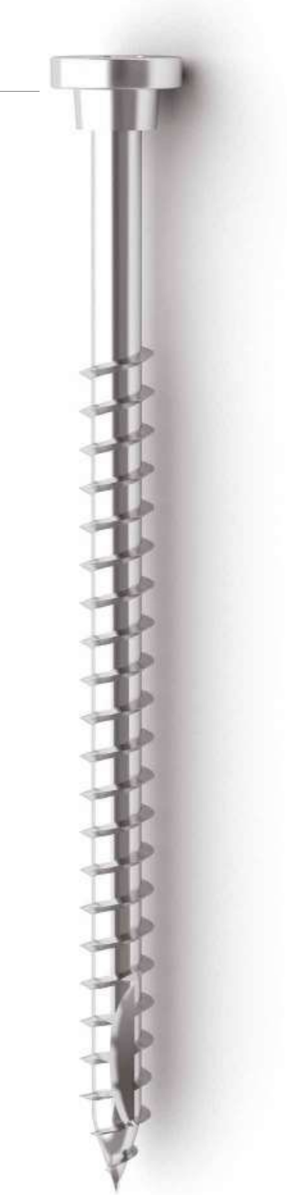

CHARACTERISTICS

**FOCUS**	steel-to-timber joints
**HEAD**	shoulder for plate
**DIAMETER**	from 8,0 to 12,0 mm
**LENGTH**	from 80 to 200 mm

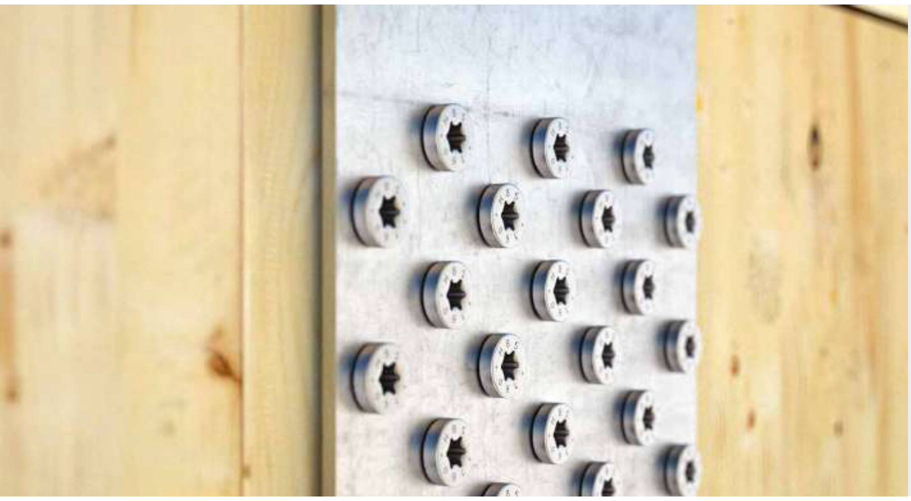

MATERIAL
Galvanized carbon steel.

FIELDS OF USE
- timber based panels
- solid timber
- glulam (Glued Laminated Timber)
- CLT, LVL
- high density woods

Service classes 1 and 2.

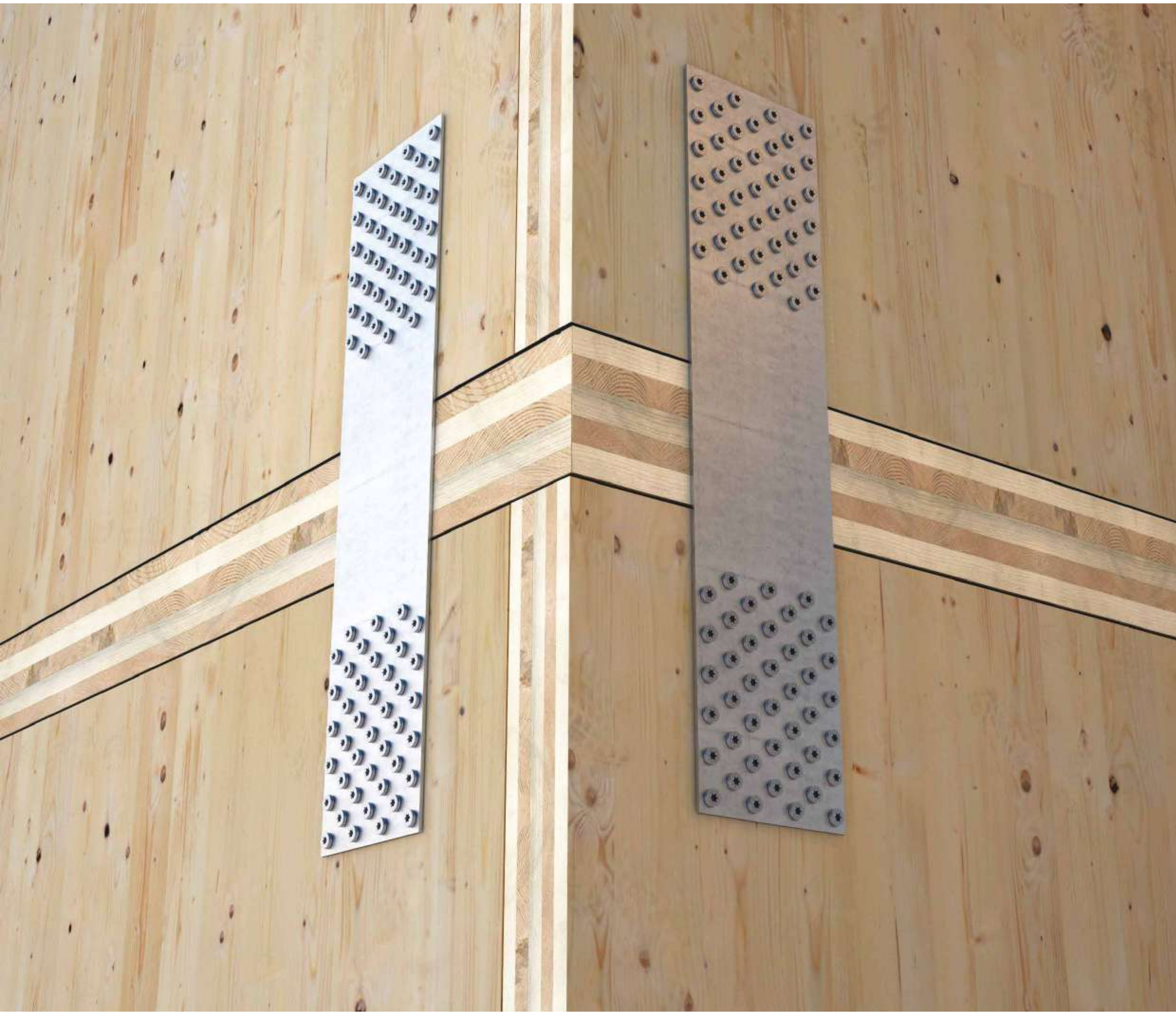

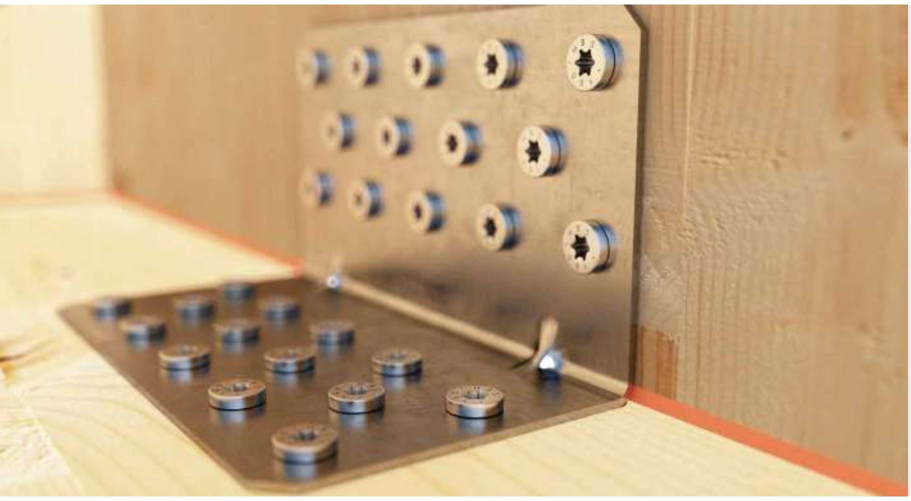

MULTISTOREY

Ideal for steel-to-timber joints with large customized plates, designed for multi-story timber buildings.

TITAN

Values also tested, certified and calculated for fastening standard Rothoblaas plates.

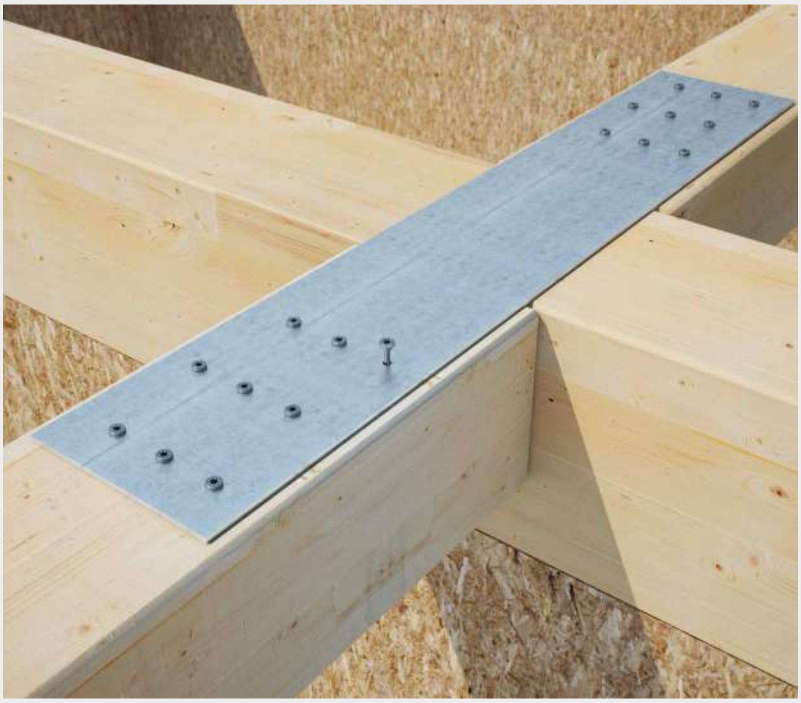

Steel-to-timber shear joint

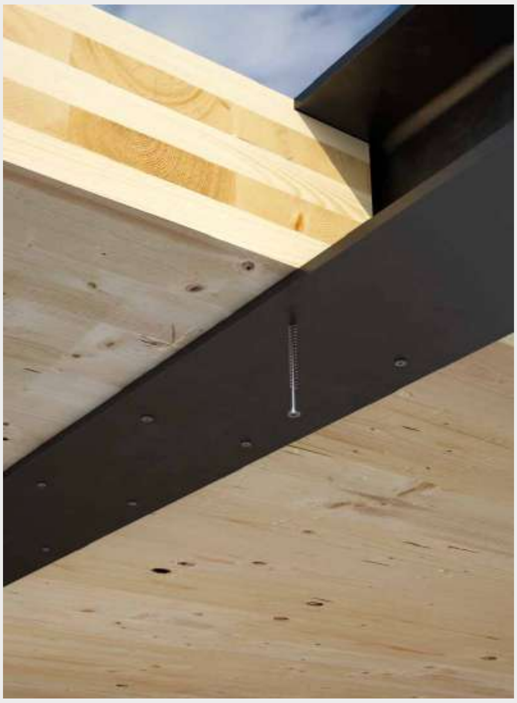

Mixed steel-to-timber structural joint

GEOMETRY AND MECHANICAL CHARACTERISTICS

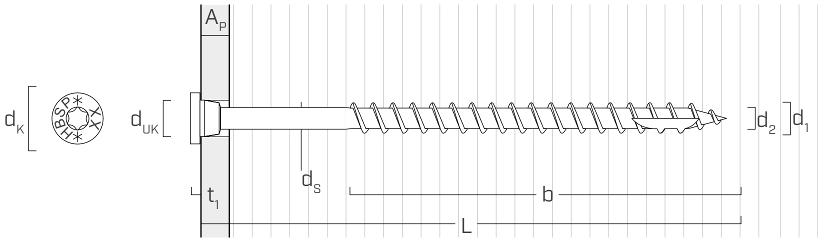

Nominal diameter	d_1	[mm]	8	10	12
Head diameter	d_K	[mm]	14,50	18,25	20,75
Tip diameter	d_2	[mm]	5,40	6,40	6,80
Shank diameter	d_S	[mm]	5,80	7,00	8,00
Head thickness	t_1	[mm]	3,40	4,35	5,00
Underhead diameter	d_{UK}	[mm]	10,00	12,00	14,00
Pre-drilling hole diameter(1)	d_V	[mm]	5,0	6,0	7,0
Recommended hole diameter on steel plate	$d_{V,steel}$	[mm]	11,0	13,0	15,0
Characteristic yield moment	$M_{y,k}$	[Nm]	20,1	35,8	48,0
Characteristic withdrawal-resistance parameter(2)	$f_{ax,k}$	[N/mm²]	11,7	11,7	11,7
Associated density	ρ_a	[kg/m³]	350	350	350
Characteristic head-pull-through parameter(2)	$f_{head,k}$	[N/mm²]	10,5	10,5	10,5
Associated density	ρ_a	[kg/m³]	350	350	350
Characteristic tensile strength	$f_{tens,k}$	[kN]	20,1	31,4	33,9

(1) Pre-drilling valid for softwood.
(2) Valid for softwood - maximum density 440 kg/m³.
For applications with different materials or with high density please see ETA-11/0030.

CODES AND DIMENSIONS

d_1 [mm] [in]	CODE	L [mm]	L [in]	b [mm]	A_P [mm]	pcs
8<br>0.32<br>TX 40	HBSP880	80	3 1/8	55	1.0 ÷ 15.0	100
	HBSP8100	100	4	75	1.0 ÷ 15.0	100
	HBSP8120	120	4 3/4	95	1.0 ÷ 15.0	100
	HBSP8140	140	5 1/2	110	1,0 ÷ 20,0	100
	HBSP8160	160	6 1/4	130	1,0 ÷ 20,0	100
10<br>0.40<br>TX 40	HBSP10100	100	4	75	1.0 ÷ 15.0	50
	HBSP10120	120	4 3/4	95	1.0 ÷ 15.0	50
	HBSP10140	140	5 1/2	110	1,0 ÷ 20,0	50
	HBSP10160	160	6 1/4	130	1,0 ÷ 20,0	50
	HBSP10180	180	7 1/8	150	1,0 ÷ 20,0	50

d_1 [mm] [in]	CODE	L [mm]	L [in]	b [mm]	A_P [mm]	pcs
12<br>0.48<br>TX 50	HBSP12120	120	4 3/4	90	1,0 ÷ 20,0	25
	HBSP12140	140	5 1/2	110	1,0 ÷ 20,0	25
	HBSP12160	160	6 1/4	120	1,0 ÷ 30,0	25
	HBSP12180	180	7 1/8	140	1,0 ÷ 30,0	25
	HBSP12200	200	8	160	1,0 ÷ 30,0	25

MINIMUM DISTANCES FOR SHEAR LOADS | STEEL-TO-TIMBER

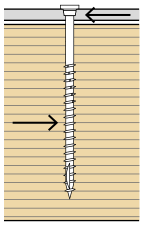

Load-to-grain angle α = 0°

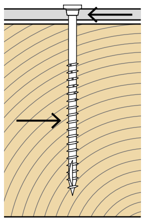

Load-to-grain angle α = 90°

		SCREWS INSERTED WITH PRE-DRILLING HOLE (α = 0°)				SCREWS INSERTED WITH PRE-DRILLING HOLE (α = 90°)			
d_1	[mm]		8	10	12		8	10	12
a_1	[mm]	5·d · 0,7	28	35	42	4·d · 0,7	22	28	34
a_2	[mm]	3·d · 0,7	17	21	25	4·d · 0,7	22	28	34
$a_{3,t}$	[mm]	12·d	96	120	144	7·d	56	70	84
$a_{3,c}$	[mm]	7·d	56	70	84	7·d	56	70	84
$a_{4,t}$	[mm]	3·d	24	30	36	7·d	56	70	84
$a_{4,c}$	[mm]	3·d	24	30	36	3·d	24	30	36

		SCREWS INSERTED WITHOUT PRE-DRILLING HOLE (α = 0°)				SCREWS INSERTED WITHOUT PRE-DRILLING HOLE (α = 90°)			
d_1	[mm]		8	10	12		8	10	12
a_1	[mm]	12·d · 0,7	67	84	101	5·d · 0,7	28	35	42
a_2	[mm]	5·d · 0,7	28	35	42	5·d · 0,7	28	35	42
$a_{3,t}$	[mm]	15·d	120	150	180	10·d	80	100	120
$a_{3,c}$	[mm]	10·d	80	100	120	10·d	80	100	120
$a_{4,t}$	[mm]	5·d	40	50	60	10·d	80	100	120
$a_{4,c}$	[mm]	5·d	40	50	60	5·d	40	50	60

d = nominal screw diameter

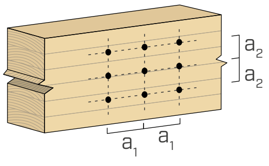

stressed end
-90° < α < 90°

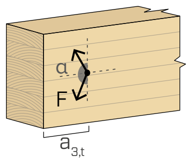

unloaded end
90° < α < 270°

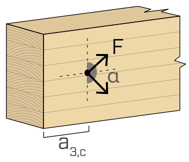

stressed edge
0° < α < 180°

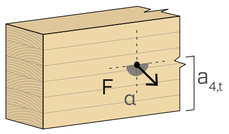

unload edge
180° < α < 360°

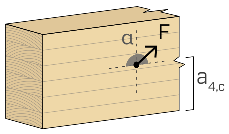

NOTES:

- The minimum distances are compliant with EN 1995:2014, according to ETA-11/0030, considering a timber characteristic density of $\rho_k \leq 420$ kg/m^3 and calculation diameter of d = nominal screw diameter.
- In the case of joints with elements in Douglas fir (Pseudotsuga menziesii), the minimum spacing and distances parallel to the grain must be multiplied by a coefficient of 1.5.

STATIC VALUES

CHARACTERISTIC VALUES
EN 1995:2014

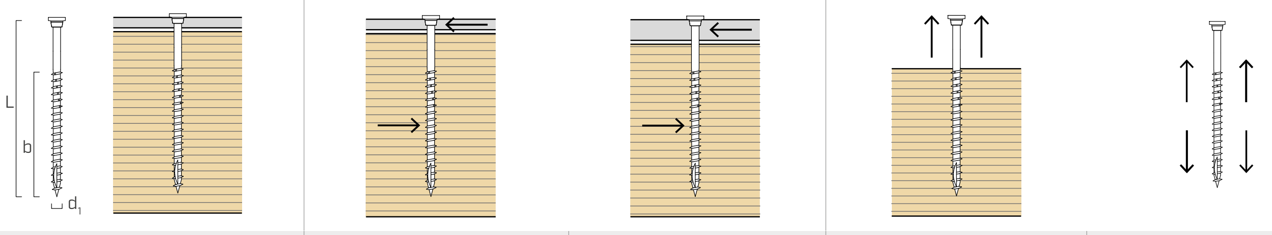

			SHEAR				TENSION	
geometry			thin steel-to-timber plate (1)		thick steel-to-timber plate (2)		thread withdrawal(3)	steel tension
d_1 [mm]	L [mm]	b [mm]		$R_{V,k}$ [kN]		$R_{V,k}$ [kN]	$R_{ax,k}$ [kN]	$R_{tens,k}$ [kN]
8	80	55	S_{PLATE} = 4,0 mm	4,07	S_{PLATE} = 8,0 mm	5,18	5,56	20,10
	100	75		4,58		5,69	7,58	
	120	95		5,08		6,19	9,60	
	140	110		5,36		6,57	11,11	
	160	130		5,36		7,08	13,13	
10	100	75	S_{PLATE} = 5,0 mm	6,01	S_{PLATE} = 10,0 mm	7,84	9,47	31,40
	120	95		6,87		8,47	12,00	
	140	110		7,34		8,95	13,89	
	160	130		7,74		9,58	16,42	
	180	150		7,74		10,21	18,94	
12	120	90	S_{PLATE} = 6,0 mm	8,19	S_{PLATE} = 12,0 mm	10,17	13,64	33,90
	140	110		8,94		10,92	16,67	
	160	120		9,32		11,30	18,18	
	180	140		9,55		12,06	21,21	
	200	160		9,55		12,82	24,24	

NOTES:

(1) The shear resistance characteristics are calculated considering the case of a thin plate ($S_{PLATE} \leq 0,5\ d_1$).

(2) The shear resistance characteristics are calculated considering the case of a thick plate ($S_{PLATE} \geq d_1$).

(3) The axial thread withdrawal resistance was calculated considering a 90° angle between the grain and the connector and for a fixing length of b.

In the case of steel-to-timber connections, generally the steel tensile strength is binding with respect to head separation or pull-through.

GENERAL PRINCIPLES:

- Characteristic values comply with the EN 1995:2014 standard in accordance with ETA-11/0030.
- Design values can be obtained from characteristic values as follows:

$$R_d = \frac{R_k \cdot k_{mod}}{\gamma_M}$$

The coefficients γ_M and k_{mod} should be taken according to the current regulations used for the calculation.

- The tensile design strength of the connector is the lower between the timber-side design strength ($R_{ax,d}$) and the steel-side design strength ($R_{tens,d}$).

$$R_{ax,d} = min \begin{cases} \dfrac{R_{ax,k} \cdot k_{mod}}{\gamma_M} \\ \dfrac{R_{tens,k}}{\gamma_{M2}} \end{cases}$$

- For the mechanical resistance values and the geometry of the screws, reference was made to ETA-11/0030.
- For the calculation process a timber characteristic density ρ_k = 385 kg/m^3 has been considered.
- Values were calculated considering the threaded part as being completely inserted into the wood.
- Sizing and verification of the timber elements, panels and steel plates must be done separately.
- The characteristic shear resistances are calculated for screws inserted without pre-drilling hole. In the case of screws inserted with pre-drilling hole, greater resistance values can be obtained.
- For different calculation methods, the MyProject software is available free of charge (www.rothoblaas.com).

VGS

AC233
ESR-4645

BIT INCLUDED

SOFTWARE

ETA-11/0030

FULLY THREADED SCREW WITH COUNTERSUNK OR HEXAGONAL HEAD

TENSION

Deep thread and high resistance steel ($f_{y,k}$ = 1000 N/mm²) for excellent tensile performance. Approved for structural applications subject to stresses in any direction vs. the grain (α = 0° - 90°).

COUNTERSUNK OR HEXAGONAL HEAD

Countersunk head up to L = 600 mm, ideal for use on plates or for concealed reinforcements. Hexagonal head L > 600 mm to facilitate gripping with screwdriver.

CHROMIUM (VI) FREE

Total absence of hexavalent chromium. Compliance with the strictest regulations governing chemical substances (SVHC).
REACH information available.

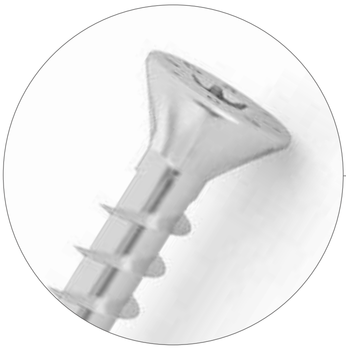

9,0 | 11,0 | 13,0 mm L ≤ 600 mm

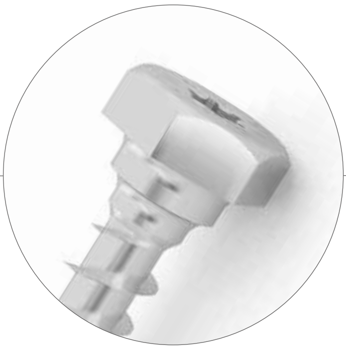

11,0 | 13,0 mm L > 600 mm

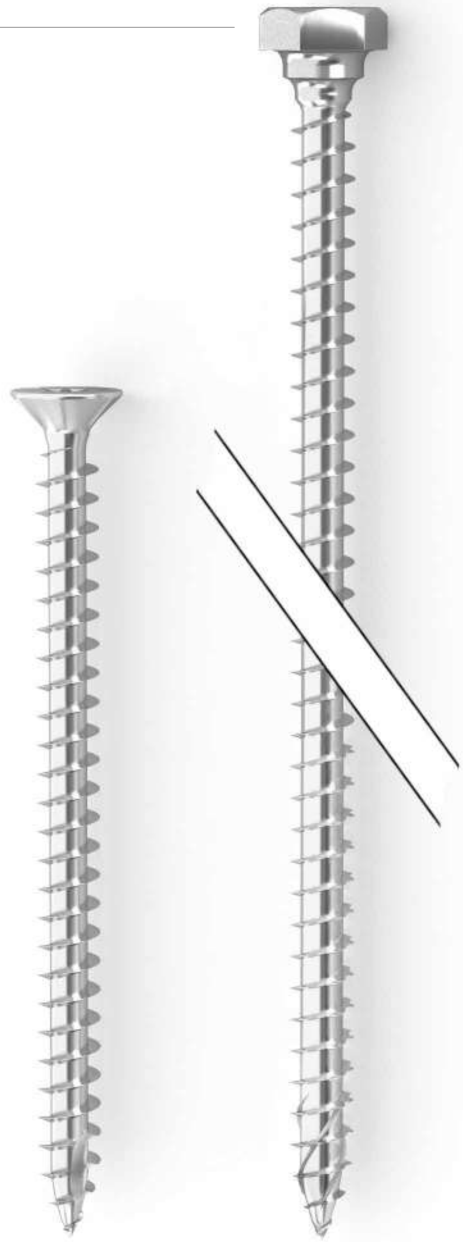

CHARACTERISTICS

**FOCUS**	45° connections, lifting and reinforcements
**HEAD**	countersunk with ribs for L ≤ 600 mm<br>hexagonal for L > 600 mm
**DIAMETER**	9,0 | 11,0 | 13,0 mm
**LENGTH**	from 100 to 1200 mm

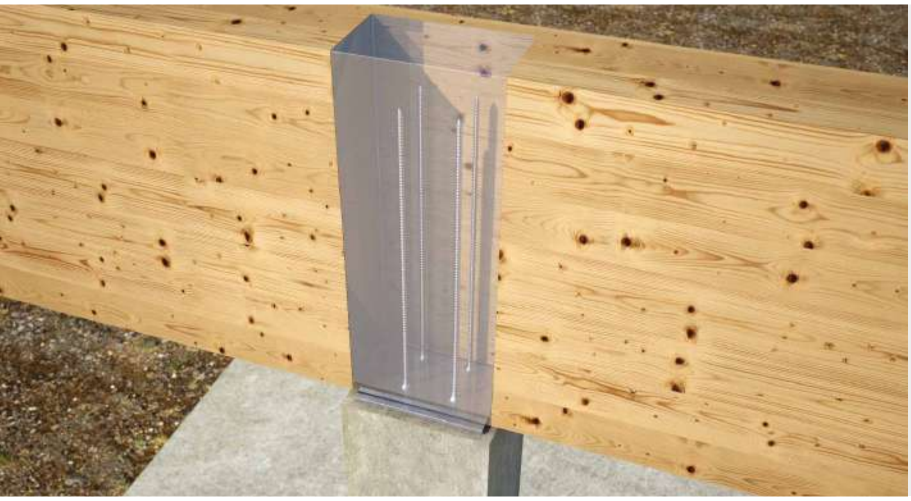

MATERIAL

Galvanized carbon steel.

FIELDS OF USE

- timber based panels
- solid timber
- glulam (Glued Laminated Timber)
- CLT, LVL
- high density woods

Service classes 1 and 2.

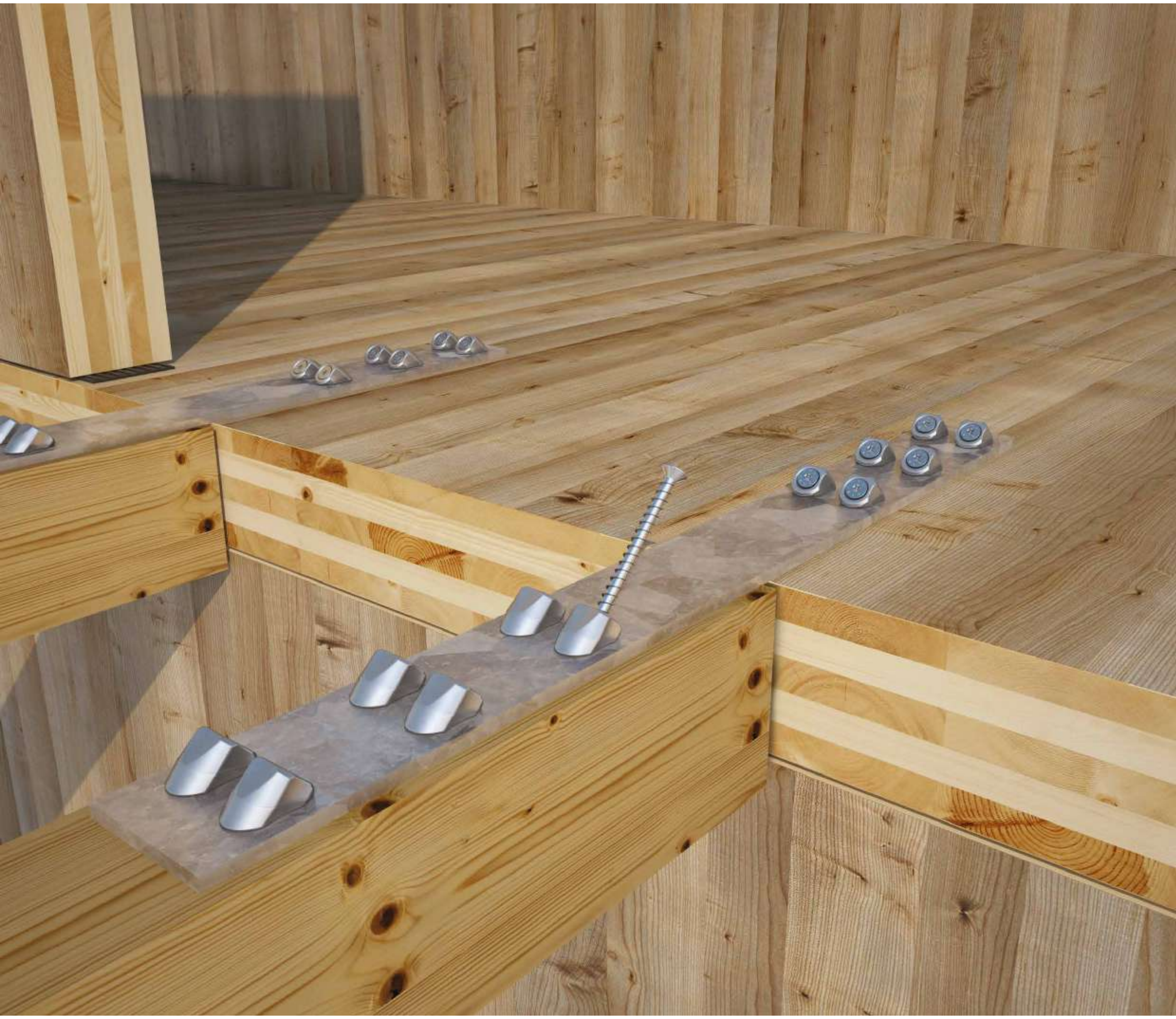

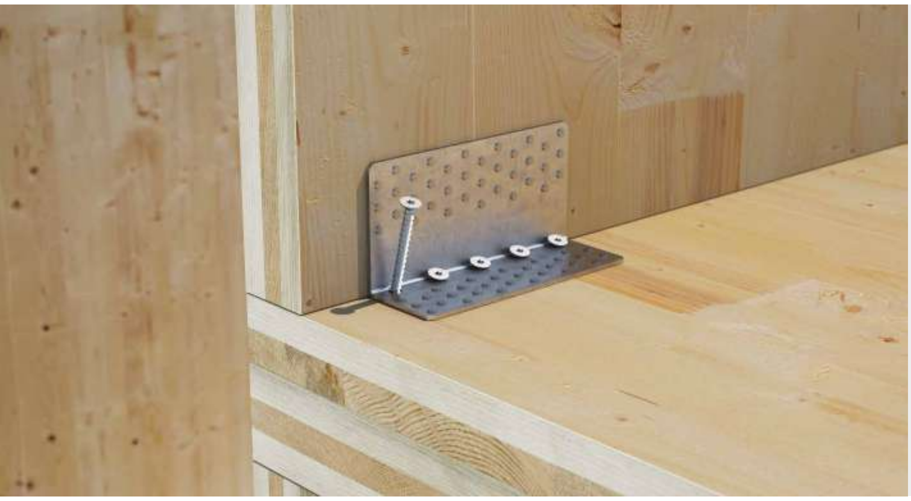

TENSILE STRENGTH

Ideal for joints requiring high tensile or sliding strength. Can be used on steel plates in combination with the VGU washer.

TITAN V

Values also tested, certified and calculated for fastening standard Rothoblaas plates.

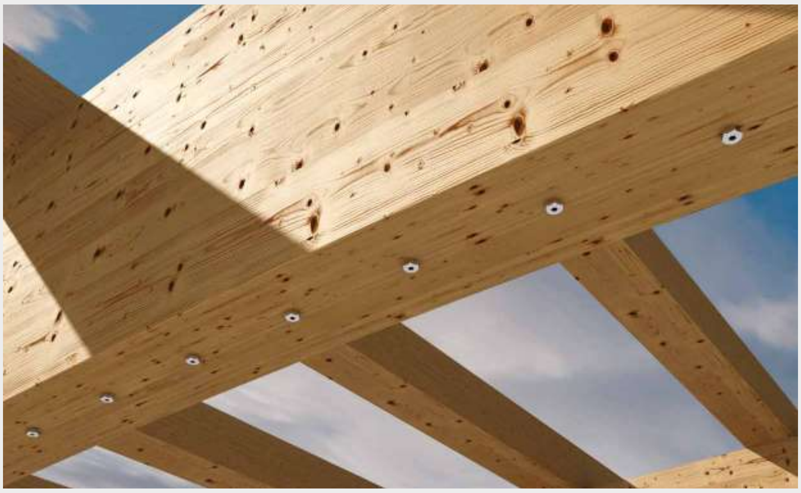

Reinforcement perpendicular to the grain of a large glulam beam.

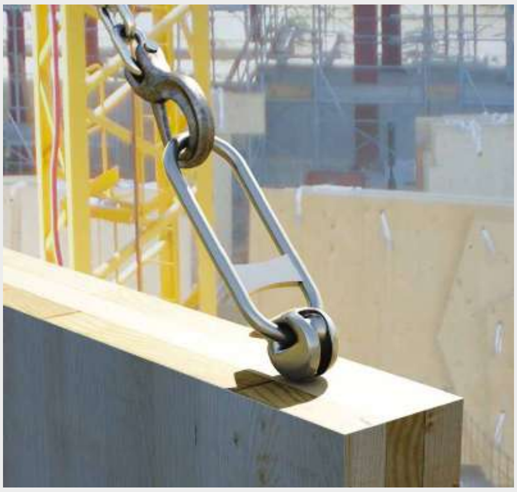

System for lifting and transport using WASP hook and VGS screw.

GEOMETRY AND MECHANICAL CHARACTERISTICS

VGS Ø9 - Ø11

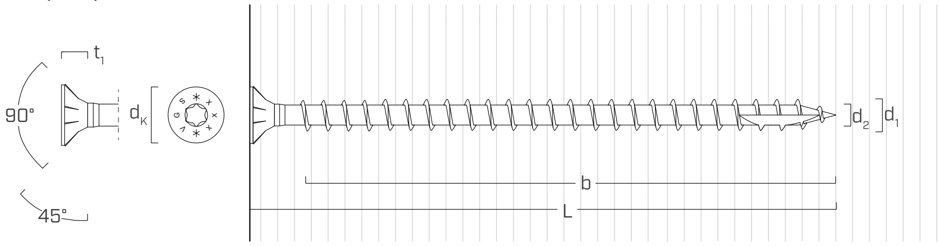

VGS Ø11 | L > 600 mm

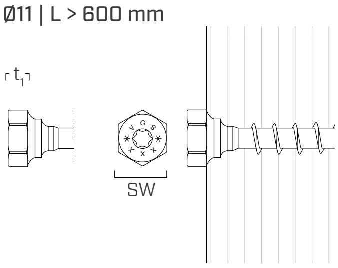

VGS Ø13 | L ≤ 600 mm

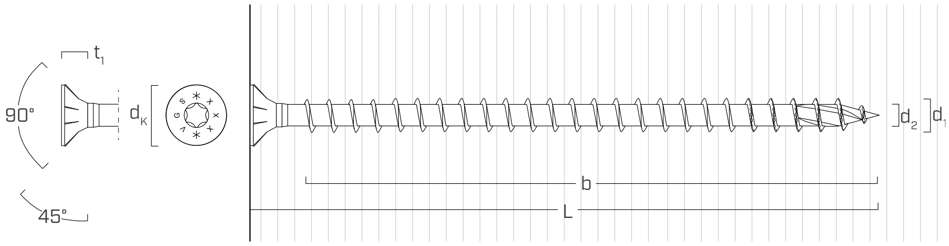

VGS Ø13 | L > 600 mm

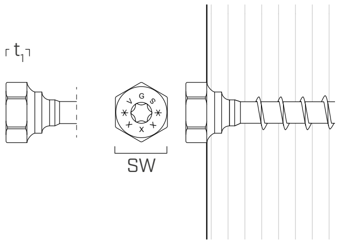

**Nominal diameter**	**d_1**	**[mm]**	**9**	**11** [L ≤ 600 mm]	**11** [L > 600 mm]	**13** [L ≤ 600 mm]	**13** [L > 600 mm]
Head diameter	d_K	[mm]	16,00	19,30	-	22,00	-
Wrench size	SW		-	-	SW17	-	SW19
Head thickness	t_1	[mm]	6,50	8,20	6,40	9,40	7,50
Tip diameter	d_2	[mm]	5,90	6,60		8,00	
Pre-drilling hole diameter(1)	d_V	[mm]	5,0	6,0		8,0	
Characteristic yield moment	$M_{y,k}$	[Nm]	27,2	45,9		70,9	
Characteristic withdrawal-resistance parameter(2)	$f_{ax,k}$	[N/mm²]	11,7	11,7		11,7	
Associated density	ρ_a	[kg/m³]	350	350		350,0	
Characteristic tensile strength	$f_{tens,k}$	[kN]	25,4	38,0		53,0	
Characteristic yield strength	$f_{y,k}$	[N/mm²]	1000	1000		1000	

(1) Pre-drilling valid for softwood.
(2) Valid for softwood - maximum density 440 kg/m³.
For applications with different materials or with high density please see ETA-11/0030.

For VGS Ø13 screw a Ø8x80 predrill is recommended.

CODES AND DIMENSIONS

d_1 [mm] *[in]*	CODE	L [mm]	L *[in]*	b [mm]	pcs
9 *0.36* TX 40	VGS9100	100	*4*	90	25
	VGS9120	120	*4 3/4*	110	25
	VGS9140	140	*5 1/2*	130	25
	VGS9160	160	*6 1/4*	150	25
	VGS9180	180	*7 1/8*	170	25
	VGS9200	200	*8*	190	25
	VGS9220	220	*8 5/8*	210	25
	VGS9240	240	*9 1/2*	230	25
	VGS9260	260	*10 1/4*	250	25
	VGS9280	280	*11*	270	25
	VGS9300	300	*11 3/4*	290	25
	VGS9320	320	*12 5/8*	310	25
	VGS9340	340	*13 3/8*	330	25
	VGS9360	360	*14 1/4*	350	25
	VGS9380	380	*15*	370	25
	VGS9400	400	*15 3/4*	390	25
	VGS9440	440	*17 1/4*	430	25
	VGS9480	480	*19*	470	25
	VGS9520	520	*20 1/2*	510	25
11 *0.44* TX 50	VGS11100	100	*4*	90	25
	VGS11125	125	*4 15/16*	115	25
	VGS11150	150	*6*	140	25
	VGS11175	175	*6 7/8*	165	25
	VGS11200	200	*8*	190	25
	VGS11225	225	*8 7/8*	215	25
	VGS11250	250	*10*	240	25
	VGS11275	275	*10 7/8*	265	25
	VGS11300	300	*11 3/4*	290	25
	VGS11325	325	*12 3/4*	315	25
	VGS11350	350	*13 3/4*	340	25
	VGS11375	375	*14 3/4*	365	25
	VGS11400	400	*15 3/4*	390	25
	VGS11450	450	*17 3/4*	440	25
	VGS11500	500	*19 3/4*	490	25
	VGS11550	550	*21 5/8*	540	25
	VGS11600	600	*23 5/8*	590	25
11 *0.44* SW17 TX 50	VGS11700	700	*27 1/2*	680	25
	VGS11800	800	*31 1/2*	780	25

d_1 [mm] *[in]*	CODE	L [mm]	L *[in]*	b [mm]	pcs
13 *0.52* TX 50	VGS13100	100	*4*	90	25
	VGS13150	150	*6*	140	25
	VGS13200	200	*8*	190	25
	VGS13300	300	*11 3/4*	280	25
	VGS13400	400	*15 3/4*	380	25
	VGS13500	500	*19 3/4*	480	25
	VGS13600	600	*23 5/8*	580	25
13 *0.52* SW 19 TX 50	VGS13700	700	*27 1/2*	680	25
	VGS13800	800	*31 1/2*	780	25
	VGS13900	900	*35 1/2*	880	25
	VGS131000	1000	*39 3/8*	980	25
	VGS131100	1100	*43 5/16*	1080	25
	VGS131200	1200	*47 1/4*	1180	25

VGU WASHER

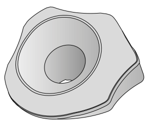

CODE	screw [mm]	pcs
VGU945	VGS Ø9	25
VGU1145	VGS Ø11	25
VGU1345	VGS Ø13	25

WASP HOOK

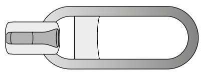

CODE	screw [mm]	max. capacity [kg]	pcs
WASP	VGS Ø11	1300	2
WASPL	VGS Ø13	5000	2

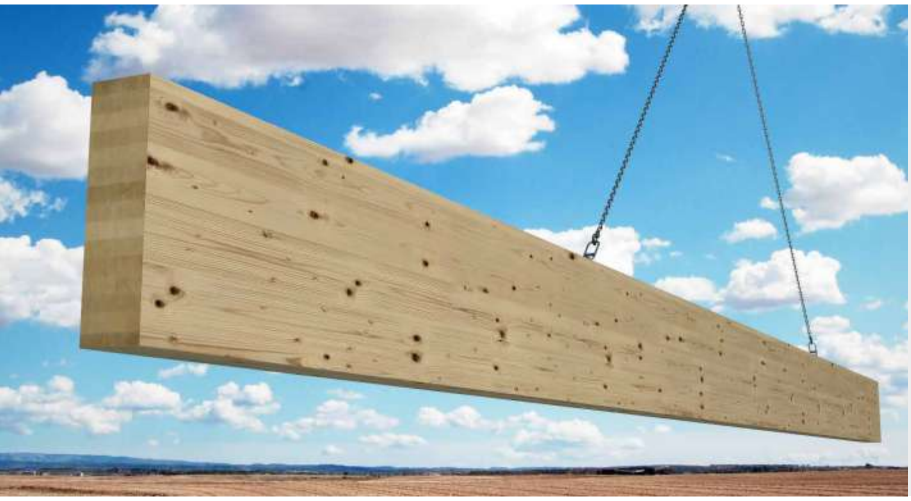

WASP

Various installation options with more types of screws for load conditions and different material.

EFFECTIVE THREAD USED IN CALCULATION

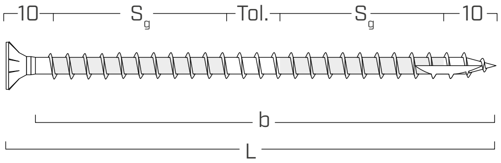

b = L - 10 mm — represents the entire length of the threaded part

S_g = (L - 10 mm - 10 mm - Tol.)/ 2 — represents the partial length of the threaded part net of a laying tolerance (Tol.) of 10 mm

The timber to timber withdrawal, shear and sliding values were calculated considering the centre of gravity of the connector placed in correspondence with the shear plane.

MINIMUM DISTANCES FOR SHEAR LOADS [1]

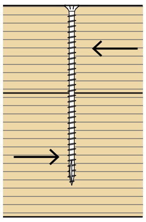

Load-to-grain angle α = 0°

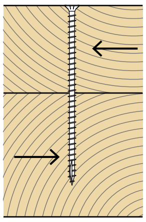

Load-to-grain angle α = 90°

		α = 0°: SCREWS INSERTED WITH PRE-DRILLING HOLE				α = 90°: SCREWS INSERTED WITH PRE-DRILLING HOLE			
d_1	[mm]		9	11	13		9	11	13
a_1	[mm]	5·d	45	55	65	4·d	36	44	52
a_2	[mm]	3·d	27	33	39	4·d	36	44	52
$a_{3,t}$	[mm]	12·d	108	132	156	7·d	63	77	91
$a_{3,c}$	[mm]	7·d	63	77	91	7·d	63	77	91
$a_{4,t}$	[mm]	3·d	27	33	39	7·d	63	77	91
$a_{4,c}$	[mm]	3·d	27	33	39	3·d	27	33	39

		α = 0°: SCREWS INSERTED WITHOUT PRE-DRILLING HOLE				α = 90°: SCREWS INSERTED WITHOUT PRE-DRILLING HOLE			
d_1	[mm]		9	11	13		9	11	13
a_1	[mm]	12·d	108	132	156	5·d	45	55	65
a_2	[mm]	5·d	45	55	65	5·d	45	55	65
$a_{3,t}$	[mm]	15·d	135	165	195	10·d	90	110	130
$a_{3,c}$	[mm]	10·d	90	110	130	10·d	90	110	130
$a_{4,t}$	[mm]	5·d	45	55	65	10·d	90	110	130
$a_{4,c}$	[mm]	5·d	45	55	65	5·d	45	55	65

d = nominal screw diameter

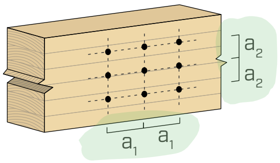

stressed end
-90° < α < 90°

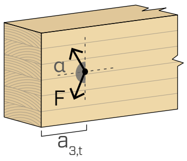

unloaded end
90° < α < 270°

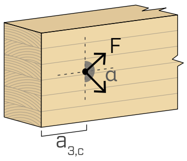

stressed edge
0° < α < 180°

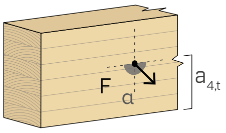

unload edge
180° < α < 360°

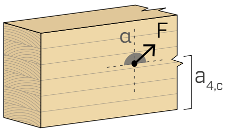

NOTES:

(1) Minimum distances are in accordance with EN 1995:2014 as per ETA-11/0030 considering a timber characteristic density of $\rho_k \leq 420$ kg/m^3.

- The minimum spacing for all steel-to-timber connections (a_1, a_2) can be multiplied by a coefficient of 0,7.
- The minimum spacing for all panel-to-timber connections(a_1, a_2) can be multiplied by a coefficient of 0,85.

MINIMUM DISTANCES FOR AXIAL STRESSES [2]

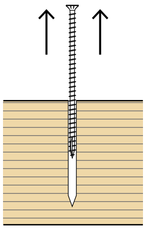

			SCREWS INSERTED WITH AND WITHOUT PRE-DRILLING HOLE		
**d_1**	**[mm]**		**9**	**11**	**13**
**a_1**	[mm]	**5·d**	45	55	65
**a_2**	[mm]	**5·d**	45	55	65
**$a_{2,LIM}$** [3]	[mm]	**2.5·d**	23	28	33
**$a_{1,CG}$**	[mm]	**10·d**	90	110	130
**$a_{2,CG}$**	[mm]	**4·d**	36	44	52
**a_{CROSS}**	[mm]	**1.5·d**	14	17	20

d = nominal screw diameter

SCREWS UNDER TENSION INSERTED WITH AN ANGLE α WITH RESPECT TO THE GRAIN

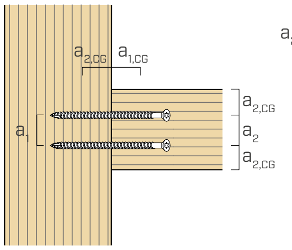

plan

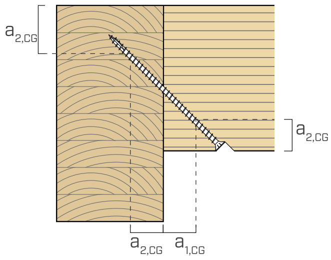

front

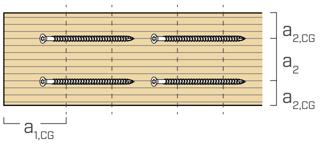

plan

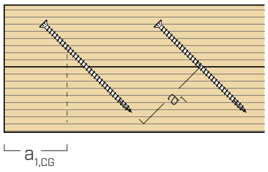

front

SCREWS INSERTED WITH α = 90° ANGLE WITH RESPECT TO THE GRAIN

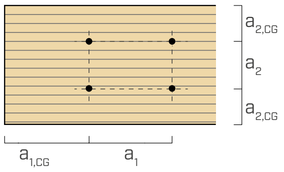

plan

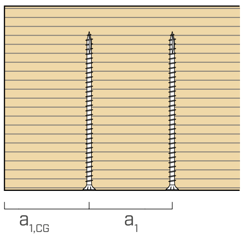

front

CROSS SCREWS INSERTED WITH AN ANGLE α WITH RESPECT TO THE GRAIN

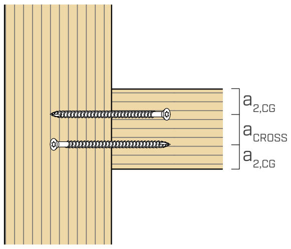

plan

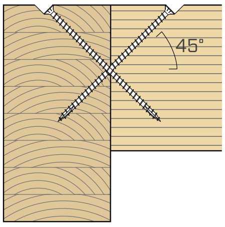

front

NOTES:

[2] The minimum distances for connectors stressed axially are independent of the insertion angle of the connector and the angle of the force with respect to the grain, in accordance with ETA-11/0030.

[3] The axial distance a_2 can be reduced down to 2,5 d_1 if for each connector a "joint surface" $a_1 \cdot a_2 = 25 \cdot d_1^2$ is maintained.

STATIC VALUES

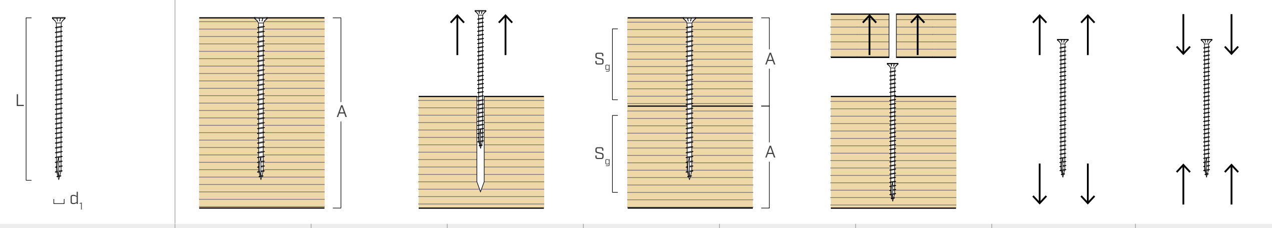

		TENSION[1]/COMPRESSION[2]							
geometry		total thread withdrawal[3]			partial thread withdrawal[3]			steel tension	instability
				timber			timber	steel	steel
d_1 [mm]	L [mm]	b [mm]	A_{min} [mm]	$R_{ax,k}$ [kN]	S_g [mm]	A_{min} [mm]	$R_{ax,k}$ [kN]	$R_{tens,k}$ [kN]	$R_{ki,k}$ [kN]
	100	90	110	10,23	35	55	3,98		
	120	110	130	12,50	45	65	5,11		
	140	130	150	14,77	55	75	6,25		
	160	150	170	17,05	65	85	7,39		
	180	170	190	19,32	75	95	8,52		
	200	190	210	21,59	85	105	9,66		
	220	210	230	23,87	95	115	10,80		
	240	230	250	26,14	105	125	11,93		
	260	250	270	28,41	115	135	13,07		
9	280	270	290	30,68	125	145	14,21	25,40	17,25
	300	290	310	32,96	135	155	15,34		
	320	310	330	35,23	145	165	16,48		
	340	330	350	37,50	155	175	17,61		
	360	350	370	39,78	165	185	18,75		
	380	370	390	42,05	175	195	19,89		
	400	390	410	44,32	185	205	21,02		
	440	430	450	48,87	205	225	23,30		
	480	470	490	53,41	225	245	25,57		
	520	510	530	57,96	245	265	27,84		
	100	90	110	12,50	35	55	4,86		
	125	115	135	15,97	48	68	6,60		
	150	140	160	19,45	60	80	8,33		
	175	165	185	22,92	73	93	10,07		
	200	190	210	26,39	85	105	11,81		
	225	215	235	29,86	98	118	13,54		
	250	240	260	33,34	110	130	15,28		
	275	265	285	36,81	123	143	17,01		
	300	290	310	40,28	135	155	18,75		
11	325	315	335	43,75	148	168	20,49	38,00	21,93
	350	340	360	47,22	160	180	22,22		
	375	365	385	50,70	173	193	23,96		
	400	390	410	54,17	185	205	25,70		
	450	440	460	61,11	210	230	29,17		
	500	490	510	68,06	235	255	32,64		
	550	540	560	75,00	260	280	36,11		
	600	590	610	81,95	285	305	39,59		
	700	680	710	94,45	335	355	46,53		
	800	780	810	108,34	385	405	53,48		

STATIC VALUES

CHARACTERISTIC VALUES
EN 1995:2014

TENSION[1]/COMPRESSION[2]

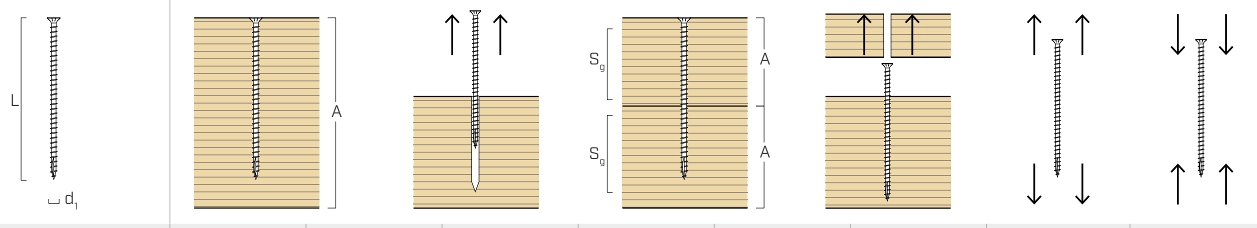

geometry		total thread withdrawal[3]			partial thread withdrawal[3]			steel tension	instability
				timber			timber	steel	steel
d_1 [mm]	L [mm]	b [mm]	A_{min} [mm]	$R_{ax,k}$ [kN]	S_g [mm]	A_{min} [mm]	$R_{ax,k}$ [kN]	$R_{tens,k}$ [kN]	$R_{ki,k}$ [kN]
	100	90	110	14,77	35	55	5,75		
	150	140	160	22,98	60	80	9,85		
	200	190	210	31,19	85	105	13,95		
	300	280	310	45,96	135	155	22,16		
	400	380	410	62,38	185	205	30,37		
	500	480	510	78,79	235	255	38,58		
**13**	600	580	610	95,21	285	305	46,78	53,00	32,69
	700	680	710	111,62	335	355	54,99		
	800	780	810	128,04	385	405	63,20		
	900	880	910	144,45	435	455	71,41		
	1000	980	1010	160,87	485	505	79,61		
	1100	1080	1110	177,28	535	555	87,82		
	1200	1180	1210	193,70	585	605	96,03		

NOTES:

(1) The tensile design strength of the connector is the lower between the timber-side design strength ($R_{ax,d}$) and the steel-side design strength ($R_{tens,d}$).

$$R_{ax,d} = min \begin{cases} \dfrac{R_{ax,k} \cdot k_{mod}}{\gamma_M} \\ \dfrac{R_{tens,k}}{\gamma_{M2}} \end{cases}$$

(2) The compression design strength of the connector is the lower between the timber-side design strength ($R_{ax,d}$) and the instability design strength ($R_{ki,k}$).

$$R_{ax,d} = min \begin{cases} \dfrac{R_{ax,k} \cdot k_{mod}}{\gamma_M} \\ \dfrac{R_{ki,k}}{\gamma_{M1}} \end{cases}$$

(3) The axial resistance of the thread to withdrawal was calculated considering a 90° angle between the fibres and the connector and for a effective thread length of b or S_g.
For intermediate values of S_g it is possible to linearly interpolate.

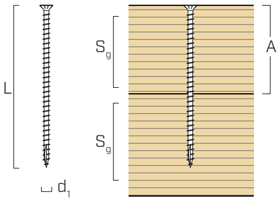

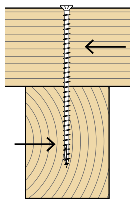

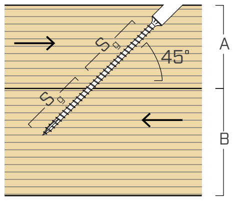

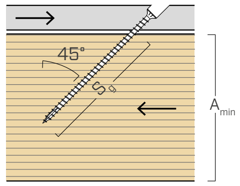

			SHEAR		SLIDING[4]						
geometry			timber-to-timber		timber-to-timber[5]			steel-to-timber[5]			
d_1 [mm]	L [mm]	S_g [mm]	A_{min} [mm]	$R_{V,k}$ [kN]	A_{min} [mm]	B_{min} [mm]	timber $R_{V,k}$ [kN]	S_g [mm]	A_{min} [mm]	timber $R_{V,k}$ [kN]	steel $R_{tens,k\ 45°}$[6] [kN]
9	100	35	50	3,53	40	55	2,81	80	75	6,43	17,96
	120	45	60	4,19	50	60	3,62	100	90	8,04	
	140	55	70	4,81	55	70	4,42	120	105	9,64	
	160	65	80	5,10	60	75	5,22	140	120	11,25	
	180	75	90	5,38	70	85	6,03	160	135	12,86	
	200	85	100	5,67	75	90	6,83	180	145	14,46	
	220	95	110	5,95	85	100	7,63	200	160	16,07	
	240	105	120	6,23	90	105	8,44	220	175	17,68	
	260	115	130	6,50	100	110	9,24	240	190	19,29	
	280	125	140	6,50	105	120	10,04	260	205	20,89	
	300	135	150	6,50	110	125	10,85	280	220	22,50	
	320	145	160	6,50	120	135	11,65	300	230	24,11	
	340	155	170	6,50	125	140	12,46	320	245	25,71	
	360	165	180	6,50	135	145	13,26	340	260	27,32	
	380	175	190	6,50	140	155	14,06	360	275	28,93	
	400	185	200	6,50	145	160	14,87	380	290	30,54	
	440	205	220	6,50	160	175	16,47	420	315	33,75	
	480	225	240	6,50	175	190	18,08	460	345	36,96	
	520	245	260	6,50	190	205	19,69	500	375	40,18	
11	100	35	50	4,27	40	55	3,44	80	75	7,86	26,87
	125	48	63	5,40	50	65	4,67	105	95	10,31	
	150	60	75	6,40	60	75	5,89	130	110	12,77	
	175	73	88	7,05	70	80	7,12	155	130	15,22	
	200	85	100	7,48	80	90	8,35	180	145	17,68	
	225	98	113	7,92	85	100	9,58	205	165	20,13	
	250	110	125	8,35	95	110	10,80	230	185	22,59	
	275	123	138	8,79	105	115	12,03	255	200	25,04	
	300	135	150	9,06	115	125	13,26	280	220	27,50	
	325	148	163	9,06	120	135	14,49	305	235	29,96	
	350	160	175	9,06	130	145	15,71	330	255	32,41	
	375	173	188	9,06	140	155	16,94	355	270	34,87	
	400	185	200	9,06	150	160	18,17	380	290	37,32	
	450	210	225	9,06	165	180	20,63	430	325	42,23	
	500	235	250	9,06	185	195	23,08	480	360	47,14	
	550	260	275	9,06	200	215	25,54	530	395	52,05	
	600	285	300	9,06	220	230	27,99	580	430	56,96	
	700	335	350	9,06	255	265	32,90	-	-	-	
	800	385	400	9,06	290	305	37,81	-	-	-	

STATIC VALUES

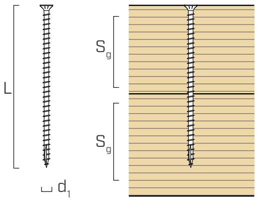

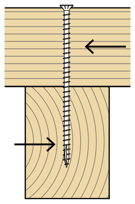

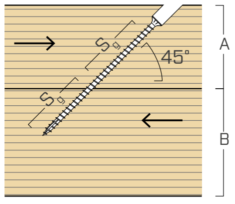

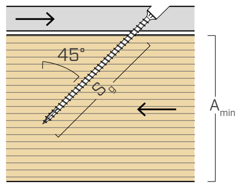

geometry			SHEAR timber-to-timber		SLIDING[4] timber-to-timber[5]			SLIDING[4] steel-to-timber[5]			
							timber			timber	steel
d_1 [mm]	L [mm]	S_g [mm]	A_{min} [mm]	$R_{V,k}$ [kN]	A_{min} [mm]	B_{min} [mm]	$R_{V,k}$ [kN]	S_g [mm]	A_{min} [mm]	$R_{V,k}$ [kN]	$R_{tens,k\ 45°}$[6] [kN]
	100	35	50	4,87	45	55	4,06	80	75	9,29	
	150	60	75	7,61	60	75	6,96	130	110	15,09	
	200	85	100	9,46	80	90	9,87	180	145	20,89	
	300	135	150	11,51	115	125	15,67	280	220	32,50	
	400	185	200	11,94	150	160	21,47	380	290	44,11	
	500	235	250	11,94	185	195	27,28	480	360	55,71	
**13**	600	285	300	11,94	220	230	33,08	580	430	67,32	37,48
	700	335	350	11,94	255	265	38,88	-	-	-	
	800	385	400	11,94	290	305	44,69	-	-	-	
	900	435	450	11,94	325	340	50,49	-	-	-	
	1000	485	500	11,94	360	375	56,30	-	-	-	
	1100	535	550	11,94	395	410	62,10	-	-	-	
	1200	585	600	11,94	430	445	67,90	-	-	-	

NOTES:

(4) The axial thread withdrawal resistance was calculated considering a 45° angle between the fibres and the connector and for an effective thread length of S_g.

(5) The design sliding resistance of the connector is either the timber-side design resistance ($R_{V,d}$) and the steel design resistance ($R_{tens,d\ 45°}$), whichever is lower.

$$R_{V,d} = min \begin{cases} \frac{R_{V,k} \cdot k_{mod}}{\gamma_M} \\ \frac{R_{tens,k\ 45°}}{\gamma_{M2}} \end{cases}$$

To properly create the joint, the head of the connector must be completely inserted in the steel plate.

(6) The connector tensile strength was calculated considering a 45° angle between the fibres and the connector.

GENERAL PRINCIPLES:

- Characteristic values comply with the EN 1995:2014 standard in accordance with ETA-11/0030.
- Design values can be obtained from characteristic values as follows:

$$R_d = \frac{R_k \cdot k_{mod}}{\gamma_M}$$

The coefficients γ_M and k_{mod} should be taken according to the current regulations used for the calculation.

- For the mechanical resistance values and the geometry of the screws, reference was made to ETA-11/0030.
- For the calculation process a timber characteristic density ρ_k = 385 kg/m^3 has been considered.
- Dimensioning and verification of timber elements and steel plates must be carried out separately.
- The characteristic shear resistances are calculated for screws inserted without pre-drilling hole. In the case of screws inserted with pre-drilling hole, greater resistance values can be obtained.
- The timber to timber withdrawal, shear and sliding values were calculated considering the centre of gravity of the connector placed in correspondence with the shear plane.

TIMBER-TO-TIMBER APPLICATION

RECOMMENDED INSERTION MOMENT: M_{ins}

VGS Ø9	M_{ins} = 20 Nm
VGS Ø11 L < 400 mm	M_{ins} = 30 Nm
VGS Ø11 L ≥ 400 mm	M_{ins} = 40 Nm
VGS Ø13	M_{ins} = 50 Nm

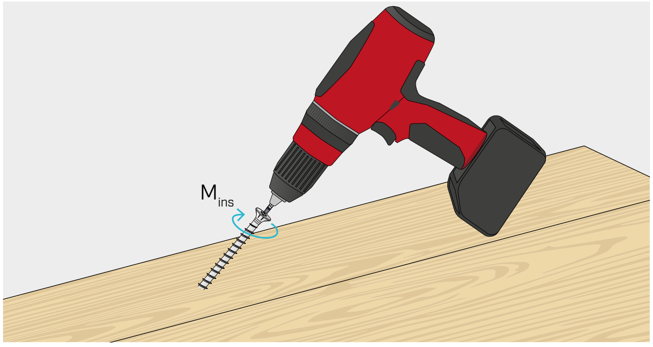

STEEL-TO-TIMBER APPLICATION

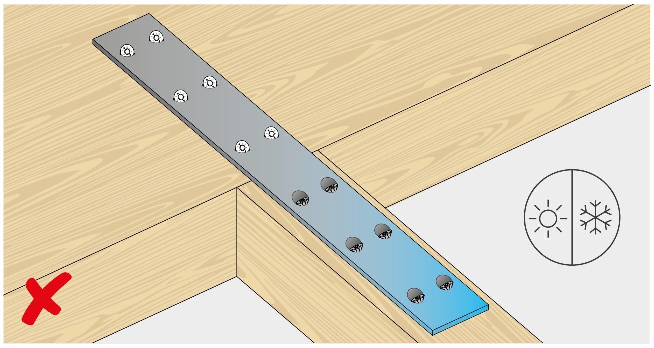

Avoid dimensional changes to the metal.

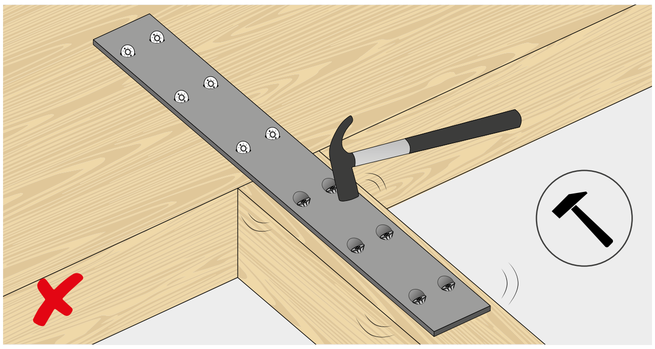

Avoid accidental stress during installation.

A. SHAPED PLATE WITH COUNTERSUNK HOLES

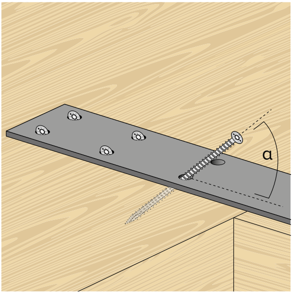

Respect the insertion angle (e.g. adopting a template).

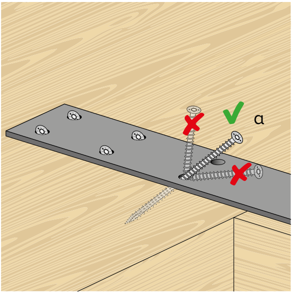

Avoid bending.

B. VGU WASHER

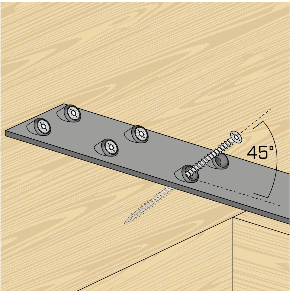

Respect the 45° insertion angle.

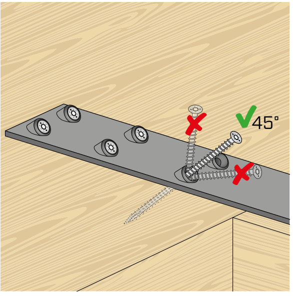

Avoid bending.

A. SHAPED PLATE

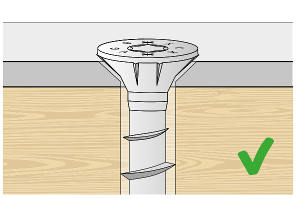

Countersunk hole.

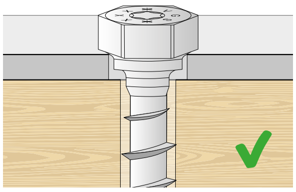

Cylindrical hole.

B. WASHERS

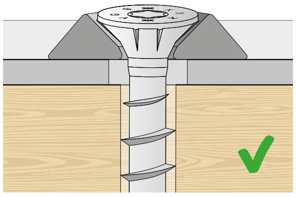

Flared washer.

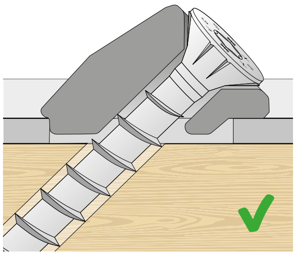

VGU washer.

APPLICATION EXAMPLES

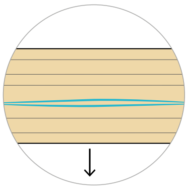

TAPERED BEAMS
apex tension reinforcement perpendicular to grain

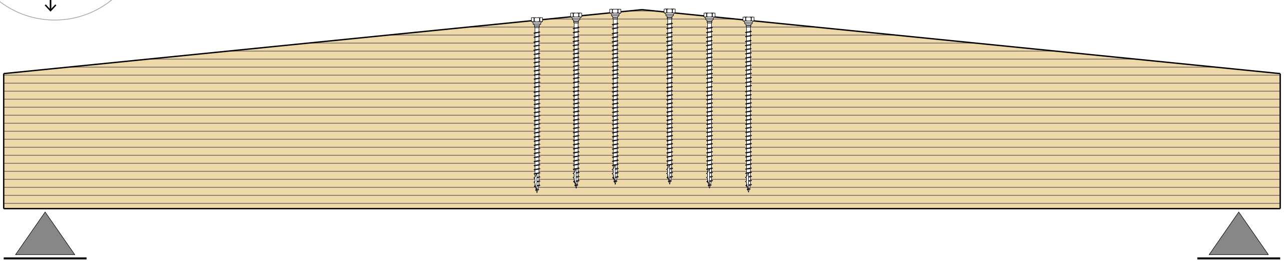

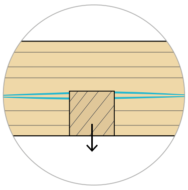

HANGING LOAD
tension reinforcement perpendicular to grain

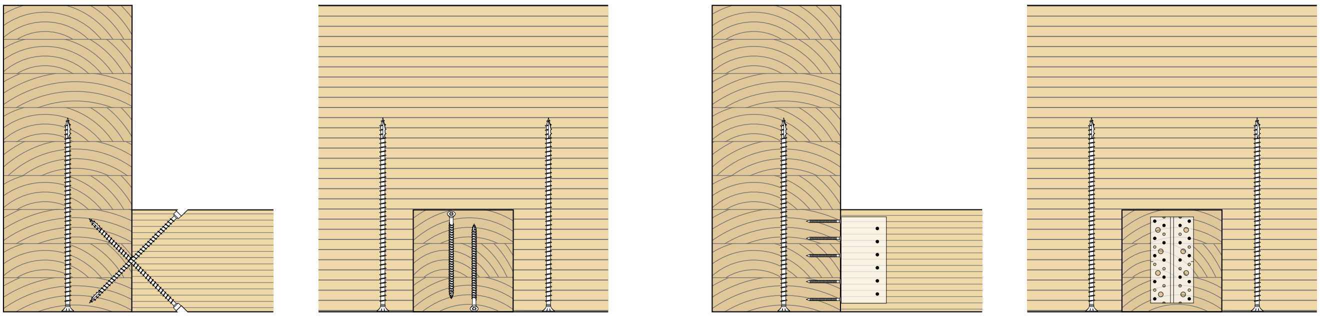

section   front   section   front

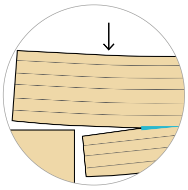

NOTCH
tension reinforcement perpendicular to grain

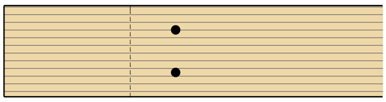

plan

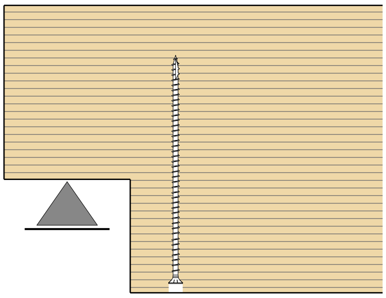

section

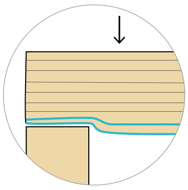

SUPPORT
compression reinforcement perpendicular to grain

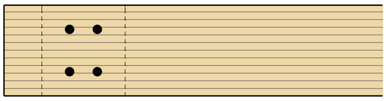

plan

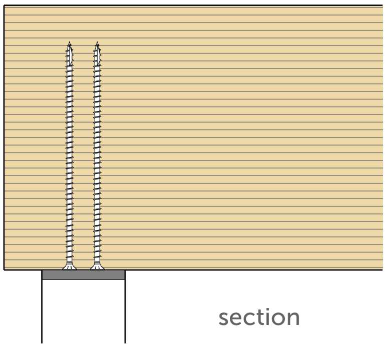

section

CALCULATION EXAMPLES: REINFORCEMENT OF BEAM WITH COMPRESSION PERPENDICULAR TO THE FIBRES

MY PROJECT SOFTWARE

PROJECT DATA	
B = 220 mm	$F_{v,Rd}$ = 158 kN
H = 560 mm	$F_{c,90,Rd}$ = 158 kN
a = 25 mm	Service class = 1
L_a = 200 mm	Load duration = medium
GL24h Glulam (ρ_k = 385 kg/m^3)	

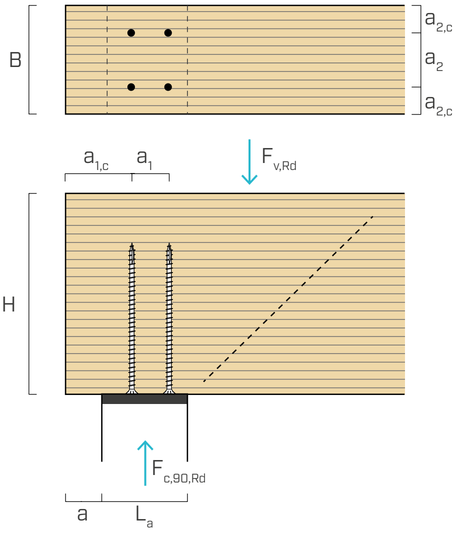

SHEAR VERIFICATION OF SUPPORT (EN 1995:2014) : $\tau_d \leq f_{v,d}$

$$\tau_d = \frac{1{,}5 \cdot F_{v,Rd}}{B \cdot H}$$

τ_d = 1,92 N/mm^2
$f_{v,k}$ = 3,50 N/mm^2

EN 1995:2014
k_{mod} = 0,8
γ_M = 1,25
$f_{v,d}$ = 2,24 N/mm^2

$\tau_d \leq f_{v,d}$ 1,92 < 2,24 N/mm^2
verification passed

Italy - NTC 2018
k_{mod} = 0,8
γ_M = 1,45
$f_{v,d}$ = 1,93 N/mm^2

$\tau_d \leq f_{v,d}$ 1,92 < 1,93 N/mm^2
verification passed

PERPENDICULAR COMPRESSION VERIFICATION OF SUPPORT-BEAM WITHOUT REINFORCEMENT (EN 1995:2014) : $\sigma_{c,90,d} \leq k_{c,90} \cdot f_{c,90,d}$

$$l_{ef,1} = L_a + a + 30$$

$$\sigma_{c,90,d} = \frac{F_{v,Rd}}{B \cdot l_{ef,1}}$$

$l_{ef,1}$ = 255 mm
$\sigma_{c,90,d}$ = 2,82 N/mm^2
$k_{c,90}$ = 1,75
$f_{c,90,k}$ = 2,50 N/mm^2

EN 1995:2014
k_{mod} = 0,8
γ_M = 1,25
$f_{c,90,d}$ = 1,60 N/mm^2

$\sigma_{c,90,d} \leq k_{c,90} \cdot f_{c,90,d}$ 2,82 < 2,80 N/mm^2
verification not passed
REQUIRES REINFORCEMENT

Italy - NTC 2018
k_{mod} = 0,8
γ_M = 1,45
$f_{c,90,d}$ = 1,38 N/mm^2

$\sigma_{c,90,d} \leq k_{c,90} \cdot f_{c,90,d}$ 2,82 < 2,41 N/mm^2
verification not passed
REQUIRES REINFORCEMENT

PERPENDICULAR COMPRESSION VERIFICATION OF SUPPORT - BEAM WITH REINFORCEMENT (EN 1995:2014 and ETA-11/0030) : $F_{c,90,Rd} \leq R_{c,90,Rd}$

$$R_{c,90,Rd} = min \begin{cases} k_{c,90} \cdot B \cdot l_{ef,1} \cdot f_{c,90d} + n \cdot R_{ax,Rd} \\ B \cdot l_{ef,2} \cdot f_{c,90d} \end{cases}$$

REINFORCEMENT CONNECTOR SELECTION

VGS 9 x 360 mm

L = 360 mm

b = 350 mm

$n_0 = 2$

$n_{90} = 2$

$n = n_0 \cdot n_{90} = 4$

$$l_{ef,2} = L + (n_0 - 1) \cdot a_1 + min\ (a_{1,CG} ; L)$$

$l_{ef,2}$ = 555 mm

The minimum distances for placement of the connectors are found in the table on p.191.
In this example it is assumed a_1 = 50 mm and $a_{1,CG}$ = 145 mm.

$$R_{ax,Rd} = min \begin{cases} R_{ax,d} = \dfrac{R_{ax,\alpha,Rk} \cdot k_{mod}}{\gamma_M} \\ R_{ki,d} = \dfrac{R_{ki,k}}{\gamma_{M1}} \end{cases}$$

$R_{ax,90°,Rk}$ = 39,78 kN

$R_{ki,k}$ = 17,25 kN

The compression resistance of the connectors calculated here is shown in the table on p.192.

EN 1995:2014

k_{mod} = 0,8

γ_M = 1,3

γ_{M1} = 1,00

$R_{ax,90°,Rd}$ = 24,48 kN

$R_{ki,d}$ = 17,25 kN

$R_{ax,Rd}$ = 17,25 kN

Italy - NTC 2018

k_{mod} = 0,8

γ_M = 1,5

γ_{M1} = 1,05

$R_{ax,90°,Rd}$ = 21,22 kN

$R_{ki,d}$ = 16,43 kN

$R_{ax,Rd}$ = 16,43 kN

$$R_{c,90,Rd} = min \begin{cases} k_{c,90} \cdot B \cdot l_{ef,1} \cdot f_{c,90d} + n \cdot R_{ax,Rd} \\ B \cdot l_{ef,2} \cdot f_{c,90d} \end{cases}$$

EN 1995:2014

$R_{c,90,Rd}$ = 195,36 kN

$F_{c,90,Rd} \leq R_{c,90,Rd}$ 158 < 195,36 kN

verification passed

Italy - NTC 2018

$R_{c,90,Rd}$ = 168,41 kN

$F_{c,90,Rd} \leq R_{c,90,Rd}$ 158 < 168,41 kN

verification passed

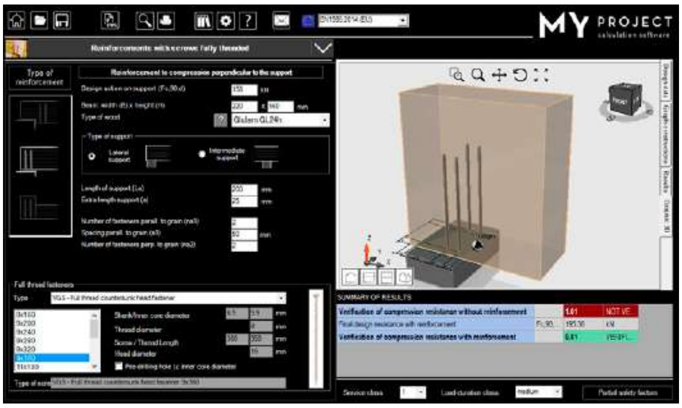

For different calculation configurations, the MyProject software is available (www.rothoblaas.com)

ETA-Danmark A/S
Göteborg Plads 1
DK-2150 Nordhavn
Tel. +45 72 24 59 00
Fax +45 72 24 59 04
Internet www.etadanmark.dk

Authorised and notified according to Article 29 of the Regulation (EU) No 305/2011 of the European Parliament and of the Council of 9 March 2011

MEMBER OF EOTA

European Technical Assessment ETA-11/0030 of 2020/12/10

I General Part

Technical Assessment Body issuing the ETA and designated according to Article 29 of the Regulation (EU) No 305/2011: ETA-Danmark A/S

**Trade name of the construction product:**	Rotho Blaas Self-tapping screws and threaded rods
**Product family to which the above construction product belongs:**	Screws and threaded rods for use in timber constructions
**Manufacturer:**	Rotho Blaas s.r.l<br>Via dell'Adige 2/1<br>IT-38040 Cortaccia (BZ)<br>Tel. + 39 0471 818400<br>Fax + 39 0471 818484<br>Internet www.rothoblaas.com
**Manufacturing plant:**	Rotho Blaas s.r.l. - Held on file by ETA-Danmark AS
**This European Technical Assessment contains:**	72 pages including 7 annexes which form an integral part of the document
**This European Technical Assessment is issued in accordance with Regulation (EU) No 305/2011, on the basis of:**	European Assessment document (EAD) no. EAD 130118-01-0603 “Screws and threaded rods for timber constructions”
**This version replaces:**	The previous ETA with the same number issued on 2019-10-08

Translations of this European Technical Assessment in other languages shall fully correspond to the original issued document and should be identified as such.

Communication of this European Technical Assessment, including transmission by electronic means, shall be in full (excepted the confidential Annex(es) referred to above). However, partial reproduction may be made, with the written consent of the issuing Technical Assessment Body. Any partial reproduction has to be identified as such.

II SPECIFIC PART OF THE EUROPEAN TECHNICAL ASSESSMENT

1 Technical description of product

Technical description of the product
Rotho Blaas “HBS”, “HBSP”, “TBS”, “KKF“, “SCI“, “VGS”, “VGZ”, “DGZ”, “KKT”, “HBSH”, “VGZH”, “VGSH” and “LBS” screws and “RTR” threaded rods are self-tapping screws to be used in timber structures. “HBS” screws are also called “SCH”, “GHS” “SNK” or “SHS” screws, “HBSP” screws are also called “GHS+” or “KGL” screws, “KKF” screws are also called „GHKF”, “KGA” or screws and “TBS” screws are also called “GHSK”, “TLL” or screws, “VGS” screws are also called “GWS” screws, “VGZ” screws are also called “GWZ” screws, “KKT” screws are also called “MN” screws. Rotho Blaas “HBS”, “HBSP”, “TBS”, “KKF“, “HBSH” and “SCI” screws shall be threaded over a part of the length. Rotho Blaas “VGS”, “VGZ”, “VGZH”, “VGSH” and “LBS” screws and “RTR” threaded rods shall be threaded over the full length. Rotho Blaas “DGZ“ and “KKT” screws shall have two threaded parts over the length. The screws shall be produced from carbon steel wire for nominal diameters of 3,0 mm to 13,0 mm and from stainless steel wire for nominal diameters of 3,5 mm to 8,0 mm. The nominal diameter of “RTR” threaded rods is 16 mm or 20 mm. Rotho Blaas "VGU" washers are used for VGS self-tapping screws in timber structures. The washers shall be produced from carbon or stainless steel. Where corrosion protection is required, the material or coating shall be declared in accordance with the relevant specification given in Annex A of EN 14592.

Geometry and Material
The nominal diameter (outer thread diameter), d, shall not be less than 3,0 mm and shall not be greater than 20,0 mm. The overall length, L, of screws shall not be less than 20 mm and shall not be greater than 1200 mm. The overall length of the threaded rods shall not be greater than 3000 mm. Other dimensions are given in Annex A.

Screw types “HBS”, “SCH”, “GHS”, “SNK”, “SHS”, “HBSP”, “GHS+”, “KGL”, “TBS”, „GHSK”, “TLL”, “DGZ”, “LBS”, “VGS”, “GWS”, “VGZ”, “GWZ” and “HBSH” , “VGZH”, “VGSH” and “RTR” threaded rods are made from carbon steel.

Screw types “KKF“ and “GHKF” are made from martensitic stainless steel 1.4006 and SCI are made from stainless steel grade 1.4401 or 1.4567.

Screw types “KKT” are made from either carbon steel or stainless steel.

The ratio of inner thread diameter to outer thread diameter d_1/d ranges from 0,55 to 0,75.

The screws are threaded over a minimum length ℓ_g of 4,0·d (i.e. $\ell_g \geq 4{,}0 \cdot d$).

The lead p (distance between two adjacent thread flanks) ranges from 0,35·d to 0,76·d.

No breaking shall be observed at a bend angle, α, of less than $(45/d^{0,7} + 20)$ degrees.

2 Specification of the intended use in accordance with the applicable European Assessment Document (hereinafter EAD)

The screws are used for connections in load bearing timber structures between members of solid timber, glued laminated timber, cross-laminated timber, and laminated veneer lumber (softwood and hardwood), similar glued members, wood-based panels or steel. The threaded rods are used for connections in load bearing timber structures between softwood members of solid timber, glued laminated timber, cross-laminated timber and laminated veneer lumber or similar glued members.

Rotho Blaas “VGS”, “VGZ”, “VGZH” and “VGSH” screws and “RTR” threaded rods are also used as tensile, compressive reinforcement perpendicular to the grain or shear reinforcement. Furthermore, Rotho Blaas screws with diameters between 6 mm and 12 mm may also be used for the fixing of thermal insulation material on rafters and on vertical facades.

Steel plates and wood-based panels except solid wood panels and cross laminated timber shall only be located on the side of the screw head. The following wood-based panels may be used:

- Plywood according to EN 636 or or ETA
- Particleboard according to EN 312 or or ETA
- Oriented Strand Board, Type OSB/3 and OSB/4 according to EN 300 or ETA
- Fibreboard according to EN 622-2 and 622-3 or ETA (minimum density 650 kg/m³) or national provisions that apply at the installation site
- Cement bonded particleboard according to EN 634 or ETA
- Solid wood panels according to EN 13353 and EN 13986 and cross laminated timber according to ETA
- Laminated Veneer Lumber according to EN 14374 or European Technical Approval
- FST according to ETA-14/0354
- Engineered wood products according to ETA, provided that the ETA for the product provides provisions for the use of self-tapping screws and these provisions are applied

The screws shall be driven into the wood without pre-drilling or after pre-drilling. The threaded rods shall be driven into softwood after pre-drilling

The screws or threaded rods are intended to be used in timber connections for which requirements for mechanical resistance and stability and safety in use in the sense of the Basic Works Requirements 1 and 4 of Regulation 305/2011 (EU) shall be fulfilled.

The design of the connections shall be based on the characteristic load-carrying capacities of the screws or threaded rods. The design capacities shall be derived from the characteristic capacities in accordance with Eurocode 5 or an appropriate national code (e.g. DIN 1052:2008-12).

The screws or threaded rods are intended for use for connections subject to static or quasi static loading.

Section 3.11 of this ETA contains the corrosion protection for Rotho Blaas screws or threaded rods made from carbon steel and the material number of the stainless steel. The screws are for use in timber structures subject to service classes 1, 2 and 3 of Eurocode 5. In service class 1 and 2 the corrosion protection is given according to EN1995-1-1, or by equivalent measures. In service class 3 the corrosion protection is given according to EN1995-1-1 or by stainless steel. Alternatively, a Zn-Al flakes coating (also called "evo coating") can be used as corrosion protection in service class 3."

The scope of the screws or threaded rods regarding resistance to corrosion shall be defined according to national provisions that apply at the installation site considering environmental conditions.

The provisions made in this European Technical Assessment are based on an assumed intended working life of the screws of 50 years.

The indications given on the working life cannot be interpreted as a guarantee given by the producer or Assessment Body, but are to be regarded only as a means for choosing the right products in relation to the expected economically reasonable working life of the works.

3 Performance of the product and references to the methods used for its assessment

Characteristic	Assessment of characteristic
**3.1 Mechanical resistance and stability*) (BWR1)**	
Tensile strength	Characteristic value $f_{tens,k}$:
Screw made from carbon steel “HBS”, “SCH”, “GHS”, “SNK”, “SHS”, “HBSP”, “GHS+”, “KGL”, “TBS”, “GHSK”, “TLL”, “DGZ”, “LBS”, “VGS”, “GWS”, “VGZ”, “GWZ”, “KKT”, “MN” and screws made from stainless steel “KKF” and “GHKF”	Screw d = 3,0 mm: 2,8 kN<br>Screw d = 3,5 mm: 3,8 kN<br>Screw d = 4,0 mm: 5,0 kN<br>Screw d = 4,5 mm: 6,4 kN<br>Screw d = 5,0 mm: 7,9 kN<br>Screw d = 5,3 mm: 11,0 kN<br>Screw d = 5,6 mm: 12,3 kN<br>Screw d = 6,0 mm: 11,3 kN<br>Screw d = 7,0 mm: 15,4 kN<br>Screw d = 8,0 mm: 20,1 kN<br>Screw d = 9,0 mm: 25,4 kN<br>Screw d = 10,0 mm: 31,4 kN<br>Screw d = 11,0 mm: 38,0 kN<br>Screw d = 12,0 mm: 33,9 kN<br>Screw d = 13,0 mm: 53,0 kN
Screws made from carbon steel “HBSH” , “VGZH”, “VGSH”	Screw d = 6,0 mm: 18 kN<br>Screw d = 8,0 mm: 32 kN
Threaded rods made from carbon steel “RTR”	Rod d = 16,0 mm: 100 kN<br>Rod d = 20,0 mm: 145 kN
Screws made from stainless steel “KKT”, “MN” and “SCI“	Screw d = 3,5 mm: 2,1 kN<br>Screw d = 4,0 mm: 2,8 kN<br>Screw d = 4,5 mm: 3,5 kN<br>Screw d = 5,0 mm: 4,3 kN<br>Screw d = 6,0 mm: 6,2 kN<br>Screw d = 8,0 mm: 11,1 kN
Insertion moment	Ratio of the characteristic torsional strength to the mean insertion moment:<br>$f_{tor,k} / R_{tor,mean} \geq 1,5$
Torsional strength	Characteristic value $f_{tor,k}$:
Screw made from carbon steel “HBS”, “SCH”, “GHS”, , “HTP”, “SNK”, “HBSP”, “GHS+”, “KGL”, “TBS”, “GHSK”, “TLL”, “DGZ”, “LBS”, “VGS”, “GWS”, “VGZ”, “GWZ”, “KKT”, “MN” and screws made from stainless steel “KKF” and “GHKF”	Screw d = 3,0 mm: 1,3 Nm<br>Screw d = 3,5 mm: 2,0 Nm<br>Screw d = 4,0 mm: 3,0 Nm<br>Screw d = 4,5 mm and LBS screw d = 5,0 mm: 5,0 Nm<br>Screw d = 5,0 mm (except LBS): 7,5 Nm<br>Screw d = 5,3 mm: 9,0 Nm<br>Screw d = 5,6 mm: 10,0 Nm<br>Screw d = 6,0 mm: 12,0 Nm<br>Screw d = 7,0 mm: 18,0 Nm<br>Screw d = 8,0 mm: 28,0 Nm<br>Screw d = 9,0 mm: 36,0 Nm<br>Screw d = 10,0 mm: 40,0 Nm<br>Screw d = 11,0 mm: 61,0 Nm<br>Screw d = 12,0 mm: 60,0 Nm<br>Screw d = 13,0 mm: 95,0 Nm

Characteristic	Assessment of characteristic
Screws made from carbon steel “HBSH” , “VGZH”, “VGSH”	Screw d = 6,0 mm: 18,0 Nm<br>Screw d = 8,0 mm: 38,0 Nm
Threaded rods made from carbon steel “RTR”	Screw d = 16,0 mm: 300 Nm<br>Screw d = 20,0 mm: 500 Nm
Screws made from stainless steel “KKT”, “MN” and “SCI“	Screw d = 3,5 mm: 1,5 Nm<br>Screw d = 4,0 mm: 2,0 Nm<br>Screw d = 4,5 mm: 3,0 Nm<br>Screw d = 5,0 mm: 5,0 Nm<br>Screw d = 6,0 mm: 8,0 Nm<br>Screw d = 8,0 mm: 18,0 Nm
**3.2 Safety in case of fire (BWR2)**	
Reaction to fire	The screws are made from steel classified as class A1 in accordance with EN 13501-1 and Commission Delegated Regulation 2016/364.
**3.8 General aspects related to the performance of the product**	The screws have been assessed as having satisfactory durability and serviceability when used in timber structures using the timber species described in Eurocode 5 and subject to the conditions defined by service classes 1, 2 and 3

*) See additional information in section 3.9 – 3.12.

3.9 Mechanical resistance and stability

The load-carrying capacities for Rotho Blaas screws or threaded rods are applicable to the wood-based materials mentioned in paragraph 1 even though the term timber has been used in the following.

The characteristic lateral load-carrying capacities and the characteristic axial withdrawal capacities of Rotho Blaas screws and threaded rods should be used for designs in accordance with Eurocode 5 or an appropriate national code.

Point side penetration length must be $\ell_{ef} \geq 4 \cdot d$, where d is the outer thread diameter of the screw or threaded rod. For the fixing of rafters, point side penetration must be at least 40 mm, $\ell_{ef} \geq 40$ mm.

Point or head side penetration thread length for screws made of carbon steel driven without pre-drilling in Beech LVL according to EN 14374 or in FST according to ETA-14/0354 must not exceed the following values:

HBSH screw d = 6,0 mm: $l_{ef} \leq 90$ mm
HBSH screw d = 8,0 mm: $l_{ef} \leq 100$ mm

Accumulated point and head side penetration length for threaded rods must not exceed 2200 mm.

Screws made of carbon steel except “KKT” or “HBSH”, “VGZH”, “VGSH” shall be driven in pre-drilled holes if the characteristic member density exceeds 550 kg/m³.

Screws made of stainless steel shall be driven in pre-drilled holes if the characteristic member density exceeds 500 kg/m³.

European Technical Assessments for structural members or wood-based panels must be considered where applicable.

Reductions in the cross-sectional area caused by Rotho Blaas screws or threaded rods with a diameter of 10 mm or more shall be taken into account in the member strength verification both, in the tensile and compressive area of members.

For screws or threaded rods in pre-drilled holes, the drill hole diameter should be considered in the member strength verification, for screws driven without pre-drilling, the inner thread diameter.

For wood-based panels the relevant ETA’s must be considered where applicable.

Lateral load-carrying capacity
The characteristic lateral load-carrying capacity of Rotho Blaas screws or threaded rods shall be calculated according to EN 1995-1-1:2008 (Eurocode 5) using the outer thread diameter d as the effective diameter of the screw. For steel-to-timber connections with screws LBS d = 5 mm, a thick steel plate may be assumed for steel plate thickness t ≥ 1,5 mm.
The characteristic yield moment is:

Rotho Blaas screws or threaded rods made from carbon steel except “HBSH” , “VGZH”, “VGSH” and “KKF” and “GHKF” screws made from stainless steel:

Screw d = 3,0 mm:	$M_{y,k}$ = 1,4 Nm
Screw d = 3,5 mm:	$M_{y,k}$ = 2,1 Nm
Screw d = 4,0 mm:	$M_{y,k}$ = 3,0 Nm
Screw d = 4,5 mm:	$M_{y,k}$ = 4,1 Nm
Screw d = 5,0 mm:	$M_{y,k}$ = 5,4 Nm
Screw d = 5,3 mm:	$M_{y,k}$ = 9,2 Nm
Screw d = 5,6 mm:	$M_{y,k}$ = 10,6 Nm
Screw d = 6,0 mm:	$M_{y,k}$ = 9,5 Nm
Screw d = 7,0 mm:	$M_{y,k}$ = 14,2 Nm
Screw d = 8,0 mm:	$M_{y,k}$ = 20,1 Nm
Screw d = 9,0 mm:	$M_{y,k}$ = 27,2 Nm
Screw d = 10,0 mm:	$M_{y,k}$ = 35,8 Nm
Screw d = 11,0 mm:	$M_{y,k}$ = 45,9 Nm
Screw d = 12,0 mm:	$M_{y,k}$ = 48,0 Nm
Screw d = 13,0 mm:	$M_{y,k}$ = 70,9 Nm
Rod d = 16,0 mm:	$M_{y,k}$ = 200 Nm
Rod d = 20,0 mm:	$M_{y,k}$ = 350 Nm

Rotho Blaas screws made from carbon steel “HBSH”, “VGZH”, “VGSH”:

Screw d = 6,0 mm:	$M_{y,k}$ = 15,8 Nm
Screw d = 8,0 mm:	$M_{y,k}$ = 33,4 Nm

Rotho Blaas screws made from stainless steel “KKT”, “MN” and “SCI”:

Screw d = 3,5 mm:	$M_{y,k}$ = 1,3 Nm
Screw d = 4,0 mm:	$M_{y,k}$ = 2,0 Nm
Screw d = 4,5 mm:	$M_{y,k}$ = 2,8 Nm
Screw d = 5,0 mm:	$M_{y,k}$ = 4,5 Nm
Screw d = 6,0 mm:	$M_{y,k}$ = 8,2 Nm
Screw d = 8,0 mm:	$M_{y,k}$ = 15 Nm

where
d outer thread diameter [mm]

The embedding strength for screws in non-pre-drilled holes in softwood or hardwood arranged at an angle between screw axis and grain direction, $0° \leq \alpha \leq 90°$ is:

$$f_{h,k} = \frac{0,082 \cdot \rho_k \cdot d^{-0,3}}{2,5 \cdot \cos^2 \alpha + \sin^2 \alpha} \qquad [\text{N/mm}^2]$$

and accordingly for screws in pre-drilled holes in softwood and hardwoods beech and oak or threaded rods in softwood:

$$f_{h,k} = \frac{0,082 \cdot \rho_k \cdot (1 - 0,01 \cdot d)}{2,5 \cdot \cos^2 \alpha + \sin^2 \alpha} \qquad [\text{N/mm}^2]$$

Where
ρ_k characteristic timber density [kg/m³], with a maximum characteristic density of 590 kg/m³;
d outer thread diameter [mm];

α angle between screw or rod axis and grain direction.

The embedding strengths given above may be applied for screws or threaded rods within single softwood layers in cross laminated timber, if the single layer is considered as a separate softwood member and the minimum spacing, end and edge distances are observed for the single layer. For inner layers, the edge distance perpendicular to the grain may be reduced to 3 · d.

Alternatively, the embedding strength for screws or threaded rods arranged parallel to the plane of cross laminated timber (layers of softwood), independent of the angle between screw axis and grain direction, $0° \leq \alpha \leq 90°$, may be calculated from:

$$f_{h,k} = 20 \cdot d^{-0,5} \qquad \text{[N/mm²]}$$

unless otherwise specified in the technical specification (ETA or hEN) for the cross laminated timber.
Where
d outer thread diameter [mm]

The embedding strength for screws or threaded rods in the wide face of cross laminated timber should be assumed as for solid timber based on 110 % of the lowest characteristic density of a board layer. If relevant, the angle between force and grain direction of the outer layer should be taken into account.

The direction of the lateral force shall be perpendicular to the screw axis and parallel to the wide face of the cross laminated timber.

The embedding strength for screws in non-pre-drilled holes in softwood LVL arranged at an angle between screw axis and grain direction, $0° \leq \alpha \leq 90°$ is:

$$f_{h,k} = \frac{0,082 \cdot \rho_k \cdot d^{-0,3}}{\left(2,5 \cdot \cos^2\alpha + \sin^2\alpha\right)\left(1,5 \cdot \cos^2\beta + \sin^2\beta\right)} \qquad \text{[N/mm²]}$$

and accordingly for screws or threaded rods in pre-drilled holes in softwood LVL:

$$f_{h,k} = \frac{0,082 \cdot \rho_k \cdot (1 - 0,01 \cdot d)}{\left(2,5 \cdot \cos^2\alpha + \sin^2\alpha\right)\left(1,5 \cdot \cos^2\beta + \sin^2\beta\right)} \qquad \text{[N/mm²]}$$

Where
ρ_k characteristic timber density [kg/m³];
d outer thread diameter [mm];
α angle between screw axis and grain direction;
β angle between screw axis and the LVL's wide face ($0° \leq \beta \leq 90°$).

The embedding strength for screws in pre-drilled or non-pre-drilled holes in Beech LVL according to EN 14374 or in FST according to ETA-14/0354 is:

$$f_{h,k} = \frac{0,082 \cdot \rho_k \cdot d^{-0,15}}{\left(2,5 \cdot \cos^2\alpha + \sin^2\alpha\right) \cdot k_\varepsilon \cdot k_\beta} \qquad \text{[N/mm²]}$$

Where
ρ_k characteristic density [kg/m³];
d outer thread diameter [mm];
α angle between screw axis and grain direction, $0° \leq \alpha \leq 90°$;
k_ε $= (0,5 + 0,024 \cdot d) \cdot \sin^2\varepsilon + \cos^2\varepsilon$;
ε angle between load and grain direction; $0° \leq \varepsilon \leq 90°$;
k_β $= 1,2 \cdot \cos^2\beta + \sin^2\beta$;
β angle between screw axis and wide face of LVL or FST member, $0° \leq \beta \leq 90°$.

The definition of the angles α, β and ε is given in Annex G.

For laterally loaded screws, the rules for multiple fastener connections in EN 1995-1-1, 8.3.1.1 (8) should be applied. For laterally loaded threaded rods, the rules for multiple fastener connections in EN 1995-1-1, 8.5.1.1 (4) should be applied.

The lateral slip modulus K_{ser} for the serviceability limit state of screws in non-predrilled holes is given in EN 1995-1-1 (Eurocode 5) clause 7.1 and table 7.1 as for nails in non-predrilled holes.

The lateral slip modulus K_{ser} for the serviceability limit state of screws in predrilled holes is given in EN 1995-1-1 (Eurocode 5) clause 7.1 and table 7.1 as for nails in predrilled holes.

The lateral slip modulus K_{ser} for the serviceability limit state of a "HBS", "HBSP", "SHS" or "TBS" screw loaded in single shear in softwood LVL and 5 mm $\leq$ d $\leq$ 10 mm should be taken independent of angle α to the grain as:

$$K_{ser} = 60 \cdot \left(\frac{d \cdot \rho_{mean}}{510}\right)^{1,5} \qquad \text{[N/mm]}$$

Where:
d outer thread diameter [mm],
ρ_{mean} softwood LVL mean density [kg/m³];

Axial withdrawal capacity

The characteristic axial withdrawal capacity of Rotho Blaas screws or threaded rods (only softwood) in solid timber (softwood and hardwood with a maximum characteristic density of 590 kg/m³), glued laminated timber (softwood and hardwood with a maximum characteristic density of 590 kg/m³), cross-laminated timber or laminated veneer lumber (softwood and hardwood or FST according to ETA-14/0354 with maximum characteristic density of 750 kg/m³) members at an angle of $0° \leq \alpha \leq 90°$ to the grain shall be calculated according to EN 1995-1-1:2008 from:

$$F_{ax,\alpha,Rk} = \frac{n_{ef} \cdot k_{ax} \cdot f_{ax,k} \cdot d \cdot \ell_{ef}}{k_\beta}\left(\frac{\rho_k}{\rho_a}\right)^{0,8} \qquad \text{[N]}$$

Where

$F_{ax,\alpha,RK}$ characteristic withdrawal capacity of the screws at an angle α to the grain [N]

n_{ef} effective number of screws according to EN 1995-1-1:2008

k_{ax} $k_{ax} = 1{,}0$ for $45° \leq \alpha \leq 90°$

$$k_{ax} = a + \frac{b \cdot \alpha}{45°} \quad \text{for } 0° \leq \alpha < 45°$$

$$a = \begin{cases} 0{,}5 & \text{for LVL} \\ 0{,}3 & \text{for timber} \end{cases}$$

$$b = \begin{cases} 0{,}5 & \text{for LVL} \\ 0{,}7 & \text{for timber} \end{cases}$$

k_β $k_\beta = 1{,}0$ for timber

$k_\beta = 1{,}5 \cdot \cos^2 \beta + \sin^2 \beta$ for LVL

$f_{ax,k}$ Characteristic withdrawal parameter

For screws in solid or glued laminated timber, cross laminated timber and SWP members with maximum characteristic density of 440 kg/m³ and ρ_a = 350 kg/m³:

$f_{ax,k}$ = 11,7 N/mm²

For threaded rods in solid or glued laminated timber and cross laminated timber members with maximum characteristic density of 440 kg/m³ and ρ_a = 350 kg/m³:

$f_{ax,k}$ = 9,0 N/mm²

For screws in non pre-drilled LVL with 460 kg/m³ ≤ ρ_k ≤ 550 kg/m³ and ρ_a = 500 kg/m³:

$f_{ax,k}$ = 15,0 N/mm²

For KKT screws in hardwood members (Oak or Beech) with maximum characteristic density of 590 kg/m³ and ρ_a = 530 kg/m³:

$f_{ax,k}$ = 28,0 N/mm²

For screws in pre-drilled LVL or FST (ETA-14/0354) with 590 kg/m³ ≤ ρ_k ≤ 750 kg/m³ and ρ_a = 730 kg/m³:

$f_{ax,k}$ = 29,0 N/mm²

For HBSH, VGZH, VGSH screws in non-pre-drilled LVL or FST (ETA-14/0354) with 590 kg/m³ ≤ ρ_k ≤ 750 kg/m³ and ρ_a = 730 kg/m³:

$f_{ax,k}$ = 42,0 N/mm²

For screws in pre-drilled hardwood members with maximum characteristic density of 590 kg/m³:

$f_{ax,k} = 7 \cdot 10^{-4} \cdot \rho_k^{1,6} \cdot d^{-0,34}$ N/mm²

For HBSH, VGSH and VGZH screws in non-predrilled hardwood members (oak, beech) with maximum characteristic density of 590 kg/m³ and ρ_a = 530 kg/m³:

$f_{ax,k}$ = 22,0 N/mm²

For HBSH, VGSH and VGZH screws in non-predrilled hardwood members (ash) with maximum characteristic density of 590 kg/m³ and ρ_a = 530 kg/m³:

$f_{ax,k}$ = 30,0 N/mm²

d outer thread diameter [mm]

ℓ_{ef} penetration length of the threaded part according to EN 1995-1-1:2008 [mm]

α angle between grain and screw axis, 0° ≤ α ≤ 90°, for threaded rods 15° ≤ α ≤ 90°,

β angle between screw axis and the LVL's wide face (0° ≤ α ≤ 90°)

ρ_k characteristic density [kg/m³]

ρ_a associated density for $f_{ax,k}$ [kg/m³]

For screws or threaded rods penetrating more than one layer of cross laminated timber, the different layers may be taken into account proportionally.

The axial withdrawal capacity for screws arranged parallel to the plane of cross laminated timber, independent of the angle between screw axis and grain direction, 0° ≤ α ≤ 90°, may be calculated from:

$$F_{ax,Rk} = 20 \cdot d^{0,8} \cdot \ell_{ef}^{0,9} \quad \text{[N]}$$

Where

d outer thread diameter [mm]^

ℓ_{ef} Penetration length of the threaded part according to EN 1995-1-1:2008 [mm]

The axial withdrawal capacity is limited by the head pull-through capacity and the tensile capacity of the screw.

For axially loaded screws or threaded rods in tension, where the external force is parallel to the screw axes, the rules in EN 1995-1-1, 8.7.2 (8) should be applied.

For inclined screws or threaded rods in timber-to-timber or steel-to-timber shear connections, where the screws or threaded rods are arranged under an angle 30° ≤ α ≤ 60° between the shear plane and the screw axis, the effective number of screws n_{ef} should be determined as follows:

For one row of n screws or threaded rods parallel to the load, the load-carrying capacity should be calculated using the effective number of fasteners n_{ef}, where

$$n_{ef} = \max\left\{n^{0,9}\,;0{,}9 \cdot n\right\}$$

and n is the number of inclined screws or threaded rods in a row. If crossed pairs of screws or threaded rods are used in timber-to-timber connections, n is the number of crossed pairs of screws or threaded rods in a row.

Note: For screws or threaded rods as reinforcement or inclined screws or threaded rods as fasteners in mechanically jointed beams or columns or for the fixing of thermal insulation material, n_{ef} = n.

The axial slip modulus K_{ser} of the threaded part of a screw or threaded rod for the serviceability limit state should be taken independent of angle α to the grain as:

$K_{ser} = 25 \cdot d \cdot \ell_{ef}$ [N/mm] for fasteners in softwood

$K_{ser} = 30 \cdot d \cdot \ell_{ef}$ [N/mm] for screws in hardwood

Where

d	outer thread diameter [mm]
ℓ_{ef}	penetration length in the timber member [mm]

Head pull-through capacity

The characteristic head pull-through capacity of Rotho Blaas screws in softwoods, hardwoods and wood-based panels shall be calculated according to EN 1995-1-1:2008 from:

$$F_{ax,\alpha,Rk} = n_{ef} \cdot f_{head,k} \cdot d_h^2 \cdot \left(\frac{\rho_k}{\rho_a}\right)^{0,8} \quad [N]$$

where:

$F_{ax,\alpha,RK}$	characteristic head pull-through capacity of the connection at an angle $\alpha \geq 30°$ to the grain [N]
n_{ef}	effective number of screws according to EN 1995-1-1:2008<br>For inclined screws: $n_{ef} = \max\left\{n^{0,9} ; 0,9 \cdot n\right\}$<br>(see axial withdrawal capacity)
$f_{head,k}$	characteristic head pull-through parameter [N/mm²]
d_h	diameter of the screw head or the washer [mm]. Outer diameter of washers d_k > 32 mm shall not be considered.
ρ_k	characteristic density [kg/m³], for wood-based panels maximum ρ_k = 380 kg/m³, for hardwood maximum ρ_k = 590 kg/m³

Characteristic head pull-through parameter for Rotho Blaas screws or for washer except “KKF” and “KKT” screws in connections with softwood and in connections with wood-based panels with thicknesses above 20 mm and ρ_a = 350 kg/m³:
$f_{head,k}$ = 10,5 N/mm²

Characteristic head pull-through parameter for Rotho Blaas “TBS” screws d = 8 mm with d_k = 24,5 mm in connections with softwood and in connections with wood-based panels with thicknesses above 20 mm and ρ_a = 350 kg/m³:
$f_{head,k}$ = 15 N/mm²

Characteristic head pull-through parameter for Rotho Blaas “KKF” and “KKT” screws in connections with softwood and in connections with wood-based panels with thicknesses above 20 mm and ρ_a = 350 kg/m³:
$f_{head,k}$ = 16,5 N/mm²

Characteristic head pull-through parameter for Rotho Blaas screws with countersunk head in connections with softwood LVL and ρ_a = 500 kg/m³:
$f_{head,k}$ = 20 N/mm²

Characteristic head pull-through parameter for Rotho Blaas “KKF”, “KKT” and “MN” screws in connections with hardwood and ρ_a = 530 kg/m³:
$f_{head,k}$ = 28 N/mm²

Characteristic head pull-through parameter for Rotho Blaas “HBSH” screws with countersunk head 60° in connections with Beech LVL or FST (ETA-14/0354) with maximum ρ_k = 750 kg/m³ and ρ_a = 730 kg/m³:
$f_{head,k}$ = 50 N/mm²

Characteristic head pull-through parameter for screws in connections with wood-based panels with thicknesses between 12 mm and 20 mm:
$f_{head,k}$ = 8 N/mm²

Characteristic head pull-through parameter for Rotho Blaas “HBSH” screws d = 6,0 mm in connections with hardwood (oak, beech, ash) and ρ_a = 530 kg/m³:
$f_{head,k}$ = 28,0 N/mm²

Characteristic head pull-through parameter for Rotho Blaas “HBSH” screws d = 8,0 mm in connections with hardwood (oak, beech, ash) and ρ_a = 530 kg/m³:
$f_{head,k}$ = 24,0 N/mm²

Screws in connections with wood-based panels with a thickness below 12 mm (minimum thickness of the wood based panels of 1,2·d with d as outer thread diameter):
$f_{head,k}$ = 8 N/mm²
limited to $F_{ax,Rk}$ = 400 N

The head diameter d_h of all screws except “KKF” and “KKT” screws shall be greater than $1,8 \cdot d_s$, where d_s is the smooth shank or the wire diameter. Otherwise the characteristic head pull-through capacity $F_{ax,\alpha,Rk}$ = 0.

The minimum thickness of wood-based panels according to the clause 3.12 must be observed.

In steel-to-timber connections the head pull-through capacity may be disregarded.

Tensile capacity

The characteristic tensile strength $f_{tens,k}$ of screws or threaded rods made from carbon steel except “HBSH”, “VGZH”, “VGSH” and of screws “KKF” and “GHKF” screws made from stainless steel is:

Screw d = 3,0 mm:	2,8 kN
Screw d = 3,5 mm:	3,8 kN
Screw d = 4,0 mm:	5,0 kN
Screw d = 4,5 mm:	6,4 kN
Screw d = 5,0 mm:	7,9 kN
Screw d = 5,3 mm:	11,0 kN
Screw d = 5,6 mm:	12,5 kN
Screw d = 6,0 mm:	11,3 kN
Screw d = 7,0 mm:	15,4 kN
Screw d = 8,0 mm:	20,1 kN
Screw d = 9,0 mm:	25,4 kN

Screw d = 10,0 mm: 31,4 kN
Screw d = 11,0 mm: 38,0 kN
Screw d = 12,0 mm: 33,9 kN
Screw d = 13,0 mm: 53,0 kN
Rod d = 16,0 mm: 100 kN
Rod d = 20,0 mm: 145 kN

The characteristic tensile strength $f_{tens,k}$ of “HBSH”, “VGZH”, “VGSH” screws made from carbon steel is:
Screw d = 6,0 mm: 18 kN
Screw d = 8,0 mm: 32 kN

The characteristic tensile strength $f_{tens,k}$ of screws made from stainless steel “KKT”, “MN” and “SCI“ is:
Screw d = 3,5 mm: 2,1 kN
Screw d = 4,0 mm: 2,8 kN
Screw d = 4,5 mm: 3,5 kN
Screw d = 5,0 mm: 4,3 kN
Screw d = 6,0 mm: 6,2 kN
Screw d = 8,0 mm: 11,1 kN

For screws used in combination with steel plates, the tear-off capacity of the screw head should be greater than the tensile strength of the screw.

When determining design values of the tensile capacity it should be used γ_{M2} partial factor according to EN 1993 or to the particular national annex.

Combined laterally and axially loaded screws or threaded rods

For connections subjected to a combination of axial and lateral load, the following expression should be satisfied:

$$\left(\frac{F_{ax,Ed}}{F_{ax,Rd}}\right)^2 + \left(\frac{F_{la,Ed}}{F_{la,Rd}}\right)^2 \leq 1$$

where

$F_{ax,Ed}$ axial design load of the screw or threaded rod
$F_{la,Ed}$ lateral design load of the screw or threaded rod
$F_{ax,Rd}$ design load-carrying capacity of an axially loaded screw or threaded rod
$F_{la,Rd}$ design load-carrying capacity of a laterally loaded screw or threaded rod

Mechanically jointed beams

“VGS”, “VGZ”, “VGSH” and “VGZH” screws with a full thread or “RTR” threaded rods may be used for connections in structural members which are composed of several parts in mechanically jointed beams or columns.

Compressive capacity

The design compressive capacity $F_{c,90,Rd}$ of “VGZ”, “VGS”, “VGZH” and “VGSH” screws or “RTR” threaded rods with full thread along the length embedded in timber with an angle between fastener axis and grain direction of $45° \leq \alpha \leq 90°$ is the minimum of the axial resistance against pushing-in and the buckling resistance:

$$F_{c,90,Rd} = \min\left\{F_{ax,Rd}; F_{ki,Rd}\right\}$$

$$F_{c,90,Rd} = \min\left\{f_{ax,d} \cdot d \cdot \ell_{ef} \cdot \left(\frac{\rho_k}{\rho_a}\right)^{0,8}; \frac{k_c \cdot N_{pl,k}}{\gamma_{M1}}\right\}$$

Where:

$$\kappa_c = \begin{cases} 1 & \text{for } \bar{\lambda}_k \leq 0,2 \\ \dfrac{1}{k + \sqrt{k^2 - \bar{\lambda}_k^2}} & \text{for } \bar{\lambda}_k > 0,2 \end{cases}$$

$$k = 0,5 \cdot \left[1 + 0,49 \cdot (\bar{\lambda}_k - 0,2) + \bar{\lambda}_k^2\right]$$

The relative slenderness ratio shall be calculated from:

$$\bar{\lambda}_k = \sqrt{\frac{N_{pl,k}}{N_{ki,k}}}$$

where

$$N_{pl,k} = \pi \cdot \frac{d_1^2}{4} \cdot f_{y,k} \qquad [N]$$

is the characteristic value for the axial capacity in case of plastic analysis referred to the inner thread cross-section.

Characteristic yield strength of screws from carbon steel:
$f_{y,k}$ = 1000 [N/mm²]

Characteristic yield strength of threaded rods:
$f_{y,k}$ = 640 [N/mm²]

Characteristic ideal elastic buckling load:

$$N_{ki,k} = \sqrt{c_h \cdot E_S \cdot I_S} \qquad [N]$$

Elastic foundation of the screw or threaded rod:

$$c_h = (0,19 + 0,012 \cdot d) \cdot \rho_k \cdot \left(\frac{\alpha}{180°} + 0,5\right) \qquad [N/mm^2]$$

Modulus of elasticity:
E_s = 210000 [N/mm²]

Second moment of area:

$$I_S = \frac{\pi}{64} \cdot d_1^4 \qquad [mm^4]$$

$f_{ax,d}$ design value of the axial withdrawal parameter of the threaded part of the screw [N/mm²]

d_1 inner thread diameter [mm]

d outer thread diameter [mm]
ℓ_{ef} penetration length of the threaded part [mm]

α angle between screw axis and grain direction
ρ_k characteristic density [kg/m³]
ρ_a associated density for $f_{ax,k}$ [kg/m³]
γ_{M1} partial factor according to EN 1993 or to the particular national annex

Compression reinforcement

See Annex C

Thermal insulation material on top of rafters
See Annex D

Shear reinforcement
See Annex E

Tensile reinforcement
See Annex F

3.11 Aspects related to the performance of the product

3.11.1 Corrosion protection
The Rotho Blaas screws or threaded rods are produced from steel wire. Screws or threaded rods made from carbon steel are electrogalvanised and yellow or blue chromate or could be coated with organic coating. The thickness of the zinc coating is minimum 5 µm.

The screws are for use in timber structures subject to service classes 1, 2 and 3 of Eurocode 5. In service class 1 and 2 the corrosion protection is given according to EN1995-1-1, or by equivalent measures.

In service class 3 the corrosion protection is given according to EN1995-1-1 or by stainless steel no. 1.4006, 1.4401 and 1.4567. Alternatively, a Zn-Al flakes coating (also called "evo coating") can be used as corrosion protection in service class 3.

Contact corrosion shall be avoided.

3.12 General aspects related to the intended use of the product

The screws or threaded rods are manufactured in accordance with the provisions of the ETA using the automated manufacturing process and laid down in the technical documentation.

The installation shall be carried out in accordance with Eurocode 5 or an appropriate national code unless otherwise is defined in the following. Instructions from Rotho Blaas s.r.l should be considered for installation.

The screws or threaded rods are used for connections in load bearing timber structures between members of solid timber, glued laminated timber, cross-laminated timber, laminated veneer lumber, similar glued members, wood-based panels or steel members.

The screws or threaded rods may be used for connections in load bearing timber structures with structural members according to an associated ETA, if according to the associated ETA of the structural member a connection in load bearing timber structures with screws according to an ETA is allowed.

Rotho Blaas fully threaded “VGS”, “VGZ”, “VGSH” and “VGZH” screws or “RTR” threaded rods are also used as tensile or compressive reinforcement perpendicular to the grain or as shear reinforcement.

Furthermore the screws with diameters of at least 6 mm may also be used for the fixing of insulation on top of rafters.

A minimum of two screws or threaded rods should be used for connections in load bearing timber structures. This does not apply for reinforcements or for the fixing of battens, rafters, purlins or similar on main beams or top plates, if the member is fixed with at least two screws in total.

The minimum penetration depth in structural members made of solid, glued or cross-laminated timber or laminated veneer lumber is:

$$\ell_{ef,req} = \min\left\{\frac{4 \cdot d}{\sin\alpha}; 20 \cdot d\right\}$$

Wood-based panels and steel plates should only be arranged on the side of the screw head. The minimum thickness of wood-based panels should be 1,2·d. Furthermore, the minimum thickness for following wood-based panels should be:

- Plywood, Fibreboards: 6 mm
- Particleboards, OSB, Cement Particleboards: 8 mm
- Solid wood panels: 12 mm

For structural members according to ETA’s the terms of the ETA’s must be considered.

If screws with an outer thread diameter d > 8 mm are used in load bearing timber structures, the structural solid or glued laminated timber, laminated veneer lumber and similar glued members must be from spruce, pine or fir. This does not apply for screws or threaded rods in pre-drilled holes.

The screws shall be driven into softwood with or without pre-drilling. The threaded rods shall be driven into softwood with pre-drilling. Except “HBSH”, “VGSH”, “VGZH” or “KKT” screws, the screws shall be driven into hardwood with a maximum characteristic density of 590 kg/m³ after pre-drilling. “HBSH”, “VGSH”, “VGZH” screws made of carbon steel may be driven into Beech LVL according to EN 14374 or in FST according to ETA-14/0354 without pre-drilling or after pre-drilling.

The maximum total penetration length of “VGZH” and “VGSH” screws with 6 mm or 8 mm diameter without pre-drilling or after partial pre-drilling is limited to:

Outer thread diameter [mm]	Maximum total penetration length [mm]	
	Hardwood non-predrilled	Beech LVL with pilot hole
6,0	260	180 mm with pilot hole ≥ 50 mm
		260 mm with pilot hole ≥ 110 mm
8,0	320	280 mm with pilot hole ≥ 100 mm
		320 mm with pilot hole ≥ 150 mm

The drill hole diameters are:

Outer thread diameter [mm]	Drill hole diameter [mm]	
	Softwood	Hardwood
3,0	2,0	-
3,5	2,0	-
4,0	2,5	-
4,5	2,5	-
5,0	3,0	3,5
5,3	3,5	4,0
5,6	3,5	4,0
6,0	4,0	4,0
7,0	4,0	5,0
8,0	5,0	6,0
9,0	5,0	6,0
10,0	6,0	7,0
11,0	6,0	7,0
12,0	7,0	8,0
13,0	8,0	9,0
16,0	13,0	-
20,0	16,0	-

The hole diameter in steel members must be predrilled with a suitable diameter.

Only the equipment prescribed by Rotho Blaas SRL shall be used for driving the screws.

In connections with screws with countersunk head according to Annex A, the head must be flush with the surface of the connected structural member. A deeper countersink is not allowed.

For screws or threaded rods arranged at angles $\alpha < 90°$ between screw axis and grain direction minimum spacing and distances are defined as follows:
Minimum spacing a_1 or a_2 is defined perpendicular to the fastener axis, minimum end or edge distances $a_{1,c}$, $a_{1,t}$, $a_{2,c}$ or $a_{2,t}$ parallel or perpendicular to the grain, respectively, are defined between the centre of the threaded length (axial loading) or the length (lateral loading) in the respective timber member and the member surface as for axially loaded screws in Figure 8.11.a EN 1995-1-1.

Laterally and/or axially loaded screws or threaded rods
For structural timber members, minimum spacing and distances for laterally or axially loaded screws or threaded rods in predrilled holes are given in EN 1995-1-1:2008 (Eurocode 5) clause 8.3.1.2 and table 8.2 as for nails in predrilled holes. Here, the outer thread diameter d must be considered.

For threaded rods, the minimum timber member thickness is $4 \cdot d$ where d id the outer thread diameter.

For screws in non-predrilled holes, minimum spacing and distances are given in EN 1995-1-1:2008 (Eurocode 5) clause 8.3.1.2 and table 8.2 as for nails in non-predrilled holes.

Minimum distances and spacing for “KKT” screws in non-predrilled holes in members with a minimum thickness $t = 4 \cdot d$ and a minimum width of 12·d or 60 mm, whichever is the greater, may be taken as:

Spacing a_1 parallel to the grain $a_1 = 8 \cdot d$
Spacing a_2 perpendicular to the grain $a_2 = 4 \cdot d$
Loaded end distance: $a_{3,t} = 12 \cdot d$
Unloaded end distance: $a_{3,c} = 5 \cdot d$
Loaded edge distance: $a_{4,t} = 5 \cdot d$
Unloaded edge distance: $a_{4,c} = 4 \cdot d$

For Douglas fir members minimum spacing and distances parallel to the grain shall be increased by 50%.

Minimum distances from the unloaded edge perpendicular to the grain may be reduced to 3·d also for timber thickness $t < 5 \cdot d$, if the spacing parallel to the grain and the end distance is at least 25·d.

These requirements do not apply for structural wood-based panels or LVL with cross layers.

Exclusively axially loaded screws or threaded rods
Minimum distances and spacing for exclusively axially loaded screws in predrilled and non-predrilled holes and for threaded rods in predrilled holes in members with a minimum thickness t = 12·d and a minimum width of 8·d or 60 mm, whichever is the greater, may be taken as:

Spacing a_1 parallel to the grain $a_1 = 5 \cdot d$
Spacing a_2 perpendicular to the grain $a_2 = 5 \cdot d$
Distance $a_{1,CG}$ from centre of the screw-part in timber to the end grain $a_{1,CG} = 10 \cdot d$
Distance $a_{2,CG}$ from centre of the screw-part in timber to the edge $a_{2,CG} = 4 \cdot d$

Spacing a_2 perpendicular to the grain may be reduced from 5·d to 2,5·d, if the condition $a_1 \cdot a_2 \geq 25 \cdot d^2$ is fulfilled.

Minimum distances and spacing for inclined or crossed "VGZ" screws d = 7 mm in joist-to-header connections arranged under 45° to the joist's end grain surface with a minimum joist depth of 18·d may be taken as:

Distance $a_{1,CG}$ from centre of the screw-part in timber to the unloaded joist end $a_{1,CG} = 8 \cdot d$
Distance $a_{2,CG}$ from centre of the screw-part in timber to the unloaded joist edge $a_{2,CG} = 3 \cdot d$

For a crossed screw or threaded rod couple the minimum spacing between the crossing screws is $a_2 = 1{,}5 \cdot d$.

Minimum thickness for predrilled structural members is t = 24 mm for screws with outer thread diameter d < 8 mm, t = 30 mm for screws with outer thread diameter d = 8 mm, t = 40 mm for screws with outer thread diameter d ≥ 9 mm, t = 80 mm for screws with outer thread diameter d ≥ 11 mm and t = 100 mm for screws or threaded rods with outer thread diameter d ≥ 13 mm. These minimum thickness values generally apply for non-predrilled softwood members, if the spacing parallel to the grain and the end distance is at least 25·d.

In all other cases, minimum thicknesses for screws in non-predrilled softwood members are given in EN 1995-1-1 (Eurocode 5) clause 8.3.1.2 as for nails in non-predrilled holes. Equation (8.18) may be applied for softwood members made of pine or for the fixing of boards, battens or wind braces, if the member is fixed with at least two screws. Otherwise EN 1995-1-1 clause 8.3.1.2 (7) applies.

Cross Laminated Timber

Unless specified otherwise in the technical specification (ETA or hEN) of cross laminated timber, minimum distances and spacing for screws or threaded rods in the wide face of cross laminated timber members with a minimum thickness t = 10·d may be taken as (see Annex B):

Spacing a_1 parallel to the grain $a_1 = 4 \cdot d$
Spacing a_2 perpendicular to the grain $a_2 = 2{,}5 \cdot d$
Distance $a_{3,c}$ from centre of the screw-part in timber to the unloaded end grain $a_{3,c} = 6 \cdot d$
Distance $a_{3,t}$ from centre of the screw-part in timber to the loaded end grain $a_{3,t} = 6 \cdot d$
Distance $a_{4,c}$ from centre of the screw-part in timber to the unloaded edge $a_{4,c} = 2{,}5 \cdot d$
Distance $a_{4,t}$ from centre of the screw-part in timber to the loaded edge $a_{4,t} = 6 \cdot d$

Unless specified otherwise in the technical specification (ETA or hEN) of cross laminated timber, minimum distances and spacing for screws or threaded rods in the edge surface of cross laminated timber members with a minimum thickness t = 10·d and a minimum penetration depth perpendicular to the edge surface of 10·d may be taken as (see Annex B):

Spacing a_1 parallel to the CLT plane $a_1 = 10 \cdot d$
Spacing a_2 perpendicular to the CLT plane $a_2 = 4 \cdot d$
Distance $a_{3,c}$ from centre of the screw-part in timber to the unloaded end $a_{3,c} = 7 \cdot d$
Distance $a_{3,t}$ from centre of the screw-part in timber to the loaded end $a_{3,t} = 12 \cdot d$
Distance $a_{4,c}$ from centre of the screw-part in timber to the unloaded edge $a_{4,c} = 3 \cdot d$
Distance $a_{4,t}$ from centre of the screw-part in timber to the loaded edge $a_{4,t} = 6 \cdot d$

Laminated Veneer Lumber

Unless specified otherwise in the technical specification (ETA or hEN) of softwood LVL, minimum distances and spacing for "HBS", "HBSH", "HBSP", "SHS" and "TBS" screws 5 mm ≤ d ≤ 10 mm perpendicular to the wide face of softwood LVL members with a minimum side member thickness $t_1 = 8{,}4 \cdot d - 9$ mm and a minimum central member thickness in double shear connections $t_2 = \min\{11{,}4 \cdot d; 75 \text{ mm}\}$ may be taken as:

Spacing a_1 parallel to the grain $a_1 = (5 + 7|\cos\alpha|)d$
Spacing a_2 perpendicular to the grain $a_2 = 5 \cdot d$
Distance $a_{3,c}$ to the unloaded end grain $a_{3,c} = 10 \cdot d$
Distance $a_{3,t}$ to the loaded end grain $a_{3,t} = (10 + 5\cos\alpha)d$
Distance $a_{4,c}$ to the unloaded edge $a_{4,c} = 5 \cdot d$
Distance $a_{4,t}$ to the loaded edge $a_{4,t} = (5 + 5\sin\alpha)d$

Unless specified otherwise in the technical specification (ETA or hEN) of softwood LVL, minimum distances and spacing for exclusively axially loaded screws "VGZ", "VGZH" "VGS" or "VGSH" with d = 7 mm or d = 9 mm in the edge surface of softwood LVL members with a minimum LVL panel thickness t = 45 mm (d = 6 mm and d = 7 mm) or t = 57 mm (d = 8 mm and d = 9 mm) and a minimum LVL member depth h = 100 mm (d = 6 mm and d = 7 mm) or h = 120 mm (d = 8 mm and d = 9 mm) may be taken as:

Spacing a_1 parallel to the grain $a_1 = 10 \cdot d$
Distance $a_{1,CG}$ from centre of the screw-part in timber to the unloaded end $a_{1,CG} = 12 \cdot d$
Distance $a_{2,CG}$ from centre of the screw-part in timber to the unloaded edge $a_{2,CG} = 3 \cdot d$

4 Attestation and verification of constancy of performance (AVCP)

4.1 AVCP system

According to the decision 97/176/EC of the European Commission1, as amended, the system(s) of assessment and verification of constancy of performance (see Annex V to Regulation (EU) No 305/2011) is 3.

5 Technical details necessary for the implementation of the AVCP system, as foreseen in the applicable EAD

Technical details necessary for the implementation of the AVCP system are laid down in the control plan deposited at ETA-Danmark prior to CE marking.

Issued in Copenhagen on 2020-12-10 by

Thomas Bruun
Managing Director, ETA-Danmark

Annex A
Drawings of Rotho Blaas screws

Annex A.01 | **Rotho Blaas screws** | rothoblaas

CARBON STEEL
PARTIAL THREAD
Ø 3.0 mm

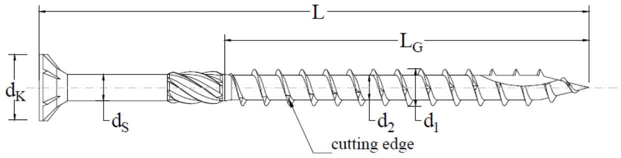

Alternative names:

HBS/SCH/SNK/GHS

Headstamps (supplier head mark and specific lenght) optional.

Alternative head types:

countersunk head
with or without milling ribs under head

"CS"

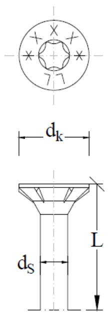

**d_1**	**3.00**
d_K	6.00 ±0.50

Alternative thread tip types:

Secondary rough thread optional.

"RBN"
with or without cutting edge

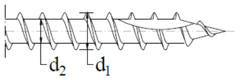

"RBN2"
with or without cutting edge

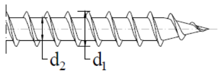

	**d_1**	d_2	d_s
**"RBN"**	**3.00** ±0.30	2.00 ±0.30	2.16 ±0.30
**"RBN2"**	**3.00** ±0.30	2.00 ±0.30	2.16 ±0.30

Lenghts and Thread Lenghts

**d_1**	L		L_G	
	min	max	min	max
**3.00**	20.0	40.0	12.0	35.0

Tolerance (L and L_G): according to EAD 130118-01-0603.
Intermediate lengths (L) are possible.
Intermediate thread lengths (L_G) are possible.

All dimensions in [mm].

Annex A.02	Rotho Blaas screws	rothoblaas

CARBON STEEL
PARTIAL THREAD
Ø 3.5 mm

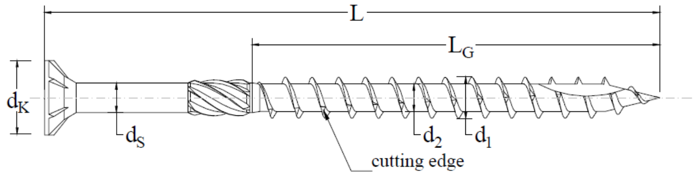

Alternative names:

HBS/SCH/SNK/GHS

Alternative head types:

Headstamps (supplier head mark and specific lenght) optional.

countersunk head
with or without milling ribs under head

"CS"

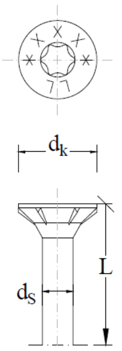

d_1	3.50
d_K	7.00 ±0.50

Alternative thread tip types:

Secondary rough thread optional.

"RBN"
with or without cutting edge

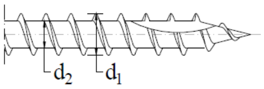

"RBN2"
with or without cutting edge

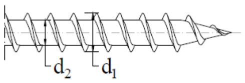

	d_1	d_2	d_s
"RBN"	3.50 ±0.30	2.25 ±0.30	2.45 ±0.30
"RBN2"	3.50 ±0.30	2.25 ±0.30	2.45 ±0.30

Lenghts and Thread Lenghts

d_1	L		L_G	
	min	max	min	max
3.50	20.0	50.0	14.0	24.0

Tolerance (L and L_G): according to EAD 130118-01-0603.
Intermediate lengths (L) are possible.
Intermediate thread lengths (L_G) are possible.

All dimensions in [mm].

CARBON STEEL
PARTIAL THREAD
Ø 4.0 mm

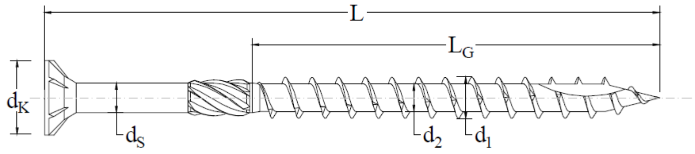

Alternative names:

HBS/SCH/SNK/GHS
HBSP/KGL/GHS+

Headstamps (supplier head mark and specific lenght) optional.

Alternative head types:

countersunk head with or without milling ribs under head **"CS"**	washer head with cylindrical underhead with or without milling ribs under head **"WU1"**
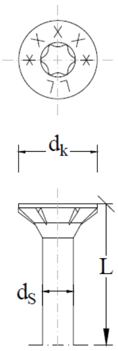	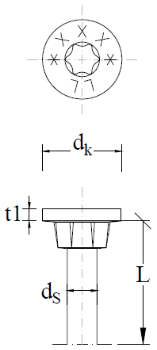

**d_1**	**4.00**
d_K	8.00 ±0.60

**d_1**	**4.00**
d_K	7.70 ±0.50
t_1	1.00 ±0.30

Alternative thread tip types:

Secondary rough thread optional.

**"RBN"** with or without cutting edge	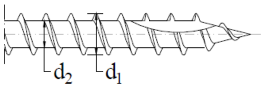
**"RBN2"** with or without cutting edge	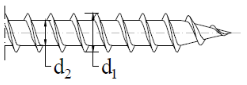

	**d_1**	d_2	d_s
**"RBN"**	**4.00** ±0.30	2.55 ±0.30	2.75 ±0.30
**"RBN2"**	**4.00** ±0.30	2.55 ±0.30	2.75 ±0.30

Lenghts and Thread Lenghts

**d_1**	L		L_G	
	min	max	min	max
**4.00**	25.0	80.0	16.0	40.0

Tolerance (L and L_G): according to EAD 130118-01-0603.
Intermediate lengths (L) are possible.
Intermediate thread lengths (L_G) are possible.

All dimensions in [mm].

Annex A.04 **Rotho Blaas screws** rothoblaas

CARBON STEEL
PARTIAL THREAD
Ø 4.5 mm

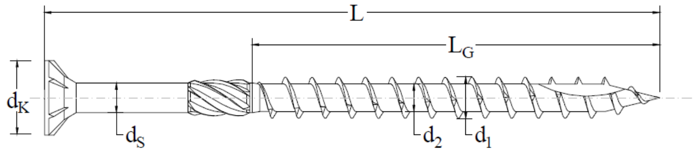

Alternative names:

HBS/SCH/SNK/GHS
HBSP/KGL/GHS+

Headstamps (supplier head mark and specific lenght) optional.

Alternative head types:

countersunk head with or without milling ribs under head **"CS"**	washer head with cylindrical underhead with or without milling ribs under head **"WU1"**
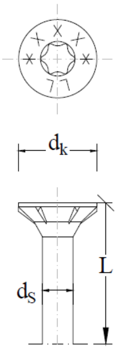	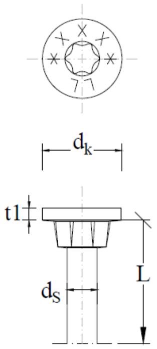

**d_1**	**4.50**
d_K	9.00 ±0.60

**d_1**	**4.50**
d_K	8.70 ±0.60
t_1	1.00 ±0.30

Alternative thread tip types:

**"RBN"** with or without cutting edge	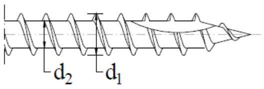
**"RBN2"** with or without cutting edge	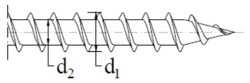

Secondary rough thread optional.

	**d_1**	d_2	d_s
**"RBN"**	**4.50** ±0.30	2.80 ±0.30	3.15 ±0.30
**"RBN2"**	**4.50** ±0.30	2.80 ±0.30	3.15 ±0.30

Lenghts and Thread Lenghts

**d_1**	L		L_G	
	min	max	min	max
**4.50**	25.0	80.0	18.0	40.0

Tolerance (L and L_G): according to EAD 130118-01-0603.
Intermediate lengths (L) are possible.
Intermediate thread lengths (L_G) are possible.

All dimensions in [mm].

Annex A.05 | **Rotho Blaas screws** | rothoblaas

CARBON STEEL
PARTIAL THREAD
Ø 5.0 mm

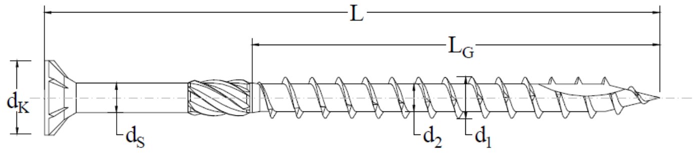

Alternative names:

HBS/SCH/SNK/GHS
HBSP/KGL/GHS+
SHS

Alternative head types:

Headstamps (supplier head mark and specific lenght) optional.

countersunk head with or without milling ribs under head **"CS"**	washer head with cylindrical underhead with or without milling ribs under head **"WU1"**	countersunk head 60° with or without milling ribs under head **"CS60"**
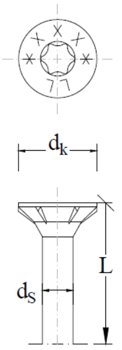	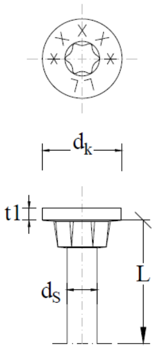	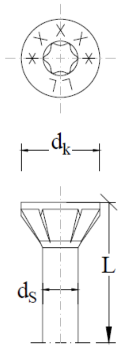

**d_1**	**5.00**
d_K	10.00 ±0.60

**d_1**	**5.00**
d_K	9.65 ±0.60
t_1	1.00 ±0.30

**d_1**	**5.00**
d_K	10.00 ±0.60

Alternative thread tip types:

**"RBN"** with or without cutting edge	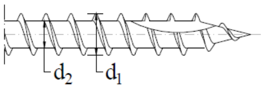
**"RBN2"** with or without cutting edge	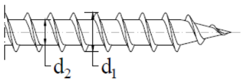

Secondary rough thread optional.

	**d_1**	d_2	d_s
**"RBN"**	**5.00** ±0.30	3.40 ±0.30	3.65 ±0.30
**"RBN2"**	**5.00** ±0.30	3.40 ±0.30	3.65 ±0.30

Lenghts and Thread Lenghts

**d_1**	L		L_G	
	min	max	min	max
**5.00**	30.0	120.0	20.0	60.0

Tolerance (L and L_G): according to EAD 130118-01-0603.
Intermediate lengths (L) are possible.
Intermediate thread lengths (L_G) are possible.

All dimensions in [mm].

Annex A.06 | **Rotho Blaas screws** | rothoblaas

CARBON STEEL
PARTIAL THREAD
Ø 6.0 mm

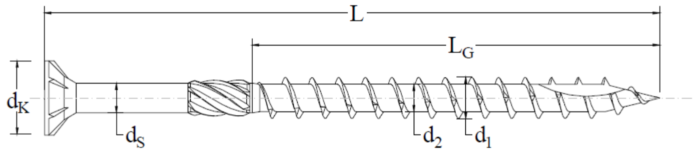

Alternative names:

HBS/SCH/SNK/GHS
HBSP/KGL/GHS+
TBS/TLL/GHSK
HBSH

Alternative head types:

Headstamps (supplier head mark and specific lenght) optional.

countersunk head with or without milling ribs under head	large washer head	large washer head	washer head with cylindrical underhead with or without milling ribs under head	countersunk head 60° with or without milling ribs under head
**"CS"**	**"LW1"**	**"LW2"**	**"WU1"**	**"CS60"**

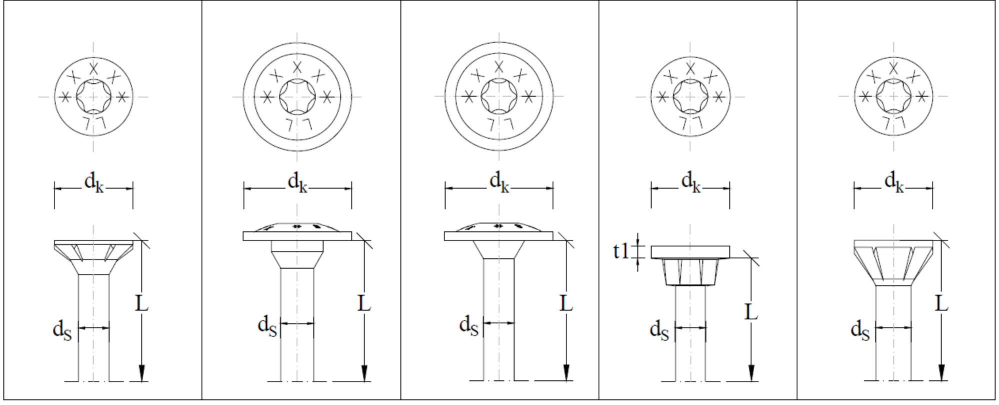

"CS":

d_1	**6.00**
d_K	12.00 ±0.60

"LW1":

d_1	**6.00**
d_K	15.50 ±0.78

"LW2":

d_1	**6.00**
d_K	15.50 ±0.78

"WU1":

d_1	**6.00**
d_K	12.00 ±0.60
t_1	1.50 ±0.30

"CS60":

d_1	**6.00**
d_K	12.00 ±0.60

Alternative thread tip types:

"RBN"
with or without
cutting edge

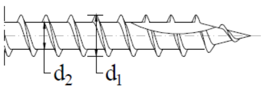

"RBN2"
with or without
cutting edge

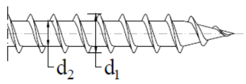

"RBH"
with or without cutting edge
with or wirhout cut with or
without saw teeth

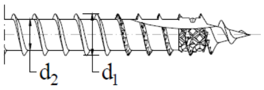

Secondary rough thread optional.

	d_1	d_2	d_s
**"RBN"**	**6.00** ±0.30	3.95 ±0.30	4.30 ±0.30
**"RBN2"**	**6.00** ±0.30	3.95 ±0.30	4.30 ±0.30
**"RBH"**	**6.00** ±0.30	4.50 ±0.30	4.80 ±0.30

Lenghts and Thread Lenghts

d_1	L		L_G		Thread tip types
	min	max	min	max	
**6.00**	40.0	300.0	24.0	100.0	RBN - RBN2
**6.00**	80.0	160.0	24.0	90.0	RBH

Tolerance (L and L_G): according to EAD 130118-01-0603.
Intermediate lengths (L) are possible.
Intermediate thread lengths (L_G) are possible.

All dimensions in [mm].

Annex A.07a **Rotho Blaas screws** rothoblaas

CARBON STEEL
PARTIAL THREAD
Ø 8.0 mm

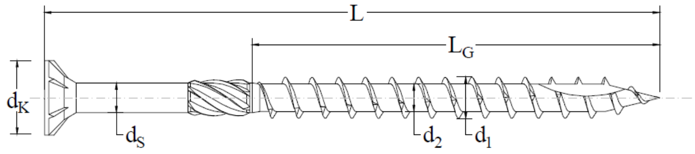

Alternative names:

HBS/SCH/SNK/GHS
HBSP/KGL/GHS+
TBS/TLL/GHSK
HBSH

Alternative thread tip types:

**"RBN"** with or without cutting edge	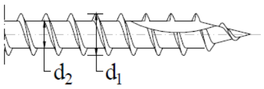
**"RBN2"** with or without cutting edge	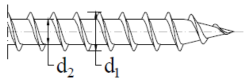
**"RBH"** with or without cutting edge with or wirhout cut with or without saw teeth	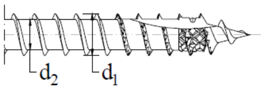

Secondary rough thread optional.

	d_1	d_2	d_s
**"RBN"**	**8.00** ±0.40	5.40 ±0.30	5.80 ±0.30
**"RBN2"**	**8.00** ±0.40	5.40 ±0.30	5.80 ±0.30
**"RBH"**	**8.00** ±0.40	5.90 ±0.30	6.30 ±0.32

Lenghts and Thread Lenghts

d_1	L		L_G		Thread tip types
	min	max	min	max	
**8.00**	40.0	520.0	32.0	130.0	RBN - RBN2
**8.00**	120.0	240.0	32.0	100.0	RBH

Tolerance (L and L_G): according to EAD 130118-01-0603.
Intermediate lengths (L) are possible.
Intermediate thread lengths (L_G) are possible.

All dimensions in [mm].

CARBON STEEL
PARTIAL THREAD
Ø 8.0 mm

Alternative head types:

Headstamps (supplier head mark and specific lenght) optional.

countersunk head with or without milling ribs under head	large washer head	large washer head	large washer head	large washer head	large washer head
**"CS"**	**"LW1"**	**"LW1"**	**"LW2"**	**"LW2"**	**"LW3"**

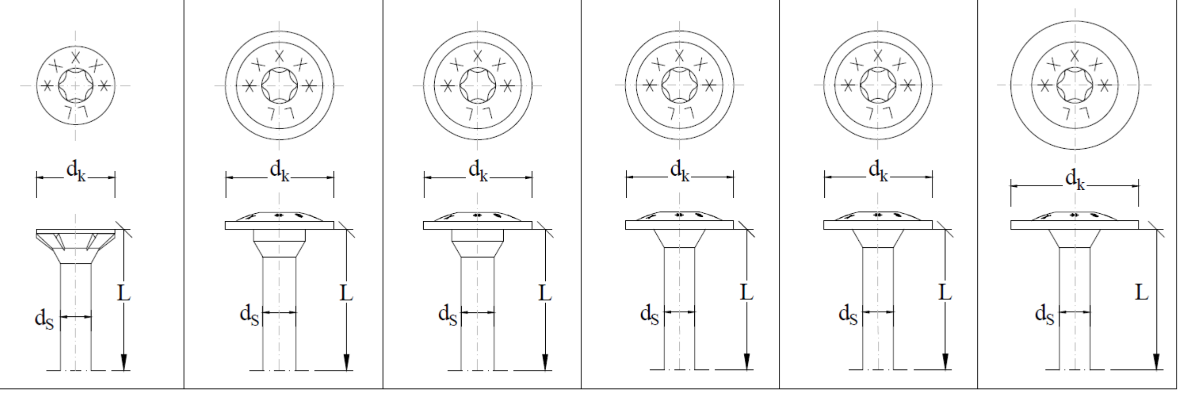

	"CS"	"LW1"	"LW1"	"LW2"	"LW2"	"LW3"
d_1	**8.00**	**8.00**	**8.00**	**8.00**	**8.00**	**8.00**
d_K	14.50 ±0.73	19.00 ±0.95	22.00 ±1.10	19.00 ±0.95	22.00 ±1.10	24.50 ±1.23

washer head with cylindrical underhead with or without milling ribs under head	washer head with cylindrical underhead with or without milling ribs under head	countersunk head 60° with or without milling ribs under head
**"WU1"**	**"WU2"**	**"CS60"**

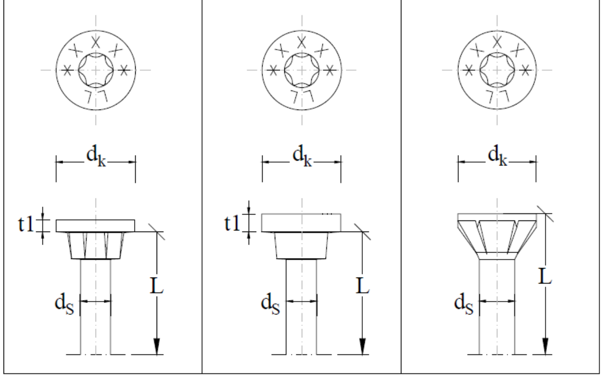

	"WU1"	"WU2"	"CS60"
d_1	**8.00**	**8.00**	**8.00**
d_K	14.50 ±0.73	14.50 ±0.73	14.50 ±0.73
t_1	1.75 ±0.30	3.40 ±0.30	

All dimensions in [mm].

Annex A.08 **Rotho Blaas screws** rothoblaas

CARBON STEEL
PARTIAL THREAD
Ø 10.0 mm

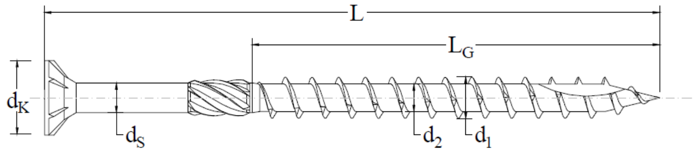

Alternative names:

HBS/SCH/SNK/GHS
HBSP/KGL/GHS+
TBS/TLL/GHSK

Alternative head types:

Headstamps (supplier head mark and specific lenght) optional.

countersunk head with or without milling ribs under head **"CS"**	large washer head **"LW1"**	large washer head **"LW2"**	washer head with cylindrical underhead with or without milling ribs under head **"WU1"**	washer head with cylindrical underhead with or without milling ribs under head **"WU2"**

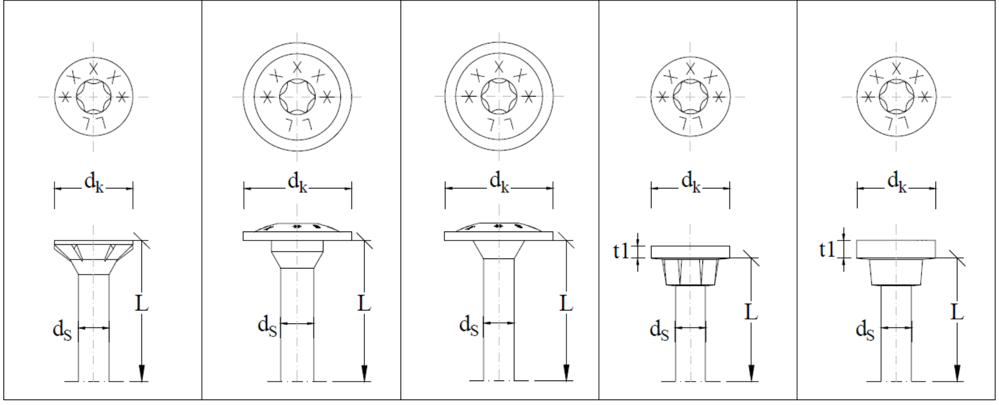

"CS":

**d_1**	**10.00**
d_K	18.25 ±0.91

"LW1":

**d_1**	**10.00**
d_K	25.00 ±1.25

"LW2":

**d_1**	**10.00**
d_K	25.00 ±1.25

"WU1":

**d_1**	**10.00**
d_K	18.25 ±0.91
t_1	2.00 ±0.30

"WU2":

**d_1**	**10.00**
d_K	18.25 ±0.91
t_1	4.35 ±0.30

Alternative thread tip types:

"RBN"
with or without cutting edge

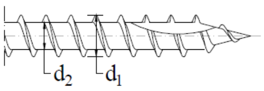

"RBN2"
with or without cutting edge

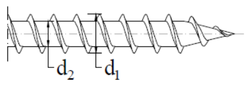

Secondary rough thread optional.

	**d_1**	d_2	d_s
**"RBN"**	**10.00** ±0.50	6.40 ±0.32	7.00 ±0.35
**"RBN2"**	**10.00** ±0.50	6.40 ±0.32	7.00 ±0.35

Lenghts and Thread Lenghts

**d_1**	L		L_G	
	min	max	min	max
**10.00**	60.0	520.0	40.0	150.0

Tolerance (L and L_G): according to EAD 130118-01-0603.
Intermediate lengths (L) are possible.
Intermediate thread lengths (L_G) are possible.

All dimensions in [mm].

Annex A.09 **Rotho Blaas screws** rothoblaas

CARBON STEEL
PARTIAL THREAD
Ø 12.0 mm

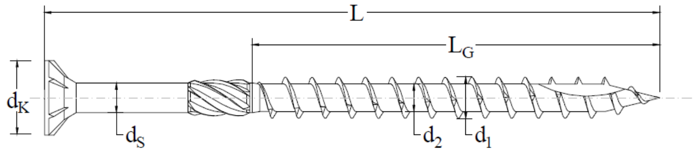

Alternative names:

HBS/SCH/SNK/GHS
HBSP/KGL/GHS+

Alternative head types:

Headstamps (supplier head mark and specific lenght) optional.

countersunk head with or without milling ribs under head **"CS"**	washer head with cylindrical underhead with or without milling ribs under head **"WU1"**	washer head with cylindrical underhead with or without milling ribs under head **"WU2"**
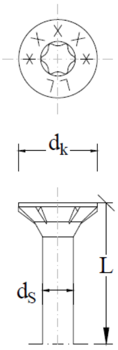	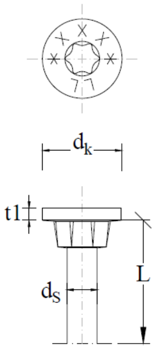	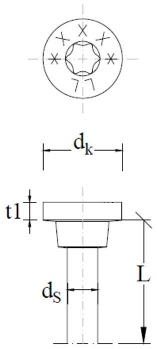

**d_1**	**12.00**
d_K	20.75 ±1.04

**d_1**	**12.00**
d_K	20.75 ±1.04
t_1	2.30 ±0.30

**d_1**	**12.00**
d_K	20.75 ±1.04
t_1	5.00 ±0.30

Alternative thread tip types:

**"RBN"** with or without cutting edge	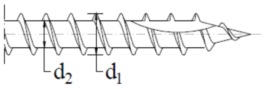
**"RBN2"** with or without cutting edge	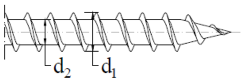

Secondary rough thread optional.

	**d_1**	d_2	d_s
**"RBN"**	**12.00** ±0.60	6.80 ±0.34	8.00 ±0.40
**"RBN2"**	**12.00** ±0.60	6.80 ±0.34	8.00 ±0.40

Lenghts and Thread Lenghts

**d_1**	L		L_G	
	min	max	min	max
**12.00**	120.0	600.0	48.0	160.0

Tolerance (L and L_G): according to EAD 130118-01-0603.
Intermediate lengths (L) are possible.
Intermediate thread lengths (L_G) are possible.

All dimensions in [mm].

Annex A.10	Rotho Blaas screws	rothoblaas

CARBON STEEL
SCREWS FOR NAILING PLATES
Ø 5.0 mm

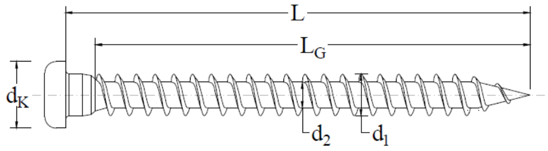

Alternative names:

LBS

Alternative head types:

Headstamps (supplier head mark and specific lenght) optional.

round head with cylindrical underhead "RU"

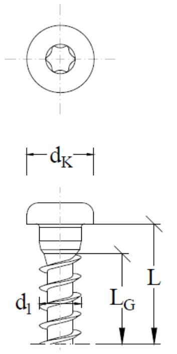

**d_1**	**5.00**
d_K	7.80 ±0.50

Alternative thread tip types:

"RBP"
with or without cutting edge
with or wirhout cut

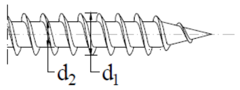

	**d_1**	d_2
**"RBP"**	**5.00** +0.00/-0.30	3.00 ±0.30

Lenghts and Thread Lenghts

**d_1**	L		L_G	
	min	max	min	max
**5.00**	25.0	70.0	20.0	66.0

Tolerance (L and L_G): according to EAD 130118-01-0603.
Intermediate lengths (L) are possible.
Intermediate thread lengths (L_G) are possible.

All dimensions in [mm].

Annex A.11 | **Rotho Blaas screws** | rothoblaas

CARBON STEEL
SCREWS FOR NAILING PLATES
Ø 7.0 mm

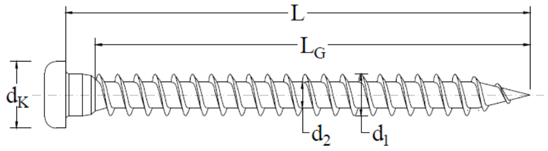

Alternative names:

LBS

Alternative head types:

Headstamps (supplier head mark and specific lenght) optional.

round head
with cylindrical
underhead

"RU"

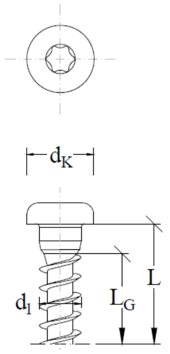

d_1	**7.00**
d_K	11.00<br>±0.60

Alternative thread tip types:

"RBP"
with or without cutting edge
with or wirhout cut

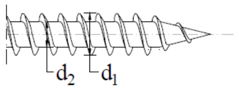

	d_1	d_2
**"RBP"**	**7.00**<br>±0.35	4.40<br>±0.30

Lenghts and Thread Lenghts

d_1	L		L_G	
	min	max	min	max
**7.00**	50.0	100.0	28.0	95.0

Tolerance (L and L_G): according to EAD 130118-01-0603.
Intermediate lengths (L) are possible.
Intermediate thread lengths (L_G) are possible.

All dimensions in [mm].

Annex A.12 | **Rotho Blaas screws** | rothoblaas

CARBON STEEL
FULL THREAD
Ø 5.3 mm

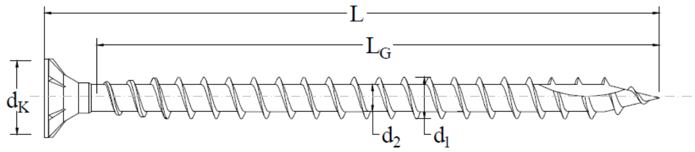

Alternative names:

VGZ/GWZ
VGS/GWS

Alternative head types:

Headstamps (supplier head mark and specific lenght) optional.

countersunk head<br>with or without milling ribs under head<br>**"CS"**	cylindrical head<br>**"CY"**
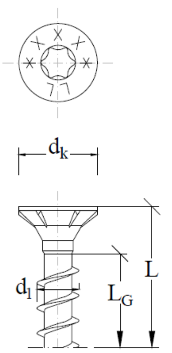	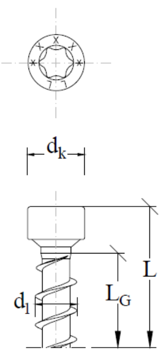

d_1	**5.30**
d_K	11.00 ±0.60

d_1	**5.30**
d_K	8.00 ±0.60

Alternative thread tip types:

**"RBN"**<br>with or without cutting edge	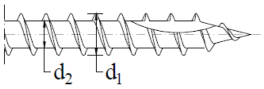
**"RBN2"**<br>with or without cutting edge	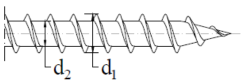

	d_1	d_2
**"RBN"**	**5.30** ±0.30	3.60 ±0.30
**"RBN2"**	**5.30** ±0.30	3.60 ±0.30

Lenghts and Thread Lenghts

d_1	L		L_G	
	min	max	min	max
**5.30**	60.0	120.0	50.0	110.0

Tolerance (L and L_G): according to EAD 130118-01-0603.
Intermediate lengths (L) are possible.
Intermediate thread lengths (L_G) are possible.

All dimensions in [mm].

Annex A.13 **Rotho Blaas screws** rothoblaas

CARBON STEEL
FULL THREAD
Ø 5.6 mm

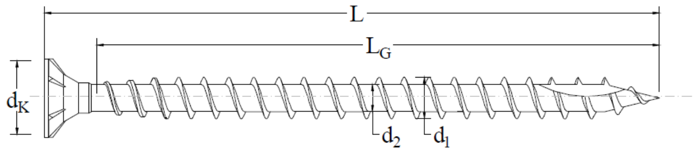

Alternative names:

VGZ/GWZ
VGS/GWS

Alternative head types:

Headstamps (supplier head mark and specific lenght) optional.

countersunk head with or without milling ribs under head "CS"	cylindrical head "CY"
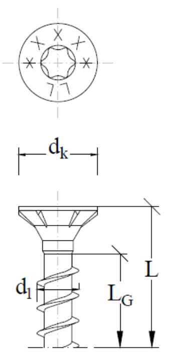	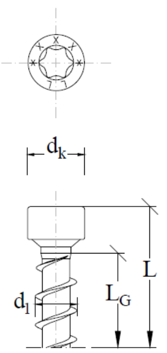

**d_1**	**5.60**
d_K	11.00 ±0.60

**d_1**	**5.60**
d_K	8.00 ±0.60

Alternative thread tip types:

**"RBN"** with or without cutting edge	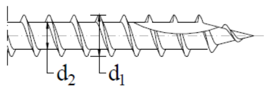
**"RBN2"** with or without cutting edge	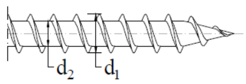

	**d_1**	d_2
**"RBN"**	**5.60** ±0.30	3.80 ±0.30
**"RBN2"**	**5.60** ±0.30	3.80 ±0.30

Lenghts and Thread Lenghts

**d_1**	L		L_G	
	min	max	min	max
**5.60**	60.0	160.0	50.0	150.0

Tolerance (L and L_G): according to EAD 130118-01-0603.
Intermediate lengths (L) are possible.
Intermediate thread lengths (L_G) are possible.

All dimensions in [mm].

Annex A.14 **Rotho Blaas screws** rothoblaas

CARBON STEEL
FULL THREAD
Ø 7.0 mm

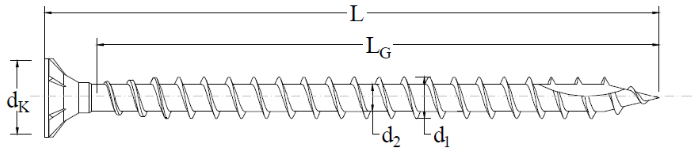

Alternative names:

VGZ/GWZ
VGS/GWS

Alternative head types:

Headstamps (supplier head mark and specific lenght) optional.

countersunk head with or without milling ribs under head **"CS"**	cylindrical head **"CY"**
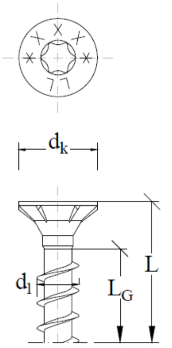	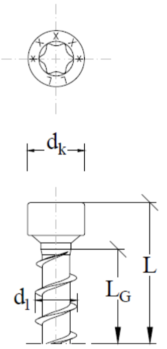

d_1	7.00
d_K	13.00 ±0.65

d_1	7.00
d_K	9.50 ±0.60

Alternative thread tip types:

**"RBN"** with or without cutting edge	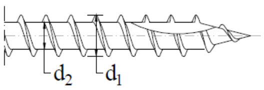
**"RBN2"** with or without cutting edge	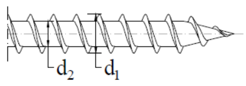

	d_1	d_2
**"RBN"**	**7.00** ±0.35	4.60 ±0.30
**"RBN2"**	**7.00** ±0.35	4.60 ±0.30

Lenghts and Thread Lenghts

d_1	L		L_G	
	min	max	min	max
**7.00**	60.0	400.0	50.0	390.0

Tolerance (L and L_G): according to EAD 130118-01-0603.
Intermediate lengths (L) are possible.
Intermediate thread lengths (L_G) are possible.

All dimensions in [mm].

Annex A.15 | **Rotho Blaas screws** | rothoblaas

CARBON STEEL
FULL THREAD
Ø 9.0 mm

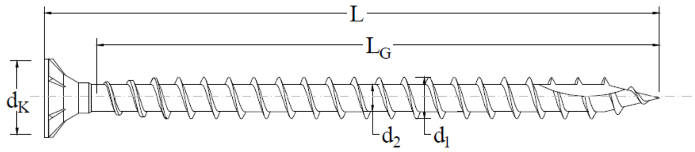

Alternative names:

VGZ/GWZ
VGS/GWS

Alternative head types:

Headstamps (supplier head mark and specific lenght) optional.

countersunk head with or without milling ribs under head **"CS"**	cylindrical head **"CY"**
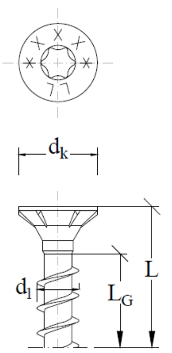	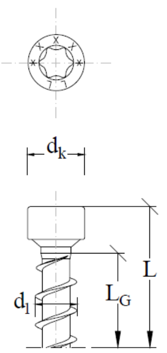

**d_1**	**9.00**
d_K	16.00 ±0.80

**d_1**	**9.00**
d_K	11.50 ±0.60

Alternative thread tip types:

**"RBN"** with or without cutting edge	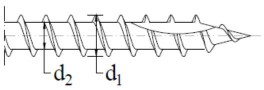
**"RBN2"** with or without cutting edge	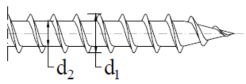

	**d_1**	d_2
**"RBN"**	**9.00** ±0.45	5.90 ±0.30
**"RBN2"**	**9.00** ±0.45	5.90 ±0.30

Lenghts and Thread Lenghts

**d_1**	L		L_G	
	min	max	min	max
**9.00**	100.0	520.0	90.0	510.0

Tolerance (L and L_G): according to EAD 130118-01-0603.
Intermediate lengths (L) are possible.
Intermediate thread lengths (L_G) are possible.

All dimensions in [mm].

Annex A.16 **Rotho Blaas screws** rothoblaas

CARBON STEEL
FULL THREAD
Ø 11.0 mm

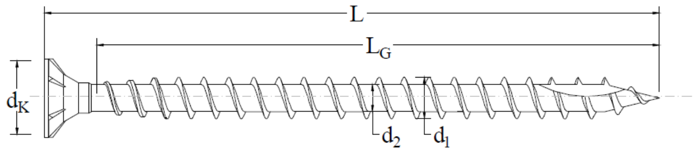

Alternative names:

VGZ/GWZ
VGS/GWS

Alternative head types:

Headstamps (supplier head mark and specific lenght) optional.

countersunk head with or without milling ribs under head "CS"	cylindrical head "CY"	hexagonal torx head "EXA"
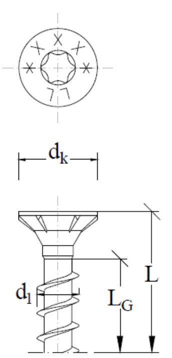	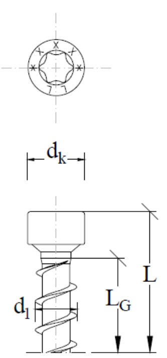	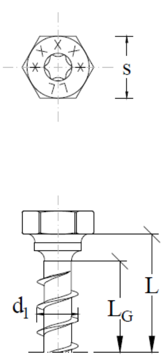

d_1	11.00
d_K	19.30 ±0.97

d_1	11.00
d_K	13.50 ±0.68

d_1	11.00
s	17.00 +0.00/-0.43

Alternative thread tip types:

**"RBN"** with or without cutting edge	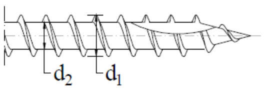
**"RBN2"** with or without cutting edge	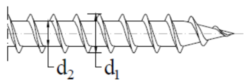
**"RBSN"** with or without cutting edge with or wirhout cut	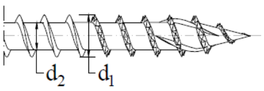

	d_1	d_2
**"RBN"**	**11.00** ±0.55	6.60 ±0.33
**"RBN2"**	**11.00** ±0.55	6.60 ±0.33
**"RBSN"**	**11.00** ±0.55	6.60 ±0.[illegible]0

Lenghts and Thread Lenghts

d_1	L		L_G	
	min	max	min	max
**11.00**	100.0	600.0	80.0	590.0

Tolerance (L and L_G): according to EAD 130118-01-0603.
Intermediate lengths (L) are possible.
Intermediate thread lengths (L_G) are possible.

All dimensions in [mm].

Annex A.17 **Rotho Blaas screws** rothoblaas

CARBON STEEL
FULL THREAD
Ø 13.0 mm

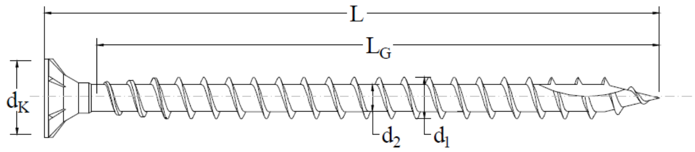

Alternative names:

VGZ/GWZ
VGS/GWS

Headstamps (supplier head mark and specific lenght) optional.

Alternative head types:

countersunk head with or without milling ribs under head "CS"	cylindrical head "CY"	hexagonal torx head "EXA"

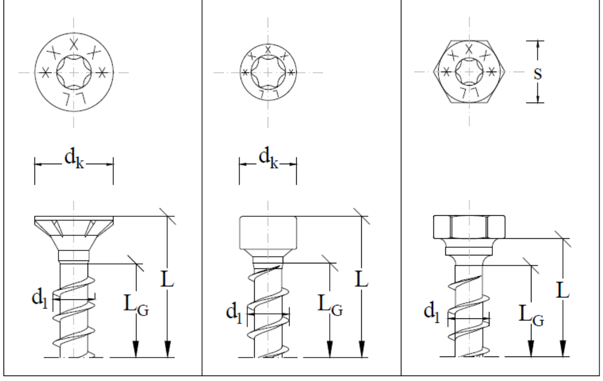

d_1	13.00
d_K	22.00 ±1.10

d_1	13.00
d_K	15.50 ±0.78

d_1	13.00
s	19.00 +0.00/-0.48

Alternative thread tip types:

"RBN"
with or without
cutting edge

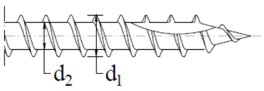

"RBN2"
with or without
cutting edge

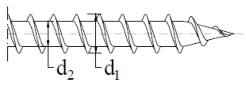

"RBSN"
with or without
cutting edge
with or wirhout cut

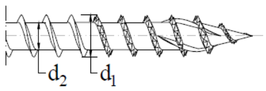

	d_1	d_2
"RBN"	13.00 ±0.65	8.00 ±0.40
"RBN2"	13.00 ±0.65	8.00 ±0.40
"RBSN"	13.00 ±0.65	8.00 ±0.40

Lenghts and Thread Lenghts

d_1	L		L_G	
	min	max	min	max
13.00	100.0	1200.0	80.0	1180.0

Tolerance (L and L_G): according to EAD 130118-01-0603.
Intermediate lengths (L) are possible.
Intermediate thread lengths (L_G) are possible.

All dimensions in [mm].

Annex A.18 **Rotho Blaas screws** rothoblaas

CARBON STEEL
FULL THREAD
Ø 6.0 mm

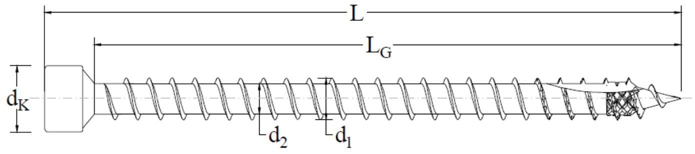

Alternative names:

VGZH / VGSH

Headstamps (supplier head mark and specific lenght) optional.

Alternative head types:

cylindrical head<br><br>**"CY"**	countersunk head 60°<br>with or without milling ribs under head<br>**"CS60"**
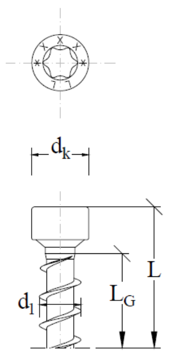	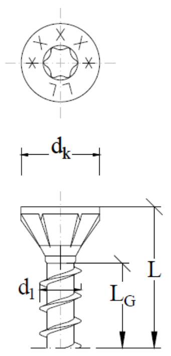

**d_1**	**6.00**
d_K	9.50 ±0.60

**d_1**	**6.00**
d_K	12.00 ±0.60

Alternative thread tip types:

"RBH"
with or without cutting edge
with or wirhout cut with or
without saw teeth

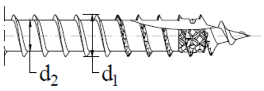

	**d_1**	d_2
**"RBH"**	**6.00** ±0.30	4.50 ±0.30

Lenghts and Thread Lenghts

**d_1**	L		L_G	
	min	max	min	max
**6.00**	80.0	260.0	70.0	250.0

Tolerance (L and L_G): according to EAD 130118-01-0603.
Intermediate lengths (L) are possible.
Intermediate thread lengths (L_G) are possible.

All dimensions in [mm].

Annex A.19 | **Rotho Blaas screws** | rothoblaas

CARBON STEEL
FULL THREAD
Ø 8.0 mm

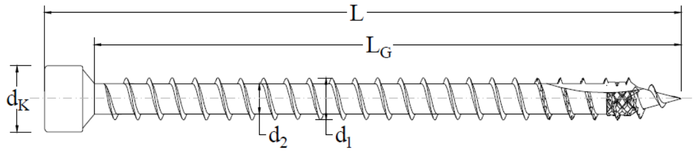

Alternative names:

VGZH / VGSH

Alternative head types:

Headstamps (supplier head mark and specific lenght) optional.

cylindrical head<br>**"CY"**	countersunk head 60°<br>with or without milling ribs under head<br>**"CS60"**
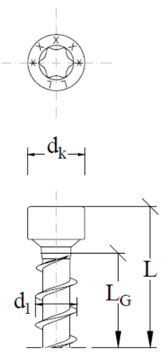	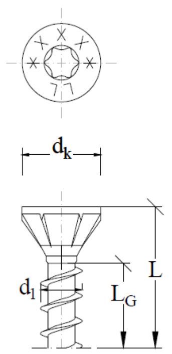

**d_1**	**8.00**
d_K	11.50<br>±0.60

**d_1**	**8.00**
d_K	14.50<br>±0.73

Alternative thread tip types:

"RBH"
with or without cutting edge
with or wirhout cut with or
without saw teeth

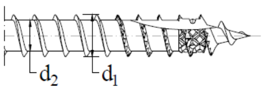

	**d_1**	d_2
**"RBH"**	**8.00**<br>±0.40	5.90<br>±0.30

Lenghts and Thread Lenghts

**d_1**	L		L_G	
	min	max	min	max
**8.00**	100.0	320.0	90.0	310.0

Tolerance (L and L_G): according to EAD 130118-01-0603.
Intermediate lengths (L) are possible.
Intermediate thread lengths (L_G) are possible.

All dimensions in [mm].

Annex A.20 **Rotho Blaas screws** rothoblaas

CARBON STEEL
DOUBLE THREAD
Ø 7.0 mm

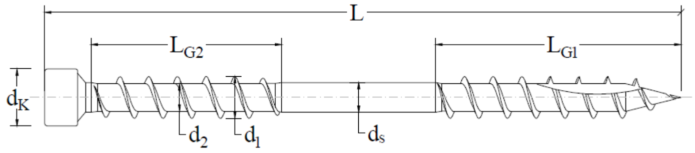

Alternative names:

DGZ/DWZ

Alternative head types:

Headstamps (supplier head mark and specific lenght) optional.

countersunk head with or without milling ribs under head "CS"	cylindrical head "CY"	large washer head "LW1"	large washer head "LW2"
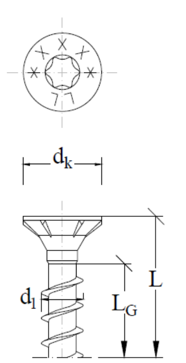	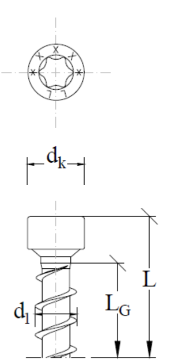	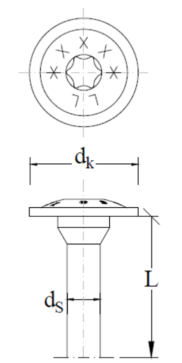	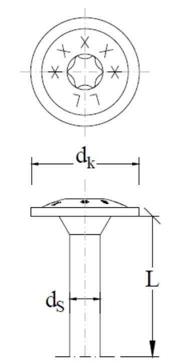

d_1	7.00
d_K	13.00 ±0.65

d_1	7.00
d_K	9.50 ±0.60

d_1	7.00
d_K	17.50 ±0.88

d_1	7.00
d_K	17.50 ±0.88

Alternative thread tip types:

**"RBN"** with or without cutting edge	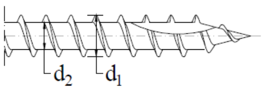
**"RBN2"** with or without cutting edge	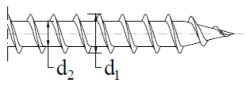

	d_1	d_2	d_s
**"RBN"**	**7.00** ±0.35	4.60 ±0.30	5.00 ±0.30
**"RBN2"**	**7.00** ±0.35	4.60 ±0.30	5.00 ±0.30

Lenghts and Thread Lenghts

d_1	L		L_{G1}		L_{G2}	
	min	max	min	max	min	max
**7.00**	220.0	400.0	40.0	100.0	40.0	80.0

Tolerance (L, L_{Gl} and L_{G2}): according to EAD 130118-01-0603.
Intermediate lengths (L) are possible.
Intermediate thread lengths (L_G) are possible.

All dimensions in [mm].

Annex A.21 **Rotho Blaas screws** rothoblaas

CARBON STEEL
DOUBLE THREAD
Ø 9.0 mm

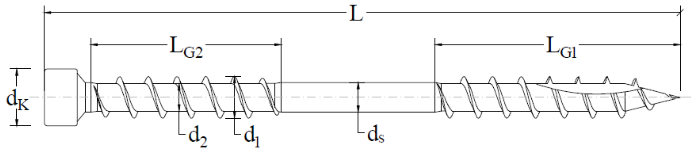

Alternative names:

DGZ/DWZ

Alternative head types:

Headstamps (supplier head mark and specific lenght) optional.

countersunk head with or without milling ribs under head "CS"	cylindrical head "CY"	large washer head "LW1"	large washer head "LW2"
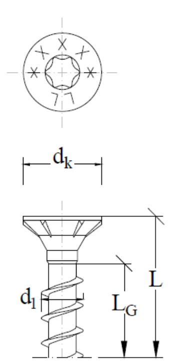	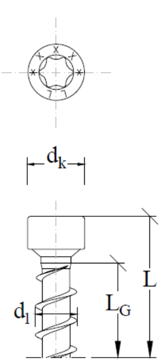	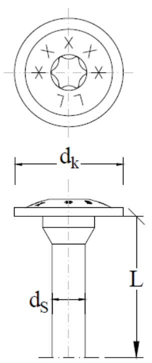	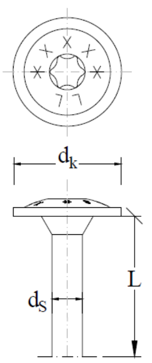

**d_1**	**9.00**
d_K	16.00 ±0.80

**d_1**	**9.00**
d_K	11.50 ±0.60

**d_1**	**9.00**
d_K	22.00 ±1.10

**d_1**	**9.00**
d_K	22.00 ±1.10

Alternative thread tip types:

**"RBN"** with or without cutting edge	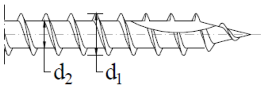
**"RBN2"** with or without cutting edge	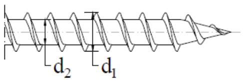

	d_1	d_2	d_s
**"RBN"**	**9.00** ±0.45	5.90 ±0.30	6.50 ±0.33
**"RBN2"**	**9.00** ±0.45	5.90 ±0.30	6.50 ±0.33

Lenghts and Thread Lenghts

d_1	L		L_{G1}		L_{G2}	
	min	max	min	max	min	max
**9.00**	220.0	520.0	40.0	100.0	40.0	80.0

Tolerance (L, L_{G1} and L_{G2}): according to EAD 130118-01-0603.
Intermediate lengths (L) are possible.
Intermediate thread lengths (L_G) are possible.

All dimensions in [mm].

STAINLESS STEEL
PARTIAL THREAD
Ø 3.5 mm

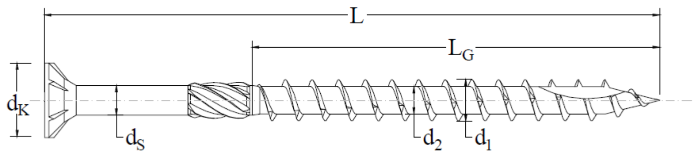

Alternative names:

SCI

Alternative head types:

Headstamps (supplier head mark and specific lenght) optional.

countersunk head
with or without milling ribs under head

"CS"

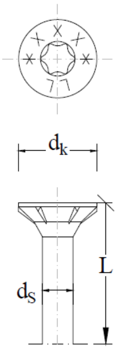

**d_1**	**3.50**
d_K	7.00 ±0.50

Alternative thread tip types:

Secondary rough thread optional.

"RBN"
with or without cutting edge

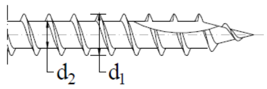

"RBN2"
with or without cutting edge

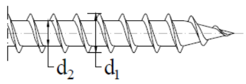

	d_1	d_2	d_s
**"RBN"**	**3.50** ±0.30	2.25 ±0.30	2.45 ±0.30
**"RBN2"**	**3.50** ±0.30	2.25 ±0.30	2.45 ±0.30

Lenghts and Thread Lenghts

d_1	L		L_G	
	min	max	min	max
**3.50**	20.0	50.0	14.0	24.0

Tolerance (L and L_G): according to EAD 130118-01-0603.
Intermediate lengths (L) are possible.
Intermediate thread lengths (L_G) are possible.

All dimensions in [mm].

Annex A.23 **Rotho Blaas screws** rothoblaas

STAINLESS STEEL
PARTIAL THREAD
Ø 4.0 mm

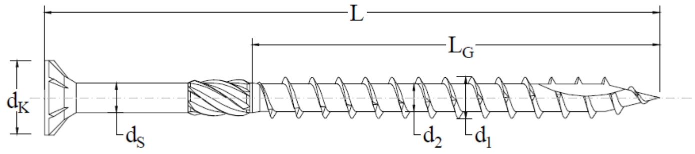

Alternative names:

SCI

Alternative head types:

Headstamps (supplier head mark and specific lenght) optional.

countersunk head
with or without milling ribs under head

"CS"

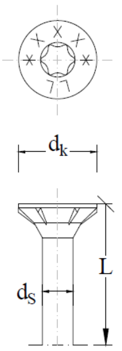

d_1	**4.00**
d_K	8.00 ±0.60

Alternative thread tip types:

Secondary rough thread optional.

"RBN"
with or without cutting edge

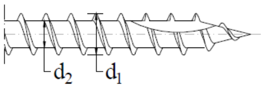

"RBN2"
with or without cutting edge

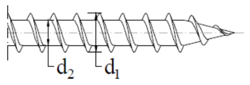

	d_1	d_2	d_s
**"RBN"**	**4.00** ±0.30	2.55 ±0.30	2.75 ±0.30
**"RBN2"**	**4.00** ±0.30	2.55 ±0.30	2.75 ±0.30

Lenghts and Thread Lenghts

d_1	L		L_G	
	min	max	min	max
**4.00**	20.0	60.0	16.0	40.0

Tolerance (L and L_G): according to EAD 130118-01-0603.
Intermediate lengths (L) are possible.
Intermediate thread lengths (L_G) are possible.

All dimensions in [mm].

Annex A.24 | **Rotho Blaas screws** | rothoblaas

STAINLESS STEEL
PARTIAL THREAD
Ø 4.5 mm

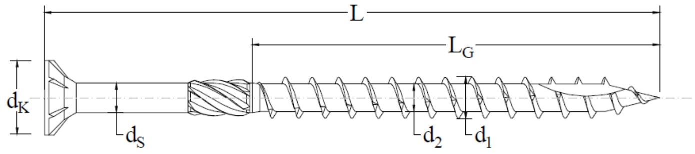

Alternative names:

SCI

Alternative head types:

Headstamps (supplier head mark and specific lenght) optional.

countersunk head
with or without milling ribs under head

"CS"

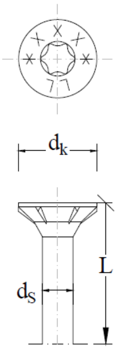

**d_1**	**4.50**
d_K	9.00 ±0.60

Alternative thread tip types:

Secondary rough thread optional.

"RBN"
with or without cutting edge

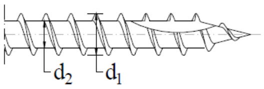

"RBN2"
with or without cutting edge

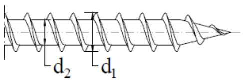

	**d_1**	d_2	d_s
**"RBN"**	**4.50** ±0.30	2.80 ±0.30	3.15 ±0.30
**"RBN2"**	**4.50** ±0.30	2.80 ±0.30	3.15 ±0.30

Lenghts and Thread Lenghts

**d_1**	L		L_G	
	min	max	min	max
**4.50**	35.0	80.0	18.0	40.0

Tolerance (L and L_G): according to EAD 130118-01-0603.
Intermediate lengths (L) are possible.
Intermediate thread lengths (L_G) are possible.

All dimensions in [mm].

Annex A.25 **Rotho Blaas screws**

STAINLESS STEEL
PARTIAL THREAD
Ø 5.0 mm

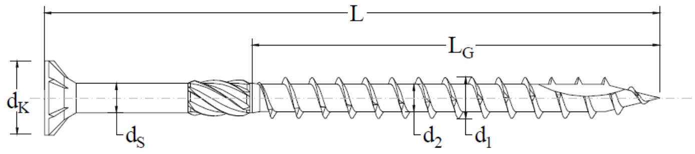

Alternative names:

SCI

Alternative head types:

Headstamps (supplier head mark and specific lenght) optional.

countersunk head
with or without milling ribs under head

"CS"

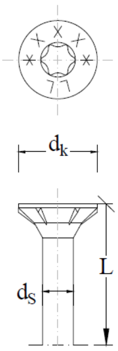

d_1	5.00
d_K	10.00 ±0.60

Alternative thread tip types:

Secondary rough thread optional.

"RBN"
with or without cutting edge

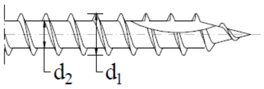

"RBN2"
with or without cutting edge

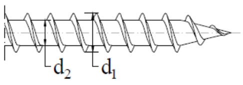

	d_1	d_2	d_s
**"RBN"**	**5.00** ±0.30	3.40 ±0.30	3.65 ±0.30
**"RBN2"**	**5.00** ±0.30	3.40 ±0.30	3.65 ±0.30

Lenghts and Thread Lenghts

d_1	L		L_G	
	min	max	min	max
**5.00**	40.0	100.0	20.0	50.0

Tolerance (L and L_G): according to EAD 130118-01-0603.
Intermediate lengths (L) are possible.
Intermediate thread lengths (L_G) are possible.

All dimensions in [mm].

Annex A.26 **Rotho Blaas screws** rothoblaas

STAINLESS STEEL
PARTIAL THREAD
Ø 6.0 mm

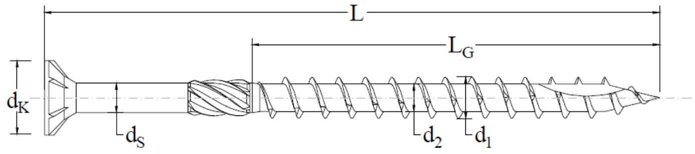

Alternative names:

SCI

Alternative head types:

Headstamps (supplier head mark and specific lenght) optional.

countersunk head
with or without milling ribs under head

"CS"

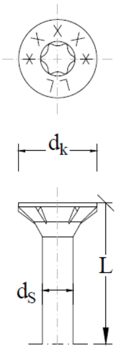

**d_1**	**6.00**
d_K	12.00<br>±0.60

Alternative thread tip types:

Secondary rough thread optional.

**"RBN"**<br>with or without cutting edge	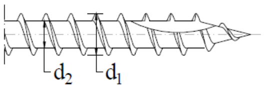
**"RBN2"**<br>with or without cutting edge	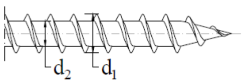

	**d_1**	d_2	d_s
**"RBN"**	**6.00**<br>±0.30	3.95<br>±0.30	4.30<br>±0.30
**"RBN2"**	**6.00**<br>±0.30	3.95<br>±0.30	4.30<br>±0.30

Lenghts and Thread Lenghts

**d_1**	L		L_G	
	min	max	min	max
**6.00**	50.0	300.0	24.0	75.0

Tolerance (L and L_G): according to EAD 130118-01-0603.
Intermediate lengths (L) are possible.
Intermediate thread lengths (L_G) are possible.

All dimensions in [mm].

Annex A.27 **Rotho Blaas screws**

STAINLESS STEEL
PARTIAL THREAD
Ø 8.0 mm

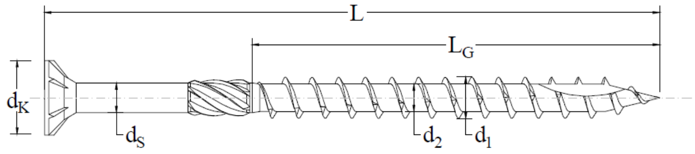

Alternative names:

SCI

Alternative head types:

Headstamps (supplier head mark and specific lenght) optional.

countersunk head
with or without milling ribs under head

"CS"

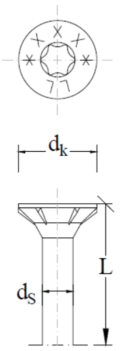

**d_1**	**8.00**
d_K	14.50 ±0.73

Alternative thread tip types:

Secondary rough thread optional.

"RBN"
with or without cutting edge

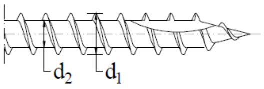

"RBN2"
with or without cutting edge

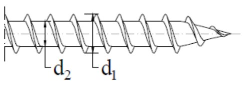

	**d_1**	d_2	d_s
**"RBN"**	**8.00** ±0.40	5.40 ±0.30	5.80 ±0.30
**"RBN2"**	**8.00** ±0.40	5.40 ±0.30	5.80 ±0.30

Lenghts and Thread Lenghts

**d_1**	L		L_G	
	min	max	min	max
**8.00**	40.0	280.0	32.0	100.0

Tolerance (L and L_G): according to EAD 130118-01-0603.
Intermediate lengths (L) are possible.
Intermediate thread lengths (L_G) are possible.

All dimensions in [mm].

Annex A.28 | **Rotho Blaas screws** | rothoblaas

STAINLESS STEEL
PARTIAL THREAD
Ø 4.0 mm

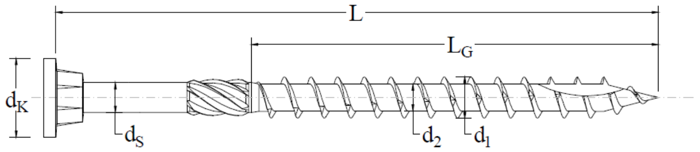

Alternative names:

KKF/KGA/GHKF

Alternative head types:

Headstamps (supplier head mark and specific lenght) optional.

washer head
with cylindrical
underhead
with or without milling
ribs under head
"WU1"

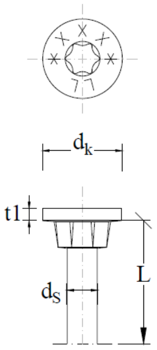

**d_1**	**4.00**
d_K	7.70 ±0.50
t_1	1.00 ±0.30

Alternative thread tip types:

Secondary rough thread optional.

"RBN"
with or without
cutting edge

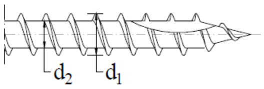

"RBN2"
with or without
cutting edge

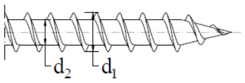

	d_1	d_2	d_s
**"RBN"**	**4.00** ±0.30	2.60 ±0.30	2.90 ±0.30
**"RBN2"**	**4.00** ±0.30	2.60 ±0.30	2.90 ±0.30

Lenghts and Thread Lenghts

**d_1**	L		L_G	
	min	max	min	max
**4.00**	30.0	80.0	16.0	40.0

Tolerance (L and L_G): according to EAD 130118-01-0603.
Intermediate lengths (L) are possible.
Intermediate thread lengths (L_G) are possible.

All dimensions in [mm].

Annex A.29 | **Rotho Blaas screws** | rothoblaas

STAINLESS STEEL
PARTIAL THREAD
Ø 4.5 mm

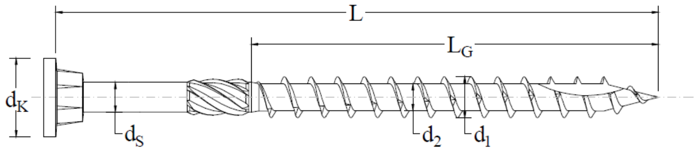

Alternative names:

KKF/KGA/GHKF

Alternative head types:

Headstamps (supplier head mark and specific lenght) optional.

washer head with cylindrical underhead
with or without milling ribs under head
"WU1"

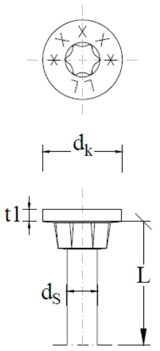

**d_1**	**4.50**
d_K	8.70 ±0.60
t_1	1.00 ±0.30

Alternative thread tip types:

Secondary rough thread optional.

"RBN"
with or without cutting edge

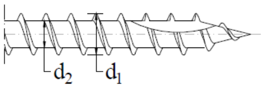

"RBN2"
with or without cutting edge

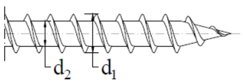

	**d_1**	d_2	d_s
**"RBN"**	**4.50** ±0.30	3.05 ±0.30	3.35 ±0.30
**"RBN2"**	**4.50** ±0.30	3.05 ±0.30	3.35 ±0.30

Lenghts and Thread Lenghts

**d_1**	L		L_G	
	min	max	min	max
**4.50**	20.0	80.0	18.0	40.0

Tolerance (L and L_G): according to EAD 130118-01-0603.
Intermediate lengths (L) are possible.
Intermediate thread lengths (L_G) are possible.

All dimensions in [mm].

Annex A.30 **Rotho Blaas screws** rothoblaas

STAINLESS STEEL
PARTIAL THREAD
Ø 5.0 mm

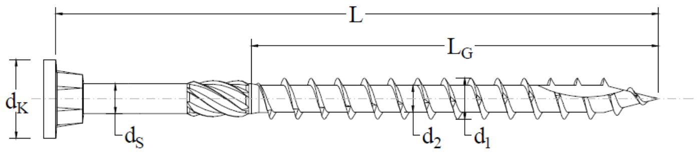

Alternative names:

KKF/KGA/GHKF

Alternative head types:

Headstamps (supplier head mark and specific lenght) optional.

washer head with cylindrical underhead<br>with or without milling ribs under head<br>**"WU1"**	washer head with cylindrical underhead<br>with or without milling ribs under head<br>**"WU1"**
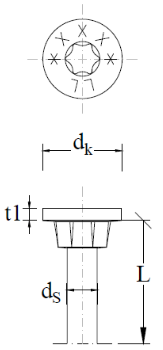	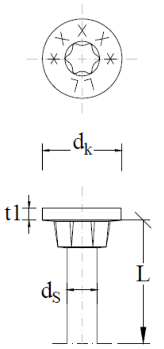

**d_1**	**5.00**
d_K	8.70 ±0.60
t_1	1.00 ±0.30

**d_1**	**5.00**
d_K	9.65 ±0.60
t_1	1.00 ±0.30

Alternative thread tip types:

**"RBN"**<br>with or without cutting edge	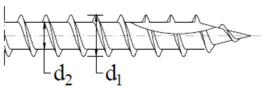
**"RBN2"**<br>with or without cutting edge	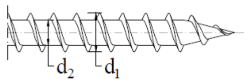

Secondary rough thread optional.

	**d_1**	d_2	d_s
**"RBN"**	**5.00** ±0.30	3.25 ±0.30	3.60 ±0.30
**"RBN2"**	**5.00** ±0.30	3.25 ±0.30	3.60 ±0.30

Lenghts and Thread Lenghts

**d_1**	L		L_G	
	min	max	min	max
**5.00**	40.0	120.0	20.0	60.0

Tolerance (L and L_G): according to EAD 130118-01-0603.
Intermediate lengths (L) are possible.
Intermediate thread lengths (L_G) are possible.

All dimensions in [mm].

Annex A.31 **Rotho Blaas screws** rothoblaas

STAINLESS STEEL
PARTIAL THREAD
Ø 6.0 mm

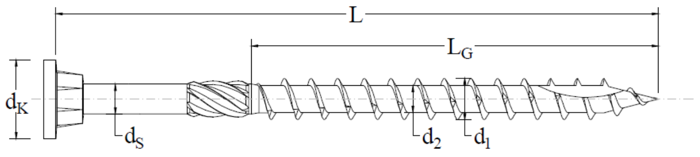

Alternative names:

KKF/KGA/GHKF

Alternative head types:

Headstamps (supplier head mark and specific lenght) optional.

washer head with cylindrical underhead
with or without milling ribs under head
"WU1"

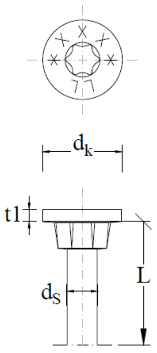

**d_1**	**6.00**
d_K	11.65 ±0.60
t_1	1.00 ±0.30

Alternative thread tip types:

Secondary rough thread optional.

"RBN"
with or without cutting edge

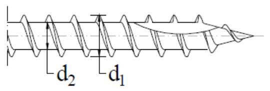

"RBN2"
with or without cutting edge

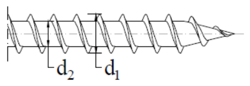

	**d_1**	d_2	d_s
**"RBN"**	**6.00** ±0.30	4.05 ±0.30	4.30 ±0.30
**"RBN2"**	**6.00** ±0.30	4.05 ±0.30	4.30 ±0.30

Lenghts and Thread Lenghts

**d_1**	L		L_G	
	min	max	min	max
**6.00**	60.0	300.0	24.0	100.0

Tolerance (L and L_G): according to EAD 130118-01-0603.
Intermediate lengths (L) are possible.
Intermediate thread lengths (L_G) are possible.

All dimensions in [mm].

Annex A.32 | **Rotho Blaas screws** | rothoblaas

STAINLESS STEEL
PARTIAL THREAD
Ø 8.0 mm

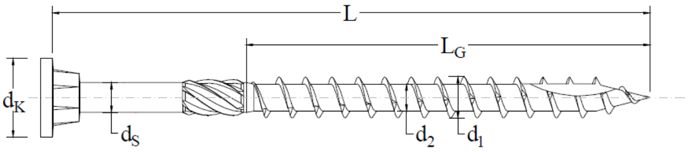

Alternative names:

KKF/KGA/GHKF

Alternative head types:

Headstamps (supplier head mark and specific lenght) optional.

washer head with cylindrical underhead<br>with or without milling ribs under head<br>**"WU1"**	washer head with cylindrical underhead<br>with or without milling ribs under head<br>**"WU2"**	countersunk head<br>with or without milling ribs under head<br>**"CS"**
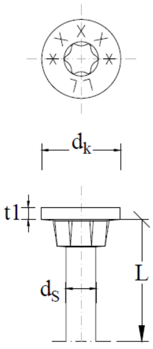	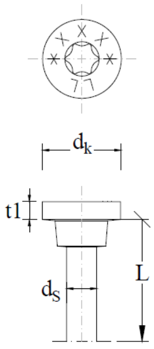	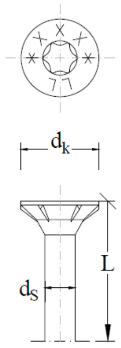

**d_1**	**8.00**
d_K	14.50 ±0.73
t_1	1.75 ±0.30

**d_1**	**8.00**
d_K	14.50 ±0.73
t_1	3.40 ±0.30

**d_1**	**8.00**
d_K	14.50 ±0.73

Alternative thread tip types:

**"RBN"**<br>with or without cutting edge	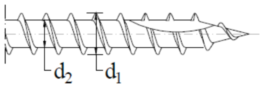
**"RBN2"**<br>with or without cutting edge	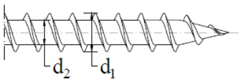

Secondary rough thread optional.

	**d_1**	d_2	d_s
**"RBN"**	**8.00** ±0.40	5.40 ±0.30	5.80 ±0.30
**"RBN2"**	**8.00** ±0.40	5.40 ±0.30	5.80 ±0.30

Lenghts and Thread Lenghts

**d_1**	L		L_G	
	min	max	min	max
**8.00**	60.0	320.0	24.0	100.0

Tolerance (L and L_G): according to EAD 130118-01-0603.
Intermediate lengths (L) are possible.
Intermediate thread lengths (L_G) are possible.

All dimensions in [mm].

Annex A.33 **Rotho Blaas threaded rods** rothoblaas

CARBON STEEL
THREADED ROD
Ø 16.0 mm

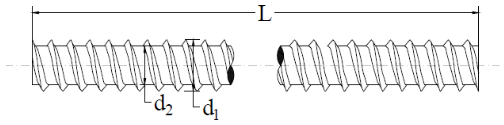

Alternative names:

RTR

Alternative thread tip types:

"RTR"
with or without
cutting edge

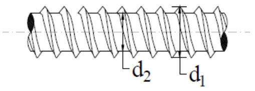

Stamps (supplier head mark and specific lenght) optional.

	d_1	d_2
**"RTR"**	**16.00** ±0.80	12.00 ±0.60

Lenghts

d_1	L min	L max
**16.00**	100.0	3000.0

Tolerance (L): according to EAD 130118-01-0603.
Intermediate lengths (L) are possible.

All dimensions in [mm].

Annex A.34 **Rotho Blaas threaded rods** rothoblaas

CARBON STEEL
THREADED ROD
Ø 20.0 mm

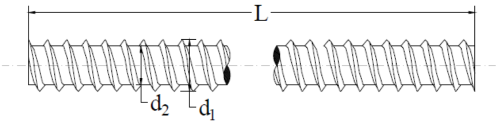

Alternative names:

RTR

Alternative thread tip types:

"RTR"
with or without
cutting edge

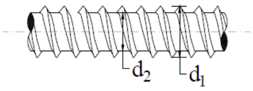

Stamps (supplier head mark and specific lenght) optional.

	d_1	d_2
**"RTR"**	**20.00** ±1.00	15.00 ±0.75

Lenghts

d_1	L min	L max
**20.00**	100.0	3000.0

Tolerance (L): according to EAD 130118-01-0603.
Intermediate lengths (L) are possible.

All dimensions in [mm].

Annex A.35 **Rotho Blaas screws** rothoblaas

CARBON OR STAINLESS STEEL
TERRACE SCREW
Ø 5.0 mm

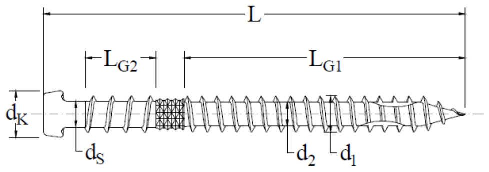

Alternative names:

KKT/MN

Alternative head types:

Headstamps (supplier head mark and specific lenght) optional.

cone-shaped head

"CO"

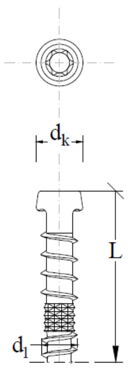

d_1	**5.00**
d_K	6.75 ±0.50

Alternative thread tip types:

"RBK1" with or without cutting edge

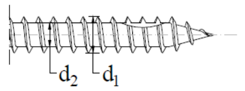

"RBK2" with or without cutting edge

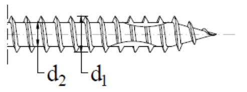

Secondary rough thread (knurled part) optional.

	d_1	d_2	d_s
**"RBK1"**	**5.00** ±0.30	3.10 ±0.30	3.80 ±0.30
**"RBK2"**	**5.00** ±0.30	3.10 ±0.30	3.80 ±0.30

Lenghts and Thread Lenghts

d_1	L		L_{G1}		L_{G2}	
	min	max	min	max	min	max
**5.00**	40.0	80.0	20.0	53.0	10.0	12.0

Tolerance (L, L_{G1} and L_{G2}): according to EAD 130118-01-0603.
Intermediate lengths (L) are possible.
Intermediate thread lengths (L_G) are possible.

All dimensions in [mm].

Annex A.36 **Rotho Blaas screws** rothoblaas

CARBON OR STAINLESS STEEL
TERRACE SCREW
Ø 5.0 mm

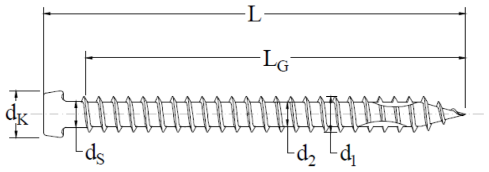

Alternative names:

KKT/MN

Alternative head types:

Headstamps (supplier head mark and specific lenght) optional.

cone-shaped head

"CO"

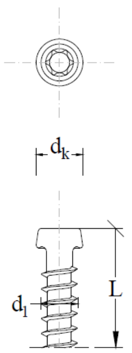

d_1	**5.00**
d_K	6.75 ±0.50

Alternative thread tip types:

"RBK3"
with or without cutting edge
with 1, 2 or without cut

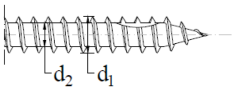

Secondary rough thread (knurled part) optional.

	d_1	d_2	d_s
**"RBK3"**	**5.00** ±0.30	3.10 ±0.30	3.80 ±0.30

Lenghts and Thread Lenghts

d_1	L		L_G	
	min	max	min	max
**5.00**	24.0	80.0	20.0	53.0

Tolerance (L and L_G): according to EAD 130118-01-0603.
Intermediate lengths (L) are possible.
Intermediate thread lengths (L_G) are possible.

All dimensions in [mm].

Annex A.37 **Rotho Blaas screws** rothoblaas

CARBON OR STAINLESS STEEL
TERRACE SCREW
Ø 6.0 mm

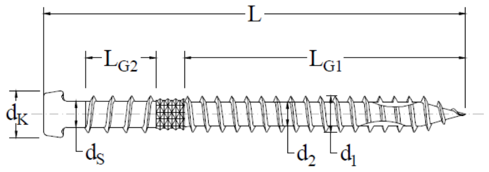

Alternative names:

KKT/MN

Alternative head types:

Headstamps (supplier head mark and specific lenght) optional.

cone-shaped head

"CO"

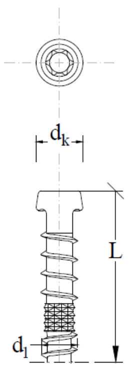

**d_1**	**6.00**
d_K	7.75 ±0.50

Alternative thread tip types:

"RBK1"
with or without cutting edge

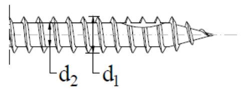

"RBK2"
with or without cutting edge

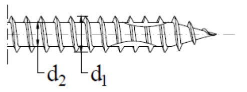

Secondary rough thread (knurled part) optional.

	**d_1**	d_2	d_s
**"RBK1"**	**6.00** ±0.30	3.90 ±0.30	4.50 ±0.30
**"RBK2"**	**6.00** ±0.30	3.90 ±0.30	4.50 ±0.30

Lenghts and Thread Lenghts

**d_1**	L		L_{G1}		L_{G2}	
	min	max	min	max	min	max
**6.00**	60.0	120.0	24.0	60.0	12.0	26.0

Tolerance (L, L_{G1} and L_{G2}): according to EAD 130118-01-0603.
Intermediate lengths (L) are possible.
Intermediate thread lengths (L_G) are possible.

All dimensions in [mm].

Annex A.38 **Rotho Blaas washers** rothoblaas

CARBON STEEL OR STAINLESS STEEL
COUNTERSUNK WASHER
Ø 6.0 ÷12.0 mm

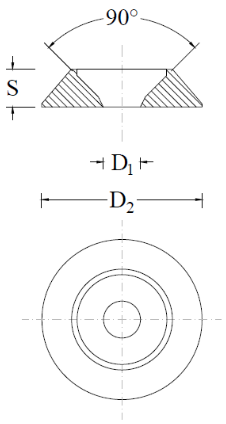

Alternative names:

HUS/SUS/SCB/SHT

Headstamps (supplier head mark and specific lenght) optional.

D_1	D_2	S
**7.50** ±0.38	20.00 ±1.00	4.60 ±0.30
**8.50** ±0.43	25.00 ±1.25	5.40 ±0.30
**10.80** ±0.54	30.00 ±1.50	6.40 ±0.33
**13.00** ±0.65	35.00 ±1.75	7.50 ±0.38
**14.00** ±0.70	37.00 ±1.85	8.50 ±0.43

Intermediate sizes are possible.

All dimensions in [mm].

Annex A.39 **Rotho Blaas washers** rothoblaas

CARBON STEEL
COUNTERSUNK WASHER 45°
Ø 9.0 ÷13.0 mm

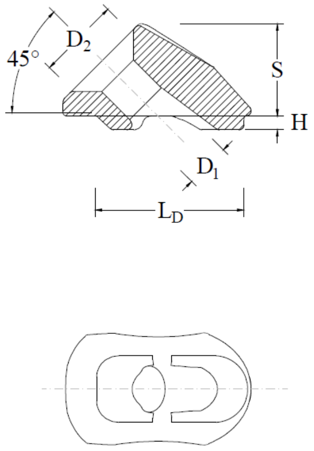

Alternative names:

VGU

Headstamps (supplier head mark and specific lenght) optional.

D_1	D_2	S	H	L_D
**9.70** ±0.49	19.00 ±0.95	20.00 ±1.00	3.00 ±0.30	31.80 ±1.50
**11.80** ±0.59	23.00 ±1.15	24.40 ±1.22	3.60 ±0.30	38.80 ±1.50
**14.00** ±0.70	27.40 ±1.37	28.70 ±1.44	4.30 ±0.30	45.80 ±1.50

Intermediate sizes are possible.

All dimensions in [mm].

Annex B
Minimum distances and spacing

Axially loaded screws or threaded rods
Single configuration

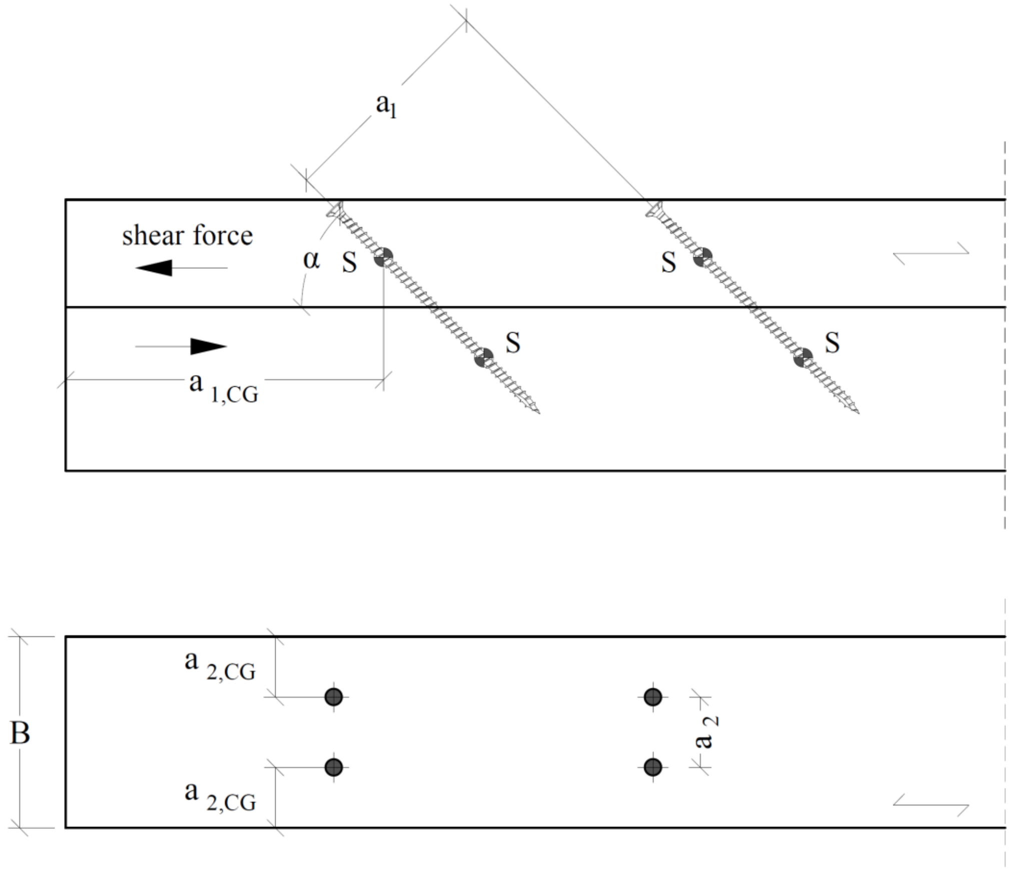

$a_1 \geq 5 \cdot d$
$a_2 \geq 2{,}5 \cdot d$ if $a_1 \cdot a_2 \geq 25 \cdot d^2$
$a_{3,c} \geq 10 \cdot d$
$a_{4,c} \geq 4 \cdot d$

Minimum distances and spacing see also 3.12
Minimum timber thickness $t = 12 \cdot d$, see also 3.12

S = centroid of the part of the screw or threaded rod in the timber

Axially loaded screws or threaded rods
Crosswise configuration

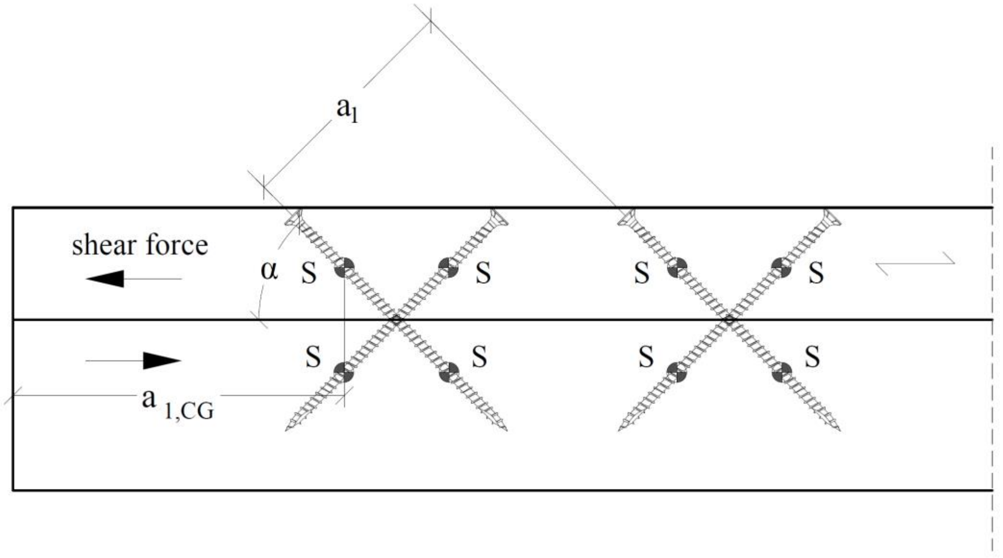

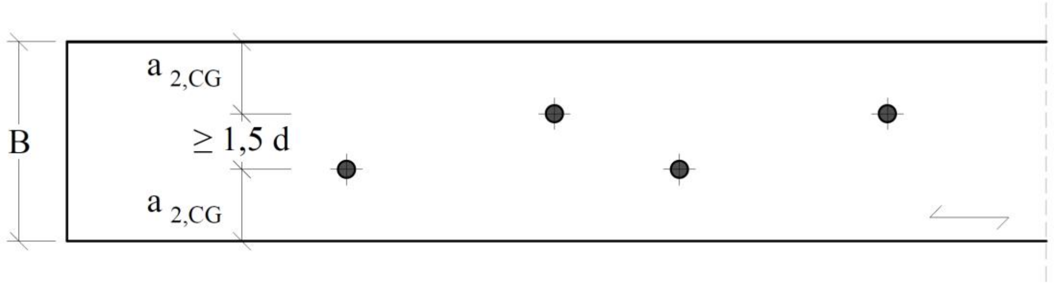

$a_1 \geq 5 \cdot d$
$a_2 \geq 1{,}5 \cdot d$ if $a_1 \cdot a_2 \geq 25 \cdot d^2$
$a_{3,c} \geq 10 \cdot d$
$a_{4,c} \geq 4 \cdot d$

Minimum distances and spacing see also 3.12
Minimum timber thickness t = 12 · d, see also 3.12

S = centroid of the part of the screw or threaded rod in the timber

Axially or laterally loaded screws or threaded rods in the plane or edge surface of cross laminated timber

Definition of spacing, end and edge distances in the plane surface unless otherwise specified in the technical specification (ETA or hEN) for the cross laminated timber:

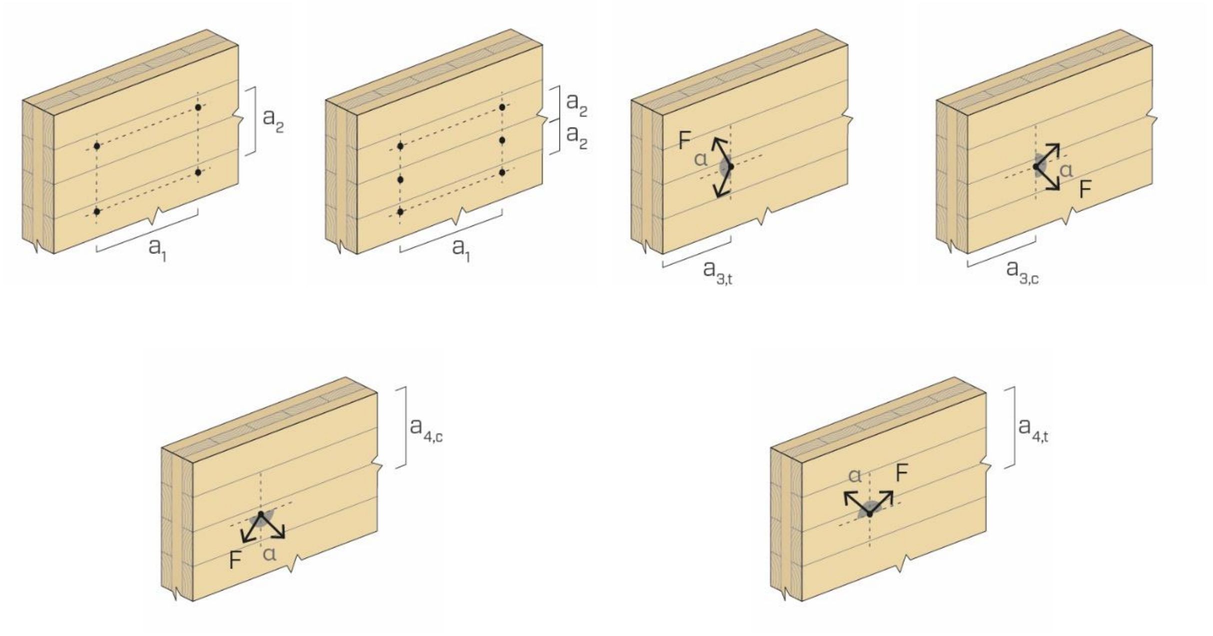

Note. Drawing is copyright Rotho Blaas

Definition of spacing, end and edge distances in the edge surface unless otherwise specified in the technical specification (ETA or hEN) for the cross laminated timber:

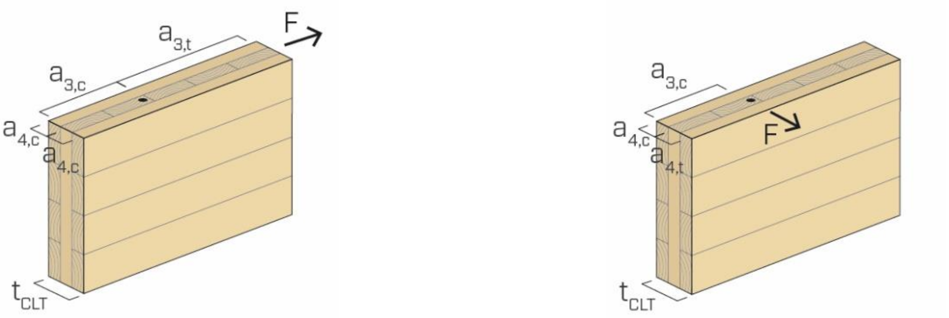

Note. Drawing is copyright Rotho Blaas

Table B1: Minimum spacing, end and edge distances of screws or threaded rods in the plane or edge surfaces of cross laminated timber

	a_1	$a_{3,t}$	$a_{3,c}$	a_2	$a_{4,t}$	$a_{4,c}$
Plane surface (see Figure 1)	$4 \cdot d$	$6 \cdot d$	$6 \cdot d$	$2,5 \cdot d$	$6 \cdot d$	$2,5 \cdot d$
Edge surface (see Figure 2)	$10 \cdot d$	$12 \cdot d$	$7 \cdot d$	$4 \cdot d$	$6 \cdot d$	$3 \cdot d$

Annex C
Compression reinforcement

“VGS”, “VGZ”, “VGZH” and “VGSH” screws with a full thread or “RTR” threaded rods may be used for reinforcement of timber members with compression stresses at an angle α to the grain of 45° < α < 90°. The compression force must be evenly distributed over all screws or threaded rods.

Reinforced end-bearings

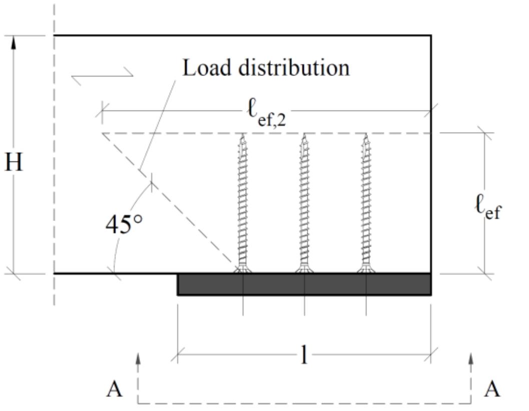

Reinforced centre-bearings

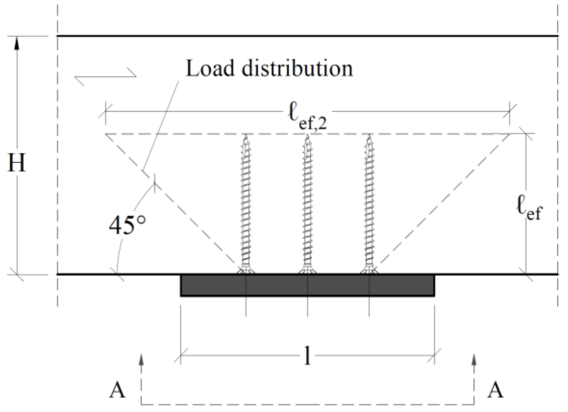

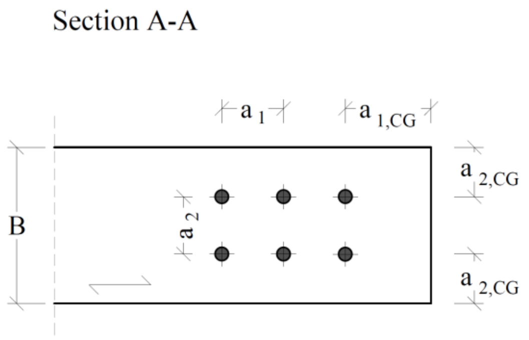

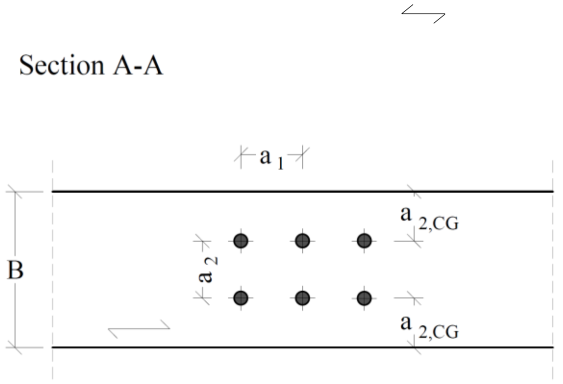

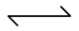 = grain direction

The design load-carrying capacity for a reinforced contact area with screws with a full thread or threaded rods at an angle α to the grain of 45° < α < 90° shall be calculated from:

$$F_{90,Rd} = \min \begin{cases} k_{c,90} \cdot B \cdot \ell_{ef,1} \cdot f_{c,90,d} + n \cdot F_{c,90,Rd} \\ B \cdot \ell_{ef,2} \cdot f_{c,90,d} \end{cases}$$

Where:

$F_{90,Rd}$	design load-carrying capacity of reinforced contact area [N]
$k_{c,90}$	factor for compression perpendicular to the grain according to EN 1995-1-1, 6.1.5
B	bearing width [mm]
H	component height [mm]
$\ell_{ef,1}$	effective length of contact area according to EN 1995-1-1, 6.1.5 [mm]
$f_{c,90,d}$	design compressive strength perpendicular to the grain [N/mm²]
n	number of reinforcement fasteners, $n = n_0 \cdot n_{90}$
n_0	number of reinforcement fasteners arranged in a row parallel to the grain
n_{90}	number of reinforcement fasteners arranged in a row perpendicular to the grain

$\ell_{ef,2}$	effective distribution length in the plane of the fastener tips [mm]
$\ell_{ef,2}$	$= 2 \cdot \ell_{ef} + (n_0 - 1) \cdot a_1$ for reinforced centre-bearings
$\ell_{ef,2}$	$= \ell_{ef} + (n_0 - 1) \cdot a_1 + \min(\ell_{ef}; a_{1,c})$ for reinforced end-bearings
ℓ_{ef}	point side penetration length [mm]
a_1	spacing parallel to grain [mm]
$a_{1,CG}$	end grain distance of the centre of the screw-part in timber [mm]
$F_{c,90,Rd}$	design compressive capacity [N]

An appropriate steel plate as intermediate layer between timber member and support has to be installed. The screws have to be driven into the timber member flush with the surface to provide both direct contact with the steel plate and direct contact between steel plate and timber.

Reinforcing screws or threaded rods for wood-based panels are not covered by this European Technical Assessment.

Annex D
Thermal insulation material on top of rafters or facades

Rotho Blaas screws with an outer thread diameter of at least 6 mm may also be used for the fixing of thermal insulation on top of rafters.

The thickness of the insulation shall not exceed 400 mm. The rafter insulation must be placed on top of solid timber or glued laminated timber rafters or cross laminated timber members and be fixed by battens arranged parallel to the rafters or by wood-based panels on top of the insulation layer. The insulation of vertical facades is also covered by the rules given here.

Screws must be screwed in the rafter through the battens or panels and the insulation without pre-drilling in one sequence.

The angle α between the screw axis and the grain direction of the rafter should be between 30° and 90°.

The rafter consists of solid timber (softwood) according to EN 338 or EN 14081, glued laminated timber according to EN 14081, cross-laminated timber, or laminated veneer lumber according to EN 14374 or to ETA or similar glued members according to ETA.

The battens must be from solid timber (softwood) according to EN 338:2003-04 or EN 14081. The minimum thickness t and the minimum width b of the battens is given as follows:

Screws $d \leq 8$ mm:	b_{min} = 50 mm	t_{min} = 30 mm
Screws $9 \leq d \leq 10$ mm:	b_{min} = 60 mm	t_{min} = 40 mm
Screws $d = 11$ mm:	b_{min} = 80 mm	t_{min} = 60 mm
Screws $12 \leq d \leq 13$ mm:	b_{min} = 100 mm	t_{min} = 80 mm

Alternatively to the battens, boards with a minimum thickness of 20 mm from plywood according to EN 636, particle board according to EN 312, oriented strand board OSB/3 and OSB/4 according to EN 300, solid wood panels according to EN 13353 or to ETA or national provision that apply at the installation site or cross laminated timber according to ETA may be used.

The insulation must comply with a European Technical Specification.

Friction forces shall not be considered for the design of the characteristic axial capacity of the screws.

The anchorage of wind suction forces as well as the bending stresses of the battens or the boards, respectively, shall be considered in design. Additional screws perpendicular to the grain of the rafter (angle α = 90°) may be arranged if necessary.

The maximum screw spacing is e_S = 1,75 m.

Thermal insulation on rafters with parallel inclined screws

Mechanical model

The system of rafter, thermal insulation material on top of rafter and battens parallel to the rafter may be considered as a beam on elastic foundation. The batten represents the beam, and the thermal insulation material on top of the rafter the elastic foundation. The minimum compression stress of the thermal insulation material at 10 % deformation, measured according to EN 826 (1), shall be $\sigma_{(10\,\%)}$ = 0,05 N/mm². The batten is loaded perpendicular to the axis by point loads F_b. Further point loads F_s are from the shear load of the roof due to dead and snow load, which are transferred from the screw heads into the battens. The battens or boards, respectively, must have sufficient strength and stiffness.

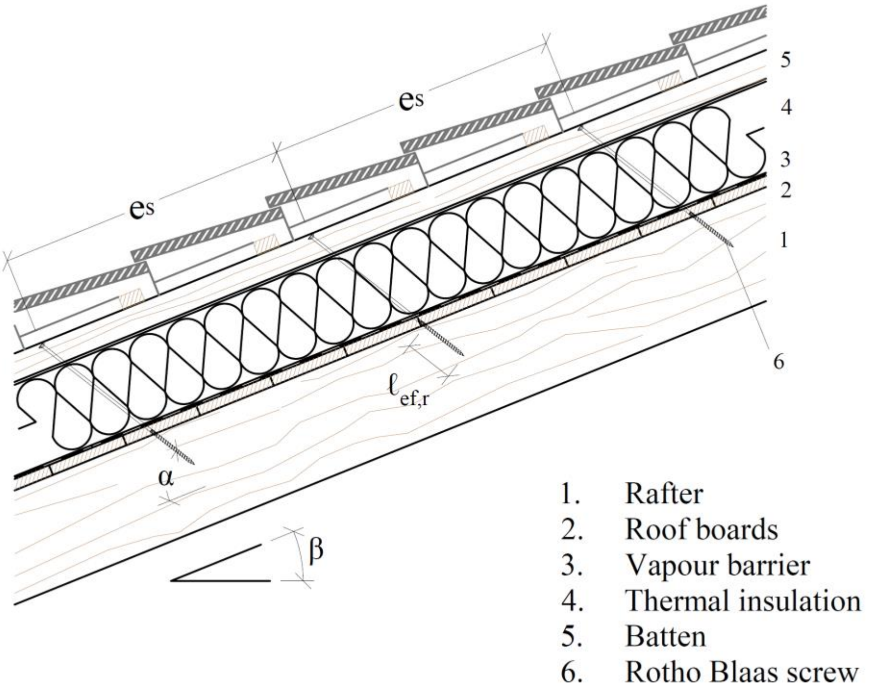

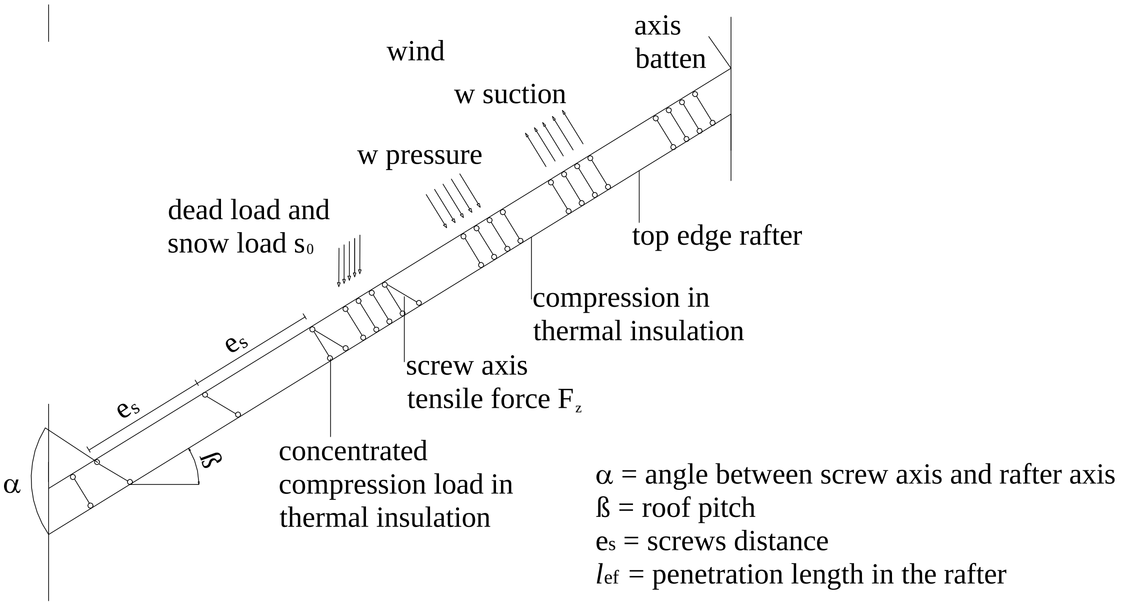

(1) EN 826:1996 Thermal insulating products for building applications - Determination of compression behaviour

Design of the battens

The bending stresses are calculated as:

$$M = \frac{(F_b + F_s) \cdot \ell_{char}}{4}$$

where

ℓ_{char} = characteristic length $\ell_{char} = \sqrt[4]{\frac{4 \cdot EI}{w_{ef} \cdot K}}$

EI = bending stiffness of the batten
K = coefficient of subgrade
w_{ef} = effective width of the thermal insulation material
F_b = point loads perpendicular to the battens
F_s = point loads perpendicular to the battens, load application in the area of the screw heads

The coefficient of subgrade K may be calculated from the modulus of elasticity E_{HI} and the thickness t_{HI} of the thermal insulation material if the effective width w_{ef} of the thermal insulation material under compression is known. Due to the load extension in the thermal insulation material the effective width w_{ef} is greater than the width of the batten or rafter, respectively. For further calculations, the effective width w_{ef} of the thermal insulation material may be determined according to:

$$w_{ef} = w + t_{HI} / 2$$

where
w = minimum width of the batten or rafter, respectively
t_{HI} = thickness of the thermal insulation material

$$K = \frac{E_{HI}}{t_{HI}}$$

The following condition shall be satisfied:

$$\frac{\sigma_{m,d}}{f_{m,d}} = \frac{M_d}{W \cdot f_{m,d}} \leq 1$$

For the calculation of the section modulus W the net cross section has to be considered.

The shear stresses shall be calculated according to:

$$V = \frac{(F_b + F_s)}{2}$$

The following condition shall be satisfied:

$$\frac{\tau_d}{f_{v,d}} = \frac{1{,}5 \cdot V_d}{A \cdot f_{v,d}} \leq 1$$

For the calculation of the cross section area the net cross section has to be considered.

Design of the thermal insulation material

The compressive stresses in the thermal insulation material shall be calculated according to:

$$\sigma = \frac{1{,}5 \cdot F_b + F_s}{2 \cdot \ell_{char} \cdot w}$$

The design value of the compressive stress shall not be greater than 110 % of the compressive stress at 10 % deformation calculated according to EN 826.

Design of the screws

The screws are loaded predominantly axially. The axial tension force in the screw may be calculated from the shear loads of the roof R_s:

$$T_S = \frac{R_S}{\cos\alpha}$$

The load-carrying capacity of axially loaded screws is the minimum design value of the axial withdrawal capacity of the threaded part of the screw, the head pull-through capacity of the screw and the tensile capacity of the screw.

In order to limit the deformation of the screw head for thermal insulation material thicknesses over 200 mm or with compressive strength below 0,12 N/mm², respectively, the axial withdrawal capacity of the screws shall be reduced by the factors k_1 and k_2:

- for “HBS”, “HBSP”, “TBS”, “KKF”, “SCI”, “HBSH” screws with partial thread:

$$F_{ax,\alpha,Rd} = \min\left\{ \frac{k_{ax} \cdot f_{ax,d} \cdot d \cdot \ell_{ef} \cdot k_1 \cdot k_2}{k_\beta} \cdot \left(\frac{\rho_k}{\rho_a}\right)^{0.8} ; f_{head,d} \cdot d_h^2 \cdot \left(\frac{\rho_k}{\rho_a}\right)^{0.8} ; f_{tens,d} \right\}$$

- for “DGZ”, “VGS”, “GWZ”, “GWS”, “VGZ”, "VGZH and VGSH" screws with full thread or double thread:

$$F_{ax,\alpha,Rd} = \min\left\{ \begin{array}{l} \frac{k_{ax} \cdot f_{ax,d} \cdot d \cdot \ell_{ef} \cdot k_1 \cdot k_2}{k_\beta} \cdot \left(\frac{\rho_k}{\rho_a}\right)^{0,8} \\ \max\left\{ f_{head,d} \cdot d_h^2 ; \frac{k_{ax} \cdot f_{ax,d} \cdot d \cdot \ell_{ef,b} \cdot k_1 \cdot k_2}{k_\beta} \right\} \cdot \left(\frac{\rho_k}{\rho_a}\right)^{0,8} \\ f_{tens,d} \end{array} \right\}$$

where:

$F_{ax,\alpha,d}$	design value of the load-carrying capacity of axially loaded screws [N]
$f_{ax,d}$	design value of the axial withdrawal parameter of the threaded part of the screw in the rafter or batten [N/mm²]
d	outer thread diameter of the screw [mm]
ℓ_{ef}	point side penetration length of the threaded part of the screw in the rafter, $l_{ef} \geq 40$ mm [mm]
$\ell_{ef,b}$	length of the threaded part in the batten including the head for tensile force [mm]
ρ_k	characteristic density of the wood-based member [kg/m³]
ρ_a	associated density [kg/m³]
$f_{head,d}$	design value of the head pull-through parameter of the screw [N/mm²]
d_h	head diameter [mm]
$f_{tens,d}$	design value of the tensile capacity of the screw [N]
k_1	min {1; 200/t_{HI}}
k_2	min {1; $\sigma_{10\%}$/0,12}
t_{HI}	thickness of the thermal insulation material [mm]
$\sigma_{10\%}$	compressive stress of the thermal insulation material under 10 % deformation [N/mm²]

If k_1 and k_2 are considered, the deflection of the battens does not need to be considered. Alternatively to the battens, panels with a minimum thickness of 20 mm from plywood according to EN 636 or an ETA or national provisions that apply at the installation site, particle board according to EN 312 or an ETA or national provisions that apply at the installation site, oriented strand board according to EN 300 or an ETA or national provisions that apply at the installation site and solid wood panels according to EN 13353 or an ETA or national provisions that apply at the installation site or cross laminated timber according to an ETA may be used.

Thermal insulation on rafters with alternatively inclined “DGZ”, “GWZ”, “GWS”, “VGZ” or “VGS” screws

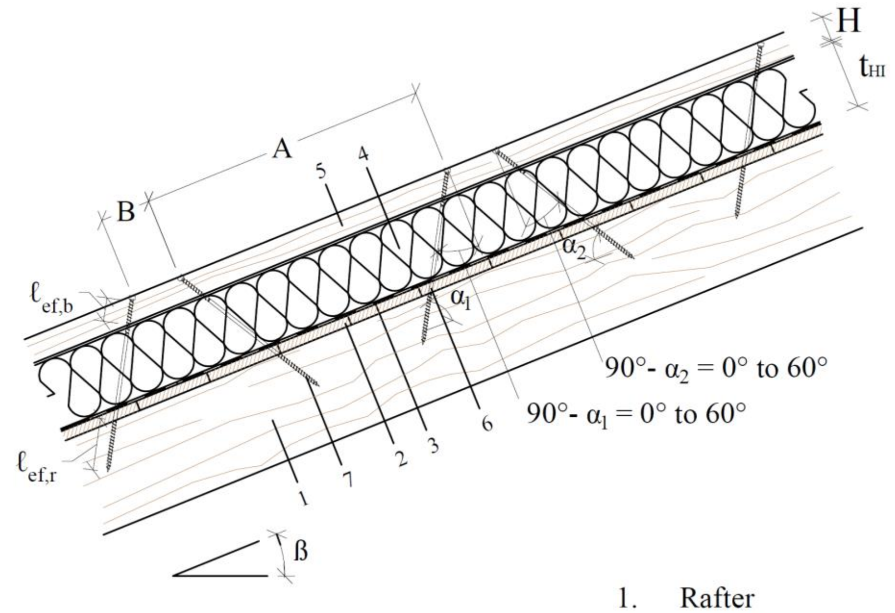

1. Rafter
2. Roof boards
3. Vapour barrier
4. Thermal insulation
5. Batten
6. Rotho Blaas screw loaded in compression
7. Rotho Blaas screw loaded in tension

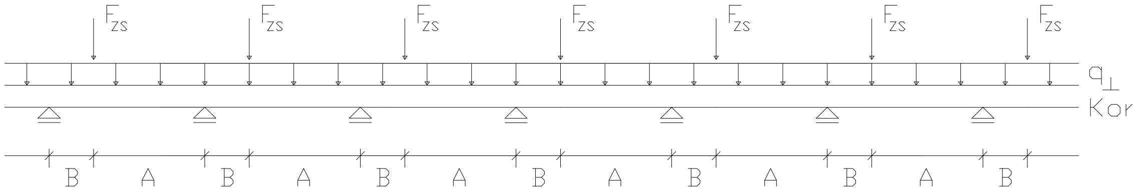

Counter batten

Mechanical model

Depending on the screw spacing and the arrangement of tensile and compressive screws with different inclinations the battens are loaded by significant bending moments. The bending moments are derived based on the following assumptions:

- The tensile and compressive loads in the screws are determined based on equilibrium conditions from the actions parallel and perpendicular to the roof plane.
  These actions are constant line loads $q_{\perp}$ and $q_{||}$.
- The screws act as hinged columns supported 10 mm within the batten or rafter, respectively. The effective column length consequently equals the length of the screw between batten and rafter plus 20 mm.
- The batten is considered as a continuous beam with a constant span ℓ = A + B. The battens or boards, respectively, must have sufficient strength and stiffness.
  The compressive screws constitute the supports of the continuous beam while the tensile screws transfer concentrated loads perpendicular to the batten axis.

The screws are predominantly loaded in withdrawal or compression, respectively. The screw’s normal forces are determined based on the loads parallel and perpendicular to the roof plane:

Compressive screw: $$F_{c,Ed} = (A+B) \cdot \left(-\frac{q_{II} \cdot \sin\alpha_2 + q_{\perp} \cdot \cos\alpha_2}{\sin(\alpha_1 + \alpha_2)} \right)$$

Tensile screw: $$F_{t,Ed} = (A+B) \cdot \left(\frac{q_{II} \cdot \sin\alpha_1 - q_{\perp} \cdot \cos\alpha_1}{\sin(\alpha_1 + \alpha_2)} \right)$$

The bending moments in the batten follow from the constant line load $q_{\perp}$ and the load components perpendicular to the batten from the tensile screws. The span of the continuous beam is (A + B). The load component perpendicular to the batten from the tensile screw is:

$$F_{ZS,Ed} = (A+B) \cdot \left(\frac{q_{II} \cdot \sin\alpha_1 \cdot \sin\alpha_2 - q_{\perp} \cdot \cos\alpha_1 \cdot \sin\alpha_2}{\sin(\alpha_1 + \alpha_2)} \right)$$

where:

q_{II}	constant line load parallel to batten
$q_{\perp}$	constant line load perpendicular to batten
α_1	angle between compressive screw axis and grain direction
α_2	angle between tensile screw axis and grain direction

A positive value for F_{ZS} means a load towards the rafter, a negative value a load away from the rafter.

Design of the screws

The load-carrying capacity of the screws shall be calculated as follows:

Screws loaded in tension:

$$F_{ax,\alpha,Rd} = \min\left\{ \frac{k_{ax} \cdot f_{ax,d} \cdot d \cdot \ell_{ef,b}}{k_\beta} \cdot \left(\frac{\rho_{b,k}}{\rho_a}\right)^{0.8}; \frac{k_{ax} \cdot f_{ax,d} \cdot d \cdot \ell_{ef,r}}{k_\beta} \cdot \left(\frac{\rho_{r,k}}{\rho_a}\right)^{0.8}; f_{tens,d} \right\}$$

Screws loaded in compression:

$$F_{ax,\alpha,Rd} = \min\left\{ \frac{k_{ax} \cdot f_{ax,d} \cdot d \cdot \ell_{ef,b}}{k_\beta} \cdot \left(\frac{\rho_{b,k}}{\rho_a}\right)^{0.8}; \frac{k_{ax} \cdot f_{ax,d} \cdot d \cdot \ell_{ef,r}}{k_\beta} \cdot \left(\frac{\rho_{r,k}}{\rho_a}\right)^{0.8}; \frac{\kappa_c \cdot N_{pl,k}}{\gamma_{M1}} \right\}$$

where:

$F_{ax,\alpha,Rd}$	design value of the load-carrying capacity of the screw [N]
$f_{ax,d}$	design value of the axial withdrawal parameter of the threaded part of the screw in the rafter or batten [N/mm²]
d	outer thread diameter of the screw [mm]
$\ell_{ef,b}$	penetration length of the threaded part of the screw in the batten including the head for tensile and excluding the head for compressive force [mm]
$\ell_{ef,r}$	penetration length of the threaded part of the screw in the rafter, $l_{ef} \geq 40$ mm [mm]
$\rho_{b,k}$	characteristic density of the batten [kg/m³]
$\rho_{r,k}$	characteristic density of the rafter [kg/m³]
α	angle α_1 or α_2 between screw axis and grain direction, $30° \leq \alpha_1 \leq 90°$, $30° \leq \alpha_2 \leq 90°$
$f_{tens,d}$	design value of the tensile capacity of the screw [N]
γ_{M1}, γ_{M2}	partial factor according to EN 1993 or to the particular national annex
$\kappa_c \cdot N_{pl,k}$	buckling capacity of the screw [N]

Buckling capacity of the screw

Free screw length [mm]	"DGZ"		"GWZ", "GWS", "VGZ" or "VGS"				"VGZH" or "VGSH"	
	7 mm	9 mm	7 mm	9 mm	11 mm	13 mm	6 mm	8 mm
	$\kappa_c \cdot N_{pl,k}$ [kN]	$\kappa_c \cdot N_{pl,k}$ [kN]	$\kappa_c \cdot N_{pl,k}$ [kN]	$\kappa_c \cdot N_{pl,k}$ [kN]	$\kappa_c \cdot N_{pl,k}$ [kN]	$\kappa_c \cdot N_{pl,k}$ [kN]	$\kappa_c \cdot N_{pl,k}$ [kN]	$\kappa_c \cdot N_{pl,k}$ [kN]
≤ 100	3,52	9,23	2,57	6,49	9,75	19,2	2,37	6,49
120	2,68	7,15	1,95	4,99	7,57	15,2	1,79	4,99
140	2,10	5,68	1,53	3,95	6,02	12,3	1,41	3,95
160	1,70	4,61	1,23	3,19	4,89	10,1	1,13	3,19
180	1,40	3,82	1,01	2,63	4,05	8,38	0,93	2,63
200	1,17	3,21	0,84	2,22	3,40	7,08		2,22
220	0,99	2,74	0,71	1,88	2,91	6,05		1,88
240	0,85	2,36	0,61	1,62	2,50	5,23		1,62
260	0,74	2,05	0,53	1,41	2,18	4,58		
280	0,65	1,80	0,47	1,23	1,91	4,03		
300	0,57	1,59	0,41	1,09	1,69	3,57		
320		1,42		0,97	1,51	3,19		
340		1,27		0,87	1,35	2,86		
360		1,15		0,79	1,22	2,58		
380		1,04		0,71	1,10	2,34		
400		0,95		0,65	1,01	2,14		
420					0,92	1,95		
440					0,84	1,80		
460					0,78	1,65		

where
free screw length = $t_{HI} / \sin \alpha$ [mm] ($\alpha = \alpha_1$ or α_2)

Annex E
Shear reinforcement

Unless specified otherwise in national provisions that apply at the installation site, the shear stress in reinforced areas of timber members with a stress component parallel to the grain shall fulfil the following condition:

$$\tau_d \leq \frac{f_{v,d} \cdot k_\tau}{\eta_H}$$

Where: τ_d is the design shear stress disregarding the reinforcement [N/mm²];

$f_{v,d}$ is the design shear strength [N/mm²];

$$k_\tau = 1 - 0{,}46 \cdot \sigma_{90,d} - 0{,}052 \cdot \sigma_{90,d}^2$$

$\sigma_{90,d}$ is the design stress perpendicular to the grain (negative value for compression) [N/mm²];

$$\sigma_{90,d} = \frac{F_{ax,d}}{\sqrt{2} \cdot b \cdot a_1}$$

$$F_{ax,d} = \frac{\sqrt{2} \cdot (1 - \eta_H) \cdot V_d \cdot a_1}{h}$$

$$\eta_H = \frac{G \cdot b}{G \cdot b + \frac{1}{2 \cdot \sqrt{2} \left(\frac{6}{\pi \cdot d \cdot h \cdot k_{ax}} + \frac{a_1}{EA_S} \right)}}$$

V_d is the design shear force [N];
G is the shear modulus of the timber member, G = 650 N/mm²,
b is the width of the timber member in mm,
d is the outer thread diameter in mm,
h is the depth of the timber member in mm,
k_{ax} is the connection stiffness between screw or rod and timber member in N/mm³,
k_{ax} = 5 N/mm³ for Rotho Blaas RTR rods d = 16 mm,
k_{ax} = 12,5 N/mm³ for Rotho Blaas VGZ screws d = 9 mm,
a_1 is the spacing parallel to the grain of the screws or rods arranged in one row in mm,
EA_S is the axial stiffness of one rod or screw [N],

$$EA_S = \frac{E \cdot \pi \cdot d_1^2}{4} = 165.000\, d_1^2$$

d_1 is the inner thread diameter of the rod or screw in mm.

The axial capacity of a threaded screw or rod shall fulfil the following condition:

$$\frac{F_{ax,d}}{F_{ax,Rd}} \leq 1$$

Where:

$F_{ax,Rd}$ Minimum of the design values of the withdrawal capacity and the tensile capacity of the reinforcing rods or screws [N]. The effective penetration length is 50 % of the threaded length.

A minimum of four screws or rods in a row are required in each reinforced area. Outside reinforced areas (shaded area in Figure E.1) the shear design shall fulfil the conditions for unreinforced members.

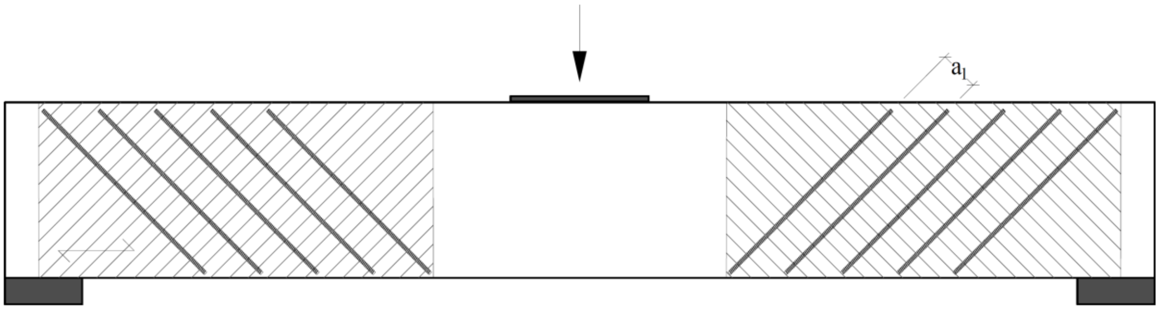

Figure E.1: Timber member with shear reinforcement; shaded areas: reinforced areas

Annex F
Tensile reinforcement perpendicular to grain

Timber members loaded by a connection force perpendicular to the grain

Unless specified otherwise in national provisions that apply at the installation site, the axial capacity of a reinforcement of a timber member loaded by a connection force perpendicular to the grain shall fulfil the following condition:

$$\frac{\left[1-3\cdot\alpha^{2}+2\cdot\alpha^{3}\right]\cdot F_{90,d}}{F_{ax,Rd}}\leq 1$$

Where

$F_{90,d}$ Design value of the force component perpendicular to the grain in N,

α = a/h

h = member depth in mm

$F_{ax,Rd}$ Minimum of the design values of the withdrawal capacity and the tensile capacity of the reinforcing screws or threaded rods where ℓ_{ef} is the smaller value of the penetration depth below or above the potential crack in N

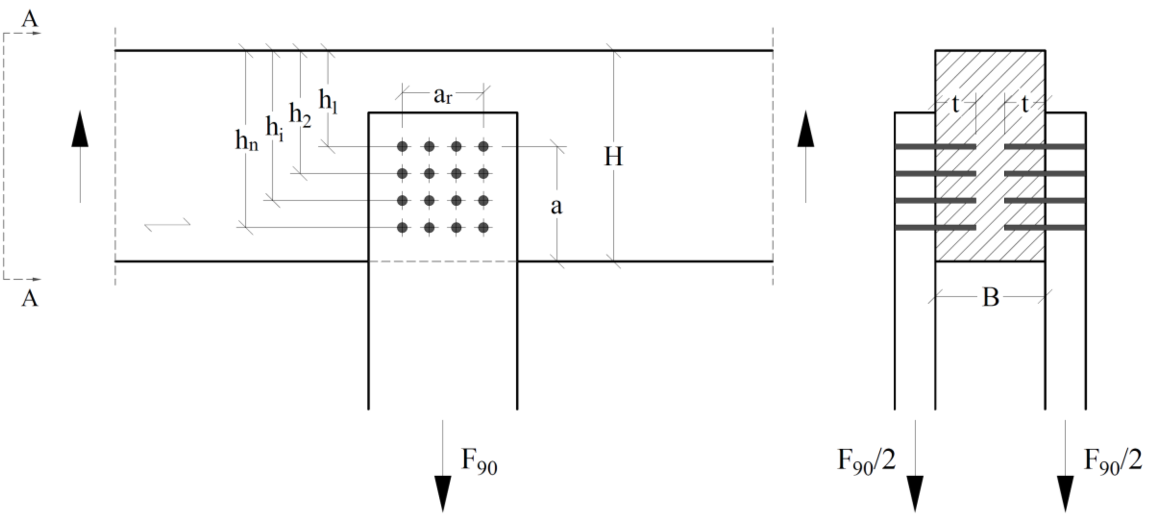

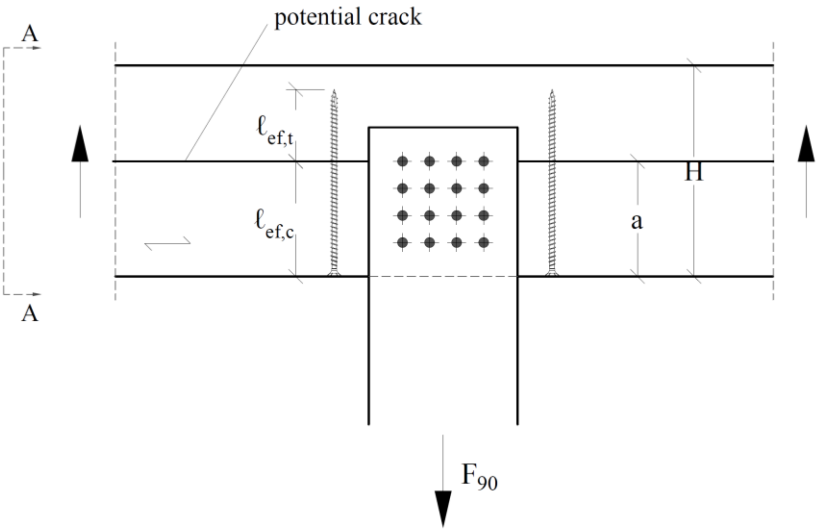

Notched beam supports

Unless specified otherwise in national provisions that apply at the installation site, the axial capacity of a reinforcement of a notched beam support shall fulfil the following condition:

$$\frac{1,3 \cdot V_d \cdot \left[3 \cdot (1-\alpha)^2 - 2 \cdot (1-\alpha)^3\right]}{F_{ax,Rd}} \leq 1$$

Where

V_d Design value of the shear force in N,

α = h/h_e

h = member depth in mm

$F_{ax,Rd}$ Minimum of the design values of the withdrawal capacity and the tensile capacity of the reinforcing screws or threaded rods where ℓ_{ef} is the smaller value of the penetration depth below or above the potential crack in N

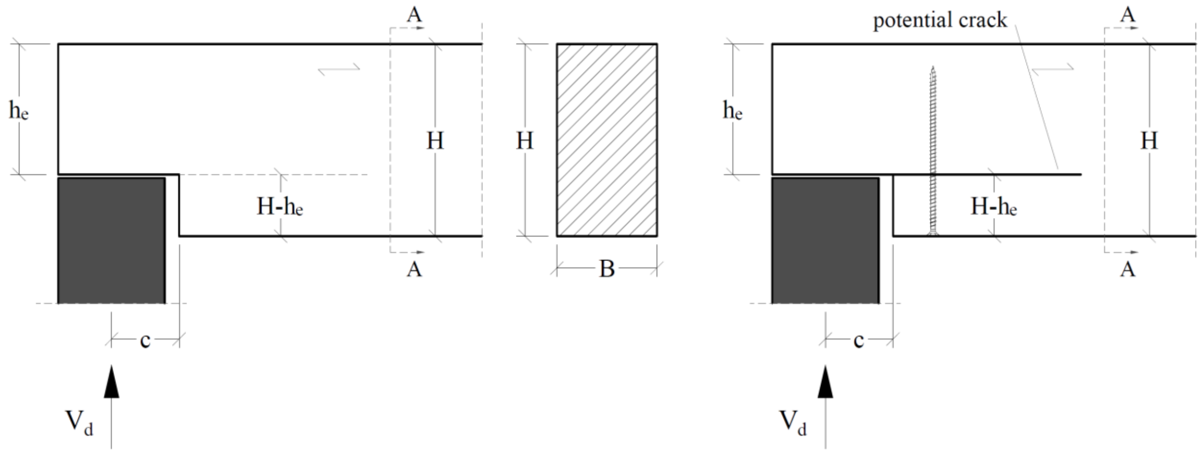

Beams with holes

Unless specified otherwise in national provisions that apply at the installation site, the axial capacity of a reinforcement of a hole in a beam shall fulfil the following condition:

$$\frac{F_{t,V,d} + F_{t,M,d}}{F_{ax,Rd}} \leq 1$$

Where

$F_{t,V,d}$ Design value of the force perpendicular to the grain due to shear force in N:

$$F_{t,V,d} = \frac{V_d \cdot h_d}{4 \cdot h} \cdot \left[3 - \frac{h_d^2}{h^2}\right]$$

V_d Design value of the member shear force at the hole end in N,

h = member depth in mm

h_d = hole depth for rectangular holes in mm

h_d = 70 % of hole diameter for circular holes in mm

$F_{t,M,d}$ Design value of the force perpendicular to the grain due to bending moment in N:

$$F_{t,M,d} = 0{,}008 \cdot \frac{M_d}{h_r}$$

M_d Design value of the member bending moment at the hole end in Nm,

h_r = min (h_{ro} ; h_{ru}) for rectangular holes in mm

h_r = min (h_{ro} ; h_{ru}) + 0,15·h_d for circular holes in mm

$F_{ax,Rd}$ Minimum of the design values of the withdrawal capacity and the tensile capacity of the reinforcing screws or threaded rods where ℓ_{ef} is the smaller value of the penetration depth below or above the potential crack in N.

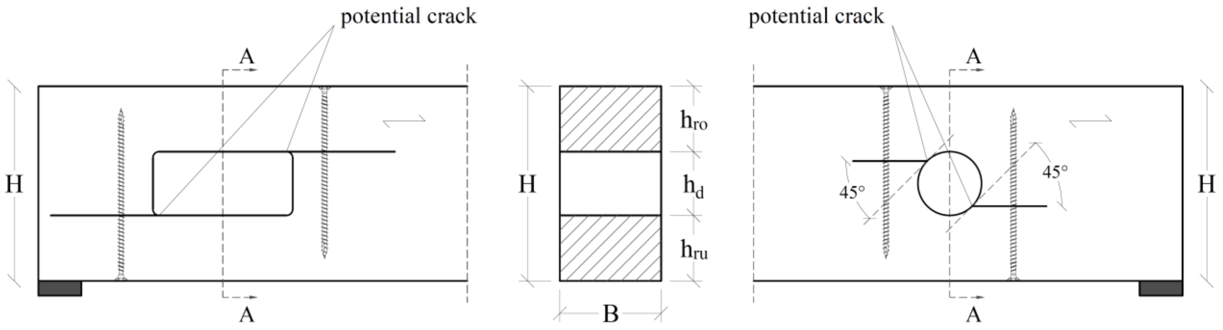

Annex G
Definition of angles in LVL

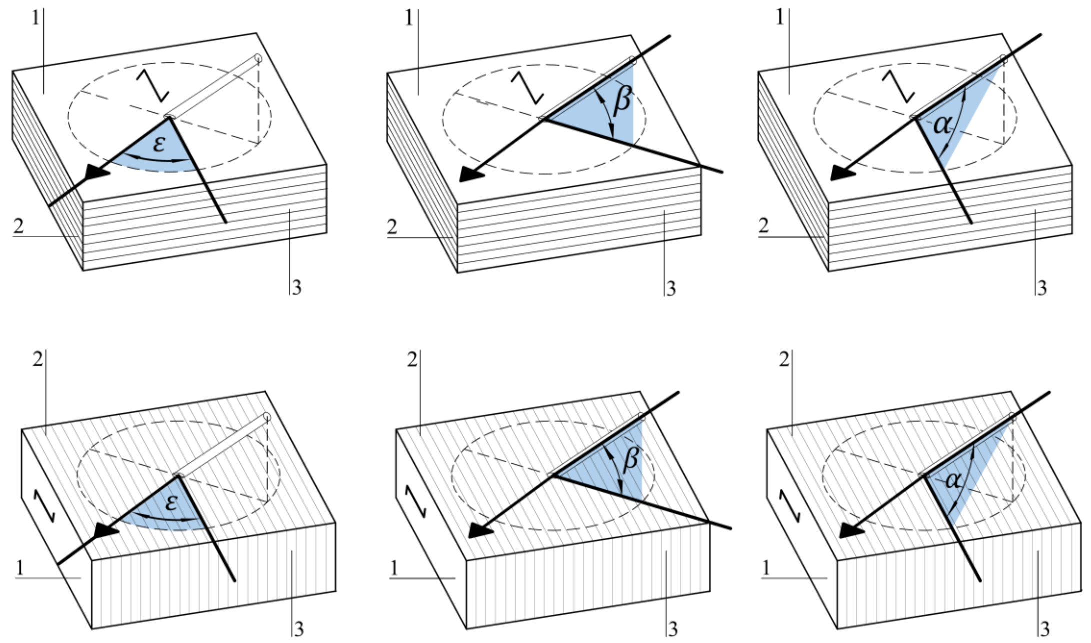

Note. Drawing is copyright Rotho Blaas

1. LVL’s wide side
2. LVL’s narrow side
3. LVL’s face side (lateral side consisting primarily of end grain)

ε: angle between load and grain direction (0° ≤ ε ≤ 90°)
α: angle between screw axis and grain direction
β: angle between screw axis and the LVL’s wide side (0° ≤ β ≤ 90°)

Tilgjengelig fra:

PRETEC

The Original Expansion Bolt for Structural Steel

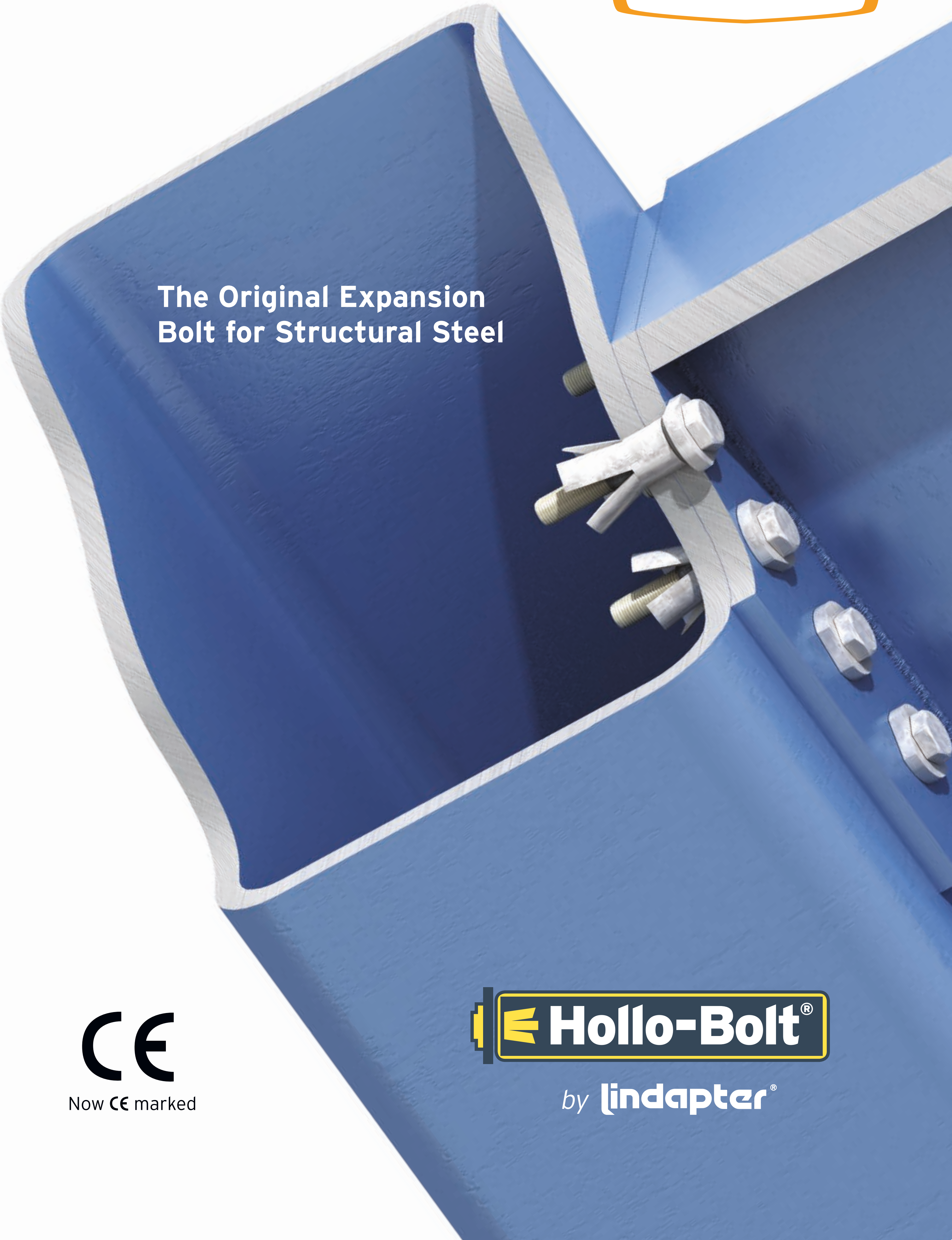

Now CE marked

Hollo-Bolt®

by Lindapter®

THE ORIGINAL EXPANSION BOLT FOR STRUCTURAL STEEL

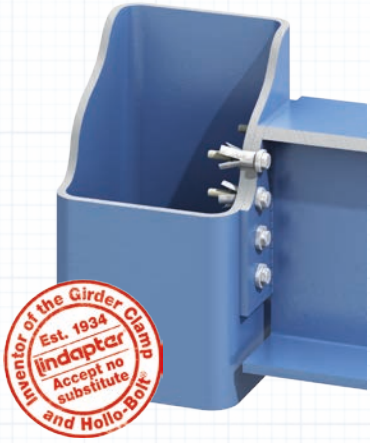

Lindapter®, the steelwork connection specialists, invented the Hollo-Bolt® as a fast, cost effective connection for Structural Hollow Section (SHS). The 'blind connection' technique requires installation access to only one side of the steel section for exceptional convenience. In comparison to alternative methods such as welding, a Hollo-Bolt connection can be quickly installed by simply inserting the fastener into pre-drilled holes and tightening with a torque wrench.

Since the Hollo-Bolt was first launched in 1995, the product range has been continuously developed to meet the diverse requirements of Structural Engineers and Architects, with enhancements including new head types, lengths, finishes and performance improvements.

10 REASONS TO USE HOLLO-BOLT

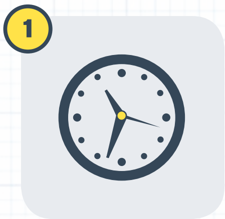

Fast, time saving installation

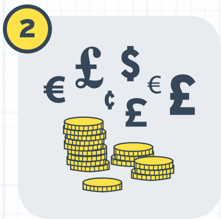

Lower labour costs

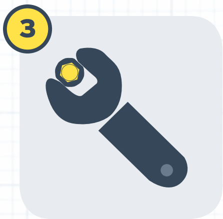

Easy to install from just one side

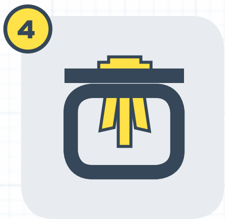

For SHS and other hollow sections

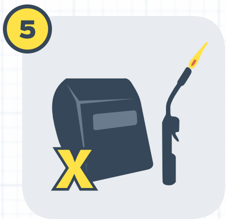

No need to weld, no hot work permits

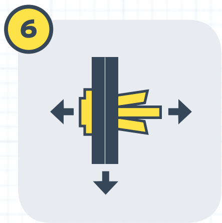

High resistance to shear and tension

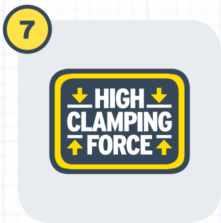

Hollo-Bolt (HCF) for 3x Clamping Force

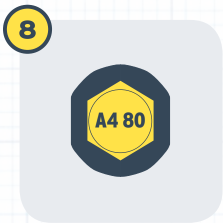

Various corrosion resistant options

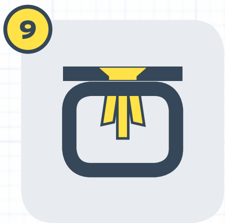

Aesthetically pleasing connections

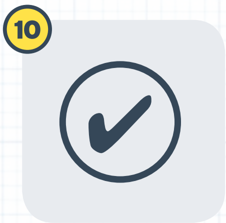

Independently approved product performance

INTERNATIONAL RECOGNITION

The British Constructional Steelwork Association (BCSA) and Steel Construction Institute (SCI) include the Hollo-Bolt as a structural connection in the design guide 'Joints in Steel Construction'[(a)]. For more information, please see pages 22 & 23.

The American Institute of Steel Construction (AISC) also recognises the Hollo-Bolt in the Steel Construction Manual[(b)]. Engineers and Architects around the world specify the Hollo-Bolt as a time and labour saving method of connecting structural steel frames or securing almost anything to SHS (see page 8-19 for project examples).

(a) (b)

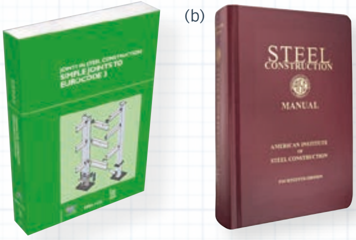

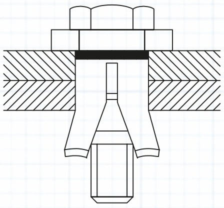

Drawing from 'Joints in Steel Construction: Simple Joints to Eurocode 3'
Cover (a) & drawing Copyright © BCSA / SCI. Cover (b) Copyright © AISC. Reprinted with permission. All rights reserved.

CONTENTS

Why use a Hollo-Bolt?........................ 4
Hollo-Bolt Options.............................. 5
Expansion Mechanism......................... 6
Clamping Force.................................... 7
Project Experience.............................. 8
Installation Guidance......................... 20
Design Data.. 22
FAQs.. 24
Lindapter Service & Products............ 26

TYPICAL HOLLO-BOLT APPLICATIONS

- Structural Frames
- Glazing and Roofs
- Staircases and Handrails
- Balconies and Canopies
- Façades and Cladding
- Towers and Masts

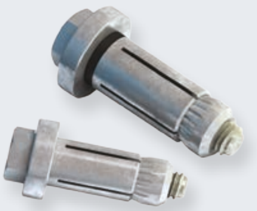

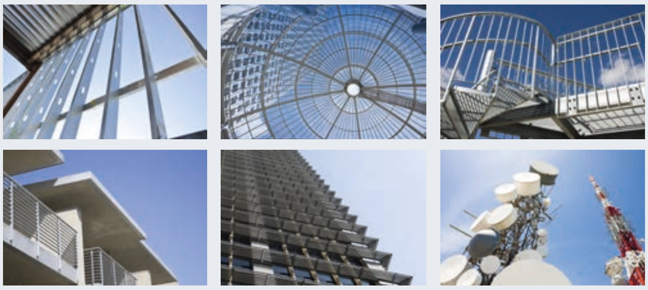

APPROVALS

CE Marking provides additional security for Engineers, Specifiers and Contractors by demonstrating Lindapter's commitment to quality manufacturing and transparency of product performance.

Lindapter's Factory Production Control System is independently evaluated to ensure only premium quality products leave the production line, whilst product performance is scrutinised under comprehensive testing programmes and published in European Technical Approvals (ETAs). In this brochure you will find the Eurocode 3 Characteristic Values (as published in the official ETA-10/0416) and also standard Safe Working Loads for resistance to tensile and shear loading. For more information on CE Marking, please refer to the website: www.lindapter.com/about/ce

DIBt - Deutsches Institut für Bautechnik is a respected organisation that approves construction products for use in Structural and Civil Engineering industries in Germany.

TÜV are the certifying authority for safety, quality and environmental protection in Germany. Hollo-Bolts are produced under strict quality and environment management systems to ensure consistently high manufacturing standards across the range.

Disclaimer
Lindapter International supplies components in good faith, on the assumption that customers fully understand the loadings, safety factors and physical parameters of the products involved. Customers or users who are unaware or unsure of any details should refer to Lindapter International before use. Responsibility for loss, damage or other consequences of misuse cannot be accepted. Lindapter makes every effort to ensure that technical specifications and other product descriptions are correct. 'Specification' shall mean the specification (relating to the use of the materials) set out in the quotation given by the Seller to the Buyer. Responsibility for errors or omissions cannot be accepted. All dimensions stated are subject to production tolerances – if in doubt please check with Lindapter.

Applications
All the applications featured in the brochure are based on real projects. For more information and further examples visit: www.hollo-bolt.com

© Lindapter International 2013
Lindapter, Hollo-Bolt and Lindibolt are registered trademarks. In the interests of improving the quality and performance of Lindapter products, we reserve the right to make specification changes without prior notice.

PRETEC Pretec AS - autorisert distributør: +47 69102460 post@pretec.no www.pretec.no

ALTERNATIVE CONNECTION METHODS

WELDING

- ✖ Hot work permit required
- ✖ Skilled labour needed
- ✖ Requires power/consumables

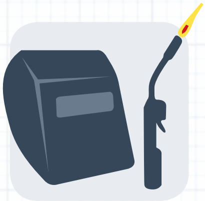

V

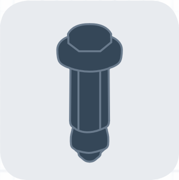

HOLLO-BOLT

✔ A safe and permanent connection that is quick to install using hand tools.

THROUGH-BOLTING

- ✖ Inappropriate for larger SHS
- ✖ Strength of connection not guaranteed
- ✖ Risk of SHS deformation

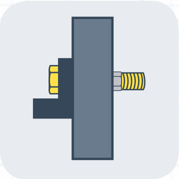

V

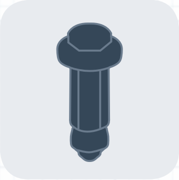

HOLLO-BOLT

✔ A reliable high strength fixing, supported by independently approved Safe Working Loads.

BRACKETS & STRAPPING

- ✖ Unsightly finish
- ✖ Time consuming installation
- ✖ Low capacity in friction

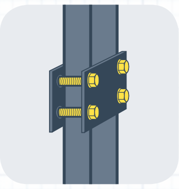

V

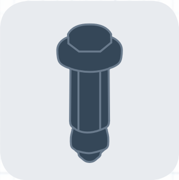

HOLLO-BOLT

✔ Architectural options include the Hollo-Bolt Flush Fit for a very discreet connection.

CUTTING ACCESS HOLES

- ✖ Expensive & time consuming
- ✖ Unsuitable for structural connections
- ✖ Defeats any architectural benefit of SHS

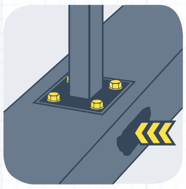

V

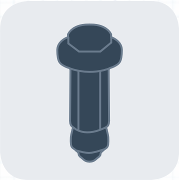

HOLLO-BOLT

✔ A neat, labour saving SHS connection, suitable for structural applications.

SIMPLE HOLLO-BOLT INSTALLATION

Project Example: Salt River Fields Stadium, Arizona, USA

1. Deliver pre-drilled steel to site.

2. Align the pre-drilled fixture and insert the Hollo-Bolt.

3. Using a torque wrench, tighten the Hollo-Bolt to the recommended torque.

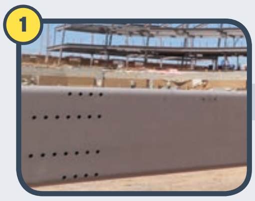

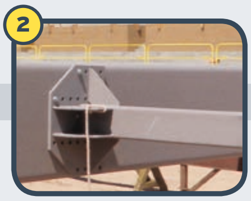

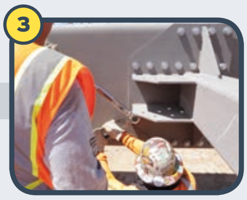

HEAD VARIANTS

The Hollo-Bolt is available in three head types to complement diverse architectural designs. Lindapter also has the capability to produce customised Hollo-Bolts; a service passionately referred to as ‘Engineered Solutions’.

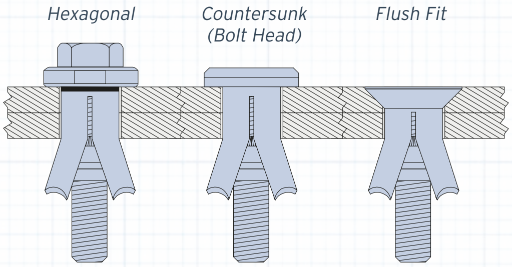

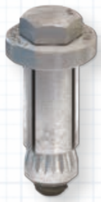

HEXAGONAL

Visible protrusion: Standard

Description: The Hollo-Bolt collar and head of the Grade 8.8 bolt are evident above the surface of the steel section. This head variant is the usual choice for the majority of SHS connections, or where architects favour an ‘industrial’ look.

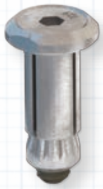

COUNTERSUNK (BOLT HEAD)

Visible protrusion: Minimal

Description: This discreet midway option has a smaller protrusion for the perfect balance of appearance and convenience, and features a Grade 10.9 countersunk bolt with a special collar designed to accommodate the entire bolt head. Drilling countersunk holes in the steel section is not required.

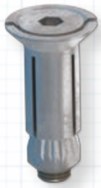

FLUSH FIT

Visible protrusion: Zero

Description: The innovative Flush Fit Hollo-Bolt is entirely concealed within a drilled countersunk hole once installed, leaving no protrusion above the surface of the steel section - the perfect solution for architects!

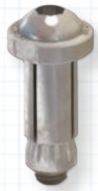

ENGINEERED SOLUTIONS

Visible protrusion: Customised

Description: For the rare connection requirement that an off-the-shelf Hollo-Bolt cannot fulfil, Lindapter’s Research and Development Facility has the capability to design and manufacture custom connection solutions. Just one example of a custom Hollo-Bolt is the tamperproof Button Security Head variant, developed for use in prisons.

CORROSION RESISTANCE

The Hollo-Bolt is available in a series of protective coatings and materials to provide a customisable yet off-the-shelf connection solution. See right for availability:

** Sheraplex is an advanced coating designed for intricately shaped and precision machined components. The two-stage treatment process first involves Sheradising (Zinc coating), then secondly applying an organic barrier layer. The resulting surface has a smooth matt grey finish that provides high corrosion resistance.*

		Hex Head	Countersunk	Flush Fit
	Bright Zinc Plated & JS500	✓	✓	✓
	Hot Dip Galvanised	✓		
	Sheraplex*	✓	✓	✓
	Stainless Steel (Grade 316)	✓	✓	✓
	M8	✓	✓	✓
	M10	✓	✓	✓
	M12	✓	✓	✓
HCF	M16	✓	✓	
HCF	M20	✓		

Sizes M16 and M20, known as the Hollo-Bolt (HCF), feature a patented **High Clamping Force** mechanism to produce three times more clamping force than the same sized product without the mechanism. The significance of clamping force and the superior performance of Lindapter’s unique Hollo-Bolt (HCF) is illustrated on page 6.

PRETEC Pretec AS - autorisert distributør: +47 69102460 post@pretec.no www.pretec.no

HOLLO-BOLT & HOLLO-BOLT (HCF)

The Hollo-Bolt is available in two versions: the original 3-part design for general hollow section connections and the larger sized 5-part **High Clamping Force** (HCF) version, for higher strength structural connections.

3-PART HOLLO-BOLT

M8 **M10** **M12**

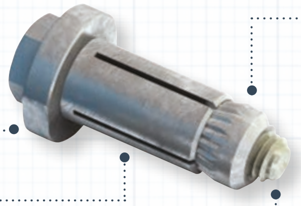

→ ← = Clamping Force

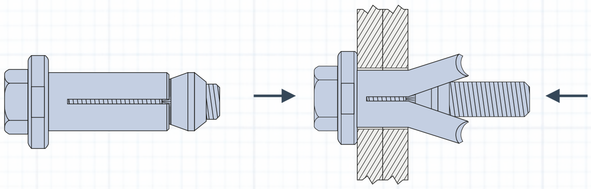

A typical connection is made by inserting the Hollo-Bolt into the pre-drilled holes of the fixture and hollow section. As the bolt head is tightened, the cone is pulled up the bolt thread, causing the legs of the sleeve to expand until the cone locks the sleeve against the inner wall of the hollow section.

At full tightening torque, a clamping action is set up between the fixture and the steel section to form a secure connection. Once installed, only the head and collar are visible.

5-PART HOLLO-BOLT (HCF)

HIGH CLAMPING FORCE

M16 **M20**

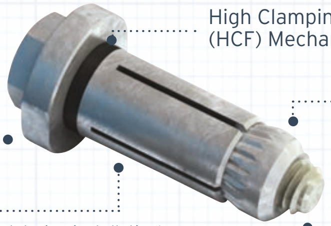

→ ← = Clamping Force

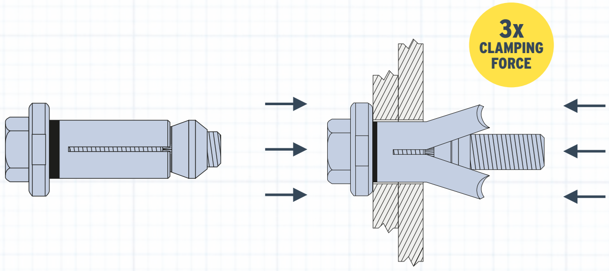

Working closely with Structural Engineers and Steel Fabricators, Lindapter identified the need for the larger M16 and M20 Hollo-Bolts to have an increased clamping force suitable for higher strength structural connections. Research and Development led to the invention of the patented 5-part design, optimised for superior performance.

The High Clamping Force (HCF) mechanism consists of a special washer that 'compresses' to significantly increase clamping force between the fixture and hollow section, when compared to a 3-part product of the same size, thereby reducing displacement.

HOLLO-BOLT (HCF)
TYPICAL PERFORMANCE INCREASE

With HCF Mechanism
5-Part Design
› Hot Dip Galvanised, Size 2

Without HCF Mechanism
3-Part Design
› Hot Dip Galvanised, Size 2

HIGH CLAMPING FORCE

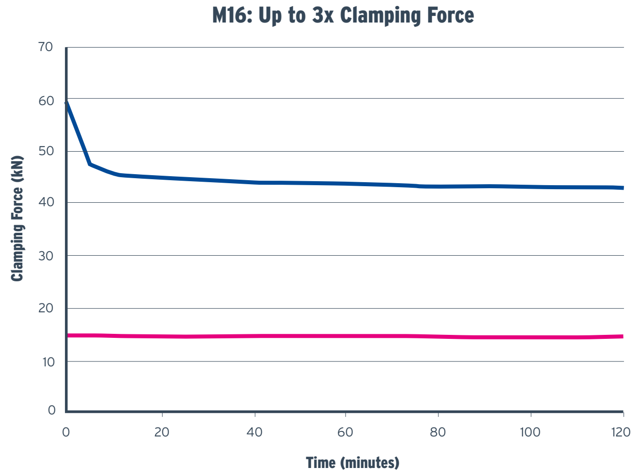

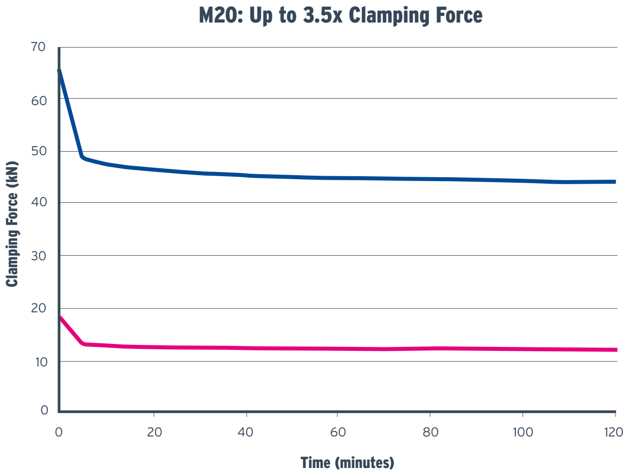

CLAMPING FORCE

As with any structural bolt, immediately after installation the bolt relaxes until a typical clamping force is reached. The typical clamping force of the Hollo-Bolt (HCF) is over three times higher than the same sized product without the HCF mechanism. This results in a more secure connection and a greater force that has to be overcome before displacement begins.

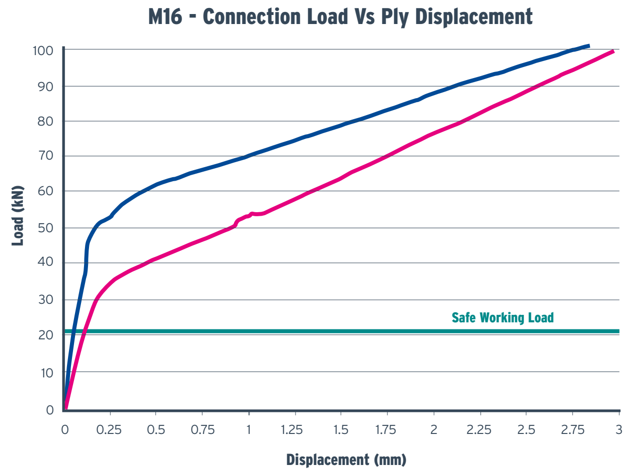

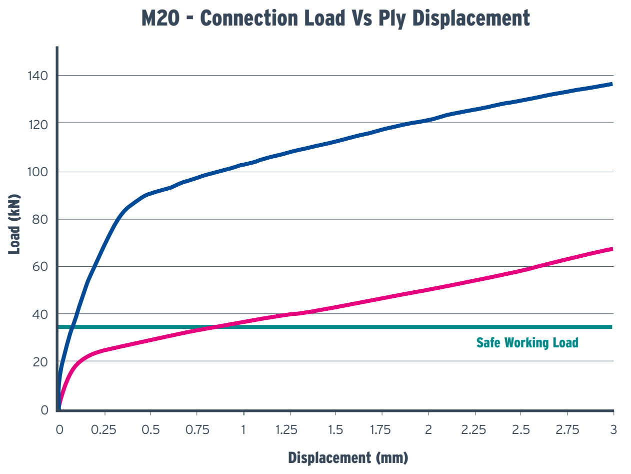

DISPLACEMENT

The significance of increased clamping force is shown in the graphs above. The blue curve demonstrates the superior performance of the Hollo-Bolt (HCF) in contrast to M16 & M20 sized products without Lindapter's patented HCF mechanism (i.e. the 3-part design in these larger sizes). When using the Hollo-Bolt (HCF), displacement (movement in the connection) is minimised at Safe Working Load for a safer and more secure connection.

SOCIÉTÉ GÉNÉRALE TRADING OFFICE

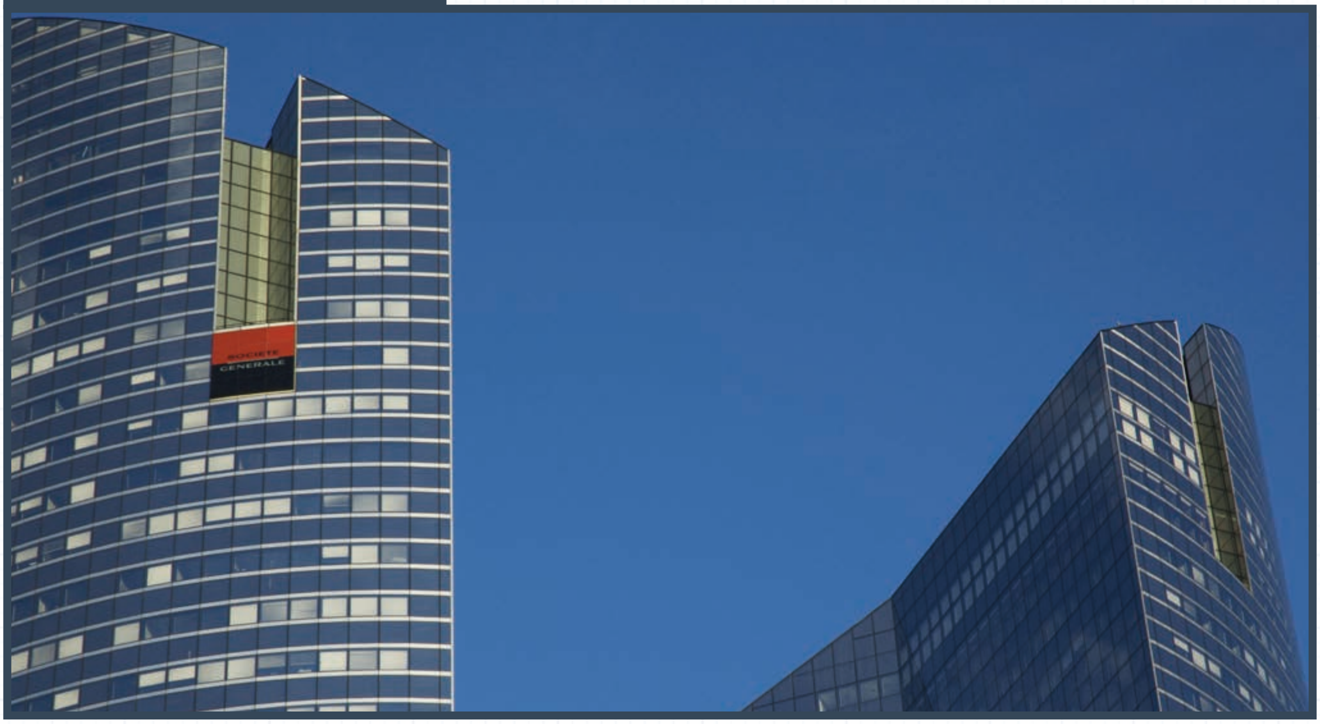

APPLICATION

Attaching façade glazing to the building's structural steel frame

LOCATION

Paris, France

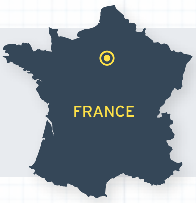

HEAD TYPE

Hexagonal

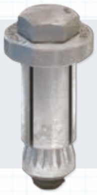

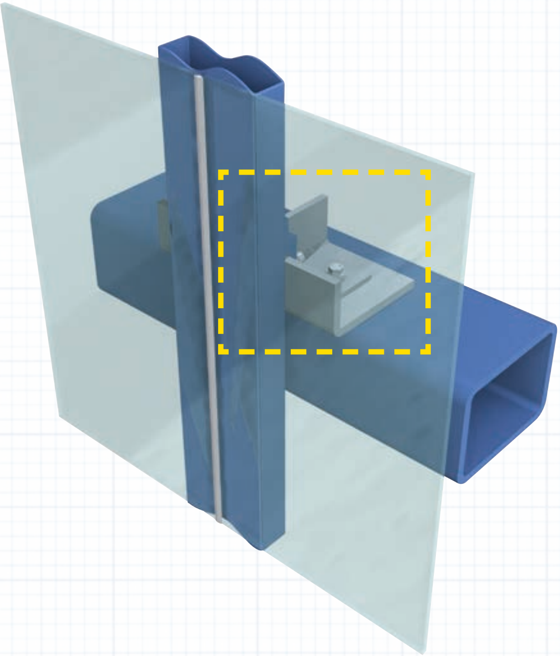

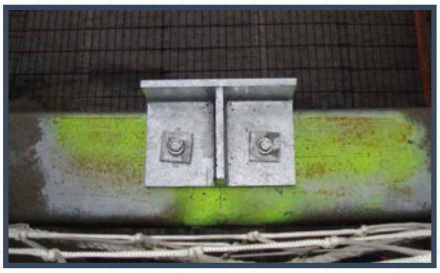

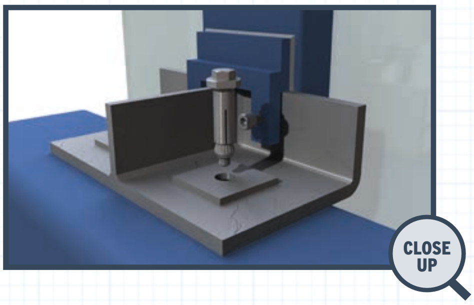

MANCHESTER MAGISTRATES COURT

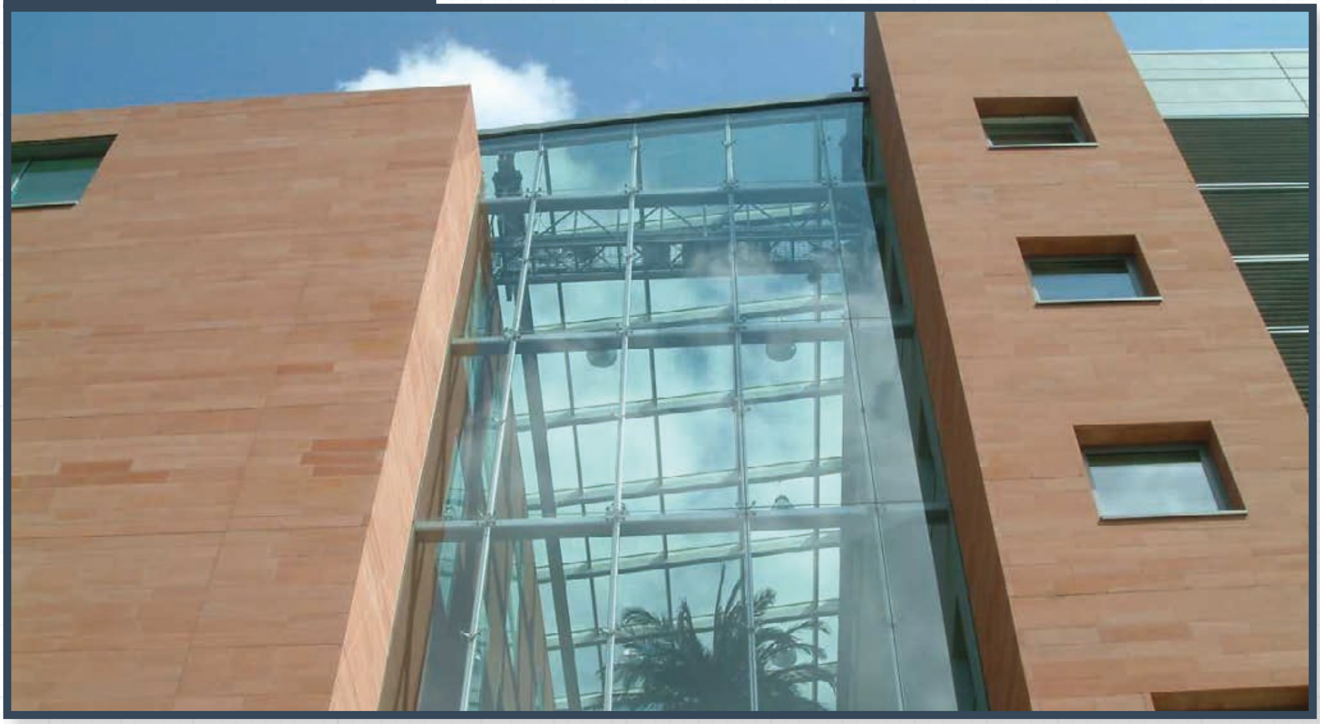

APPLICATION

Connecting façade spider brackets to structural hollow section

LOCATION

Manchester, UK

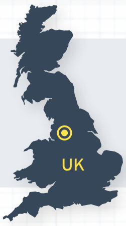

HEAD TYPE

Hexagonal

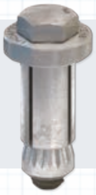

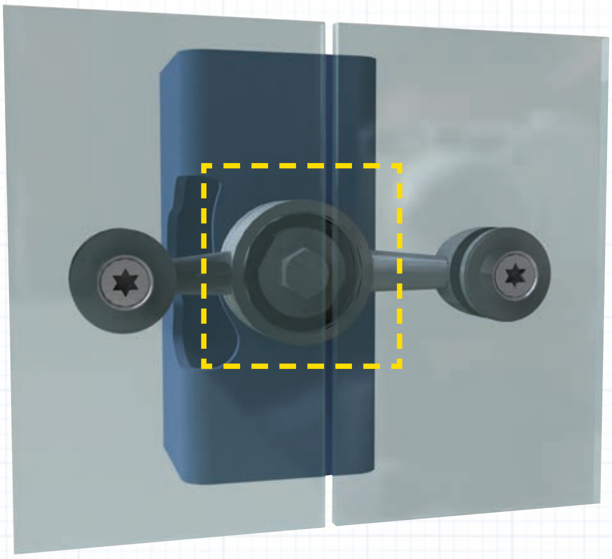

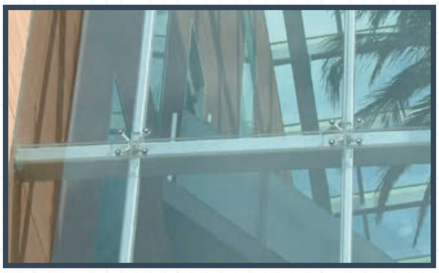

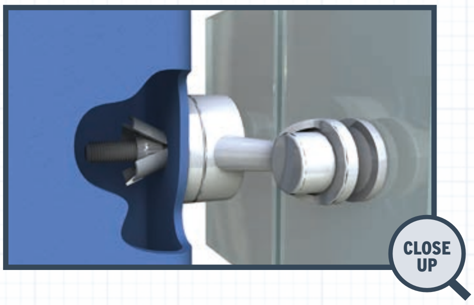

HISTORY MUSEUM

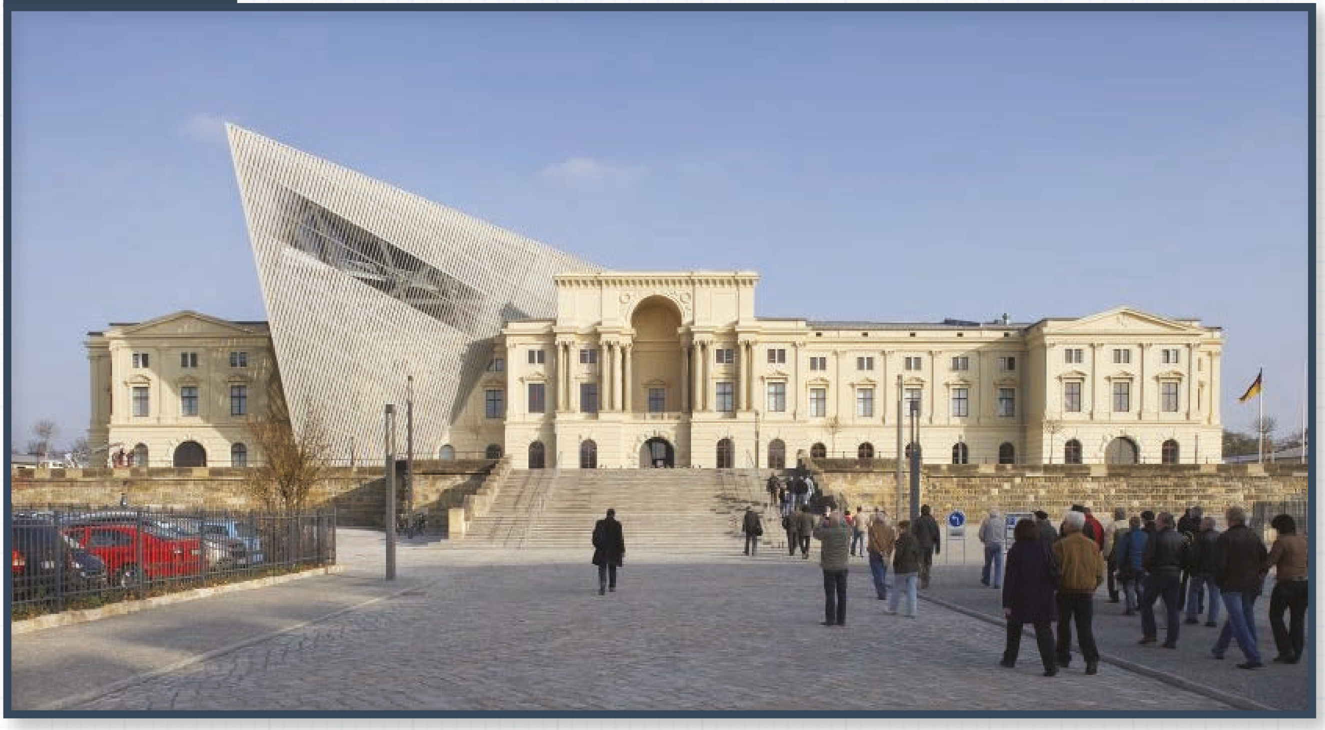

© Hufton & Crow

APPLICATION

Connecting perforated steel cladding to structural hollow section

LOCATION

Dresden, Germany

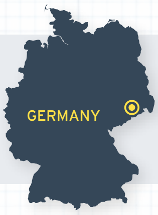

HEAD TYPE

Hexagonal

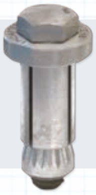

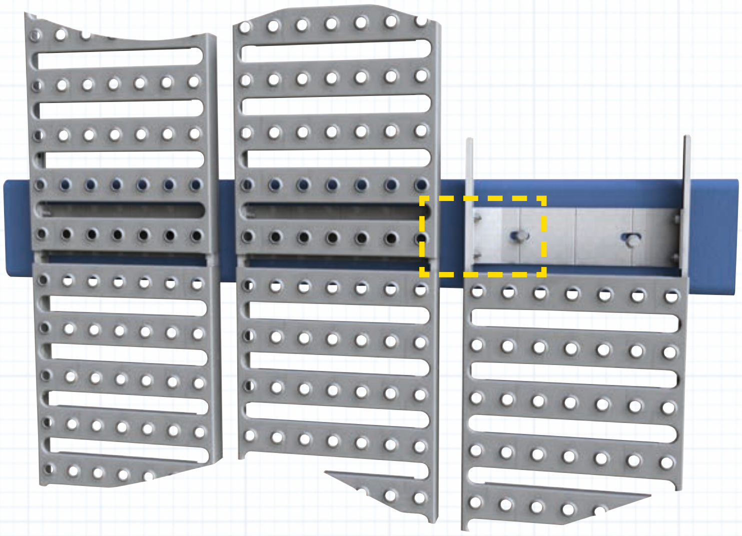

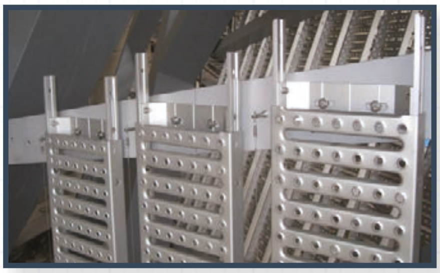

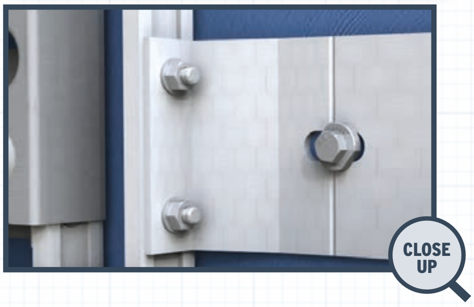

THAMES EXCHANGE

Image courtesy of OAG UK

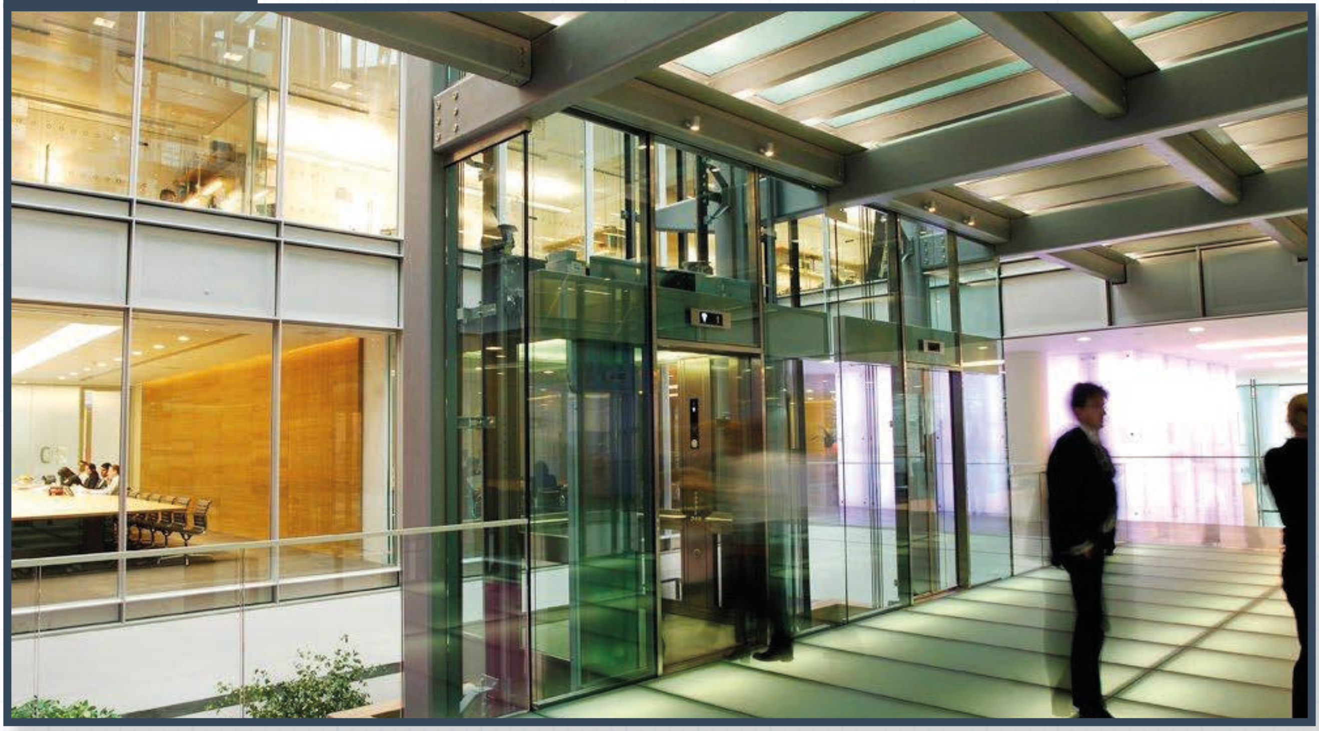

APPLICATION

Structural connections of the elevator glazing frame

LOCATION

London, UK

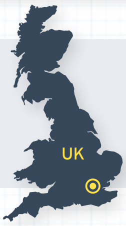

HEAD TYPE

Countersunk (Bolt Head)

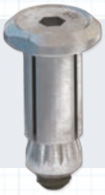

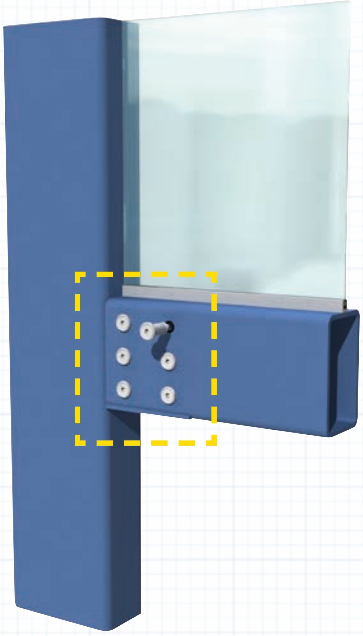

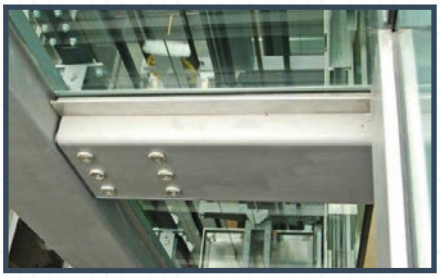

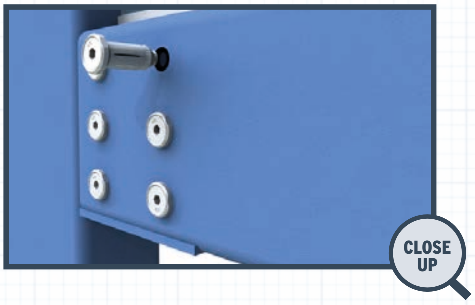

ROSE HILL CONSERVATORY

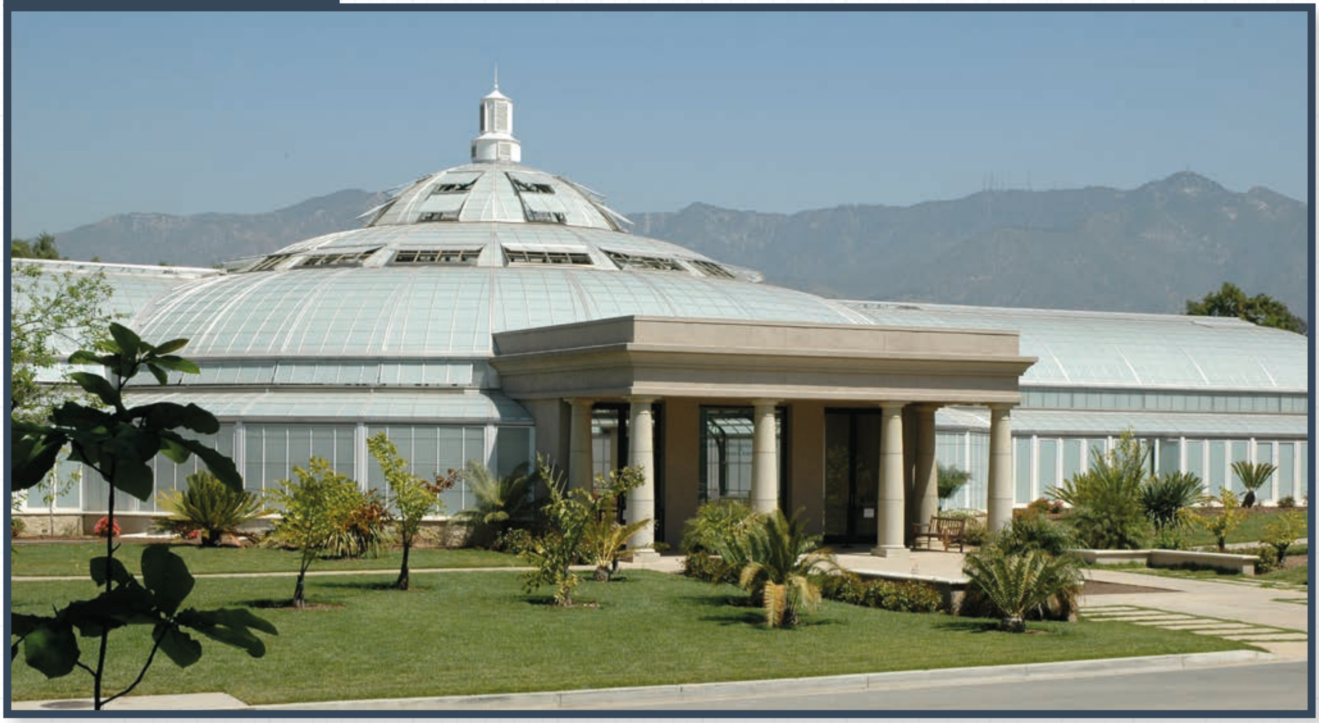

Image: The Huntington Library, Art Collections and Botanical Gardens

APPLICATION

Structural steel truss connections of the conservatory frame

LOCATION

San Marino, CA, USA

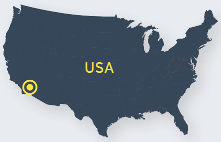

HEAD TYPE

Hexagonal

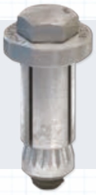

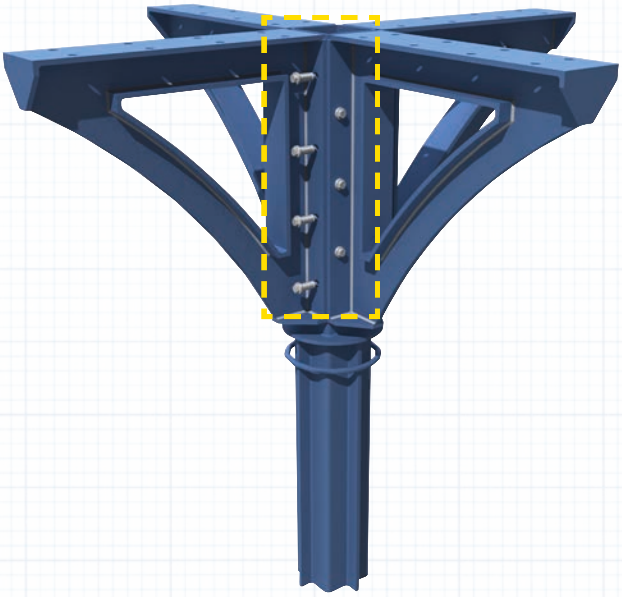

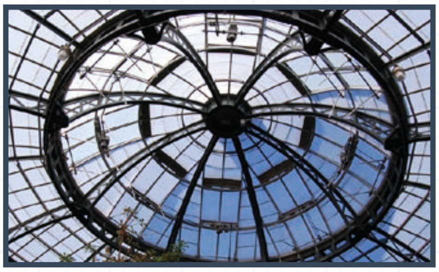

Image: Far Out Flora (faroutflora.com)

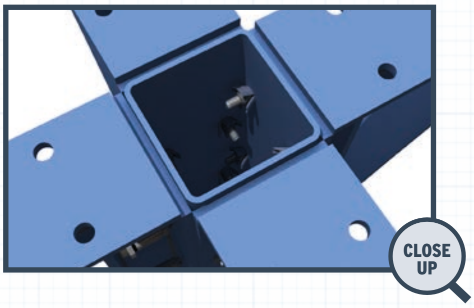

HAFEN CITY

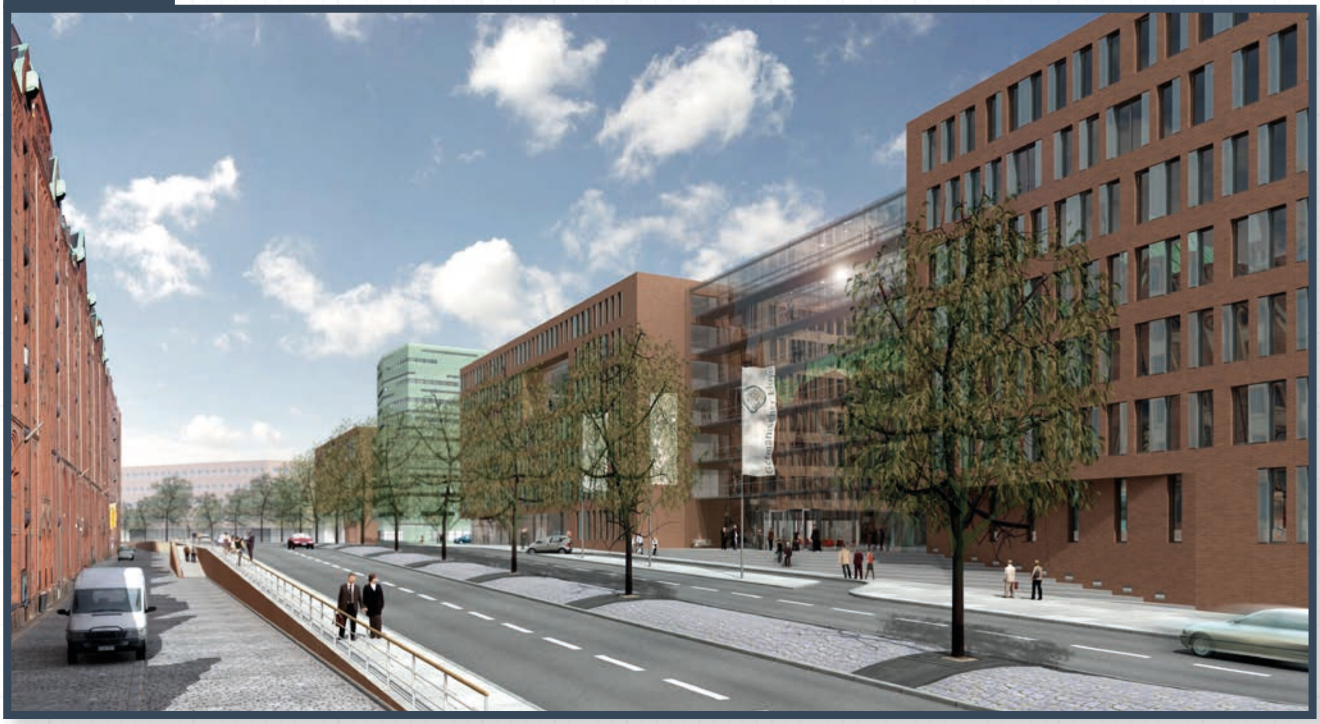

Image: Quantum Immobilien AG

APPLICATION

Connecting the glazing support frame and roof

LOCATION

Hamburg, Germany

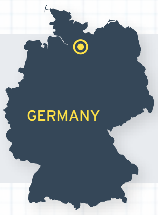

HEAD TYPE

Flush Fit

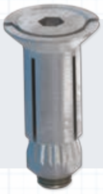

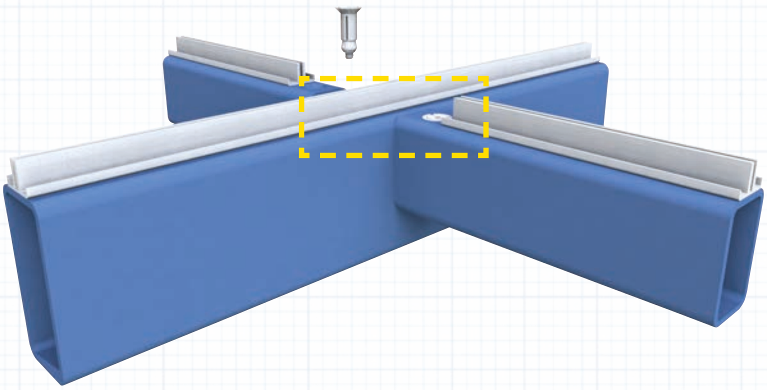

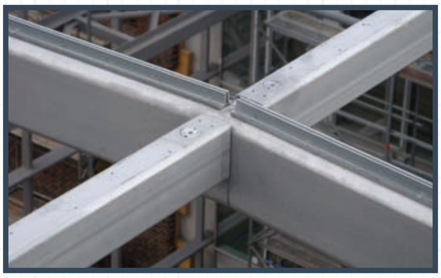

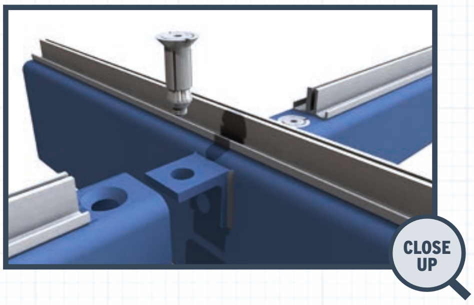

KIMMEL CENTER

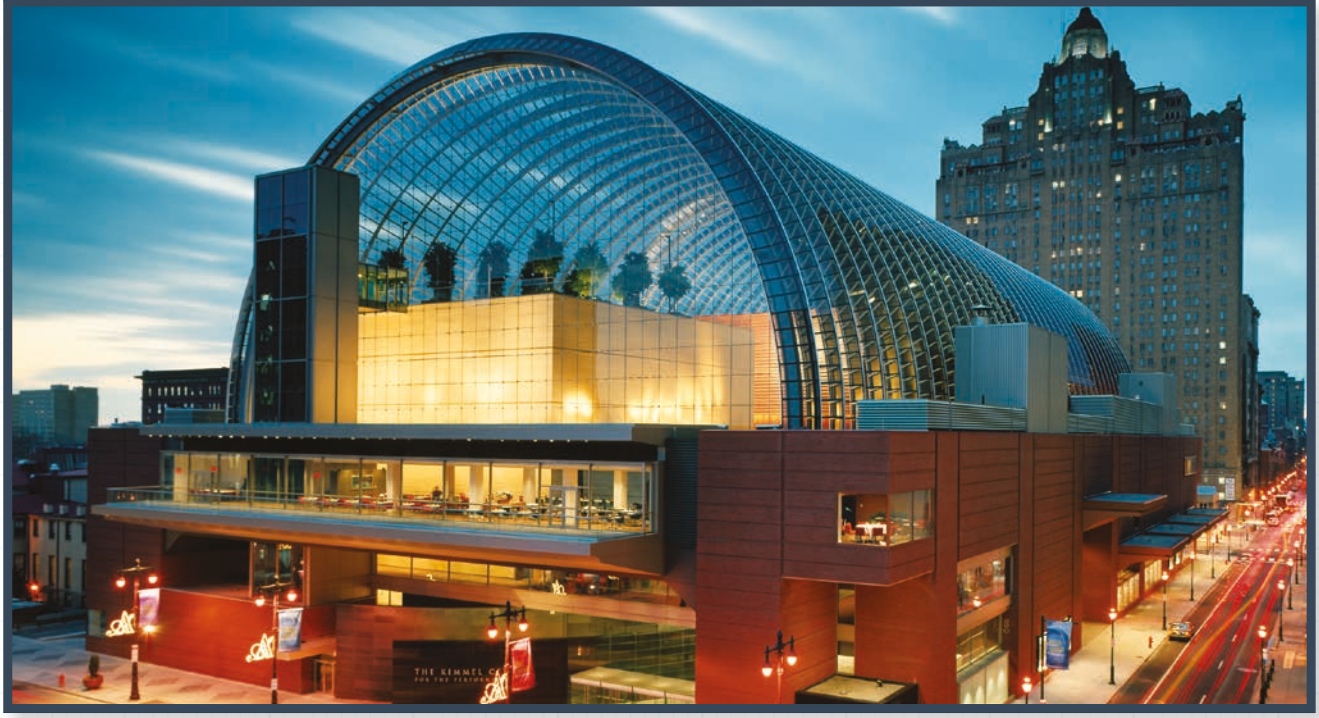

Image: Jeff Goldberg/Esto, courtesy of Kimmel Center

APPLICATION

Connecting the barrel-vault roof

LOCATION

Philadelphia, PA, USA

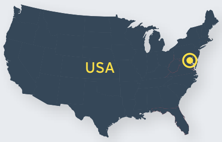

HEAD TYPE

Hexagonal

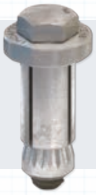

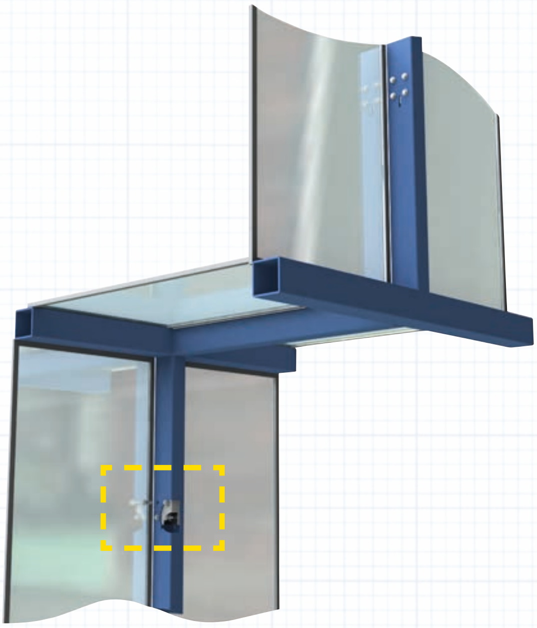

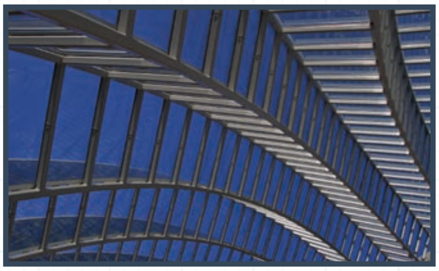

Image: R. Bradley Maule

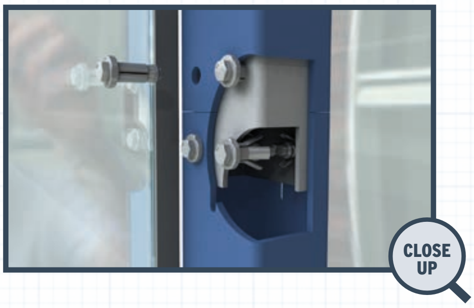

SALT RIVER FIELDS STADIUM

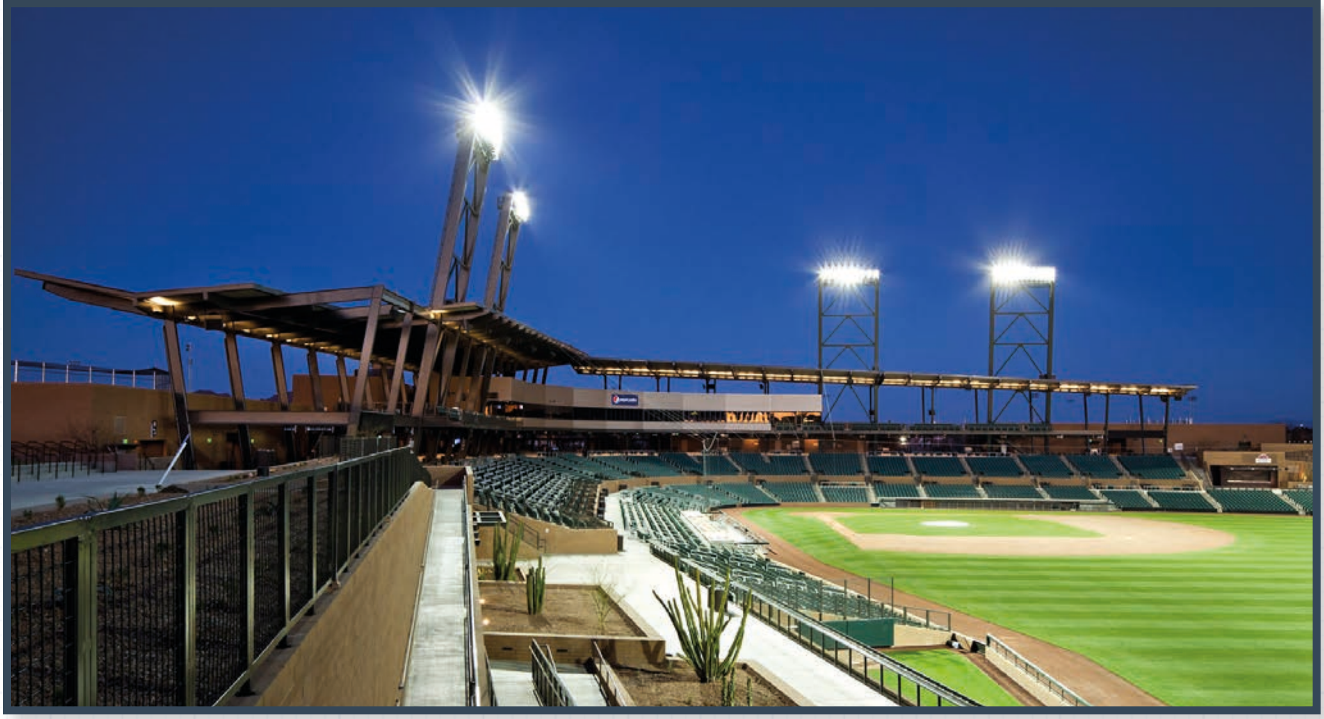

APPLICATION

SHS connections for the floodlighting frame

LOCATION

Scottsdale, AZ, USA

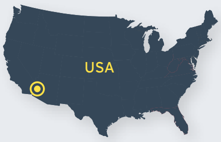

HEAD TYPE

Hexagonal

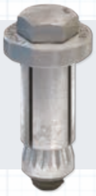

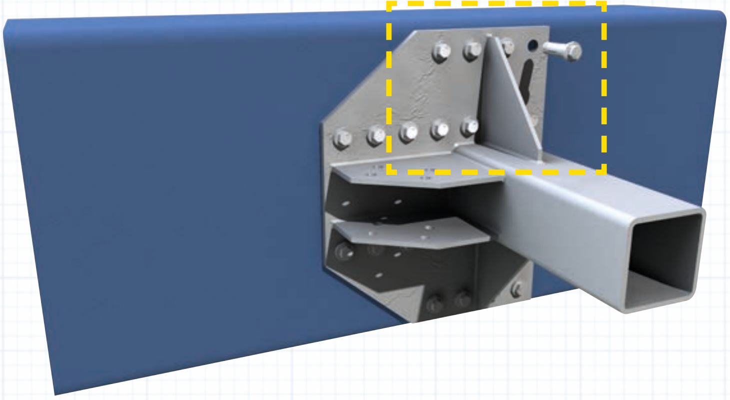

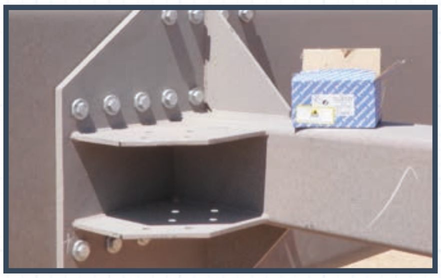

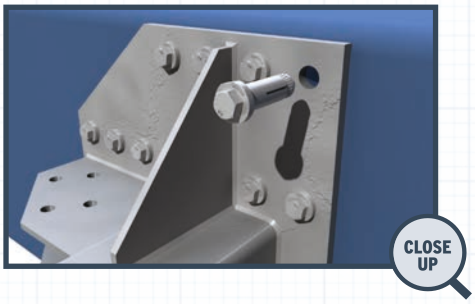

BMW WORLD

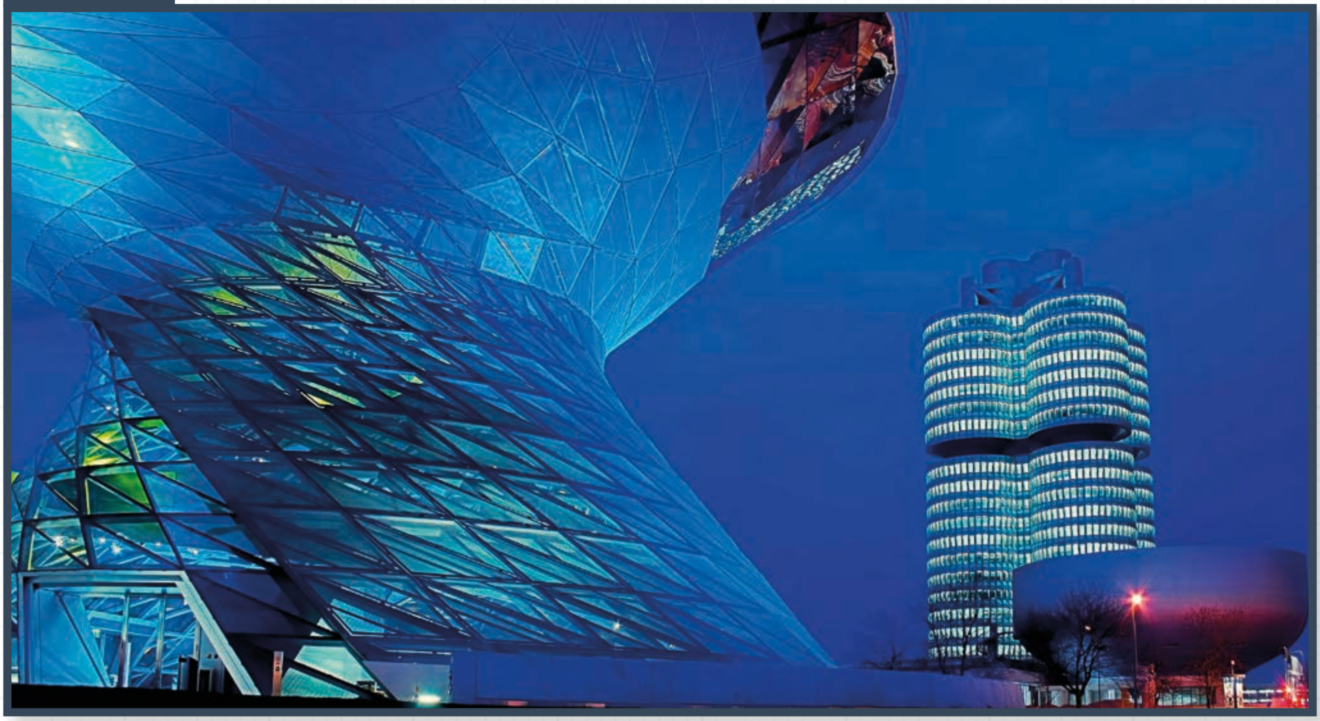

APPLICATION

Connection of mounting points for solar panels

LOCATION

Munich, Germany

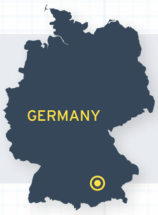

HEAD TYPE

Hexagonal

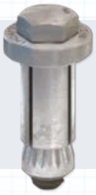

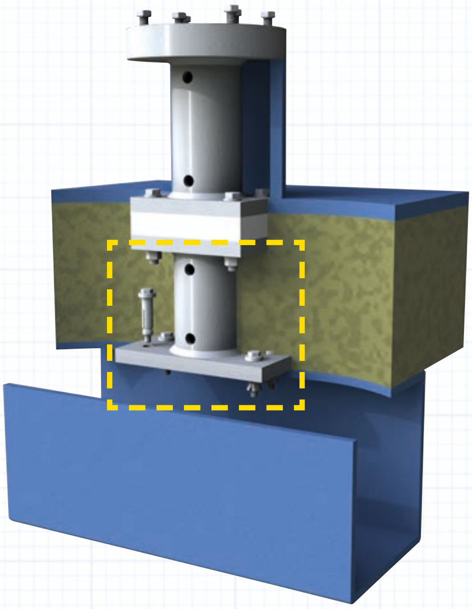

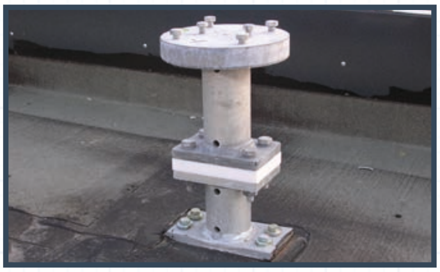

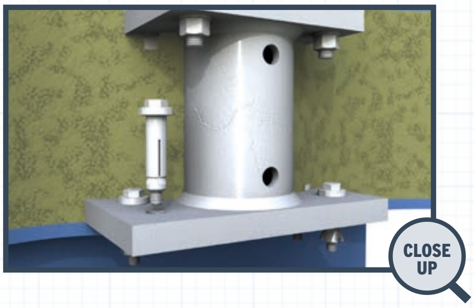

SNORRE OFFSHORE

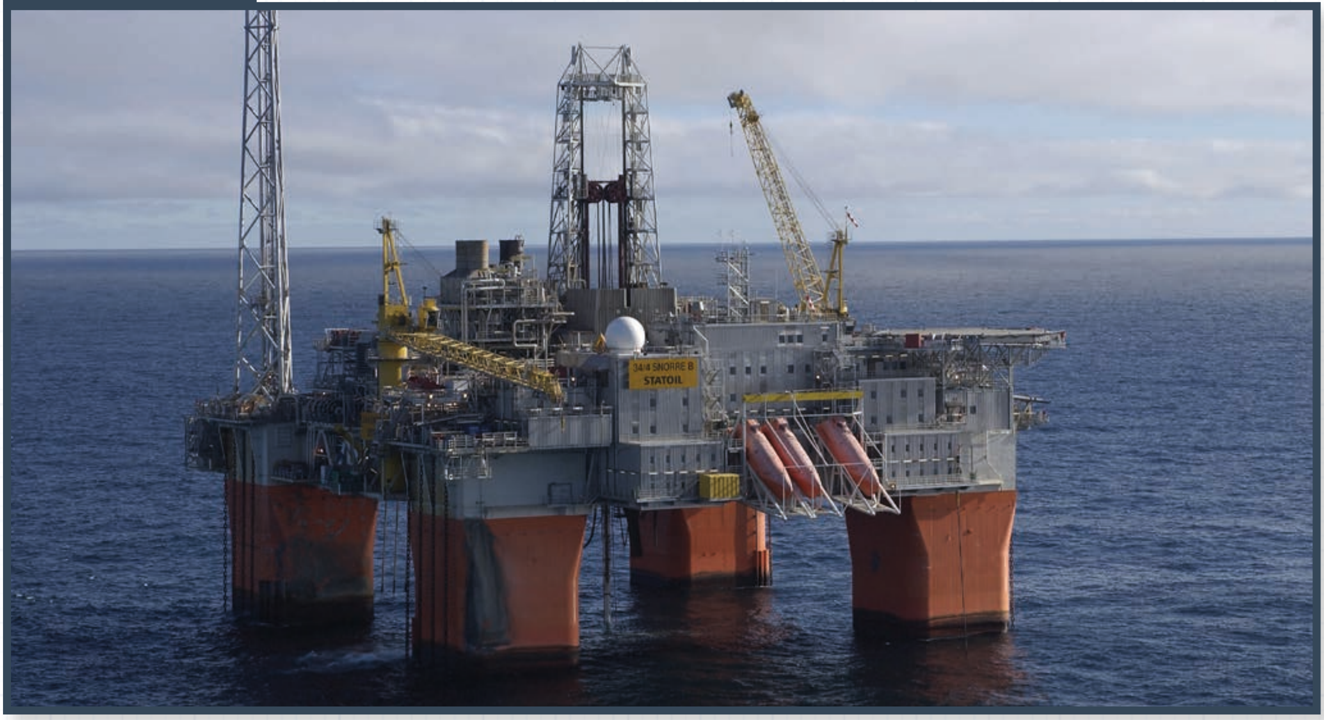

APPLICATION

Securing handrails to fabricated hollow section

LOCATION

Norwegian
North Sea

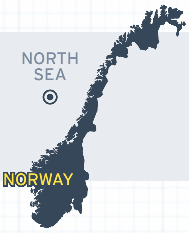

HEAD TYPE

Hexagonal

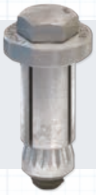

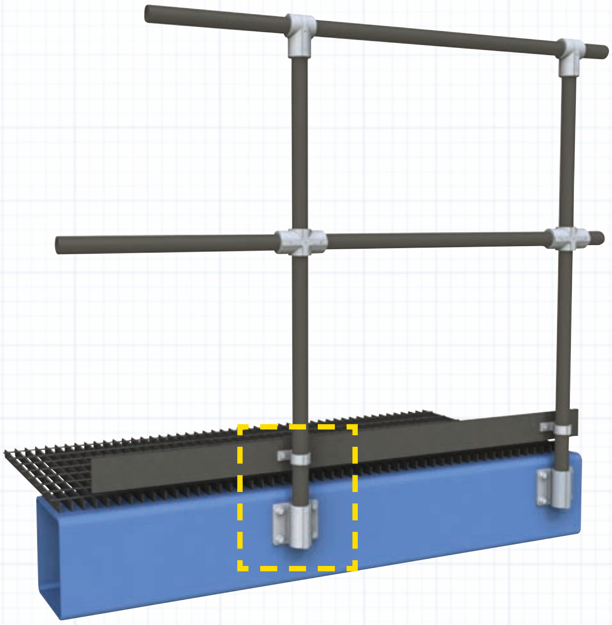

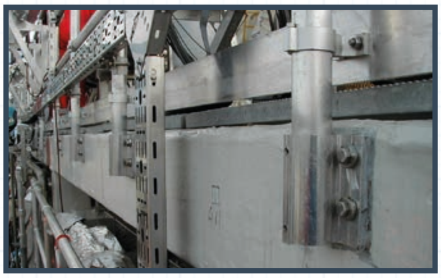

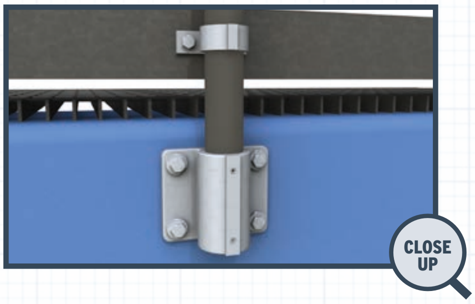

PHOENIX METRO LIGHT RAIL

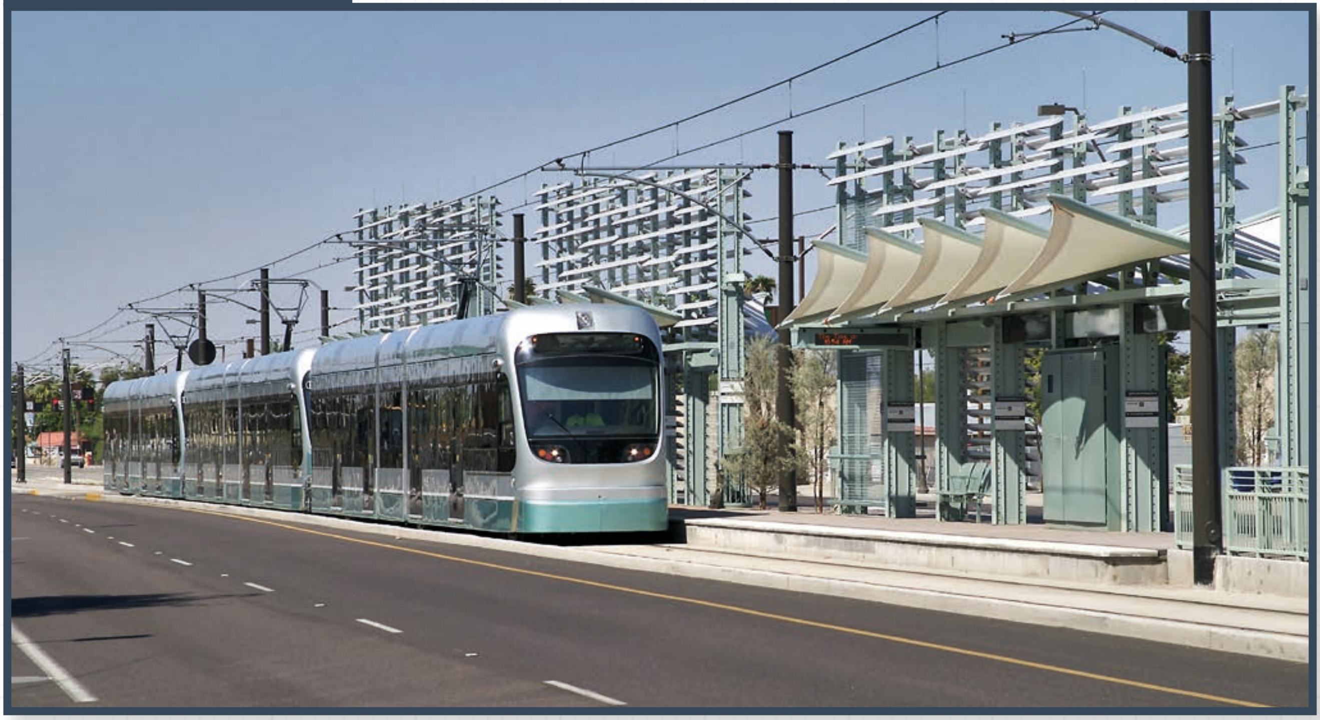

APPLICATION

Securing station signage and seating to structural frames

LOCATION

Phoenix, AZ, USA

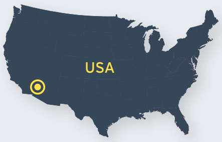

HEAD TYPE

Button Security

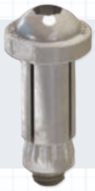

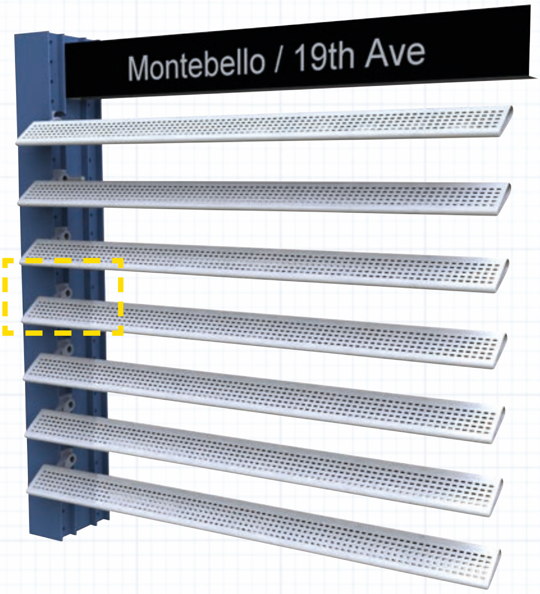

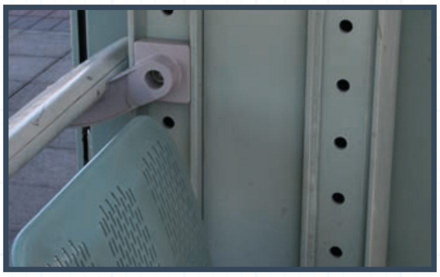

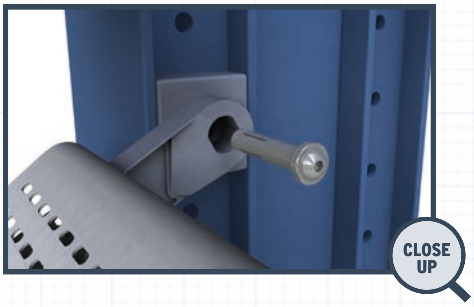

TELSTRA STADIUM / OLYMPIC STADIUM

Image: Brian Parcy

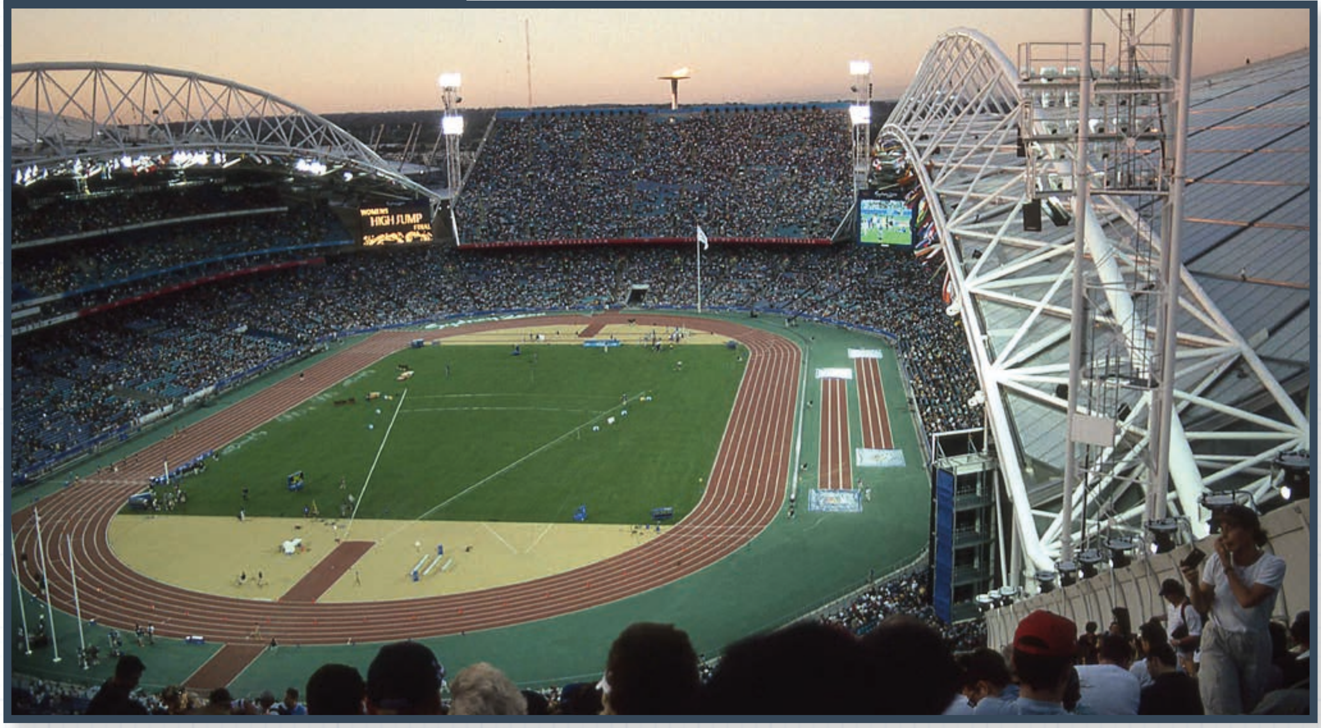

APPLICATION

Securing temporary seating to supporting steel structure

LOCATION

Sydney, Australia

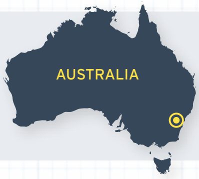

HEAD TYPE

Hexagonal & Countersunk

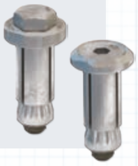

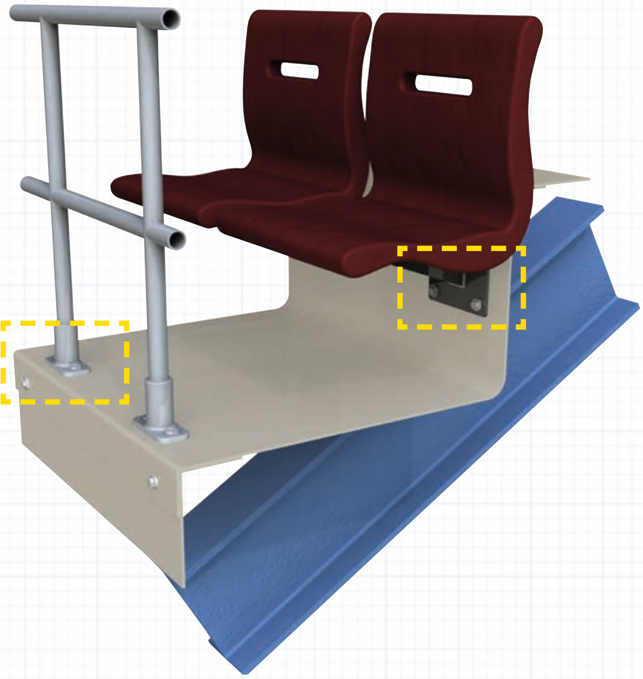

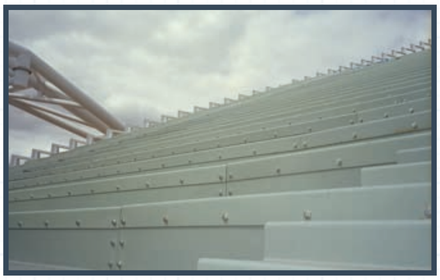

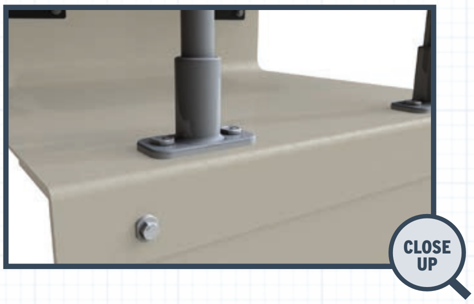

HEXAGONAL & COUNTERSUNK (BOLT HEAD)

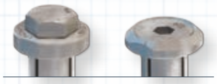

DRILLING & PREPARATION

Ensure that holes are drilled in both the fixture and the section according to the drilling guidance below.
Please note that clearance holes are slightly larger than standard bolt clearance holes to accommodate the sleeve and cone.

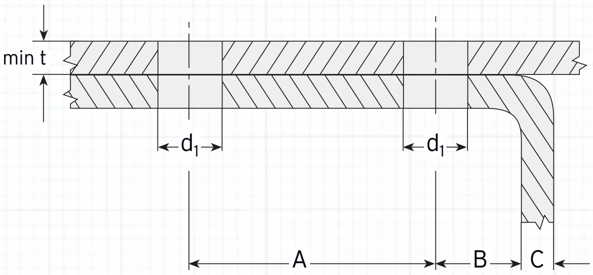

Size	Clearance Hole Ø d1 mm	Hole Distances min A mm	min B mm	Edge Distances B+C mm
M8	14 (+1.0 / -0.2)	35	13	B + C > 17.5
M10	18 (+1.0 / -0.2)	40	15	B + C > 22.5
M12	20 (+1.0 / -0.2)	50	18	B + C > 25.0
M16	26 (+2.0 / -0.2)	55	20	B + C > 32.5
M20*	33 (+2.0 / -0.2)	70	25	B + C > 33.0

* Hexagonal Head only

Sizes M16 and M20 require the thickness of the outer ply (min t) to be at least 8mm. If necessary, spacer washers should be used beneath the collar to increase the thickness to 8mm.

INSTALLATION

1. **Align pre-drilled fixture and section and insert Hollo-Bolt [a].**
2. **Grip the Hollo-Bolt collar with an open ended spanner.**
3. **Using a calibrated torque wrench, tighten the central bolt to the recommended torque [b].**

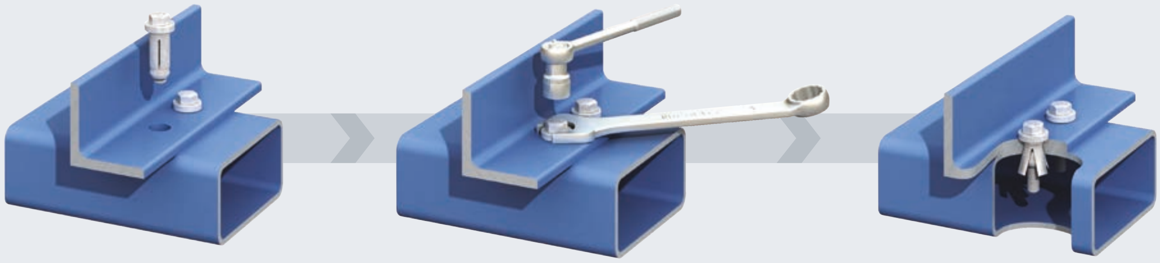

a) Before tightening, ensure that the materials that are to be connected together are touching.
b) See page 22 for tightening torque.
Power tools, such as an impact wrench, may be used to speed up the tightening of the Hollo-Bolt. However, when using power tools, always complete the tightening process with a torque wrench to ensure the correct torque is applied to the Hollo-Bolt.

FLUSH FIT

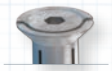

DRILLING & PREPARATION

Ensure that countersunk holes are drilled in the fixture, and standard holes are drilled in the section, according to the drilling guidance below. Please note that clearance holes are slightly larger than standard bolt clearance holes to accommodate the sleeve and cone.

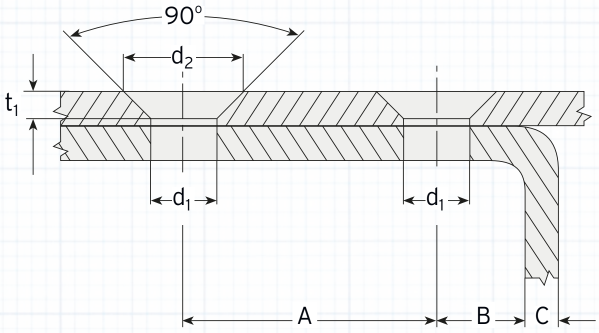

Size	Clearance Hole Ø	Countersunk Ø	Countersunk Depth	Hole Distances		Edge Distances
	d_1	d_2	t_1	min A	min B	B+C
	mm	mm	mm	mm	mm	mm
M8	14 (+1.0 / -0.2)	27	6.5	35	13	B + C > 17.5
M10	18 (+1.0 / -0.2)	31	6.5	40	15	B + C > 22.5
M12	20 (+1.0 / -0.2)	35	7.5	50	18	B + C > 25.0

INSTALLATION

1. **Align pre-drilled fixture and section and insert Hollo-Bolt[a].**
2. **Apply installation nut and grip with an open ended spanner.**
3. **Using a calibrated torque wrench, tighten the central countersunk bolt to the recommended torque[b].**

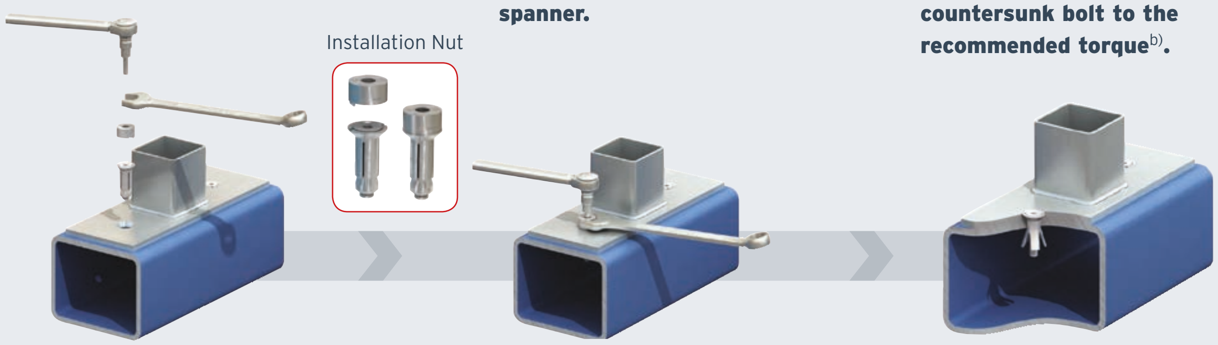

a) Before tightening, ensure that the materials that are to be connected together are touching.
b) See page 22 for tightening torque.
Power tools, such as an impact wrench, may be used to speed up the tightening of the Hollo-Bolt. However, when using power tools, always complete the tightening process with a torque wrench to ensure the correct torque is applied to the Hollo-Bolt.

a HEXAGONAL

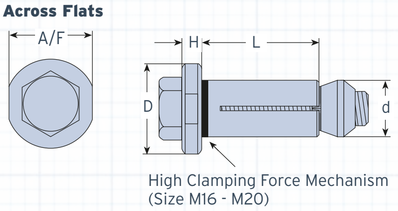

b COUNTERSUNK (BOLT HEAD)

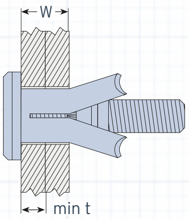

c FLUSH FIT

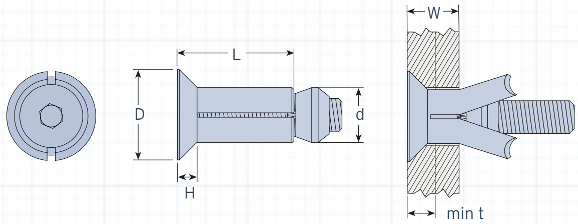

	a HEXAGONAL Product Code	a HEXAGONAL Bolt (mm)	b COUNTERSUNK Product Code	b COUNTERSUNK Countersunk Bolt (mm)	Clamping Thickness W (mm)	Outer Ply[1] min t (mm)	Sleeve Length L (mm)	Sleeve Outer Ø d (mm)	Collar Height H (mm)	Collar Ø D (mm)	Collar A/F (mm)	Tightening Torque (Nm)	Safe Working Loads[2] (5:1 Factor of Safety) Tensile (kN)	Safe Working Loads[2] (5:1 Factor of Safety) Single Shear (kN)
	HB08-1	M8 x 50	HBCSK08-1	M8 x 50	3 - 22	-	30							
	HB08-2	M8 x 70	HBCSK08-2	M8 x 70	22 - 41	-	49	13.75	5	22	19	23	4.0	5.0
	HB08-3	M8 x 90	HBCSK08-3	M8 x 90	41 - 60	-	68							
	HB10-1	M10 x 55	HBCSK10-1	M10 x 50	3 - 22	-	30							
	HB10-2	M10 x 70	HBCSK10-2	M10 x 70	22 - 41	-	48	17.75	6	29	24	45	8.5	10.0
	HB10-3	M10 x 90	HBCSK10-3	M10 x 90	41 - 60	-	67							
	HB12-1	M12 x 60	HBCSK12-1	M12 x 55	3 - 25	-	35							
	HB12-2	M12 x 80	HBCSK12-2	M12 x 80	25 - 47	-	57	19.75	7	32	30	80	10.5	15.0
	HB12-3	M12 x 100	HBCSK12-3	M12 x 100	47 - 69	-	79							
High Clamping Force (HCF)	HB16-1	M16 x 75	HBCSK16-1	M16 x 70	12 - 29	8	41.5							
High Clamping Force (HCF)	HB16-2	M16 x 100	HBCSK16-2	M16 x 100	29 - 50	8	63	25.75	8	38	36	190	21.0	30.0
High Clamping Force (HCF)	HB16-3	M16 x 120	HBCSK16-3	M16 x 120	50 - 71	8	84							
High Clamping Force (HCF)	HB20-1	M20 x 90	-	-	12 - 34	8	50							
High Clamping Force (HCF)	HB20-2	M20 x 120	-	-	34 - 60	8	76	32.75	10	51	46	300	35.0	40.0
High Clamping Force (HCF)	HB20-3	M20 x 150	-	-	60 - 86	8	102							

Sizes M16 and M20, known as the Hollo-Bolt (HCF), feature a patented **High Clamping Force** mechanism to produce three times more clamping force than the same sized product without the mechanism. The significance of clamping force and the superior performance of Lindapter's unique Hollo-Bolt (HCF) is illustrated on page 6.

c FLUSH FIT Product Code	Countersunk Bolt (mm)	Clamping Thickness W (mm)	Outer Ply min t (mm)	Sleeve Length L (mm)	Sleeve Outer Ø d (mm)	Collar Height H (mm)	Collar Ø D (mm)	Installation Nut A/F (mm)	Tightening Torque (Nm)	Safe Working Loads[2] (5:1 Factor of Safety) Tensile (kN)	Safe Working Loads[2] (5:1 Factor of Safety) Single Shear (kN)
HBFF08-1	M8 x 50	10 - 27	8	35							
HBFF08-2	M8 x 70	27 - 45	8	54	13.75	5	24	19	23	4.0	5.0
HBFF08-3	M8 x 90	45 - 64	8	73							
HBFF10-1	M10 x 50	12 - 27	10	36							
HBFF10-2	M10 x 70	27 - 45	10	54	17.75	6	30	24	45	8.5	10.0
HBFF10-3	M10 x 90	45 - 64	10	73							
HBFF12-1	M12 x 55	12 - 30	10	42							
HBFF12-2	M12 x 80	30 - 52	10	64	19.75	7	33	30	80	10.5	15.0
HBFF12-3	M12 x 100	52 - 74	10	86							

1) Sizes M16 and M20 require the thickness of the outer ply (min t) to be at least 8mm. If necessary, spacer washers should be used beneath the collar to increase the thickness to 8mm.
2) The Hollo-Bolt can be used on a wide variety of steel hollow sections; safe working loads shown are based on use in S275 structural hollow section. The safe working loads, in both tension and shear, are applicable to the Hollo-Bolt only. Failure of the section, particularly on those with thin walls and a wide chord face, could occur at a lower figure and strength of the section should be checked by a qualified structural engineer.

The tables above state the safe working loads with a 5:1 factor of safety and should be used for secondary applications. For primary design, please consult the guide Joints in Steel Construction - Simple Connections. The guide provides design guidance for the use of Hollo-Bolt and gives essential information for structural steelwork connections for use in buildings designed by the 'Simple Method' i.e. braced frames where connections carry mainly shear and axial loads only. To obtain further details on the Simple Connections guide please contact:
The Steel Construction Institute Tel: +44 (0) 1344 636 525 / Fax: +44 (0) 1344 636 570 / www.steel-sci.com

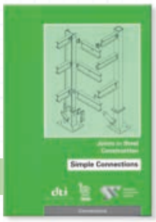

Published by SCI/BCSA Connections Group. Publication Number: P212 / ISBN 1 85942 072 9.

PRETEC
Pretec AS - autorisert distributør: +47 69102460 post@pretec.no www.pretec.no

Characteristic values of tensile and shear resistance for Hollo-Bolt taken from ETA-10/0416. **For more information visit www.lindapter.com/about/ce**

For designing to **Eurocode 3 standard only**

HOLLO-BOLT HEXAGONAL

	Product Code	Nominal Size	Tensile $F_{t,Rk}$ (kN)	Shear $F_{v,Rk}$ (kN)	Material Strength of Sleeve (N/mm²)
	HB08	M8	23.1	32.9	430
	HB10	M10	39.6	54.2	430
	HB12	M12	45.8	71.0	430
HCF	HB16	M16	84.3	139.0	430
HCF	HB20	M20	124.0	211.0	390

HOLLO-BOLT HEXAGONAL STAINLESS STEEL

	Product Code	Nominal Size	Tensile $F_{t,Rk}$ (kN)	Shear $F_{v,Rk}$ (kN)	Material Strength of Sleeve (N/mm²)
	HBST08	M8	26.8	30.7	500
	HBST10	M10	46.0	51.0	500
	HBST12	M12	53.3	65.0	500
HCF	HBST16	M16	98.0	128.0	500
HCF	HBST20	M20	154.0	205.0	500

HOLLO-BOLT COUNTERSUNK (BOLT HEAD)

	Product Code	Nominal Size	Tensile $F_{t,Rk}$ (kN)	Shear $F_{v,Rk}$ (kN)	Material Strength of Sleeve (N/mm²)
	HBCSK08	M8	23.1	32.9	430
	HBCSK10	M10	39.6	54.2	430
	HBCSK12	M12	45.8	71.0	430
HCF	HBCSK16	M16	84.3	139.0	430

HOLLO-BOLT COUNTERSUNK (BOLT HEAD) STAINLESS STEEL

	Product Code	Nominal Size	Tensile $F_{t,Rk}$ (kN)	Shear $F_{v,Rk}$ (kN)	Material Strength of Sleeve (N/mm²)
	HBSTCSK08	M8	26.8	30.7	500
	HBSTCSK10	M10	46.0	51.0	500
	HBSTCSK12	M12	53.3	65.0	500
HCF	HBSTCSK16	M16	98.0	128.0	500

Sizes M16 and M20, known as the Hollo-Bolt (HCF), feature a patented **High Clamping Force** mechanism to produce three times more clamping force than the same sized product without the mechanism. The significance of clamping force and the superior performance of Lindapter's unique Hollo-Bolt (HCF) is illustrated on page 6.

HOLLO-BOLT FLUSH FIT

Product Code	Nominal Size	Tensile $F_{t,Rk}$ (kN)	Shear $F_{v,Rk}$ (kN)	Material Strength of Sleeve (N/mm²)
HBFF08	M8	23.1	32.9	430
HBFF10	M10	39.6	54.2	430
HBFF12	M12	45.8	71.0	430

HOLLO-BOLT FLUSH FIT STAINLESS STEEL

Product Code	Nominal Size	Tensile $F_{t,Rk}$ (kN)	Shear $F_{v,Rk}$ (kN)	Material Strength of Sleeve (N/mm²)
HBSTFF08	M8	26.8	30.7	500
HBSTFF10	M10	46.0	51.0	500
HBSTFF12	M12	53.3	65.0	500

HOLLO-BOLT BUTTON HEAD / SECURITY

* Please contact Lindapter to discuss the available options.

Product Code	Nominal Size	Tensile $F_{t,Rk}$ (kN)	Shear $F_{v,Rk}$ (kN)	Material Strength of Sleeve (N/mm²)
HBBH/HBFT/HBPR	M8	23.1	32.9	430
HBBH/HBFT/HBPR	M10	39.6	54.2	430
HBBH/HBFT/HBPR	M12	45.8	71.0	430

The characteristic values for the Hollo-Bolt listed in the above tables are for use when designing bolted connections to Eurocode 3 only, these are not standard safe working loads.

Hollo-Bolt lengths 1, 2 and 3 are covered by this ETA 10/0416. The characteristic values are used to determine the design resistance of the Hollo-Bolt. The design resistance is calculated by dividing the characteristic value by a partial factor γm2. The partial factor is a nationally determined parameter (for example: γm2 =1.25 in the UK). For Hollo-Bolt safe working loads with a factor of safety of 5:1 please refer to the Hollo-Bolt tables on Page 22 of this brochure.

The characteristic values are valid for the Hollo-Bolt assembly itself, in any connection detail the design resistance of the connection may be limited to a lesser value. For example, when the thickness of the connected component is small, pull out failure may occur before failure of the Hollo-Bolt.

Design checks should be carried out on the section member to determine the static design resistance. The SCI Greenbook publication P.358 Joints in Steel construction, Simple Joints to Eurocode 3 contains a number of checks on the section. The characteristic values are only valid when the Hollo-Bolts are installed as per our installation instructions.

The SCI Greenbook publication P.358 Joints in Steel construction, Simple Joints to Eurocode 3 contains a number of checks on the section. The characteristic values are only valid when the Hollo-Bolts are installed as per our installation instructions. To obtain further details on the Simple Connections guide please contact:
The Steel Construction Institute Tel: +44 (0) 1344 636 525 / Fax: +44 (0) 1344 636 570 / www.steel-sci.com

Published by SCI/BCSA Connections Group. Publication Number: P358 / ISBN 978-1-85942-201-4.

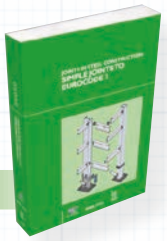

PRETEC
Pretec AS - autorisert distributør: +47 69102460 post@pretec.no www.pretec.no

YOUR QUESTIONS ANSWERED...

Can Lindapter Hollo-Bolts be used in all sizes & shapes of SHS?

Yes, the Lindapter Hollo-Bolt can be used in all sizes of Structural Hollow Section (SHS) and is suitable for use in those of square, rectangular, circular or elliptical shape.

The capacity figures for the Lindapter Hollo-Bolts shown in both SCI 'Green Books' are different to the figures shown in the Lindapter catalogue. Which figures should I use?

The loads shown on page 22 of this brochure are Safe Working Loads, with Lindapter's typical Factor of Safety of 5:1, and are for general use.

For structural use, the loads shown in the SCI design guides are not Safe Working Loads, they are Design Capacities, to be compared in calculations with the structural capacity of the supporting column wall (SHS).

Who is responsible for checking the capacity of the structural section when using Lindapter Hollo-Bolts?

It is the responsibility of a Structural Engineer to ensure a hollow section has sufficient capacity to take the necessary loads. Help can be found within either of the current SCI/BCSA 'Green Books', where P.212 should be used if designing simple connections to BS5950 whilst P.358 should be used if designing simple joints to Eurocode 3.

How does the HCF mechanism increase clamping force?

Without the HCF mechanism on the larger sizes (M16 & M20), the majority of the preload in the bolt is transferred into expanding the sleeve. Lindapter's patented HCF mechanism in the 5-part Hollo-Bolt (HCF) allows the sleeve to expand and converts some of the preload into clamping force to hold the connection securely together.

What is the significance of increased clamping force?

Clamping force is the compressive force which holds the connection together. An M16 or M20 connection using the 5-part Hollo-Bolt (HCF) will be held together with a greater force than a 3-part product of the same size, and have less movement at safe working load. With the 5-part Hollo-Bolt (HCF), a higher load is needed initially to pull the connection apart.

Why don't you make all Hollo-Bolts to the 5-part design?

The M16 & M20 sized Hollo-Bolt (HCF) was designed specifically for larger structural connections that require high clamping force. The Hollo-Bolt M8, M10 & M12 are not generally used for structural joints and adding the HCF mechanism to these smaller sizes would not create a significant advantage when compared to the superior performance of the M16 and M20 Hollo-Bolt (HCF).

Why is there some displacement, even on the Hollo-Bolt (HCF)?

The Hollo-Bolt is a ductile connection and the chord face of the hollow section can deflect. The Safe Working Load for the Hollo-Bolt has been set at an area of minimal displacement (please view the Load/Displacement graphs on page 7).

Can Hollo-Bolts be used in slotted holes?

Yes, it is possible to use Hollo-Bolts with slotted holes in the outer bracket or end plate as long as there is no horizontal load in the direction of the slot. However, the hole in the hollow section into which the Hollo-Bolt is to be installed must be circular and within the tolerance stated in this brochure.

Can Lindapter Hollo-Bolts be sealed to prevent water ingress?

Yes. Although the vast majority of Lindapter Hollo-Bolts used globally do not use any sealing method, special washers have been supplied on a limited number of occasions. However, it is important not to ignore the interface between the structural tube and plate or bracket which is being attached.

Can I use stainless steel Hollo-Bolts to connect brackets to mild steel hollow section?

Where possible the best option is to ensure that the section, bracket and Hollo-Bolt are all produced from the same material, or are close to each other on the galvanic corrosion chart. If stainless components are in contact with mild steel, bimetallic corrosion will be accelerated.

Can I use Hollo-Bolts in concrete filled sections?

The Hollo-Bolt was designed for connecting to structural sections and needs an obstacle free area for the sleeve to expand. Once the component is installed correctly the section can then be filled with concrete.

@ IF YOU HAVE ANY FURTHER QUESTIONS PLEASE CONTACT enquiries@lindapter.com

Are Lindapter Hollo-Bolts removable?

Yes. Although designed as a permanent connection, it is possible to remove the Hexagon and Countersunk (Bolt Head) variants by following this procedure:

1. Grip the Hollo-Bolt collar with an open ended spanner / wrench to prevent the collar from rotating.
2. Use an impact wrench / torque wrench to remove the bolt (anti-clockwise rotation).
3. Hollo-Bolt sizes M8, M10 & M12 only: remove the sleeve by prying the collar with a pinch or crow bar. Note: this additional step is not required to remove the M16 & M20 due to their 5-part design.

The special Security Button Head Hollo-Bolt is designed so that it cannot be easily removed without the Security Key.

Can I use the Hollo-Bolt to connect timber to steel?

Yes, although it is important to ensure that the timber is capable of withstanding the clamping force created when applying torque to the Hollo-Bolt. In some cases a spreader washer can be used under the collar of the Hollo-Bolt to distribute the force over a greater area.

Why aren't all the head variants available in Hot Dip Galvanised finish?

When components with a hexagon socket are Hot Dip Galvanised, the high build up of zinc in the recess results in a reduced A/F dimension meaning that a standard Allen/Hexagon Key no longer fits correctly. This would make it very difficult for the installer to apply the required torque to ensure the Hollo-Bolt expands correctly.

PRETEC Pretec AS - autorisert distributør: +47 69102460 post@pretec.no www.pretec.no

TECHNICAL SUPPORT

The comprehensive technical support from Lindapter's experienced engineers ensures an efficient specification process with a free connection design service and bills of materials upon request. Lindapter's philosophy is to deliver the highest quality at every stage of the service, from initial connection design to installation guidance.

- Free connection design based on your requirement
- Optimised solution for cost and performance
- Bespoke drawings delivered in 2D and interactive 3D formats
- CAD files for import into major software applications
- Contractor training

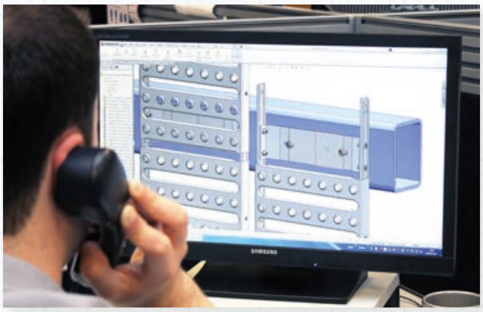

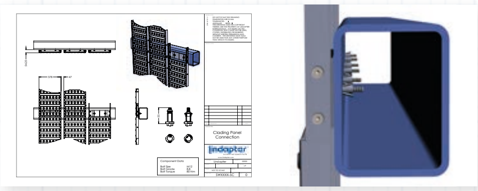

ENGINEERED SOLUTIONS

Lindapter's unique R&D capability facilitates a bespoke product development service, passionately referred to as 'Engineered Solutions'.

The service offered to clients includes:

- Design and development of customised products
- Full strength and performance analysis
- Thoroughly tested with detailed reports
- Manufactured to Lindapter's exacting standards

R&D Facility >
One of two 1000 kN testing machines

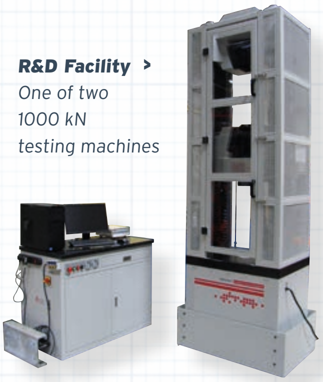

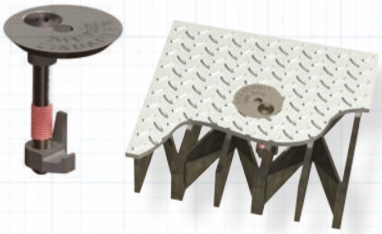

^ Type 1055
Bespoke product designed to fit solid plate flooring to open grid flooring for Amec/Shell

LINDIBOLT® 2

CE

In addition to the Hollo-Bolt, Lindapter also invented the Lindibolt. The self heading expansion bolt is suitable for connecting steelwork to hollow-sections, tubes and where access is available from one side only. The Lindibolt 2 uses a standard clearance hole for convenience.

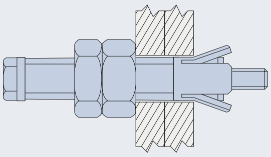

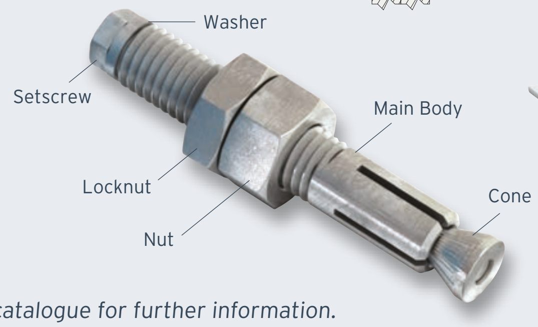

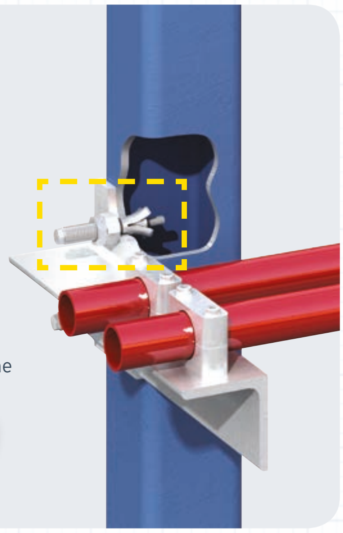

Please refer to the full Lindapter catalogue for further information.

PRETEC
Pretec AS - autorisert distributør: +47 69102460 post@pretec.no www.pretec.no

FULL PRODUCT RANGE

LINDAPTER CATALOGUE

AVAILABLE NOW!

WHAT'S INSIDE?

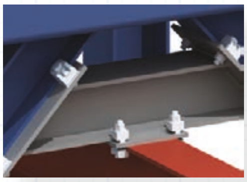

STEELWORK FIXINGS

Lindapter has pioneered a unique & proven concept: innovative clamping systems that eliminate the need to weld or drill, reducing installation time & labour costs.

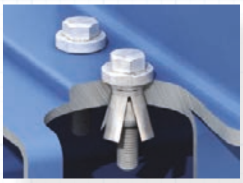

CAVITY FIXINGS

The range consists of the legendary Hollo-Bolt and Lindibolt, creating simple, cost-effective connections for SHS and other hollow sections.

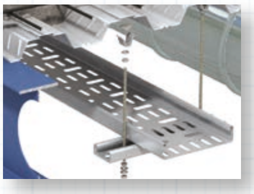

COMPOSITE DECKING FIXINGS

Easy to install connections to fit inside the dovetail shaped re-entrant channel of all major decking profiles. Ideal for supporting HVAC equipment and cable trays without weakening the decking profile.

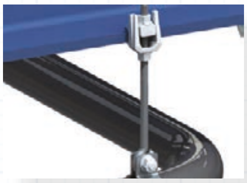

SUPPORT FIXINGS

Lindapter provides a wide range of connection solutions for suspending building services, such as pipe work, sprinklers & suspended ceilings, from structural or supporting steel.

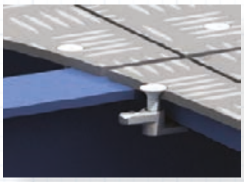

FLOOR FIXINGS

Lindapter's unique no-weld no-drill concept extends to the connection of steel flooring. Open bar grating & chequer plate flooring can be installed by one person from above.

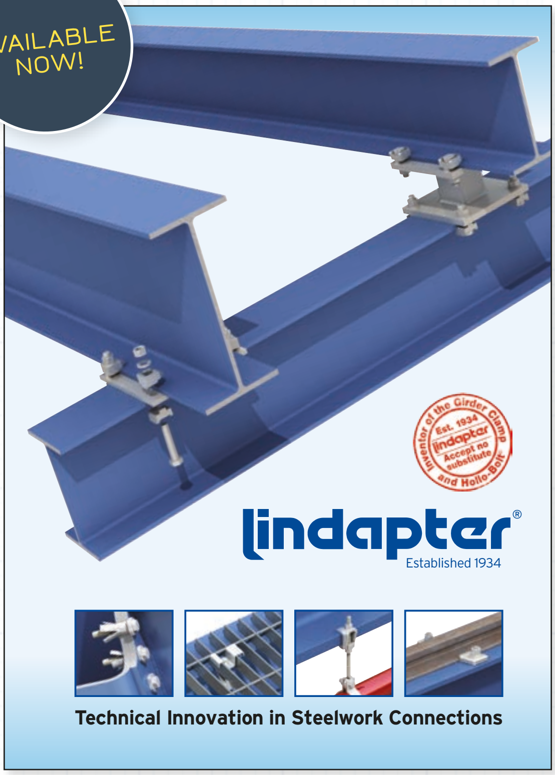

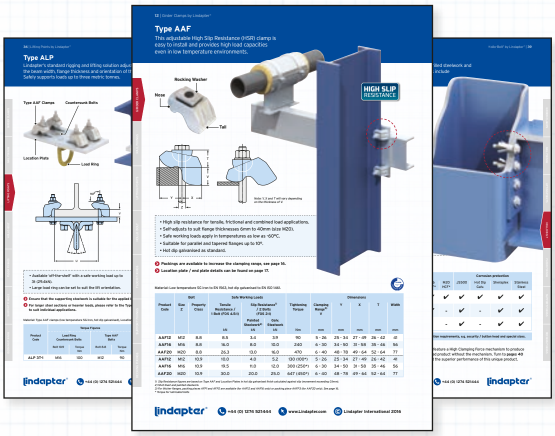

Request your copy today by contacting **enquiries@lindapter.com**

PRETEC
Pretec AS - autorisert distributør: +47 69102460 post@pretec.no www.pretec.no

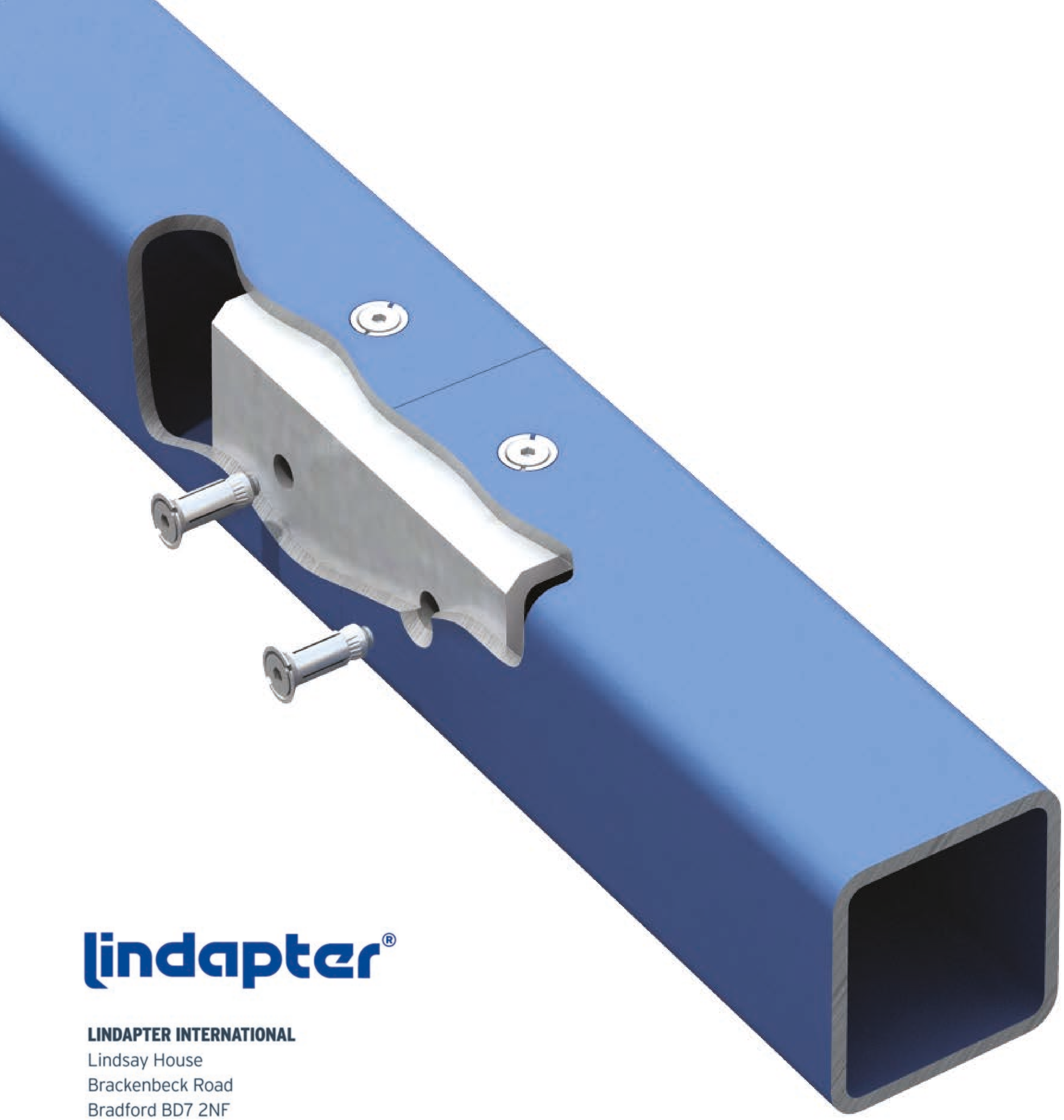

lindapter®

LINDAPTER INTERNATIONAL
Lindsay House
Brackenbeck Road
Bradford BD7 2NF
England
T: +44 (0) 1274 521444
F: +44 (0) 1274 521 130
E: enquiries@lindapter.com
www.lindapter.com

PRETEC
Pretec AS - autorisert distributør: +47 69102460 post@pretec.no www.pretec.no
